# Promises and pitfalls of long-read sequencing for resolving microbial complexity

**DOI:** 10.1093/gigascience/giag087

**Published:** 2026-08-17

**Authors:** Xing Rao, Yuzheng Gu, Gabriella, Jiayi Ma, Haoyu Wang, Yuanqiang Zou

**Affiliations:** State Key Laboratory of Genome and Multi-omics Technologies, BGI Research, Shenzhen 518083, China; College of Life Science, University of Chinese Academy of Sciences, Beijing 100049, China; State Key Laboratory of Genome and Multi-omics Technologies, BGI Research, Shenzhen 518083, China; Key Laboratory of Agricultural and Environmental Microbiology, Ministry of Agriculture and Rural Affairs, Department of Microbiology, College of Life Sciences, Nanjing Agricultural University, Nanjing 210095, China; State Key Laboratory of Genome and Multi-omics Technologies, BGI Research, Shenzhen 518083, China; Department of Food Science and Biotechnology, Faculty of Agricultural Technology, Universitas Brawijaya, Malang 65145, Indonesia; State Key Laboratory of Genome and Multi-omics Technologies, BGI Research, Shenzhen 518083, China; College of Life Science, University of Chinese Academy of Sciences, Beijing 100049, China; State Key Laboratory of Genome and Multi-omics Technologies, BGI Research, Shenzhen 518083, China; College of Life Science, University of Chinese Academy of Sciences, Beijing 100049, China; State Key Laboratory of Genome and Multi-omics Technologies, BGI Research, Shenzhen 518083, China; Shenzhen Key Laboratory of Human commensal microorganisms and Health Research, BGI Research, Shenzhen 518083, China

**Keywords:** long-read sequencing, microbial genomics, metagenomics, genome assembly

## Abstract

Long-read sequencing (LRS) has driven a transition in microbial genomics, overcoming the assembly fragmentation inherent to short-read sequencing. This review elucidates the impact of LRS across isolate genomics, metagenomics, and multi-omics domains. By spanning extensive repetitive regions, LRS facilitates the reconstruction of circular chromosomes and precisely resolves mobile genetic elements (MGEs). In metagenomics, LRS enables strain-level resolution, the recovery of circular metagenome-assembled genomes, and the precise localization of MGEs within host replicons. Furthermore, the single-molecule, amplification-free properties of LRS provide enhanced resolution of native epigenetic modifications and full-length transcriptomes. Despite these advancements, widespread implementation remains constrained by multidimensional challenges, including stringent high-molecular-weight DNA requirements, depth deficits, and computational overhead. Nevertheless, LRS is increasingly becoming the method of choice for isolate genomics and metagenomics. As detection technologies and algorithms progress, LRS will further improve our ability to decipher the structural and functional diversity of microbial ecosystems.

## Introduction

The evolution of sequencing technologies has improved our ability to resolve structural complexity in microbial genomes. From Sanger sequencing to single-molecule real-time platforms, each technical transition reflects improvements in throughput, cost efficiency, and analytical scope. Sanger sequencing, characterized by dideoxynucleotide chain termination, enabled the first genome draft assembly of a cultured microorganism, *Haemophilus influenzae*, marking the formal entry of microbiology into the genomic era [1, 2]. However, throughput constraints necessitated the development of short-read sequencing (SRS) technologies, such as Illumina, DNA nanoball sequencing (DNBSEQ), and Ion Torrent [3–5]. By integrating bridge amplification with sequencing-by-synthesis, SRS circumvented the need for microbial cultivation and supported landmark metagenomic initiatives like the Human Microbiome Project and METAgenomics of the Human Intestinal Tract [6–8].

Despite these advances, SRS reads remain short relative to many microbial genomic features [9]. This limitation is particularly evident in 3 contexts. First, repetitive elements, including ribosomal RNA (rRNA) operons and insertion sequences (ISs), can constitute up to 10% of some bacterial genomes, and their length may exceed the resolving capacity of SRS algorithms [10–12]. Second, for mobile genetic elements (MGEs), such as plasmids and prophages, shared sequences and integration sites are difficult to assign from short reads alone [13–15]. Third, extreme guanine–cytosine (GC) content may introduce coverage bias and lead to the underrepresentation or loss of affected regions [16–18].

In response to these challenges, LRS platforms such as PacBio, Oxford Nanopore Technologies (ONT), and CycloneSEQ generate substantially longer reads and preserve broader genomic context [19–21]. These features have expanded the analytical scope of microbial genomics. However, current reviews of LRS have focused mainly on biomedical surveys or algorithmic analyses [22–24]. For instance, Agustinho et al. provided a comprehensive synthesis of computational pipelines for long-read metagenomic assembly and taxonomic characterization, serving as an invaluable methodological reference [25]. Nevertheless, that review was primarily methodological and centered on metagenomics. Our review systematically covers the reconstruction of complete isolate genomes, the resolution of metagenomic populations with host–element linkages, and the characterization of native epigenomic and full-length transcriptomic landscapes. We also offer an evaluation of the technical limitations of LRS relative to SRS. Together, these perspectives clarify the role of LRS in resolving the genomic and functional diversity of microorganisms.

## Genomics of isolates: reshaping the standard of “reference genomes”

**Figure 1:**
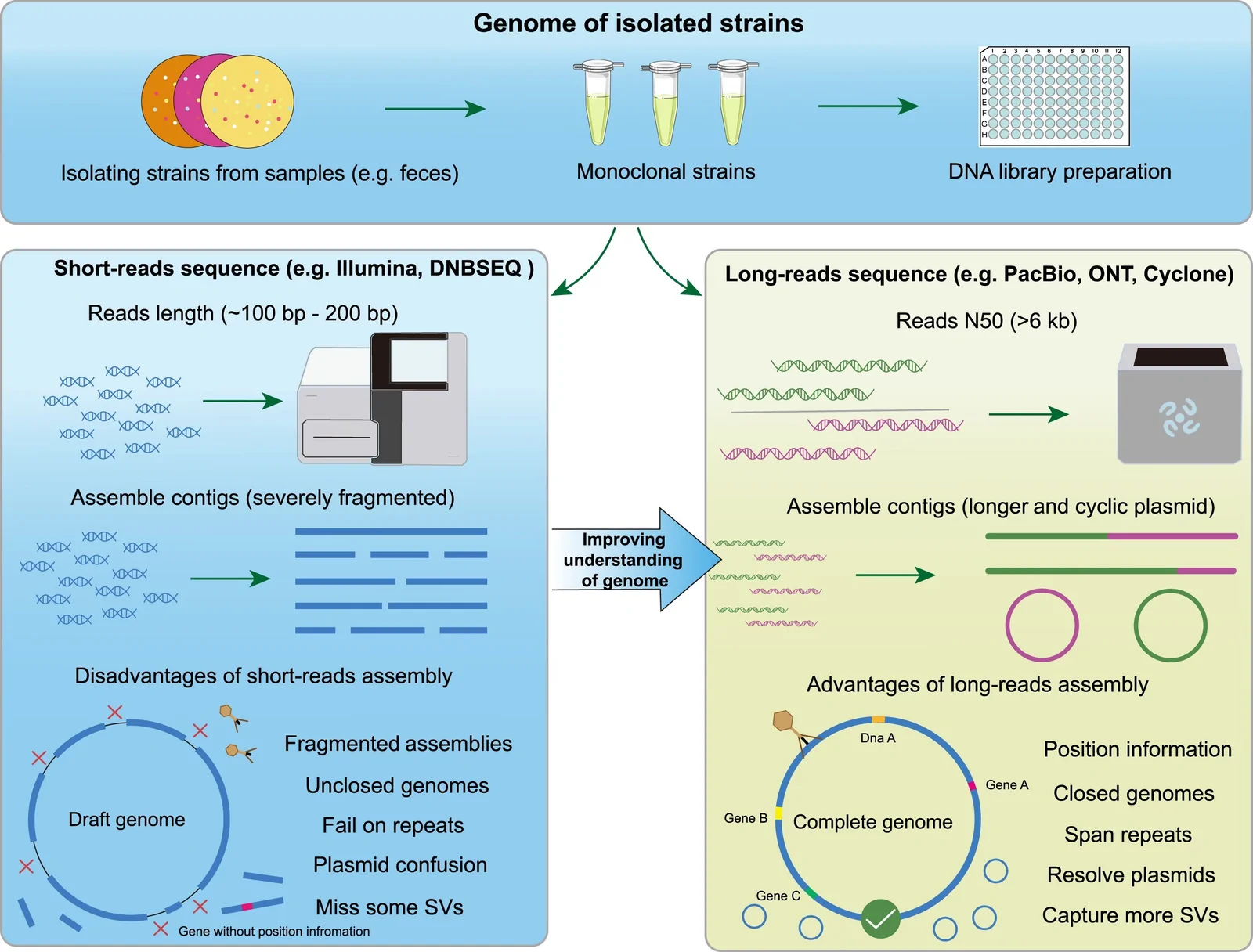
Comparative assembly of isolate microbial genomes using SRS and LRS. Conventional SRS assemblies may incompletely resolve complex repetitive elements, extrachromosomal plasmids, and structural variations (SVs). Conversely, LRS provides longer reads to yield complete genomes supporting plasmid resolution, and comprehensive SV detection for downstream genomic interpretation.

The standard for high-quality reference genomes has expanded beyond base accuracy to requiring structural completeness (Fig. 1). This change is driven by broader access to analytical tools and reduced sequencing costs [26–29]. Historically, isolate microbial reference genomes relied heavily on SRS platforms [30]. SRS assembly commonly uses approaches based on de Bruijn graphs, in which short reads are decomposed into *k*-mers and reconstructed by connecting overlapping *k*-mers [26, 27, 31]. This strategy is computationally efficient, but it becomes ambiguous when complex regions exceed the length of short reads. In contrast, LRS assembly commonly adopts an overlap layout consensus (OLC) approach, in which longer reads are compared directly and ordered according to overlaps between reads to generate a consensus sequence. This enables more reliable reconstruction of complete chromosomal genomes and extrachromosomal elements, including plasmids and circular phage DNA [32].

### Achieving gapless genomes: from fragmentation to continuity

The commercialization of LRS platforms, initiated by PacBio SMRT in 2011 and followed by ONT MinION in 2014, represented a milestone in third-generation sequencing technologies [33]. By spanning genomic regions that are difficult to resolve with SRS, these platforms made complete genome assembly of isolates more feasible (Fig. 1 and Table 1).

**Table 1:** Studies demonstrating the implementation of LRS for isolate microbial genome reconstruction (2013–26)

Year	Study	Platform strategy	Isolate(s)/dataset	Main contribution to genome quality
2013	Koren et al.	PacBio RS	6 bacterial genomes + 2,267 reference genomes	LRS reduced assembly complexity, showing over 70% of known bacterial and archaeal genomes could be finished using a single PacBio library
2013	Chin et al.	PacBio RS SMRT	*Escherichia coli* K-12 MG1655*, Meiothermus ruber* DSM1279*, Pedobacter heparinus* DSM2366	Demonstrated non-hybrid finishing from a single long-insert library improved resolution of long repeats, and consensus accuracy exceeding 99.999%
2015	Loman et al.	ONT MinION	*Escherichia coli* K-12 MG1655	Produced a single 4.6 Mb contig with 99.5% nucleotide identity, providing early proof that nanopore-only data could recover complete chromosome structure
2017	Wick et al.	ONT MinION + Illumina HiSeq	12 *Klebsiella pneumoniae* isolates	Multiplex MinION sequencing with hybrid assembly could enable a scalable, automated bacterial genome closure with fully resolved assemblies
2017	Teng et al.	PacBio RS II + Illumina HiSeq	*Burkholderia pseudomallei*	PacBio resolved the high-GC, 2-chromosome genome with >99.9% identity, while Illumina assemblies remained fragmented
2017	George et al.	ONT MinION + Illumina	8 *Enterobacteriaceae* isolates	Chromosomal contigs matched >95 % of the reference chromosomal sequence length. Plasmid reconstruction recovered 70% and increased to 78% with combined assembler strategy
2019	De Maio et al.	Illumina + ONT MinION or PacBio RS	20 *Enterobacteriaceae* isolates	LRS improves the reads on repeats, plasmid, and complex regions. In hybrid workflows with Illumina data, it enables more complete, accurate, and automation-friendly bacterial genome assemblies
2019	Sydenham et al.	Illumina MiSeq + ONT MinION	6 *Bacteroides fragilis* isolates	Generated complete circular assemblies of all isolates, identified 11 circular plasmids, and resolved complete insertion sequences upstream of AMR genes
2022	Sereika et al.	ONT R10.4	Pure cultures	Assembled near-finished microbial genomes without polishing, reaching 99% accuracy
2024	Sanderson et al.	ONT R10.4.1	13 bacterial isolates	Updated chemistry and ≥40× coverage enabled accurate bacterial reconstruction using just LRS reads
2024	Luan et al.	ONT R9.4.1 + Illumina MiSeq	15 *Salmonella enterica serovar*	LRS reached 99.95% accuracy for complete genomes, but “near-perfect” 99.9999% required hybrid approach
2025	Fan et al.	DNBSEQ-T5 + CycloneSEQ-WT02	*Lactiplantibacillus plantarum* BGI-J9	A hybrid of SRS and LRS strategy achieved a complete genome assembly with 100% completeness
2025	Abdel-Glil et al.	ONT R10.4.1 + Illumina MiSeq	*Y. Similis* DSM 18211, *K. pneumonia* DSM 30104, *E. coli* ATCC 25922, *E. coli* ATCC 35218, and *A. baumannii* DSM 30007	Demonstrated that nanopore-only assemblies are highly reproducible, though short-read polishing further refines consensus accuracy
2025	Wang et al.	DNBSEQ-T5 + CycloneSEQ-WT02	*Lacticplantibacillus plantarum* BGI-N6	Achieved a complete genome assembly, replacing fragmented draft structure with a fully resolved genome
2025	Xu et al.	ONT R10.4.1	*E. coli* isolates	Provided a high-quality reconstruction of genomes, plasmids, and ARG contexts, yielding 1,016 genomes and 2,647 circular plasmids
2025	Wang et al.	DNBSEQ-T5 + CycloneSEQ-WT02	*Lacticaseibacillus rhamnosus* OF44	Achieved a complete genome assembly of a single-contig with 99.99% completeness and 0.19% contamination
2025	Adams et al.	PacBio HiFi + Illumina NextSeq	*Staphylococcus aureus* 63–2,498	Produced a closed single–contig chromosome assembly (98.62% complete) along with 3 plasmids
2026	Nagy et al.	ONT R10.4.1 + Illumina NovaSeq	92 *Enterobacterales* isolates	Demonstrated similar results from LRS only assemblies to hybrid assemblies when using Autocycler+Medaka assembler
2026	Rao et al.	DNBSEQ-T5 + CycloneSEQ-WT02 (Hybrid)	*Lacticaseibacillus paracasei* BGI-N2	Achieved a complete single-contig genome assembly of 3,102,337 bp, with 46.4% GC content and 2,937 CDs

Chin et al. showed that PacBio SMRT sequencing was able to produce *de novo* microbial genome assemblies with over 99.999% consensus accuracy, demonstrating that complete genomes no longer required labor-intensive finishing strategies [34]. Through an analysis of 2,267 complete bacterial and archaeal genomes, Koren et al. found that most microbial genomes could be closed using LRS [35]. The advantage of LRS was not limited to contiguity; ultra-long reads exceeding 32 kb enabled sequencing of entire adhesin-encoding genes (>6 kb) and antibiotic biosynthesis loci (>15 kb), preserving the genomic context of functional loci that was difficult to reconstruct with short read data [36]. In *Lactiplantibacillus pentosus* LP309, LRS recovered the complete chromosome and 8 plasmids, enabling the prediction of over 150 probiotic functional genes involved in acid resistance and vitamin synthesis [37]. Similarly, a recent human gut genome collection of 1,150 circular genomes across 199 species-level clusters further demonstrated that complete assemblies substantially improve downstream functional inference [38]. Metabolic models constructed from these complete genomes recovered 4,764 additional reactions relative to draft-based models, the majority of which were transport-related [39]. Collectively, these studies supported a shift in genome quality standards from draft assemblies toward complete microbial reference genomes, reflecting the ability of LRS to recover genome continuity at the chromosomal level and provide a stronger basis for functional and structural analysis.

### Beyond the chromosome: unveiling the full architecture of plasmids and MGEs

Microbial genomes harbor a diverse repertoire of MGEs that drive horizontal gene transfer (HGT), including plasmids, prophages, transposons, ISs, integrons, and integrative and conjugative elements [40]. SRS can identify many MGE markers, but reconstructing the architecture of plasmids and other accessory replicons remains difficult, especially for large plasmids with shared or repetitive regions. A benchmark of 42 bacterial genomes containing 148 plasmids showed that SRS-based approaches frequently failed to recover complete plasmid structures [41, 42]. As a result, accessory genes encoded within these structures, such as antibiotic resistance genes (ARGs) and virulence factors, may be assigned to incomplete or incorrect genomic contexts, reducing the accuracy of downstream analysis [41, 43].

LRS enables plasmids to be reconstructed as individual replicons while preserving the associated MGE context. Jiang et al. reconstructed resistance plasmid architecture using PacBio SMRT sequencing, characterizing pooled conjugative plasmids from multidrug-resistant *Klebsiella pneumoniae* [44]. They identified 124 ARGs and 317 MGEs, and established a relationship network between MGEs and ARGs that described dissemination by mobile elements. Beyond structural resolution, reconstructed plasmids also revealed biological relevance. For instance, McLeod et al. resolved the extensive plasmid complement of *Lactiplantibacillus plantarum* MF1298, yielding 14 complete plasmid sequences, one of which carried functional genes associated with vitamin B12 transformation [45]. Wang et al. showed that resolved plasmid-borne genes included functions related to fructose metabolism, indicating that accessory replicons can contribute directly to host-associated metabolic capacity [38].

### Resolving microbial variations and evolutionary dynamics

LRS technologies facilitate the identification of a broader spectrum of variations (Fig. 1). This includes single nucleotide variants (SNVs), which are single base differences at specific genomic positions, especially when these variants occur within complex genomic architecture. ONT-based benchmarking showed that SRS failed to detect 9–68 SNPs per 1,000 sites in repetitive reference regions. Conversely, by spanning these complex regions, ONT long reads improved SNP calls across previously inaccessible genomic regions [46]. Capturing this broader view is essential because microbial evolution depends not just on massive structural shifts, but also on small, localized mutations and shifting repeat regions [47]. Beyond SNVs, LRS is particularly valuable for resolving structural variants (SVs), which are genomic changes larger than 50 bp, including inversions, duplications, insertions, deletions, and translocations. In *Bordetella pertussis*, LRS confirmed both duplications and a large inversion of ∼1 Mbp between isolates from the same patient [48]. Similarly, in *Salmonella enterica* serovar Typhi, LRS confirmed large genome structure rearrangements generated during long-term culture, and isolates with altered genome structures showed transcriptional changes relative to the parental strain [49].

On an evolutionary scale, the value of LRS is reinforced by population-level analyses. In 134 multidrug-resistant *S. enterica* isolates, LRS revealed resistance-associated structural changes, including differences in the presence and orientation of *Salmonella* pathogenicity islands, and chromosomal insertion of resistance plasmids [50]. Canalda-Baltrons et al. constructed a pangenome reference graph from 859 high-quality LRS assemblies of *Mycobacterium tuberculosis* and showed that SVs are not marginal events, but an important component of genome evolution, virulence-associated deletions, and drug-resistance architecture [51]. Their subsequent genotyping across 41,134 isolates linked recurrent SVs to multiple first- and second-line drug resistance phenotypes, underscoring that long-read-enabled SV analysis contributes not only to structural characterization, but also to understanding clinically relevant adaptation. Taken together, these studies show that the key contribution of LRS is not merely improved contiguity, but the ability to recover chromosome-scale structural change with sufficient precision to study its functional and evolutionary consequences.

### Precise localization of genetic elements through complete genomes

The precise localization of MGEs provides a critical window into understanding microbial organisms. Once chromosomes and accessory replicons are fully assembled, genetic elements can be assigned to a defined chromosomal or plasmid backbone. Sheppard et al. applied LRS to 17 blaKPC-positive *Enterobacteriaceae* isolates. This enabled precise localization of blaKPC and its associated transposon Tn4401, clarifying the linked roles of blaKPC-bearing host strain transmission, plasmid transfer across strains and species, and Tn4401 transposition among plasmids in blaKPC dissemination [52]. Beyond resistance spread, complete genome resolution of the probiotic *Lactobacillus crispatus* precisely anchored Tn7088, a novel integrative mobile element encoding a class I bacteriocin biosynthetic gene cluster (BGC) that drives antimicrobial competition and probiotic fitness [53]. At a larger scale, Benler et al. delineated 6,549 Tn7-like transposons at base-pair resolution and showed that 96% carried cargo genes enriched not only in defense and antibiotic resistance functions but also in central carbon metabolism [54]. Together, these findings show that complete genome assemblies both recover more sequence, and reveal the genomic context required to understand mobility and adaptation.

## Genome-resolved metagenomics of microbial communities

**Figure 2:**
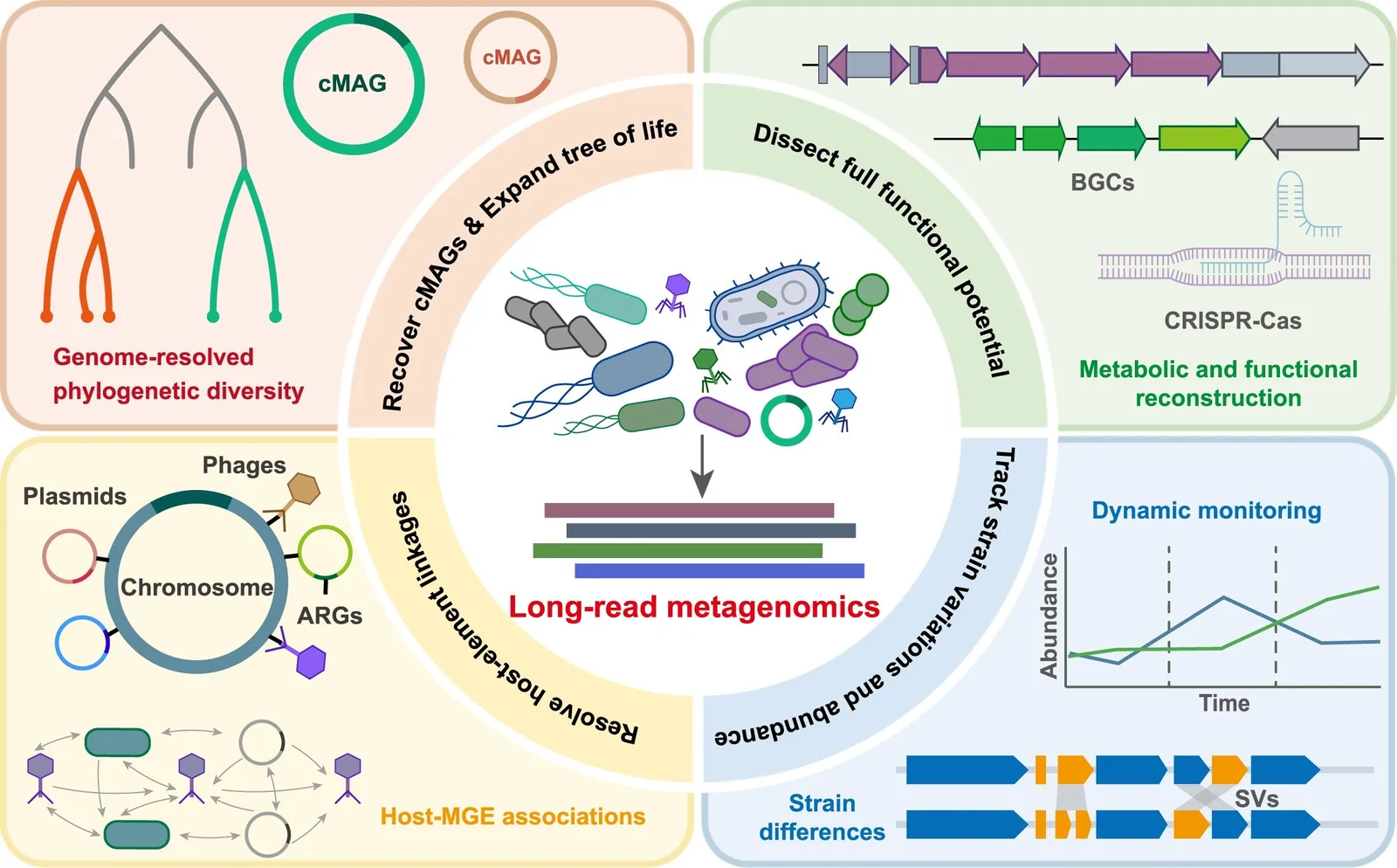
Applications of long-read metagenomics in microbial ecosystems. This schematic delineates the multidimensional analytical capacities unlocked by LRS to overcome the limitations of conventional metagenomics. LRS facilitates the reconstruction of circular metagenome-assembled genomes (cMAGs), expanding genome-resolved phylogenetic diversity by generating complete or near-complete genome representatives that can be placed in phylogenomic frameworks and linked to functional elements. Concurrently, the recovery of intact functional loci, such as BGCs and CRISPR systems, enables robust metabolic and functional profiling. Strain-level resolution further allows for the dynamic monitoring of strains. Furthermore, LRS supports evidence-based host–MGE association by resolving integration junctions and improving phage–host inference, while complementary strategies provide evidence for assigning plasmids, phages, and ARG-bearing elements to likely hosts.

Metagenomics has vastly expanded our understanding of the diversity and metabolic potential of uncultured microorganisms. Yet many ecological and functional questions require more than detecting genes or taxa alone. They depend on knowing which genome carries a given functional locus and what neighboring genes or mobile elements surround it. Recent improvements in the throughput and raw-read accuracy of LRS, together with long-read-aware assemblers, polishers, and binning frameworks, have begun to relax this constraint [25, 55]. These developments enable long-read metagenomics to recover complete or near-complete genomes, distinguish closely related strains, and determine where functional loci and integrated mobile elements occur within assembled genomes (Fig. 2 and Table 2).

**Table 2:** Representative LRS studies for MAG and cMAG recovery from metagenomes

Year	Study	Platform and strategy	Metagenomic dataset	MAG/cMAG recovery
2019	Bertrand et al.	Illumina + ONT nanopore; OPERA-MS hybrid assembly	Mock communities and 28 human gut metagenomes	Improved contiguity ∼200-fold; recovered rare-species genomes and >80 closed plasmid/phage sequences
2020	Moss et al.	ONT MinION R9.4; Lathe workflow with short-read polishing	13 human stool metagenomes	Recovered 20 circular bacterial genomes directly from microbiomes
2021	Singleton et al.	ONT PromethION + Illumina; long-read assembly with short-read support	Activated sludge from 23 Danish WWTPs	Recovered 1,083 HQ MAGs, including 57 closed circular genomes
2022	Bickhart et al.	PacBio HiFi + Hi-C binning; MAGPhase strain-level resolution	Sheep fecal metagenome	Recovered 428 MAGs, including 44 single-contig circular MAGs and 220 lineage-resolved MAGs
2022	Feng et al.	PacBio HiFi; hifiasm-meta assembler	Seven empirical metagenomic datasets	Reconstructed tens to hundreds of complete circular bacterial genomes per dataset
2022	Kim et al.	PacBio HiFi, including Sequel II data; metaFlye/HiCanu/hifiasm-meta plus cMAGfilter	Five human fecal metagenomes	Recovered 102 accurate cMAGs, including uncultured gut taxa
2022	Gounot et al.	Illumina HiSeq4K + ONT MinION/GridION/PromethION; OPERA-MS; Hi-C subset	109 Southeast Asian gut metagenomes	Recovered 4,497 medium/HQ MAGs; 1,708 were absent from short-read-only analysis
2024	Benoit et al.	PacBio HiFi; metaMDBG assembler	Human gut, anaerobic digester, and sheep rumen datasets	Increased HQ circular MAG recovery, with improved plasmid and virus assembly
2025	Sereika et al.	ONT P2/P24, SQK-LSK114/FLO-PRO114M; Flye/Medaka + mmlong2 workflow	154 soil and sediment samples	Recovered 23,843 MAGs, dereplicated into 15,640 species-level MAGs
2026	Benoit et al.	ONT R10.4.1; nanoMDBG assembler	Human gut, Zymo Fecal Reference, and soil metagenomes	Recovered more near-complete MAGs than ONT comparators; showed ONT can approach HiFi MAG recovery at matched depth
2026	Shaw et al.	PacBio HiFi and ONT R10.4; myloasm assembler	Gut, oral, and other metagenomic datasets	Improved strain-resolved assembly and recovery of complete circular contigs

### Circular metagenome-assembled genomes

Complete or near-complete metagenome-assembled genomes (MAGs) recovered directly from microbial communities provide genome-resolved references for metabolic reconstruction, comparative genomics, and ecological interpretation. Early nanopore-based studies showed that closed bacterial genomes could be recovered directly from human fecal metagenomes, indicating that complete community genomes were no longer restricted to isolate sequencing [56]. At population scale, this additional continuity translates directly into a more complete view of microbiome diversity. In a study of Southeast Asian gut microbiomes integrating LRS with Hi-C, a proximity ligation method used to link DNA fragments from the same cell, more than 1,700 MAGs were absent from short-read-only analyses, assembly contiguity improved by more than 28-fold, and thousands of population-specific strains and BGCs were recovered [57]. Similar gains have been reported outside the human gut. In activated sludge, hybrid long- and short-read approaches generated more than 1,000 high-quality MAGs, including dozens of circular genomes, and restored full-length rRNA genes that are typically absent from short-read MAGs [58]. More recently, deep long-read surveys across terrestrial habitats recovered genomes from 15,314 previously undescribed microbial species, expanding the phylogenetic diversity of the prokaryotic tree of life by 8%. These assemblies also recovered thousands of complete rRNA operons, BGCs, and CRISPR-Cas systems [59]. Together, these findings indicate that long-read metagenomics can improve assembly of known taxa, recover poorly represented lineages, and support systematic discovery of repetitive and functionally important elements involved in microbial adaptability and evolution.

### Strain-level resolution

Long-read metagenomics is also redefining strain resolution in microbial communities, particularly when multiple closely related lineages coexist within the same sample. Mechanistically, individual long reads link SNVs, small indels, and SVs over long genomic intervals, thereby preserving haplotypic linkage and improving strain resolution. Long reads can also preserve the genomic context of structurally variable accessory loci, including BGCs, prophages, CRISPR arrays, and IS-flanked islands [25, 60, 61]. Recent algorithmic advances further improve practical access to this resolution. Myloasm uses polymorphic *k*-mers and high-resolution string graphs for modern PacBio HiFi and ONT R10.4 long reads, improving the recovery of complete circular contigs and previously inaccessible within-species diversity in metagenomic datasets [62]. These principles have been demonstrated in animal and human gut microbiomes. In sheep faecal microbiomes, LRS recovered lineage-resolved cMAGs and revealed hidden population structure while improving assignment of BGCs and mobile elements to specific hosts [61]. In the human gut microbiome, long-read analyses further showed that SV is highly individualized and contributes substantially to within-species diversity [60]. Together, these studies show that strain-level interpretation often depends on multi-kilobase genomic segments that are poorly represented in short-read consensus assemblies.

The clinical relevance of this enhanced resolution is becoming increasingly apparent. In fecal microbiota transplantation (FMT), therapeutic interpretation depends not only on whether a species is detected after treatment, but also on which donor strain engrafts and persists in the recipient gut. Long-read metagenomics enables post-FMT strain tracking with substantially greater specificity than short-read approaches, particularly when multiple conspecific strains coexist in the same sample. In cohorts with recurrent *Clostridioides difficile* infection and inflammatory bowel disease, long-read analyses identified hundreds of engrafted donor strains and tracked their persistence for up to 5 years, while also capturing epigenetic stability and large-scale SVs acquired during adaptation to the recipient environment [63]. In a pediatric undernutrition cohort, Minich et al. used long-read metagenomics to follow the gut microbiota over 11 months and linked SV in commensal strains to host growth-associated phenotypes, suggesting that clinically relevant microbiome signals may depend on fine-scale genomic plasticity rather than taxonomic composition alone [64]. These findings indicate that strain resolution should not be viewed as a technical refinement of species-level profiling, but as a shift toward a more appropriate biological scale. Many clinically and ecologically relevant processes, including engraftment, persistence, competition, and adaptation, operate at the level of strains rather than species. Long-read metagenomics therefore offers a framework in which microbial population dynamics can be interpreted in terms of inheritance, selection, and genome plasticity, rather than inferred indirectly from abundance changes alone.

### Establishing host–element linkages

One of the most important contributions of LRS to microbial ecology is its ability to bridge the assembly gap between MGEs and their host chromosomes (Fig. 2). In short-read datasets, researchers can quantify the abundance of these elements, yet still lack direct evidence for their host range or transfer potential. LRS can strengthen host–MGE analysis in 2 distinct ways. For integrated MGEs, including prophages, reads, or contigs that span the element–chromosome junction provide direct sequence evidence for the host genome [25, 65]. In addition, longer viral and bacterial contigs can improve phage–host association by recovering more complete viral genomes, CRISPR arrays, and host taxonomic signals. For example, viralFlye showed that long-read assemblies increased CRISPR-based virus–host predictions and improved host assignment precision relative to short-read assemblies [66]. Extrachromosomal plasmids are often harder to assign because they lack obligatory chromosomal junctions, have mosaic sequence composition, and may circulate across multiple hosts. Therefore, plasmid–host and ARG-bearing MGE assignments in metagenomes often require orthogonal evidence, such as Hi-C/3C proximity ligation, methylation-aware binning, or ultra-long reads when they span integration or cointegration junctions [25, 67].

Beyond host assignment, LRS is reshaping mobilome discovery by recovering complete extrachromosomal elements and resolving their structural diversity. In the human gut, long-read metagenomics enabled direct recovery of complete extrachromosomal MGEs, revealing plasmids and phage-associated DNA that are underrepresented in short-read catalogs [68]. In the oral microbiome, long-read assembly uncovered a family of giant plasmid-like extrachromosomal elements associated with *Streptococcus*, indicating that mobile gene pools in host-associated communities have been substantially underestimated [69]. Long reads have also sharpened analyses of phage ecology. In a 2-year longitudinal study of the human gut microbiome, Wirbel et al. found that prophage–host relationships were more flexible than predicted by a strictly host-specific model: bacterial lineages with and without particular prophages could coexist stably, and some phages were detected in distinct host lineages across family boundaries [15]. The same study identified a class of phages termed “IScream” phages, which appear to exploit bacterial IS30 transposases to mediate their own mobility and integration. These findings demonstrate that the mobilome is more dynamic and cross-lineage than previously appreciated, and that LRS is becoming an important tool for resolving this complexity.

The advantage of LRS is particularly valuable in resistome analysis. In an early proof-of-principle study, combining long-read assembly with proximity ligation substantially increased recovery of ARGs and virus–host associations in the rumen microbiome relative to short-read assembly alone [70]. Works in wastewater treatment plants, activated sludge systems, and urban sewage networks showed that LRS can determine whether specific ARGs are stably chromosome-encoded or carried by conjugative plasmids, while resolving how host associations and mobility profiles change along environmental gradients [71–73]. Long-read datasets have likewise clarified the effects of local selection pressures on MGE dynamics. In hospital wastewater, chlorine disinfection was associated with altered community structure, increased ARG diversity, and higher mobility potential within the resistome [74]. In microplastic-associated biofilms, plasmid-borne ARGs were enriched relative to surrounding water and natural rock substrates, suggesting that artificial polymer surfaces may act as hotspots for plasmid-mediated resistance transfer [75]. Together, these studies show that LRS improves resistome and mobilome analysis by placing functional genes in their genomic background. In short-read datasets, detection of an ARG, virulence gene, or prophage often shows only that the sequence is present. LRS can further indicate whether these loci are located on chromosomes, plasmids, or transposons, which is essential for assessing their host association and potential mobility.

### Pathogen genomics and real-time detection

ONT-based LRS provides a culture-independent route from clinical specimens to pathogen information, making it an important translational extension of genome-resolved microbial genomics. Its distinctive advantage lies in the ability to generate analyzable reads during the run. Reads can be classified during sequencing, allowing pathogen detection and resistance-gene screening to begin before the sequencing run is complete. In lower respiratory infection, Charalampous et al. developed a host-depleted nanopore metagenomic workflow that produced results within 6 h from sample to result, detected bacterial pathogens with 96.6% concordance with culture, and accurately detected antibiotic-resistance genes [76]. In infected body fluids, Gu et al. evaluated clinical metagenomic next-generation sequencing using both Illumina and nanopore platforms. With the nanopore platform and real-time computational analysis, pathogens were identified after a median of 50 min of sequencing within a 6 h sample-to-answer workflow [77]. More recently, Alcolea-Medina et al. developed a unified host-depletion and nanopore workflow for respiratory samples that detected RNA and DNA viruses, bacteria, atypical bacteria, and fungi; generated first automated reports after 30 min of sequencing within a 7 h workflow; and achieved prospective bacterial detection sensitivity and specificity of 90 and 100% after 2 h of sequencing [78]. This study also showed that genome recovery was feasible for a subset of reported organisms after longer sequencing, supporting the potential of nanopore metagenomics to move beyond detection toward strain typing and resistance or virulence analysis when pathogen load and read depth are sufficient [78]. These studies indicate that nanopore-based LRS is most relevant when culture is slow, antibiotic exposure may reduce culture sensitivity, or rapid resistance information is needed. Clinical implementation nevertheless remains constrained by host-background DNA, contamination control, pathogen-load thresholds, database interpretation, genotype–phenotype concordance for antimicrobial resistance, and the need for prospective validation.

## Extended applications: multi-omics beyond DNA sequences

**Figure 3:**
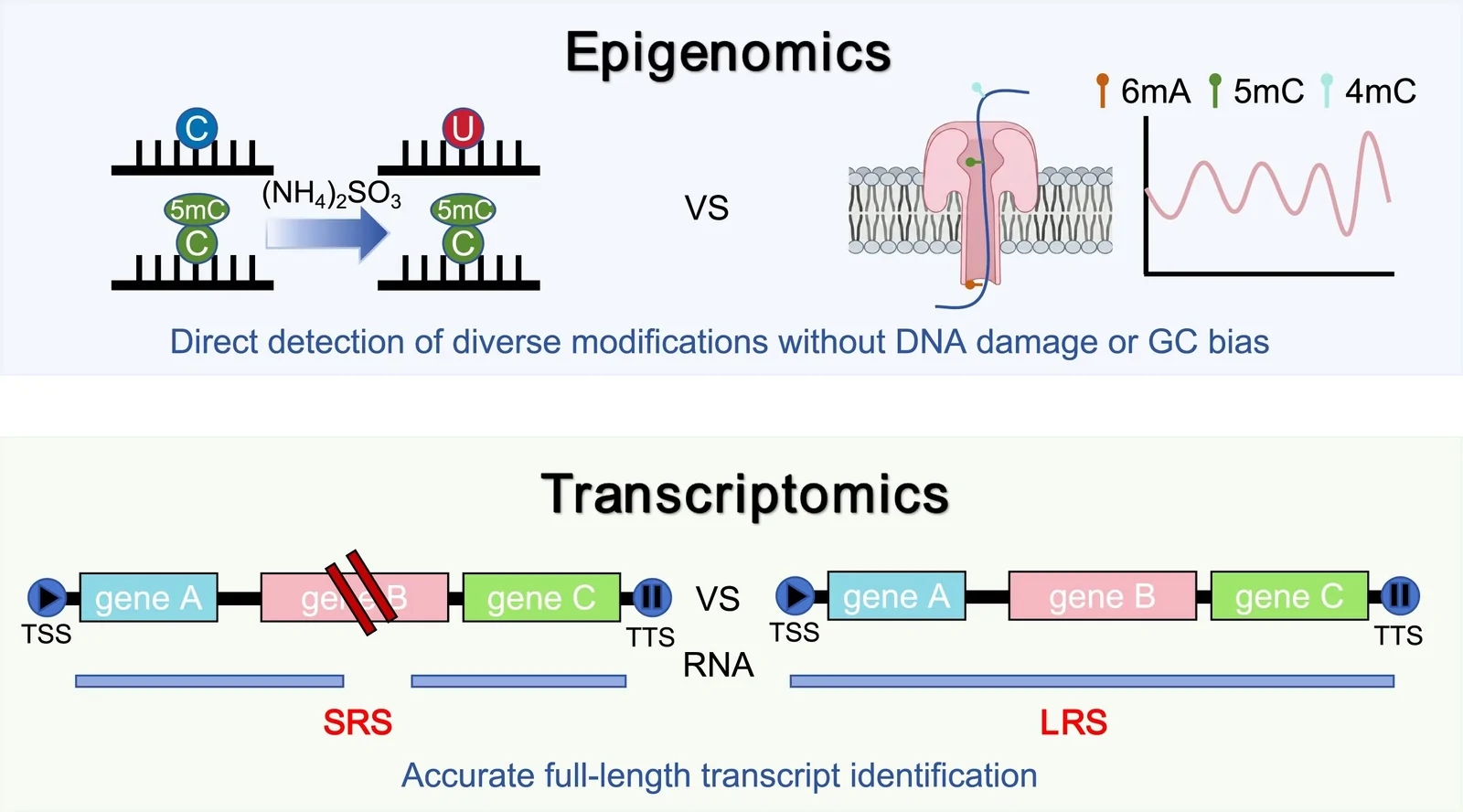
Paradigm shifts in microbial multi-omics driven by single-molecule sequencing. This schematic contrasts the analytical mechanisms of conventional assays against single-molecule technologies across 2 core omics dimensions. Epigenomics: Conventional epigenetic profiling necessitates chemical conversions (e.g., bisulfite treatment), which induce severe DNA damage and sequence bias. In contrast, LRS enables the direct detection of diverse native modifications (such as 6mA, 5mC, and 4mC) via nanopore current disruptions. Transcriptomics: SRS makes it difficult to recover contiguous linkage within polycistronic operons. Conversely, LRS achieves full-length transcript identification, capturing contiguous RNA molecules spanning intact multi-gene organizations from transcriptional start sites (TSSs) to transcriptional termination sites (TTSs).

Genome continuity alone is insufficient to explain microbial phenotypes such as antibiotic resistance, virulence, and environmental adaptability. Epigenetic modifications and dynamic transcriptomic regulation provide additional layers linking genotype to phenotype. SRS presents inherent limitations in these dimensions. This section systematically elucidates the technical advantages and frontier applications of LRS in microbial epigenomics and full-length transcriptomics (Fig. 3).

### Microbial epigenomics: from native signals to regulatory landscapes

Microbial epigenetic modifications, predominantly N6-methyladenine (6mA), 5-methylcytosine (5mC), and N4-methylcytosine (4mC), are associated with restriction–modification (R-M) systems, gene expression, and host–pathogen interactions. Historically, SRS-based approaches for DNA methylation detection have often relied on bisulfite chemical conversion, which can cause DNA degradation and sequence bias. Because methylation profiles are inferred from aggregated sequencing reads, these approaches generally provide population-level methylation profiles rather than direct measurements of individual DNA molecules. In contrast, LRS preserves native DNA molecules and enables direct modification detection: PacBio SMRT utilizes polymerase kinetic characteristics, while ONT detects changes in nanopore ionic current. Both platforms enable simultaneous DNA sequencing and single-molecule methylation detection without PCR amplification or chemical conversion, allowing direct linkage of epigenetic modifications to their genomic context on individual DNA molecules [79, 80] (Fig. 3). Today, LRS is extensively applied to methylation detection, complete R-M system resolution, and the linking of epigenetics to phenotypic regulation. Notably, the 2 platforms exhibit divergent strengths: PacBio HiFi currently provides high sensitivity and confidence for 6mA detection, whereas recent developments, including the R10.4.1 pore and improved basecalling models, have drastically improved 5mC and 4mC detection, making it particularly attractive for cost-effective, large-scale meta-epigenomic studies [81, 82].

Compared with SRS, LRS can associate methylation motifs with cognate methyltransferases across the same long DNA molecules [83]. This precise anchoring allows for the resolution of R-M systems carried by plasmids, revealing their core roles in restriction of HGT and phage defense [84, 85]. Furthermore, LRS maps methylation modifications onto specific functional elements (promoters, operons, and MGEs), identifying potential regulatory sites of single-base modifications on gene expression. In microbial communities, long-read metagenomic methylation profiling improves strain-level resolution and assignment of plasmids and phages to their hosts through shared methylation signatures [86].

However, several challenges remain. Accurate long-read metagenomic methylation analysis depends on the recovery of high-molecular-weight (HMW) DNA, which is required to generate long reads and contiguous assemblies that enable reliable assignment of methylation motifs to chromosomes, plasmids, and phages. In addition, the methylation characterization of low-abundance taxa remains limited by sequencing depth. Ding et al. found that at the 25× sequencing depth recommended by PacBio, only 16.24% of phage genomes met this requirement, and the number of detected methylation sites increased with deeper sequencing [87]. Furthermore, different types of methylation modifications exhibit distinct depth requirements. For instance, 40× depth is sufficient to profile most 6mA sites, whereas 4mC resolution requires at least 60× depth to achieve comparable sensitivity [88].

### Full-length transcriptomics: reshaping operons and isoforms

Bacterial transcriptomes are substantially organized around polycistronic operons and contain a rich repertoire of alternative transcripts, antisense RNAs (asRNAs), and non-coding RNAs that collectively coordinate environmental adaptation, stress responses, and virulence. Traditional short-read RNA sequencing often fragments transcripts, hindering accurate reconstruction of complete transcriptional units (Fig. 3). LRS can generate reads that span entire RNA molecules in a single pass [85, 89].

A major advantage of LRS is its ability to precisely delineate operon architecture, including transcriptional start sites (TSSs), transcriptional termination sites (TTSs), internal promoters, and co-transcribed gene clusters. By linking multiple genes within individual polycistronic transcripts, LRS transforms inferred operon models into experimentally validated maps of active transcription units [90, 91]. Furthermore, LRS provides unambiguous information to accurately annotate regulatory elements such as asRNAs, as well as overlapping genes, which typically pose challenges for short–read–based assembly [92]. For instance, ONT direct RNA sequencing refined the transcriptional boundaries of previously identified operons, revealing extended boundaries for 225 operons in *E. coli* and 89 operons in *S. aureus* [93]. Moreover, direct RNA sequencing enables the detection of native RNA modifications, such as m5C and m6A, providing new insights into the complexity of post-transcriptional regulation in bacteria [94]. In a community context, integrating metatranscriptomics with nanopore long-read metagenomics enabled the reconstruction of trimethylamine metabolic networks and the identification of key microbial taxa [95]. Nevertheless, applying LRS in the microbiome faces several technical challenges. Full–length RNA recovery is difficult due to rapid RNA degradation and inhibitory compounds (e.g., humic acids in soil) [96]. rRNA depletion is another essential step, as rRNA typically constitutes more than 90% of total bacterial RNA [97]. Assignment of transcripts to specific strains represents a third barrier, owing to high sequence similarity among closely related species and the lack of reference genomes [98].

## Multidimensional technical challenges in LRS

As delineated in preceding sections, LRS has promoted a shift across isolate genomics, metagenomics, and multi-omics dimensions, elevating the analytical resolution of microbiology. Nevertheless, despite its exceptional potential to reconstruct genomic architectures, the systematic application of LRS in large-scale sequencing studies remains impeded by cost-effectiveness limitations [22, 99]. Crucially, this economic burden extends far beyond direct sequencing expenses (such as reagents and instruments) [100]; it is rooted in systemic technical limitations that affect the entire experimental workflow (Table 3), spanning sample preparation, data acquisition, and bioinformatic assembly.

**Table 3:** Comparison of short- and long-read sequencing platforms

	SRS		LRS		
	Illumina (NovaSeq X)	MGI (DNBSEQ-T20)	PacBio (Revio)	ONT (PromethION 24)	MGI (CycloneSEQ-G400-ER)
DNA input requirements	100 ng	200 ng	500 ng	1 µg	500 ng
Read length	2 × 150 bp	2 × 150 bp	15–20 kb	20 bp to >4 Mb	100 bp to >1Mb
Raw accuracy	>99.9%	>99.9%	∼99.9%	∼99%	∼97%
Throughput	2 × (8–10.5) Tb	6 × 12 Tb	4 × (100–120) Gb	24 × (100–200) Gb	1 × 400 Gb
Runtime	48 h	24∼30 h	24 h	72 h	72 h

### Sample preparation constraints in LRS

By preserving native nucleic acid information, LRS circumvents the sequence biases and errors introduced by PCR amplification in SRS [101], representing an important advance in genomic research. However, while LRS increases read lengths (Table 3), it simultaneously imposes stringent requirements on upstream sample preparation [22, 102]. This requirement for high purity and HMW DNA is a major technical challenge limiting the accessibility of LRS for complex environmental niches.

First, LRS is sensitive to co-purified impurities. In traditional SRS library construction, the PCR amplification process dilutes easily co-precipitated inhibitors, such as polysaccharides and bile acids in gut samples, or residual extraction reagents [103]. Conversely, the amplification-free nature of LRS allows these contaminants to persist in the final library. These impurities inhibit single-molecule polymerase activity or obstruct nanopores, leading to premature termination of the sequencing reaction [102, 103]. This issue is pronounced in complex environmental samples such as soil, where abundant humic acids, sharing chemical structural similarity with nucleic acids, are readily co-purified. Attempts to remove these impurities through additional purification steps may result in fragmentation of the DNA molecules [104]. Second, the extraction of HMW DNA confronts a fundamental paradox. Even routine extraction procedures, including pipette aspiration, column purification, or mild vortexing, generate physical shear forces to induce the breakage of long DNA fragments [105, 106]. Within microbiome research, this challenge is substantially amplified. Due to the robust peptidoglycan architecture characteristic of many bacteria, gentle enzymatic lysis protocols designed to preserve the DNA backbone fail to lyse Gram-positive bacteria, resulting in bias and loss of taxonomic representation within the community [107]. Conversely, adopting bead-beating methods to ensure comprehensive and unbiased lysis tends to produce fragmented DNA extracts, which limits the advantages of LRS [103].

Finally, low-biomass samples present an additional challenge. For samples characterized by low microbial abundance, the yield from conventional extraction methods may not meet the input requirements of LRS platforms (Table 3) [25, 108]. As demonstrated by Serghiou et al. in skin microbiomes and Child et al. in environmental soils, standard commercial kits often fail to balance yield and integrity in these niches, recovering only small amounts of fragmented DNA [109, 110]. Determining how to secure adequate extraction recovery rates from low initial biomass while simultaneously preserving DNA length and purity remains a technical issue in the field.

### Inherent limitations of data throughput and base-calling accuracy

Beyond sample preparation requirements, the limitations of LRS regarding data yield and sequencing depth also restrict its application. Compared to the terabase-scale single run throughput characteristic of SRS platforms, current LRS platforms exhibit a disadvantage in data output per unit cost. For instance, the maximum throughput per flow cell for PacBio and ONT platforms is ∼100–200 Gb, whereas Illumina systems can exceed 8 Tb (Table 3). This throughput limitation causes insufficient resolution for complex samples, which leads to subsequent analytical challenges.

First, this throughput deficit increases the dominance of highly abundant species over low-abundance taxa in the microbial communities. In complex habitats, most sequencing reads originate from dominant microbes. Consequently, due to insufficient overall depth, the probability of capturing species with relative abundances below 1% is substantially reduced [111]. Second, insufficient depth limits the assembly of uncultured microorganisms and leads to information loss. For uncultured taxa lacking reference genomes, the inability to achieve the target coverage requisite for *de novo* assembly makes it difficult to generate high-quality closed genomes or extensive contiguous sequences. Consequently, a massive proportion of the sequencing data remains fragmented, exhibiting low completeness scores upon evaluation with algorithms such as CheckM2, and is ultimately discarded as invalid data during the binning phase [112].

Furthermore, the errors associated with LRS in homopolymer regions also lead to downstream analytical challenges. Constrained by resolution limits of nanopore electrical signals or the kinetic characteristics of single-molecule polymerases, LRS are prone to insertion or deletion errors across consecutive identical bases [102]. Notably, these specific inaccuracies account for over 35% of the total sequencing error profile [113]. In metagenomic samples characterized by low sequencing depth and lacking supplemental error correction from SRS data, these non-random errors may induce frameshift mutations within protein-coding regions [114, 115]. Such sequence alterations affect accurate prediction of open reading frames and subsequent functional gene annotation.

### Algorithmic burdens in LRS

The convergence of algorithmic architectures and intrinsic sequencing error rates leads to substantial computational demands during the genomic assembly phase, particularly for metagenomes. Although OLC has a higher tolerance for sequencing inaccuracies [116], establishing topological relationships between long reads requires pairwise all-vs.-all alignments. This alignment mechanism increases computational overhead; for instance, during the assembly of the HG002 human genome, the alignment stage accounted for ∼79% of the total execution time [117, 118].

Furthermore, when processing metagenomic samples, contemporary assembly tools generate structural defects despite the allocation of massive computational resources. Evaluations indicate that prominent long-read metagenomic assemblers, including HiCanu, metaFlye, hifiasm-meta, and metaMDBG [119–122], generate an average of over 40 errors per 100 megabases of contiguous sequences. These inaccuracies transcend simple base-level substitutions and manifest as complex structural anomalies, such as chimeras and prematurely circularized sequences [123].

## Current sequencing choices and future perspectives

While recent reviews, such as the work by Agustinho et al., focus primarily on computational workflows and software tools used for long-read data analysis, our review centers on practical applications across biological scales. In this applied context, the optimal choice among SRS, LRS, and hybrid sequencing is now best defined by the biological question, sample complexity, and accuracy requirement. SRS remains widely used in taxonomic profiling, abundance estimation, and large cohort studies. For isolated bacterial genomes, recent PacBio HiFi and ONT R10/R10.4.1 workflows have shown that complete or near-complete chromosomes and plasmids can often be reconstructed without mandatory short-read polishing, provided that coverage, read length, and quality-control metrics are adequate [102, 124–126]. This does not make hybrid sequencing obsolete. Instead, hybrid approaches remain particularly useful when the study requires maximal consensus accuracy, conservative SNP or indel interpretation, recovery of difficult plasmids, validation of problematic low-coverage, and homopolymer-rich regions [106, 125–127]. In metagenomics, LRS-only approaches are increasingly powerful for cMAG recovery and structural resolution, but uneven species abundance, rare community members, strain mixtures, and assembly artifacts still make short-read supplementation or other orthogonal data valuable in selected settings [54, 121–123, 128–130].

What is changing most rapidly now is the accuracy. PacBio HiFi sequencing showed that long reads can reach a level of accuracy suitable for both assembly and variant analysis [102]. Subsequent microbial studies showed that complete or near-complete genomes can often be recovered from isolates and microbiomes without mandatory short-read correction [55, 121, 126]. On the ONT side, R10.4.1 chemistry and improved basecalling have narrowed the historical gap in bacterial genome reconstruction, especially at sufficient coverage [125]. The field is therefore moving toward Q30-class long reads, corresponding to ∼1 error per 1,000 bases, as a realistic standard in optimized workflows. Based on this, Q40-grade reads, corresponding to ∼1 error per 10,000 bases, are also becoming plausible [106]. On the other hand, ultra-long reads matter not simply because they are longer, but because they can span the regions that still create ambiguity in microbial assembly [131, 132]. Once such regions are bridged by single molecules, some assembly problems no longer need to be solved indirectly through graph inference. Even so, this will depend on more than sequencing chemistry. It will also require reliable extraction of HMW DNA from difficult samples and further reduction of systematic error.

At the same time, LRS is now limited as much by interpretation as by data generation. This is especially clear in metagenomics, where assembly still has to accommodate extreme abundance variation, microdiversity, shared repeats, and very large datasets. Recent assemblers such as metaMDBG, nanoMDBG, and myloasm show that algorithmic redesign can still improve memory efficiency, runtime, and recovery of cMAGs, plasmids, and viruses [62, 122, 133]. Binning is evolving in the same way. LorBin and recent cross-platform benchmarks indicate that long-read-aware and hybrid-aware strategies can substantially improve the recovery of high-quality genomes from communities [134, 135]. However, continuity does not guarantee correctness. Long-read metagenome assemblies can still contain chimeras, false duplications, haplotyping artifacts, and premature circularization [123]. This point matters because future tools will need to support automated validation, and better distinguish true biological microbial diversity from assembly artifact. Machine-learning and deep-learning methods are likely to contribute to this process. For instance, recent studies show that deep learning underlies modern nanopore basecalling, affects read accuracy and computational cost, and can further support microbial viability inference from raw nanopore signals [136–138]. Similar approaches are likely to influence methylation calling, assembly validation, binning, and cross-omic integration. If LRS is to become routine rather than specialist, these analytical advances will also need to be matched by lower cost, more standardized workflows, and greater automation [139].

Recent advances in nanopore protein sequencing have demonstrated its feasibility for single-molecule proteomics, including repeated reading of single polypeptides, single-amino acid sensitivity, and peptide discrimination for protein identification [140, 141]. This platform offers potential advantages over conventional mass spectrometry, as it does not require enzymatic digestion, enabling the direct characterization of proteoforms and post–translational modifications at single-molecule resolution [140, 142]. Although direct nanopore proteomics has not yet been applied to isolated bacterial strains or complex metagenomic samples, its prospective utility for microbiomics is substantial. In community settings, reading complete polypeptides could resolve the pervasive challenge of peptide sharing, where identical short sequences cannot be uniquely assigned among closely related strains.

**Figure 4:**
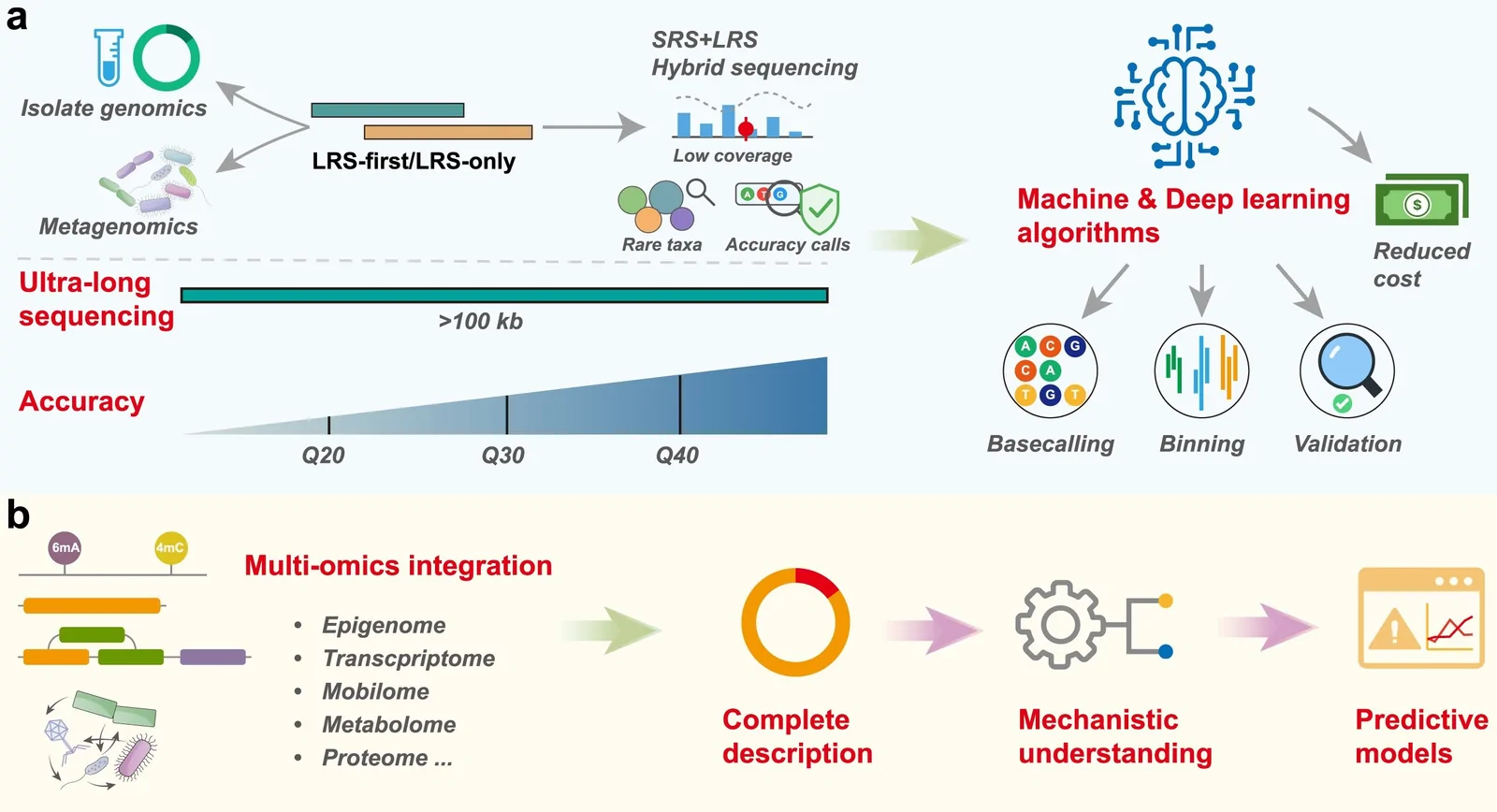
Future perspectives of long-read microbial genomics. (a) Technological and algorithmic evolution. High-accuracy LRS and emerging ultra-long sequencing approaches (>100 kb) are expanding comprehensive resolution across isolate genomics and metagenomics, with hybrid sequencing retained for depth-limited or accuracy-critical applications. Concurrently, continuous advancements in sequencing platforms are systematically driving raw accuracy toward Q30 and Q40 standards. Machine learning and deep learning methods are improving basecalling accuracy, genome binning, assembly validation, and computational efficiency. (b) Toward multi-omics integration. LRS empowers applications across the epigenome, transcriptome, mobilome, proteome, and metabolome. This integration supports more complete descriptions of genomic architectures, a mechanistic understanding of ecological interplay, and predictive models for microbial community dynamics.

The broader significance of LRS may become clearest in multi-omics studies (Fig. 4). Its main strength is that it preserves molecular context, allowing genome structure to be linked with DNA methylation, transcription, and, indirectly, protein and metabolite profiles. In bacteria, long-read transcriptomics has already shown that transcript organization is more complex than suggested by fragment-based RNA sequencing, revealing extended operons and substantial transcriptional heterogeneity [85]. More recently, nanopore direct RNA sequencing has begun to support joint analysis of the bacterial transcriptome and epitranscriptome within the same experiment [93]. LRS is also becoming increasingly important for epigenomic profiling and transcript-level analysis more broadly [143, 144]. The real opportunity for microbiology lies in combining these measurements with genome-resolved metagenomics, metatranscriptomics, metaproteomics, and metabolomics. Standardized multi-omics studies have already shown that linked analysis of genomes, functions, and metabolites can reveal ecological structure that is invisible to any single modality [145–147]. In this setting, LRS could serve as the scaffold that anchors different measurements to the same genome or strain. That would make it easier to ask which lineages are active, which elements are mobilized, which methylation states accompany regulatory change, and how these properties shift over time or under perturbation. In the longer term, such a framework may support models that monitor strain turnover, improve prediction of engraftment or instability, and provide earlier warning of resistance emergence or community collapse. For this reason, the future value of LRS is unlikely to be defined by assembly alone. Its deeper contribution may be to help microbial genomics move from increasingly complete description toward a more mechanistic and predictive framework.

## Conclusion

This review provides a systematic evaluation of the application potential and challenges of LRS within microbial genomics. LRS overcomes the inherent limitations of sequence fragmentation and amplification bias. This technological advancement not only facilitates the reconstruction of complete isolate genomes and precise localization of genetic elements, but also enhances metagenomics by enabling the recovery of cMAGs, strain-resolved analysis, and the linkage of MGEs to their specific replicons. Furthermore, LRS extends microbial analysis into multi-omics dimensions by directly detecting epigenetic modifications and capturing full-length transcripts. However, LRS remains constrained by challenges, primarily the requirements for HMW DNA extraction, sequencing depth deficits, and substantial computational burdens. Currently, while high-accuracy LRS is increasingly preferred for isolate genomics and metagenomics, hybrid sequencing is retained for depth-limited, difficult-replicon, rare-taxon, or accuracy-critical applications. Looking forward, ongoing advancements in sequencing accuracy, efficient bioinformatic tools, and multi-omics integration will further propel LRS to decode global microbial diversity and map the architecture of microbial ecosystems.

## List of abbreviations

ARG: antibiotic resistance gene; BGC: biosynthetic gene cluster; cMAG: circular metagenome-assembled genome; DNBSEQ: DNA nanoball sequencing; FMT: fecal microbiota transplantation; GC: guanine–cytosine; HGT: horizontal gene transfer; HMW DNA: high-molecular-weight DNA; IS: insertion sequence; LRS: long-read sequencing; MAG: metagenome-assembled genome; MGE: mobile genetic element; OLC: overlap layout consensus; ONT: Oxford Nanopore Technologies; R-M: restriction–modification; rRNA: ribosomal RNA; SNV: single nucleotide variant; SRS: short-read sequencing; SV: structural variant; TSS: transcription start site; TTS: transcription termination site.

## Supplementary Material

giag087_Authors_Response_To_Reviewer_Comments_Original_Submission

giag087_Authors_Response_To_Reviewer_Comments_Revision_1

giag087_GIGA-D-26-00124_Original_Submission

giag087_GIGA-D-26-00124_Revision_1

giag087_GIGA-D-26-00124_Revision_2

giag087_Reviewer_1_Report_Original_Submission Reviewer 1 -- 5/17/2026

giag087_Reviewer_1_Report_Revision_1 Reviewer 1 -- 7/1/2026

giag087_Reviewer_2_Report_Original_Submission Reviewer 2 -- 5/19/2026

giag087_Reviewer_2_Report_Revision_1 Reviewer 2 -- 6/30/2026

giag087_Reviewer_2_Report_Revision_2 Reviewer 2 -- 8/12/2026

giag087_Reviewer_3_Report_Revision_2 Reviewer 3 -- 8/12/2026

## Data Availability

Not applicable.
