# Supplementary material for: Promises and pitfalls of long-read sequencing for resolving microbial complexity
Source: Gigascience. 2026 Aug 17;15:giag087. doi: 10.1093/gigascience/giag087 (PMC13543314; doi:10.1093/gigascience/giag087)
Supplement: Supplementary file 1 [file giag087_authors_response_to_reviewer_comments_original_submission.pdf]

Author's Response To Reviewer Comments

Dear editor and Reviewers,

We thank all of you for taking the time to edit or review this manuscript. We appreciate all the professional comments and suggestions, which are very helpful in improving this manuscript. We have carefully considered these critical comments and thoughtful suggestions. The point-to-point responses to the reviewers' concerns are listed below. Besides, a marked-up version of the revised manuscript with the changes has also been submitted.

Regards,
Yuanqiang Zou, PhD

Responses to reviewers:

#Reviewer 1:

This review provides a comprehensive overview of the paradigm shift that long-read sequencing (LRS) has brought to microbial genomics and appropriately discusses its current technical limitations. A particular strength of the manuscript is the unusually rich set of case studies focused on pathogenic bacteria and antibiotic resistance genes, which the authors discuss from several complementary angles. This emphasis is, in my view, the most distinctive contribution of the review. Based on this strength, I would like to suggest the following improvements to further enhance the manuscript:
* Addition of a schematic figure that specifically illustrates how LRS has benefited pathogenic bacterial genomics would substantially help readers situate the technological gains in a translational context.
* The long-read metagenomics sections would benefit from additional content connecting LRS to pathogenic bacterial genomics. For example, the current status of real-time pathogen detection enabled by long-read metagenomics is a particularly impactful topic and would be a natural extension.
* If the authors retain the current emphasis on pathogenic bacteria throughout the manuscript, a corresponding revision of the title to more accurately reflect this scope would be warranted.

Response:
We sincerely thank the reviewer for the positive and encouraging evaluation of our work. We also greatly appreciate the constructive feedback and insightful concerns you have raised to further improve the manuscript. We have carefully considered each of your points and have made comprehensive revisions accordingly.
We are pleased that the pathogenic-bacteria and antibiotic-resistance case studies were viewed as a distinctive strength of the review. We agree that the translational implications of LRS should be made clearer, especially in the metagenomics section. In response, we have added a new subsection entitled "Pathogenic bacterial genomics and real-time detection," focusing on culture-independent long-read metagenomics from clinical specimens. This new section discusses real-time nanopore pathogen detection, AMR-gene screening, and the possibility of partial genome recovery when pathogen load and sequencing depth are sufficient. We cite primary studies demonstrating rapid nanopore metagenomic diagnosis of lower respiratory infection, real-time pathogen detection from infected body fluids, and a recent unified host-depletion nanopore workflow for detection of bacteria, fungi and viruses from respiratory samples. The added text can be found in Lines 432-457 as "Long-read metagenomics provides a culture-independent route from clinical specimens to pathogen information, making it an important translational extension of genome-resolved microbial genomics. Its distinctive advantage in this setting is the combination of long molecular reads and real-time nanopore analysis. Reads can be classified during sequencing, allowing pathogen detection and resistance-gene screening to begin before the sequencing run is complete. In lower respiratory infection, Charalampous et al. developed a host-depleted nanopore metagenomic workflow that produced results within 6 h from sample to result, detected bacterial pathogens with 96.6% sensitivity compared with culture, and accurately detected antibiotic-resistance genes[84]. In infected body fluids, Gu et al. showed that nanopore mNGS coupled with real-time computational analysis enabled pathogen identification with a median 50 min of sequencing and a 6 h sample-to-answer time[85]. More recently,

Alcolea-Medina et al. developed a unified host-depletion and nanopore workflow for respiratory samples that detected RNA and DNA viruses, bacteria, atypical bacteria and fungi, generated first automated reports after 30 min of sequencing within a 7 h workflow, and achieved prospective bacterial detection sensitivity and specificity of 90% and 100% after 2 h of sequencing[86]. This study also showed that genome recovery was feasible for a subset of reported organisms after longer sequencing, supporting the potential of clinical long-read metagenomics to move beyond detection toward strain typing and resistance or virulence analysis when pathogen load and read depth are sufficient[86]. These studies indicate that LRS-based metagenomics is most relevant when culture is slow, antibiotic exposure may reduce culture sensitivity, or rapid resistance information is needed. Clinical implementation nevertheless remains constrained by host-background DNA, contamination control, pathogen-load thresholds, database interpretation, genotype-phenotype concordance for AMR, and the need for prospective validation."
Furthermore, we carefully considered the suggestion to add a dedicated schematic figure and revise the title. However, because the review still covers microbial genomics broadly, including isolate genome closure, MAG/cMAG recovery, mobile genetic elements, strain variation, epigenomics, transcriptomics, and metagenomic ecology across both pathogenic and non-pathogenic contexts, we have decided not to add a pathogen-specific figure or narrow the title. We understand that the pathogenic examples in Section 2 may created a misleading impression. To correct this imbalance and better reflect our original intent, we have revised as follows. We removed the pathogenic cases of Chryseobacterium indologenes and Klebsiella pneumoniae, and added probiotic examples to explicitly demonstrate the broad microbial relevance. The added text can be found in Lines 146-150 as "Beyond genome completeness, LRS also enhanced functional recovery. For Lactiplantibacillus pentosus LP309, LRS resolved its complete chromosome and eight plasmids, enabling the prediction of over 150 probiotic functional genes involved in acid resistance and vitamin synthesis[44].", Lines 188-190 "For instance, McLeod et al. resolved the extensive plasmid complement of Lactiplantibacillus plantarum MF1298, yielding 14 complete plasmid sequences, one of which carried functional genes associated with vitamin B12 transformation[54].", and Lines 250-253 "Beyond resistance spread, complete genome resolution of the probiotic Lactobacillus crispatus precisely anchored Tn7088, a novel integrative mobile element encoding a class I bacteriocin biosynthetic gene cluster that drives antimicrobial competition and probiotic fitness[62]."

References:
[44]Marin-Gordillo A et al. Hybrid genome 942 assembly and comprehensive genomic analysis of Lactiplantibacillus pentosus 943 LP309 reveal its probiotic and technological potential. BMC Genomics. 2026; 944 .doi: 10.1186/s12864-026-12678-y.
[54]McLeod A et al. Large Plasmid Complement Resolved: Complete Genome Sequencing of Lactobacillus plantarum MF1298, a Candidate Probiotic Strain Associated with Unfavorable Effect. Microorganisms. 2019; doi: 10.3390/microorganisms7080262.
[62] Colombini L et al. The mobilome of Lactobacillus crispatus M247 includes two novel genetic elements: Tn7088 coding for a putative bacteriocin and the siphovirus prophage PhiM247. Microb Genom. 2023; doi: 10.1099/mgen.0.001150.
[84] Charalampous et al. Nanopore metagenomics enables rapid clinical diagnosis of bacterial lower respiratory infection. Nature Biotechnology. 2019. doi: 10.1038/s41587-019-0156-5.
[85] Gu et al. Rapid pathogen detection by metagenomic next-generation sequencing of infected body fluids. Nature Medicine. 2021. doi: 10.1038/s41591-020-1105-z.
[86] Alcolea-Medina et al. Unified metagenomic method for rapid detection of microorganisms in clinical samples. Communications Medicine. 2024. doi: 10.1038/s43856-024-00554-3.

Major comments
1. Section 2.3 — Claim that long reads outperform short reads in SNP detection (Lines 205-208, p.10)
The current text states that ONT long reads outperform short reads for SNP calling. Given that, on a per-base basis, short reads still have a substantially lower error rate than long reads, this conclusion may strike many readers as counter-intuitive. In all likelihood, the long-read advantage is restricted to specific genomic contexts (for example, repetitive regions and loci embedded in structural variants or mobile genetic elements) where short reads cannot be uniquely mapped. I recommend that the authors explicitly clarify the underlying reason why long reads outperform short reads at SNP calling in these specific contexts. This framing will help prevent readers from overestimating the general per-base SNP accuracy of LRS platforms.
Response:

We thank the reviewer for this important and valuable guidance. We agree that the raw per-base consensus accuracy of short reads retains a lower per-base error rate and remains highly reliable for genomic study. Our original phrasing inadvertently overstated the generalized accuracy of long reads which overshadowed the contribution of SRS. To address this, we have explicitly mentioned at Line 196-204 "Although one of the clearest advantages of LRS lies in resolving large inversions, duplications and other structural variants, they also improve the detection and interpretation of smaller-scale variants such as single nucleotide variants (SNVs), when these variants occur within complex genomic architecture. Recent ONT-based benchmarking showed that Illumina- and k-mer-based SNP typing failed to detect 9 to 68 SNPs per 1,000 sites in repetitive reference regions. Conversely, by spanning these complex regions on one long single molecule, ONT long reads improved SNP calls across the previously inaccessible genomic regions, where SRS alignment is at disadvantage." to clarify the advantage of LRS in SNV calling is localized to repetitive regions, duplications, and structurally variable loci and these were the result of LRS ability to produce a single molecule with enough length (10 to 100kb) that span across genomic architecture. In these specific contexts, short reads struggle with multi-mapping ambiguities due to fragmented reads were mentioned.
Accordingly, we revised words and sentence selection in Section 2 to prevent overstating and misinterpretation. Exaggerated claim in Line 199 "perfect full SNP calls" was replaced with a more nuanced language emphasizing LRS contribution in improving variant resolution in intricate regions.

2. Figure 2 / Section 3 — Why does cMAG construction expand phylogenetic diversity? (Lines 288-289, p.14)
The current sentence — "LRS facilitates the direct reconstruction of cMAGs, thereby significantly expanding the known phylogenetic diversity" — is asserted without an explicit rationale. If the sole goal were to expand phylogenetic diversity, conventional 16S rRNA gene surveys would, in principle, already suffice. I recommend that the authors clarify (and cite supporting primary literature, both in the main text and in the Figure 2 caption) the specific reasons why cMAG construction is required to expand phylogenetic diversity beyond what marker-gene approaches can achieve.
Response:
Thanks very much for this helpful comment. We agree that the original wording did not clearly explain why cMAG construction expands phylogenetic diversity beyond what marker-gene surveys can provide. Although 16S rRNA gene surveys can detect divergent lineages, they do not provide complete genome representatives, phylogenomic placement based on conserved single-copy genes, or linkage between taxonomic identity and functional elements such as metabolic pathways, BGCs, CRISPR-Cas systems and MGEs. In contrast, cMAGs enable uncultivated lineages to be represented as genomes and interpreted in both evolutionary and functional contexts. We have added supporting citations to primary literature showing that genome-resolved metagenomics expands the microbial genome tree and that long-read terrestrial metagenomics recovered >15,000 previously undescribed genomes together with complete rRNA operons, BGCs, and CRISPR-Cas systems. We have therefore revised both Section 3.1 as "More recently, deep long-read surveys across terrestrial habitats recovered genomes representing more than 15,000 previously undescribed genomes and expanded the known phylogenetic breadth of prokaryotic diversity. Meanwhile, LRS converted divergent marker-gene signals or fragmented metagenomic assemblies into high-quality genome representatives that can be placed in phylogenomic frameworks and linked to complete ribosomal RNA operons, BGCs, and CRISPR-Cas systems[69]. This indicates that long-read metagenomics contributes not only to improved assembly of known taxa and recovery of lineages that remain poorly represented in genome-centred reference frameworks, but also to help explore repetitive, structurally complex, and functionally critical elements that define microbial adaptability and evolution." in Lines 315-325 and revised Figure 2 caption as "LRS facilitates the reconstruction of cMAGs, expanding genome-resolved phylogenetic diversity by generating complete or near-complete genome representatives that can be placed in phylogenomic frameworks and linked to functional elements" in Lines 274-278.

References:
[69] Sereika M, Mussig AJ, Jiang C, et al. Genome-resolved long-read sequencing expands known microbial diversity across terrestrial habitats. Nat Microbiol. 2025; doi: 10.1038/s41564-025-02062-z.

3. Chromosome-MGE linkage in metagenomic contexts (Lines 292-294, p.14)
I agree with the statement that long reads enable the separation of chromosomal contigs and MGE contigs in single-isolate assemblies. However, in a true metagenomic context, identifying the chromosomal host of

an MGE remains difficult even with long reads alone. The current wording risks overstating the present capability. I suggest the authors add a sentence describing which complementary strategies can (at least partially) establish chromosome-MGE linkages in metagenomes with appropriate citation, such as proximity-ligation methods (Hi-C / 3C), methylation-based host assignment, and ultra-long reads that physically span the integration site between the MGE and its chromosomal flanking sequence.
Response:
Thanks the reviewer for this important clarification. We agree that the previous wording overstated host assignment as a general capability of long-read metagenomics. For integrated MGEs, including prophages, LRS can provide direct sequence evidence when reads or contigs span the element-chromosome junction. Long-read assemblies can also improve phage-host association because longer viral and bacterial contigs improve CRISPR-based matching and host taxonomic assignment. However, extrachromosomal plasmids remain more difficult to assign because they usually lack a fixed chromosomal junction, often have mosaic sequence composition, and may move across taxonomic boundaries. We therefore added complementary strategies, including Hi-C/3C proximity ligation and methylation-based binning, as orthogonal evidence for plasmid-host and broader MGE-host assignment in metagenomic datasets.
We have revised Section 3.3 to distinguish different types of MGE-host evidence as "LRS can strengthen host-MGE analysis in two distinct ways. For integrated MGEs, including prophages, reads or contigs that span the element-chromosome junction provide direct sequence evidence for the host genome[76]. In addition, longer viral and bacterial contigs can improve phage-host association by recovering more complete viral genomes, CRISPR arrays and host taxonomic signals. For example, viralFlye showed that long-read assemblies increased CRISPR-based virus-host predictions and improved host assignment precision relative to short-read assemblies[77]. Extrachromosomal plasmids are often harder to assign because they lack obligatory chromosomal junctions, have mosaic sequence composition and may circulate across multiple hosts. Therefore, plasmid-host and ARG-bearing MGE assignments in metagenomes often require orthogonal evidence, such as Hi-C/3C proximity ligation, methylation-aware binning, or ultra-long reads when they span integration or cointegration junctions[78]." in Lines 380-392.
We also have revise the Figure 2 caption as "Furthermore, LRS supports evidence-based host-MGE association by resolving integration junctions and improving phage-host inference, while complementary strategies provide evidence for assigning plasmids, phages and ARG-bearing elements to likely hosts" in Lines 281-284.

References:
[76] Yahara K, Suzuki M, Hirabayashi A, et al. Long-read metagenomics using PromethION uncovers oral bacteriophages and their interaction with host bacteria. Nat Commun. 2021; doi: 10.1038/s41467-020-20199-9.
[77] Antipov D, Raiko M, Lapidus A, Pevzner PA. viralFlye: assembling viruses and identifying their hosts from long-read metagenomics data. Genome Biology. 2022; doi: 10.1186/s13059-021-02566-x.
[76] Beaulaurier J, Zhu S, Deikus G, et al. Metagenomic binning and association of plasmids with bacterial host genomes using DNA methylation. Nat Biotechnol. 2018; doi: 10.1038/nbt.4037.

4. Section 3.2 — Mechanism of strain-resolution long-read metagenomics, and recent tooling (p.16)
The emphasis on strain resolution as a key strength of long-read metagenomics is well taken and highly valuable for the field. To strengthen this section further, please add a concise explanation of how strain resolution is actually achieved with long reads (e.g., preservation of haplotypic linkage on a single molecule, phasing of SNVs/SVs across long stretches, and traversal of structurally variable accessory loci). In addition, the recent release of myloasm (Shaw J, Marin MG, Li H. High-resolution metagenome assembly for modern long reads with myloasm. Nat Biotechnol. 2026; https://doi.org/10.1038/s41587-026-03053-z) makes strain-resolution long-read metagenomic assembly substantially more accessible in practice, and I encourage the authors to mention this development in this section.
Response:
Thanks very much for this constructive suggestion. We agree that Section 3.2 should explain not only that long-read metagenomics improves strain resolution, but also how this resolution is achieved. LRS preserve haplotypic linkage on individual DNA molecules, allowing single-nucleotide variants, indels, and SV breakpoints to be phased across long genomic intervals. They also traverse structurally variable accessory loci, including prophages, BGCs, CRISPR arrays, and insertion-sequence-flanked islands, that are often fragmented or collapsed in short-read assemblies. We also added the recent myloasm study to this section, noting that modern long-read assemblers are making strain-resolved metagenome assembly more

accessible by improving graph resolution in metagenomic samples.
We have revised the text as "Long-read metagenomics is also redefining strain resolution in microbial communities. particularly when multiple closely related lineages coexist within the same sample. Their differences are encoded not only by SNVs, but also by accessory gene cassettes, inversions, prophage carriage, tandem duplications and large insertions. Mechanistically, long reads improve strain resolution because individual molecules preserve haplotypic linkage across distant variants, allowing SNVs, small indels and SV breakpoints to be phased over long genomic intervals rather than inferred from unlinked short-read fragments. Long molecules can also traverse structurally variable accessory loci, including BGCs, prophages, CRISPR arrays and IS-flanked islands, that would otherwise fragment assembly graphs or collapse related strains into consensus representations. Recent algorithmic advances further improve practical access to this resolution: myloasm uses polymorphic k-mers and high-resolution string graphs for modern HiFi and ONT R10.4 long reads, improving the recovery of complete circular contigs and previously inaccessible within-species diversity in metagenomic datasets[72]" in Lines 327-341.

References:
[72] Shaw J, Marin MG, Li H. High-resolution metagenome assembly for modern long reads with myloasm. Nat Biotechnol. 2026; doi: 10.1038/s41587-026-03053-z.

5. Section 4.1 — HMW DNA, low-abundance modifications and sequencing depth (Lines 488-491, p.23)
Two related points require clarification here. First, please explain explicitly why HMW DNA extraction improves the accuracy of methylation calling. Second, the statement that ONT "lacks sufficient accuracy in detecting very low-abundance modifications" is essentially equivalent to saying that sequencing depth is insufficient — and this is not a limitation specific to methylation analysis. At current depths, low-abundance chromosomes and MGEs frequently fail to assemble at all. I therefore recommend either reframing this limitation more precisely or, ideally, if possible, providing a practical description for the sequencing depth required to recover reliable methylation profiles from representative samples such as the human gut microbiome.
Response:
Thank you for this insightful comment. We fully agree with your comments and have revised the text accordingly. First, regarding HMW DNA extraction: we have now explicitly explained that its role in improving methylation calling accuracy lies in enabling longer reads and more contiguous assemblies, which are essential for reliably assigning methylation motifs to specific genomic elements. Second, you correctly pointed out that the original wording was imprecise and that the core issue is sequencing depth, a limitation not unique to methylation analysis. We have therefore reframed this statement to focus explicitly on depth, and we have added a practical depth benchmark with a note that even deeper sequencing may be needed for rare taxa in complex samples such as the human gut microbiome. The revised paragraph is as follows: "However, several challenges remain. Accurate long-read metagenomic methylation analysis depends on the recovery of HMW DNA, which is required to generate long reads and contiguous assemblies that enable reliable assignment of methylation motifs to chromosomes, plasmids, and phages. In addition, the methylation characterization of low-abundance taxa remains limited by sequencing depth. Ding et al. found that at the 25× sequencing depth recommended by PacBio, only 16.24% of phage genomes met this requirement, and the number of detected methylation sites increased with deeper sequencing[96]. Furthermore, different types of methylation modifications exhibit distinct depth requirements. For instance, 40× depth is sufficient to profile most 6mA sites, whereas 4mC resolution requires at least 60× depth to achieve comparable sensitivity[97]."(lines 514-524)

References:
[96] Ding Y, Zhao L, Wang G, et al. PacBio sequencing of human fecal samples uncovers the DNA methylation landscape of 22 673 gut phages. Nucleic Acids Res. 2023; doi: 10.1093/nar/gkad977.
[97] Wilbanks EG, Dore H, Ashby MH, et al. Metagenomic methylation patterns resolve bacterial genomes of unusual size and structural complexity. ISME J. 2022; doi: 10.1038/s41396-022-01242-7.

6. Table 1 / Section 6 — Over-representation of CycloneSEQ studies and disclosure of competing interests
In Table 1, every entry from 2025-2026 uses a CycloneSEQ-WT02 + DNBSEQ-T5 hybrid strategy and is contributed by BGI-affiliated groups. Given that several of the authors of this review are affiliated with BGI Research, the present selection risks the appearance of a non-representative emphasis on a single platform that is commercially associated with the authors' institution. I recommend two adjustments: (i) explicitly

add a Competing Interests statement disclosing the authors' affiliation with BGI and the relationship to the CycloneSEQ platform; and (ii) balance the recent rows of Table 1 (and the corresponding narrative in Section 6) with comparable 2024-2026 studies using ONT or PacBio platforms. This will substantially strengthen the reader's confidence that platform recommendations in this review reflect the broader field rather than the authors' in-house technology.
Response:
Thanks very much for these important suggestions. We agree that the previous version of the table over-represented recent CycloneSEQ-WT02 + DNBSEQ-T5 hybrid studies, which could create the impression of platform imbalance given our institutional affiliation. We have therefore revised the table to better represent recent developments across ONT, PacBio, and CycloneSEQ/DNBSEQ platforms. Specifically, we added recent ONT and PacBio studies evaluating LRS or hybrid bacterial genome reconstruction, including Abdel-Glil et al (2025, Microb Genom), Xu et al (2025, Nat Commun), Adams et al (2025, Microbiol Resour Announc), Nagy et al (2026, Microb Genom). We also added a Competing Interests statement disclosing the authors' affiliation with BGI Research and the institutional relationship to BGI-associated sequencing platforms as "The CycloneSEQ was initially developed by BGI Research and is now being marketed as an advanced technology in MGI. All authors except Yuzheng Gu are employees of BGI Research. The authors declare that this affiliation did not influence the selection, interpretation, or presentation of the literature reviewed in this article." at Lines 797-800.
In Section 6, we revised the related text as "However, SRS alone often fail to resolve complete chromosomes, plasmids, long repeats, and complex structural variants. For isolated bacterial genomes, recent PacBio HiFi and ONT R10/R10.4.1 workflows have shown that complete or near-complete chromosomes and plasmids can often be reconstructed without mandatory short-read polishing, provided that coverage, read length, and quality-control metrics are adequate" at Lines 656-661.
Furthermore, we would like to respectfully point out that many studies in the original manuscript, including those by Zhao et al., Jiang et al., Canalda-Baltrons et al., Moss et al., Bickhart et al., Singleton et al., Sereika et al., Chin et al., Koren et al., Li et al., Fan et al., and Yan et al., were performed using ONT and PacBio platforms, and we presented their advantages in a balanced and objective manner. Thus, the manuscript has not exhibited a systematic bias toward the CycloneSEQ platform.

7. Section 6 / Abstract / Conclusion — Tone of the "hybrid remains the most practical strategy" recommendation should be revised
The manuscript's top-line recommendation — that "hybrid sequencing strategies remain the contemporary method" (Abstract line 47; Conclusion line 753) — no longer reflects the current state of the field. As the authors themselves note in Section 6, complete or near-complete genomes can often be recovered from isolates and microbiomes without mandatory short-read correction (Lines 667-669), and the field is moving toward Q30-class long reads as a realistic standard (Lines 671-672). In practice, for the most common use case — isolated genome sequencing — LRS-only workflows are already sufficient, and hybrid sequencing is only required in specific situations such as low-coverage regions, capture of rare community members in metagenomes, and applications where the highest possible consensus accuracy or conservative variant interpretation is critical (as the authors describe in Lines 657-662). I recommend that the authors revise the overall tone of Section 6, the Abstract, and the Conclusion to reflect this reality: namely, that LRS-only should now be presented as the default for isolate genomics and increasingly for metagenomics, with hybrid sequencing positioned as a targeted strategy for the specific conditions in which it remains clearly justified. Without this rebalancing, the current top-line statement risks understating the rapid accuracy gains of the past two years and inadvertently steering readers away from workflows that the same Section 6 acknowledges as already viable.
Response:
We thank the reviewer for this important comment. We agree that the original statement that "hybrid sequencing strategies remain the contemporary method" was too broad and could understate recent improvements in long-read accuracy. We have revised the Abstract , Section 6, Figure 4 caption and Conclusion to present sequencing strategy as application-dependent rather than as a single universal hierarchy as follows:
"Nevertheless, LRS is increasingly becoming the method of choice for isolate genomics and metagenomics" in the Abstract in Lines 45-46.
"In this applied context, the optimal choice among SRS, LRS, and hybrid sequencing is now best defined by the biological question, sample complexity, and accuracy requirement. SRS remains widely used in microbial genomics because it provides high-throughput, cost-effective depth, and high per-base accuracy,

particularly for taxonomic profiling, abundance estimation, and large cohort studies. However, SRS alone often fail to resolve complete chromosomes, plasmids, long repeats, and complex structural variants. For isolated bacterial genomes, recent PacBio HiFi and ONT R10/R10.4.1 workflows have shown that complete or near-complete chromosomes and plasmids can often be reconstructed without mandatory short-read polishing, provided that coverage, read length, and quality-control metrics are adequate. This does not make hybrid sequencing obsolete. Instead, hybrid approaches remain particularly useful when the study requires maximal consensus accuracy, conservative SNP or indel interpretation, recovery of difficult plasmids, validation of problematic low-coverage, and homopolymer-rich regions. In metagenomics, LRS-only approaches are increasingly powerful for cMAG recovery and structural resolution, but uneven species abundance, rare community members, strain mixtures and assembly artefacts still make short-read supplementation or other orthogonal data valuable in selected settings." in the Section 6 in Lines 651-669.
"High-accuracy LRS and emerging ultra-long sequencing approaches (>100 kb) are expanding comprehensive resolution across isolate genomics and metagenomics, with hybrid sequencing retained for depth-limited or accuracy-critical applications." in the Figure 4 caption in Lines 743-746.
"Currently, while high-accuracy LRS is increasingly preferred for isolate genomics and metagenomics, hybrid sequencing is retained for depth-limited, difficult-replicon, rare-taxon, or accuracy-critical applications." in the Conclusion in Lines 773-776.

8. Sections 4.2 and 4.3 — Unclear metagenomic relevance of full-length transcriptomics and single-molecule proteomics
Sections 4.2 (full-length transcriptomics) and 4.3 (single-molecule proteomics) are written essentially as general technology overviews of long-read transcriptome / proteome sequencing, without demonstrating how either technique is actually applied — or could plausibly be applied — in a microbial metagenomic context. Given that the central theme of this review is the resolution of microbial complexity in complex communities, it is unclear what additional value these two sections contribute in their current form. Specifically: (i) Section 4.2 discusses bacterial isolate transcriptomics in detail, but does not address the metatranscriptomic challenges that would actually be relevant to community-scale analysis (e.g., full-length RNA recovery from complex communities, rRNA depletion, assignment of transcripts back to specific lineages or strains); (ii) Section 4.3 contains essentially no microbial application examples, as refs 96-100 are technology-development papers, and the connection to "microbial complexity" is not made explicit. I therefore recommend either substantially expanding these sections to include concrete metagenomic / community-level use cases (even at the pilot or proof-of-concept stage), or, if such examples are scarce at present, relocating this material to an Outlook box or a subsection of Section 6 (Future perspectives) and tightening the body of the review to its actual scope.
Response:
We thank the reviewer for this constructive and insightful feedback. We agree that the previous version of these sections focused too heavily on technical principles rather than their direct relevance to resolving microbial complexity in community-level analyses. Accordingly, we have substantially revised Sections 4.2 and 4.3 as detailed below.
1.For Section 4.2: In the revised manuscript, we have added concrete application examples regarding community-scale analysis in lines 539-546 "For instance, ONT direct RNA sequencing extended the boundaries of 225 known E. coli operons and 89 defined S. aureus operons. Moreover, direct RNA sequencing enables the detection of native RNA modifications, such as m5C and m6A, providing new insights into the complexity of post-transcriptional regulation in bacteria. In a community context, integrating metatranscriptomics with nanopore long-read metagenomics enabled the reconstruction of trimethylamine metabolic networks and the identification of key microbial taxa[104]". Furthermore, we also discussed technical challenges of applying LRS in microbiomes, such as full-length RNA recovery from complex communities, rRNA depletion, the difficulty of assigning transcripts to specific strains."Nevertheless, applying LRS in the microbiome faces several technical challenges. Full-length RNA recovery is difficult due to rapid RNA degradation and inhibitory compounds (e.g., humic acids in soil)[105]. rRNA depletion is another essential step, as rRNA typically constitutes more than 90% of total bacterial RNA[106]. Assignment of transcripts to specific strains represents a third barrier, owing to high sequence similarity among closely related species and the lack of reference genomes[107]." in lines 546-552.
2.For Section 4.3: The reviewer correctly noted that this section contained no microbial application examples and that references 96–100 are technology-development papers. We agree with the reviewer's assessment, as direct nanopore protein sequencing has indeed not yet been validated on bacterial isolates

or complex metaproteomic samples. Therefore, we have moved the entire original Section 4.3 into Section 6. This paragraph highlights recent technological breakthroughs, potential advantages over mass spectrometry, and prospective applications in microbial communities. "Recent advances in nanopore protein sequencing have demonstrated its feasibility for single-molecule proteomics, including repeated reading of single polypeptides, single-amino acid sensitivity, and peptide discrimination for protein identification. This platform offers potential advantages over conventional mass spectrometry, as it does not require enzymatic digestion, enabling the direct characterization of proteoforms and post-translational modifications at single-molecule resolution[148]. Although direct nanopore proteomics has not yet been applied to isolated bacterial strains or complex metagenomic samples, its prospective utility for microbiomics is substantial. In community settings, reading complete polypeptides could resolve the pervasive challenge of peptide sharing, where identical short sequences cannot be uniquely assigned among closely related strains."in lines 706-716.

Reference:

[104] Simo C, Mamani-Huanca M, Hernandez-Hernandez O, et al. Application of nanopore long-read sequencing and metabolomics in an in vitro dynamic intestinal digestion model: A genome-centric metatranscriptomic approach to investigating microbial TMA and SCFA metabolism. J Pharm Biomed Anal. 2025; doi: 10.1016/j.jpba.2025.116896.

[105] Barber DG, Davies CA, Hartley IP, et al. Evaluation of commercial RNA extraction kits for long-read metatranscriptomics in soil. Microb Genom. 2024; doi: 10.1099/mgen.0.001298.

[106] Culviner PH, Guegler CK, Laub MT. A Simple, Cost-Effective, and Robust Method for rRNA Depletion in RNA-Sequencing Studies. mBio. 2020; doi: 10.1128/mBio.00010-20.

[107] Zhang W, Li XJ, Liu F, et al. Fungen: clustering and correcting long-read metatranscriptomic data for exploring eukaryotic microorganisms. Sci China Life Sci. 2026; doi: 10.1007/s11427-024-2853-x.

[148] Liu X, Dong Z, Timp G. Calling the amino acid sequence of a protein/peptide from the nanospectrum produced by a sub-nanometer diameter pore. Sci Rep. 2022; doi: 10.1038/s41598-022-22305-x.

Minor comments

1. p.4, Lines 82-83 — Additional citation suggested

The discussion of prophages confounding short-read assembly would benefit from an additional citation: Wirbel J, Hickey AS, Chang D, Enright NJ, Dvorak M, Chanin RB, Schmidtke DT, Bhatt AS. Long-read metagenomics reveals phage dynamics in the human gut microbiome. Nature. 2025; 1-9.

Response:

We thank the reviewer for the specific citation suggestion. As recommended, we have added the reference (Wirbel et al., Nature, 2026) in the relevant section (Lines 81), where we discuss that short reads frequently fail to establish linkage between mobile genetic elements (including prophages) and the chromosome.

2. Insufficient in-text citation of figures

Although the manuscript contains four figures, the figures are not adequately cited at the relevant points in the main text. Please add explicit in-text citations at every point where the corresponding figure panel supports the narrative, so that readers can map the discussion to the visual summary.

Response:

We thank the reviewer for this careful observation. We agree that clear in-text citations help readers connect the narrative with the visual summaries. Accordingly, we have now added explicit citations of the figures at all relevant points throughout the manuscript. Specifically, "line 131 – added citation of Figure 1; line 196 – added citation of Figure 1; line 303 – added citation of Figure 2; line 374 – added citation of Figure 2; line 498– added citation of Figure 3; line 531 – added citation of Figure 3." We believe these additions will improve the clarity and usability of the manuscript. Thank you again for this helpful suggestion.

Reviewer #2 - Major Revision

In this work, Rao and Gu et al. review the application of long read-sequencing to reconstruct microbial genomes from microbial isolate culture and metagenome-assembled genomes from microbiome samples. They provide an overview of the main long read-sequencing approaches and the analytical challenges they improve on, notably the resolution of extended repeats, genome rearrangements, and the detection of epigenetic modifications. While the authors cover the recent literature, I have the following comments:

Major comments:
1.The authors cite the work by Agustinho et al. (2024) Nature Methods. I found the overlap between the 2024 review and the present review quite large. Please specify what novel information the present review provides compared to previous reviews on long read-sequencing in the context of isolate genomes and/or metagenomes. It might be advisable to adjust the text accordingly for the reader to better appreciate what sets this review apart from other, very recent works.
Response:
Thank you for raising this important point. We have carefully read the 2024 review by Agustinho et al. in Nature Methods and fully acknowledge its value as a comprehensive methodological resource. In our revised manuscript, we have explicitly cited this work and clarified our distinct positioning in the Introduction (Lines 85-92): "For instance, Agustinho et al. provided a comprehensive synthesis of computational pipelines for long-read metagenomic assembly and taxonomic characterization, serving as an invaluable methodological reference. Nevertheless, that review was primarily methodological and centered on metagenomics. In contrast, our review systematically covers the reconstruction of gapless isolate genomes, the resolution of metagenomic populations with host–element linkages, and the characterization of native epigenomic and full-length transcriptomic landscapes." and at the beginning of the Section 6 (Lines 649-651): "While recent reviews, such as the work by Agustinho et al., focus primarily on computational workflows and software tools used for long-read data analysis, our review centers on practical applications across biological scales."
Although both reviews address LRS and metagenomics, we believe that our review makes distinct contributions in the following aspects:
Divergent focus: The review by Agustinho et al. primarily focuses on metagenomic analysis pipelines (assembly, binning, taxonomic profiling, variant detection, etc.) and compares different bioinformatics tools, such as MetaMaps, MEGAN-LR, MMseqs2, CDKAM, and BugSeq for taxonomic classification. In contrast, our review focuses more on what biological questions LRS can help to answer. For example, in the context of isolate genomes, LRS enables precise localization of mobile genetic elements and allows investigation of their transfer between hosts (Section 2.4).
Expanded metagenomics content: For the metagenomics section that both reviews cover, we offer a more comprehensive discussion than Agustinho et al. Specifically, Section 3.3 (Lines 371-430) systematically discusses the use of LRS in establishing host-element linkages, detailing how long molecular reads bridge assembly gaps to track mobile genetic elements, plasmids, phages, and antibiotic resistance genes. Furthermore, Section 3.4 (Lines 431-451) focuses on pathogen genomics and real-time detection, highlighting the clinical application of nanopore sequencing for rapid, culture-independent pathogen identification. These two parts are not detailed in their publication.
Broader scope: The review by Agustinho et al. is mainly centered on metagenomics. Our review covers three major application areas of LRS: isolate genomics, metagenomics, and multi-omics. Agustinho et al. barely discuss isolate genome assembly, whereas we provide an in-depth discussion in Section 2 on the contributions of LRS to microbial isolates. For instance, LRS enables the construction of complete, gapless bacterial genomes, which in turn allows the prediction of more functional genes. Furthermore, regarding extended applications, although both reviews touch upon methylation detection, we further discuss in Section 4 the use of LRS in transcriptomics and in Section 6 the emerging field of nanopore protein sequencing, these topics not covered in Agustinho et al. These unique sections constitute a substantial proportion of our total manuscript.

2.I found the text to read repetitive frequently, such as about fragmented assemblies using short reads and the possibility to sequence free of prior amplification with long reads. The authors refer to improved assembly using long reads but only introduce in section 5.3 how assembly actually takes place; a different order would help reduce redundancy.
Response：
We sincerely appreciate the reviewer’s careful reading and constructive feedback. We agree that our initial manuscript, in an effort to emphasize the limitations of SRS and the advantages of LRS, inadvertently introduced some repetitiveness. Section 1 already provides a comprehensive account of SRS shortcomings (repetitive regions, mobile genetic elements, GC bias, etc.). In later sections, we have eliminated redundant elaboration while preserving the key message. Following the reviewer’s suggestion, we have moved the comparison of SRS and LRS assembly strategies (including the explanation of De Bruijn graphs for SRS and OLC for LRS) from the original Section 5.3 to Section 2, where this earlier introduction helps readers understand the fundamental assembly differences, thereby reducing later redundancy and

improving overall coherence. The specific modifications are as follows:
"SRS assembly typically employs De Bruijn graphs, which decompose short reads (typically 100–300 bp) into short k-mers. As a result, short reads frequently fail to resolve complex repetitive regions, leading to gaps, misassemblies, and ambiguous contig ordering. In contrast, LRS adopts an overlap layout consensus (OLC) framework, where long reads (ranging from 10 kb to 30 kb or more) can span repetitive elements, enabling single circularized chromosomes and full reconstruction of extrachromosomal elements." in lines 106-113.
"Although SRS platforms generate accurate reads that are well suited for identifying conserved genomic regions, these reads are too short to disentangle extrachromosomal sequences with repetitive regions." in lines 168-170.
"However, short-read metagenomic sequencing often yields fragmented assemblies, which detach functional genes from their chromosomal, plasmid, or phage contexts."in lines 262-264.
"Historically, SRS relied on bisulfite chemical conversion, which frequently caused DNA degradation and severe GC bias, thus failing to simultaneously detect DNA sequence and native modifications at single-molecule resolution." in lines 488-491.

3.The authors declare no competing interests. However, Rao and Zou are both affiliated to BGI Research, with BGI producing the CycloneSeq platform. I think that in the interest of transparency, this should be disclosed by the authors.
Response:
We thank the reviewer for raising this important point regarding transparency. We fully agree that any potential affiliation with a commercial platform developer should be explicitly disclosed. In light of this suggestion, we have added the following statement to the Competing interests section (lines 797-800):"The CycloneSEQ was initially developed by BGI Research and is now being marketed as an advanced technology in MGI. All authors except Yuzheng Gu are employees of BGI Research. The authors declare that this affiliation did not influence the selection, interpretation, or presentation of the literature reviewed in this article." We believe this addition appropriately ensures full transparency.

4.The authors repeatedly refer to "physical linkage". Please carefully review this and appropriate use of respective phrasing in the text. While long read-sequencing can produce circular chromosomes and circular plasmids, physical linkage of these genetic elements is only established through complementary approaches, e.g., proximity ligation approaches. Put differently, circular chromosomes and circular plasmids are not automatically put into their shared organismal context through long read-sequencing.
Response:
We thank the reviewer for this rigorous comment. We fully agree that in its strict sense, "physical linkage" implies direct molecular connection, which LRS cannot establish across different replicons. Our intended meaning throughout the manuscript was to describe the ability of LRS to resolve the localization of MGEs, such as determining whether an MGE is located on a chromosome or a plasmid, and its precise position within a replicon. We realize that our original wording "physical linkage" was imprecise and could mislead readers, and we appreciate the reviewer for pointing this out.
To address this concern, we have thoroughly revised the manuscript to replace ambiguous uses of "physical linkage" with more accurate terminology. Specifically, "In metagenomics, LRS enables high-resolution strain parsing, the recovery of circular metagenome-assembled genomes, and the precise localization of MGEs within host replicons."in Lines 38-40. In the Introduction, we revised the related text as"Furthermore, the ubiquity of MGEs, such as plasmids and prophages, complicates reconstruction because short reads frequently fail to establish their integration sites within the primary chromosome" in Lines 79-81.

5. In the interest of consistency, please include a table like Table 1 but for metagenome-assembled genomes. Importantly, the use of long read-sequencing for isolate genomes is largely an accepted standard by now, while the use for the recovery of metagenome-assembled genomes remains a challenge.
Response:
Good suggestion! We agree that the manuscript should provide a metagenome-focused counterpart to the isolate-genome table, particularly because LRS-based isolate genome closure is now comparatively mature whereas MAG/cMAG recovery from metagenomes remains technically more challenging. We have added a new table as Table 2 summarizing representative long-read and hybrid studies for MAG/cMAG recovery, including the sequencing strategy, sample type, and main genome-recovery output.

Table 2 Representative LRS studies for MAG and cMAG recovery from metagenomes.
Year Study Platform and strategy Metagenomic dataset MAG/cMAG recovery
2019 Bertrand et al. Illumina + ONT nanopore; OPERA-MS hybrid assembly Mock communities and 28 human gut metagenomes Improved contiguity ~200-fold; recovered rare-species genomes and >80 closed plasmid/phage sequences.
2020 Moss et al. ONT MinION R9.4; Lathe workflow with short-read polishing 13 human stool metagenomes Recovered 20 circular bacterial genomes directly from microbiomes.
2021 Singleton et al. ONT PromethION + Illumina; long-read assembly with short-read support Activated sludge from 23 Danish WWTPs Recovered 1,083 HQ MAGs, including 57 closed circular genomes.
2022 Bickhart et al. PacBio HiFi + Hi-C binning; MAGPhase haplotype phasing Sheep faecal metagenome Recovered 428 MAGs, including 44 single-contig circular MAGs and 220 lineage-resolved MAGs.
2022 Feng et al. PacBio HiFi; hifiasm-meta assembler Seven empirical metagenomic datasets Reconstructed tens to hundreds of complete circular bacterial genomes per dataset.
2022 Kim et al. PacBio HiFi, including Sequel II data; metaFlye/HiCanu/hifiasm-meta plus cMAGfilter Five human faecal metagenomes Recovered 102 accurate cMAGs, including uncultured gut taxa.
2022 Gounot et al. Illumina HiSeq4K + ONT MinION/GridION/PromethION; OPERA-MS; Hi-C subset 109 Southeast Asian gut metagenomes Recovered 4,497 medium/HQ MAGs; 1,708 were absent from short-read-only analysis.
2024 Benoit et al. PacBio HiFi; metaMDBG assembler Human gut, anaerobic digester and sheep rumen datasets Increased HQ circular MAG recovery, with improved plasmid and virus assembly.
2025 Sereika et al. ONT P2/P24, SQK-LSK114/FLO-PRO114M; Flye/Medaka + mmlong2 workflow 154 soil and sediment samples Recovered 23,843 MAGs, dereplicated into 15,640 species-level MAGs.
2026 Benoit et al. ONT R10.4.1; nanoMDBG assembler Human gut, Zymo Fecal Reference and soil metagenomes Recovered more near-complete MAGs than ONT comparators; showed ONT can approach HiFi MAG recovery at matched depth.
2026 Shaw et al. PacBio HiFi and ONT R10.4; myloasm assembler Gut, oral and other metagenomic datasets Improved strain-resolved assembly and recovery of complete circular contigs.

Minor comments:
1.Please use nuanced language. Long read-sequencing has not "shattered", and definitely not "thoroughly", anything just yet, albeit it is a promising technology. Moreover, it remains to be seen whether there has indeed been an "era" of epitranscriptomics inaugurated. While I agree that the ease of accessing epigenetic modifications has been simplified with some long read-sequencing approaches (by ONT and PacBio), this has yet to be applied on a larger scale. Related to this, please check for the repeated use of "bottleneck". And why is it surprising that long read-sequencing "enable the recovery of thousands of complete ribosomal RNA operons …", given that this is exactly where long read-sequencing can show its strengths?
Response:
We sincerely thank you for pointing this out. We completely agree that our previous language was overly enthusiastic and lacked the necessary scientific nuance. And we have carefully revised the manuscript to adopt a more objective tone. Specifically, we have removed absolute terms such as "thoroughly shattered" and "inaugurated the era." We have also moved the entire discussion of direct nanopore protein sequencing to Section 6 (Future perspectives) to clearly indicate that while it is an emerging emerging approach rather than a currently established tool in microbiological contexts. Furthermore, we conducted a thorough review of the manuscript and removed the repetitive use of the word "bottleneck," replacing it with more precise terms like "limitations" and "constraints." Regarding the recovery of rRNA operons, we agree that this is an expected strength of long-read sequencing rather than a surprising finding; we have adjusted the phrasing in the respective sections to reflect this objectively.

2. The authors refer to "complex" when referring to microbiomes. I assume this is to differentiate microbiomes from isolate cultures, but it is extraneous if it is clear whether isolate genomes or metagenome-assembled genomes are targeted. More generally, what constitutes a "complex" microbiome and what constitutes a "simple" microbiome? I would advise against the use of such terms whenever possible.
Response:
Thanks very much for this helpful comment. We agree that the terms “complex” and “simple” are not operationally defined in our manuscript and may imply an artificial binary distinction among microbiomes. We have therefore revised the text to avoid using “complex microbiome/community” as a generic

descriptor. Where the intended contrast was between isolate genomics and metagenomics, we now use explicit terms such as “isolate genomes,” “microbial communities,” “metagenomic samples,” or “metagenome-assembled genomes.” For examples, we have replaced “Complex-community metagenomics is therefore moving beyond taxonomic inventories...” into “Long-read metagenomics is therefore moving beyond taxonomic inventories...” in Lines 267 and “Transformative applications of long-read metagenomics in complex microbial ecosystems” into “Applications of long-read metagenomics in microbial ecosystems” in Line 272.

3. Please pay attention to the introduction of abbreviations before their use, e.g., "TSS", "TTS", "SNVs", or "SV". Notably after possibly adjusting the text order as pointed out in the major comments. Moreover, if an abbreviation is only introduced but never reused, please consider not writing it in the first place, e.g., "WGS" which is introduced in line 247 but not used thereafter.
Response:
We thank the reviewer for this careful comment. We have thoroughly reviewed the manuscript to ensure that all abbreviations are defined at their first occurrence, and that abbreviations used only once are removed. Accordingly, we have relocated the full definitions of SVs (Lines 118), SNVs (Lines 199), cMAGs (Lines 275), BGCs (Lines 279), TSS (Lines 478), and TTS (Lines 479) to their respective first appearances in the text. In addition, we have removed abbreviations that appeared only once, such as “WGS”.

4. What do the authors mean by "haplotypic parsing"? Did they mean "haplotype phasing"? If so, please adjust throughout.
Response:
We thank the reviewer for this terminological clarification. What we intended to convey is the co-occurrence of multiple genetic variants (such as SNVs and SVs) along the same DNA molecule. We realize that the term "parsing" was improperly used to describe this. We have gladly accepted your suggestion and revised the manuscript to replace "haplotypic parsing" with "haplotype phasing" in the Figure 2 legend (lines 280).

5.Please pay attention to the correct spelling throughout, notably including also the figures. "CRISPR" is misspelled in Figure 2.
Response:
Thanks for pointing this out. We have corrected it. We also have thoroughly checked our manuscript to avoid similar mistakes.

6. What is the purpose of reference 144? I understand that the authors want to disclose the use of Gemini for language redaction, but the included link is a generic link to Gemini and not to the prompts that were used. On that note, from the reading it was readily apparent that a large language model was used as the language was often times exaggerated, see also my earlier comment on the advice to use more nuanced language.
Response:
We thank the reviewer for raising this point. Regarding the citation format (Reference 144), its inclusion is strictly to comply with the GigaScience "Instructions to Authors." The journal explicitly mandates the citation of generative AI tools and provides a specific template for authors to follow (requiring the version, model, date, user, and URL). Regarding the use of exaggerated language, we completely agree with your assessment. We recognize that the AI-assisted language redaction introduced some exaggerated phrasing. Taking this and your earlier advice, we have conducted a comprehensive manual review. For example, we have replaced the phrase “structural bottlenecks” with “these challenges” (line 87) and changed “transformative force” to “an important tool” (Line 87).

6.There appears to be an issue with Table 1. The description of the right-most column seems to overlap for the first line.
Response:
Thank you for noting this formatting issue. We have revised the layout of Table 1 by adjusting the column width and row height of the right-most column. The table has also been rechecked in the final version to ensure that the first-line description no longer overlaps and that all text is clearly readable.

7.Regarding proteomic sequencing, I would ask the authors to nuance this a bit. This approach is not yet

readily available and is likely to take several more years until it might be applied outside of specialized labs or as proof-of-principle. It seems that mass spectrometry based proteomics will remain the norm for the near future. To me, the text currently reads as if proteomic sequencing had entered the "mainstream", which is not the case.
Response:
We thank the reviewer for this important clarification. We fully agree that direct nanopore protein sequencing is still at an early stage and is not a widely available technology. Mass spectrometry remain the mainstream approach for protein detection. We regret that our original wording may have unintentionally suggested otherwise. In addition, we have moved the this content from the original Section 4.3 to Section 6 (Future perspectives). This relocation clearly signals that the technology is not yet a mature tool but rather a promising direction for the longer term. "Recent advances in nanopore protein sequencing have demonstrated its feasibility for single-molecule proteomics, including repeated reading of single polypeptides, single-amino acid sensitivity, and peptide discrimination for protein identification. This platform offers potential advantages over conventional mass spectrometry, as it does not require enzymatic digestion, enabling the direct characterization of proteoforms and post-translational modifications at single-molecule resolution. Although direct nanopore proteomics has not yet been applied to isolated bacterial strains or complex metagenomic samples, its prospective utility for microbiomics is substantial. In community settings, reading complete polypeptides could resolve the pervasive challenge of peptide sharing, where identical short sequences cannot be uniquely assigned among closely related strains."in lines 706-716.

em Editorial Manager®

Cookie Preference Center

We use cookies which are necessary to make our site work. We may also use additional cookies to analyze, improve and personalize our content and your digital experience. For more information, see our Cookie Policy.

You may choose not to allow some types of cookies. However, blocking some types may impact your experience of our site and the services we are able to offer. See the different category headings below to find out more or change your settings.

Allow all

Manage Consent Preferences

Strictly Necessary Cookies

Always active

These cookies are necessary for the website to function and cannot be switched off in our systems. They are usually only set in response to actions made by you which amount to a request for services, such as setting your privacy preferences, logging in or filling in forms. You can set your browser to block or alert you about these cookies, but some parts of the site will not then work.

Functional Cookies

These cookies enable the website to provide enhanced functionality and personalisation. They may be set by us or by third party providers whose services we have added to our pages. If you do not allow these cookies then some or all of these services may not function properly.

Performance Cookies

These cookies allow us to count visits and traffic sources so we can measure and improve the performance of our site. They help us to know which pages are the most and least popular and see how visitors move around the site.

Targeting Cookies

These cookies may be set through our site by our advertising partners. They may be used by those companies to build a profile of your interests and show you relevant adverts on other sites. If you do not allow these cookies, you will experience less targeted advertising.

Cookie List

Clear

Apply Cancel

Consent Leg.Interest

Confirm my choices

Powe
