# Supplementary material for: Promises and pitfalls of long-read sequencing for resolving microbial complexity
Source: Gigascience. 2026 Aug 17;15:giag087. doi: 10.1093/gigascience/giag087 (PMC13543314; doi:10.1093/gigascience/giag087)
Supplement: Supplementary file 2 [file giag087_authors_response_to_reviewer_comments_revision_1.pdf]

Author's Response To Reviewer Comments

Reviewer reports:

Reviewer #1: The authors provided appropriate responses to each comment and improved the manuscript, so I think it is possible to publish this review covering the current possibilities of long-read sequencing in microbial genomics.
Response:
We sincerely thank the reviewer for the positive evaluation of our revised manuscript and for recognizing the improvements made in response to the previous comments. We are also grateful for the reviewer's constructive suggestions, which helped us clarify the scope and strengthen the discussion of current applications of long-read sequencing in microbial genomics.

Reviewer #2: Summary: In this revised version of their review manuscript on long-read sequencing for resolving microbial complexity, Rao et al. implemented my comments to a limited extent.

Major comments:
1. Despite the authors' claim to have reduced redundancy, many parts still read repetitive. Notably, the point about reduced fragmentation due to longer reads being used in genomic assembly is coming up again and again. Often even as the main point, thus adding little new information. After all, this is what long read sequencing was developed for. This point also only differs marginally between isolate cultures and microbiomes. Meta-assemblers provide additional solutions for the difference in coverage but otherwise are highly similar to "non-meta" assemblers. Moreover, long reads hold potential beyond contigs, i.e., due to their length, relevant functional information covering neighboring genes could be recovered. As one early concrete example of repetitiveness, take L64+L65 and L72ff. Basically, L72ff are examples of "intricate genomic architecture". However, the reading flow is disrupted and comes back on a previous point. This is persistent throughout the manuscript.
Response:
We thank the reviewer for this important comment. We agree that the previous version still repeated the point that long reads reduce assembly fragmentation. In the revised manuscript, we therefore performed a more systematic revision to reduce redundancy and improve the flow of the review.
First, we reorganized the Introduction so that the genomic features that challenge SRS, including repetitive elements, MGEs, and GC-biased regions, are discussed together before LRS is introduced. This avoids returning to the same argument after the role of LRS has already been presented. "By integrating bridge amplification with sequencing-by-synthesis, SRS circumvented the need for microbial cultivation and supported landmark metagenomic initiatives like the Human Microbiome Project and METAgenomics of the Human Intestinal Tract. Despite these advances, SRS reads remain short relative to many microbial genomic features. This limitation is particularly evident in three contexts. First, repetitive elements, including ribosomal RNA (rRNA) operons and insertion sequences (ISs), can constitute up to 10% of some bacterial genomes, and their length may exceed the resolving capacity of SRS algorithms. Second, for mobile genetic elements (MGEs), such as plasmids and prophages, shared sequences and integration sites are difficult to assign from short reads alone. Third, extreme guanine–cytosine (GC) content may introduce coverage bias and lead to the underrepresentation or loss of affected regions. In response to these challenges, LRS platforms such as PacBio, Oxford Nanopore Technologies (ONT) and CycloneSEQ generate substantially longer reads and preserve broader genomic context. These features have expanded the analytical scope of microbial genomics. "(Lines 61-76)
Second, we revised repetitive descriptions that contrasted fragmented drafts with complete assemblies. For example, we removed the sentence "As a result, short reads frequently fail to resolve complex repetitive regions, leading to gaps, misassemblies, and ambiguous contig ordering". We also condensed the discussion of Koren et al. to focus on the main finding "Through an analysis of 2,267 complete bacterial and archaeal genomes, Koren et al. found that most microbial genomes could be closed using LRS" (Lines 121-122). In addition to these examples, we made revisions in other parts of the manuscript (Lines 122-126, 146-149, 214-216, 485-487, and 585-586) and made several smaller wording changes throughout the text to further reduce redundancy. Across these revisions, we shifted the emphasis from contig length and reduced fragmentation alone to the biological information enabled by LRS, including plasmid and

phage context, ARG localization, and strain variation.

2. I find that the points I raised have been addressed rather superficially. My comment on "haplotype parsing" was apparently only considered for Figure 2. However, uses in the text remain unchanged. Capitalization is inconsistent, notably in Figure 1 and Figure 4. As listed below several minor comments remain. The references seem not to have been checked thoroughly, e.g., L958 "48. . !!! INVALID CITATION !!! [45]."
Response:
Thanks very much for pointing this out. We have made the corrections and checked these issues throughout the manuscript. First, we reviewed "haplotype parsing" and related wording throughout the manuscript. We replaced the remaining uses of "strain parsing" or ambiguous "parsing" terminology with more precise terms, such as "strain resolved analysis". Second, we checked the capitalization in the figures and revised them for consistency. Third, we carefully rechecked the reference list and in text citations. The invalid citation noted by the reviewer has been corrected, and the reference formatting has been reviewed to remove citation errors and inconsistencies. Finally, we also addressed the remaining minor comments listed below and revised the corresponding text throughout the manuscript. We appreciate the reviewer's careful reading, which helped us improve the consistency, accessibility, and accuracy of the revised review.

3. The review requires a certain domain knowledge to be accessible to the readers. The authors refer to several things that are not explained, e.g., de Bruijn graph-based assembly vs. OLC assembly. This limits the accessibility to the uninformed reader.
Response:
Good suggestion. We revised the Section 2 to clarify the distinction between assembly based on de Bruijn graphs and OLC assembly. Specifically, we added the following explanatory text: "SRS assembly commonly uses approaches based on de Bruijn graphs, in which short reads are decomposed into k-mers and reconstructed by connecting overlapping k-mers. This strategy is computationally efficient, but it becomes ambiguous when complex regions exceed the length of short reads. In contrast, LRS assembly commonly adopts an overlap layout consensus (OLC) approach, in which longer reads are compared directly and ordered according to overlaps between reads to generate a consensus sequence. This enables more reliable reconstruction of complete chromosomal genomes and extrachromosomal elements, including plasmids and circular phage DNA." (Lines 92-101)
In addition, we reviewed the manuscript for other terms that may be difficult for readers who are not specialists in LRS and added brief explanations for SNVs, SVs, Q30 and Q40. Specifically, we added: "This includes single nucleotide variants (SNVs), which are single base differences at specific genomic positions, especially when these variants occur within complex genomic architecture." (Lines 164-166) We also added: "LRS is particularly valuable for resolving structural variants (SVs), which are genomic changes larger than 50 bp, including inversions, duplications, insertions, deletions and translocations." (Lines 171-174). We clarified the notation for microbial epigenetic modifications by revising the text to "Microbial epigenetic modifications, predominantly N6-methyladenine (6mA), 5-methylcytosine (5mC), and N4-methylcytosine (4mC)…" (Lines 415-417). Finally, we clarified sequencing quality scores as follows: "The field is therefore moving toward Q30-class long reads, corresponding to approximately one error per 1,000 bases, as a realistic standard in optimized workflows. Based on this, Q40-grade reads, corresponding to approximately one error per 10,000 bases, are also becoming plausible." (Lines 604-608)

4. This is up to the journal, but I could not find where the authors comply with the requirement of "A summary of use (particularly in the introduction or among methods) needs to be included at the end of the paper, and the outputs should also be included as a supplementary file hosted in GigaDB or other open repositories." The authors only mention that Gemini 3.1 Pro and what for, but not how or what the outputs were.
Response:
We thank the reviewer for noting this requirement. We have provided a more detailed statement in the "Disclosure of use of AI-assisted tools including generative AI" section at the end of the manuscript. The revised disclosure states: "The authors used Gemini 3.1 Pro as an auxiliary tool for language checking and wording suggestions during manuscript preparation. No AI-generated content was directly incorporated into this study without author review and revision." (Lines 744-747) Accordingly, we understand that there is no corresponding AI generated content to submit as a supplementary file.

L60: Why is DNBSEQ listed here, representing the arguably most recent technology in the field? Other approaches, such as by SOLID or IonTorrent, predate DNBSEQ by many years. Hence presenting it in this way is not logical to me.
Response:
We thank the reviewer for pointing this out.Our intention was not to present DNBSEQ as one of the earliest SRS technologies, but to give examples of representative SRS platforms. We have revised the sentence to present these platforms as examples of SRS technologies rather than as a chronological sequence: "However, throughput constraints necessitated the development of short-read sequencing (SRS) technologies, such as Illumina, DNA nanoball sequencing (DNBSEQ), and Ion Torrent."(Lines 58-60)

L79: Where is "MGE" introduced?
Response:
We thank the reviewer for pointing this out. We have revised the Introduction to define mobile genetic elements (MGEs) at their first occurrence in the main text.

L88: Remove "the" from "the microbiological"
Response:
We thank the reviewer for pointing this out. Because we reorganized the Introduction, the original sentence containing "the microbiological" has been removed from the revised manuscript. Therefore, the problematic phrase no longer appears in the text.

L98: What do the authors mean by "integrative perspectives"?
Response:
We apologize that the original wording was not sufficiently clear. Our intention was to refer to the combined discussion of isolate genomics, metagenomic host element linkages, epigenomics and full-length transcriptomics, and technical limitations. To improve clarity, we have revised the sentence and replaced "integrative perspectives" with a more explicit statement: "Together, these perspectives clarify the role of LRS in resolving the genomic and functional diversity of microorganisms." (Lines 85-86)

L106: Not sure the "single" is necessary.
Response:
We thank the reviewer for this comment. We intended to refer to genomes obtained from pure cultured isolates, but agree that "single" overlaps in meaning with "isolate." We have therefore revised "single-isolate" to "isolate" throughout manuscript for consistency.

L134: Not sure what is meant by "high accuracy read per base"?
Response:
We thank the reviewer for pointing this out. We intended to refer to the high nucleotide accuracy of SRS reads, but the sentence repeated an earlier point about the limitation of SRS. We have therefore removed this wording and revised the sentence. "By spanning genomic regions that are difficult to resolve with SRS, these platforms made complete genome assembly of isolates more feasible." (Lines 115-117)

L135: What is specifically meant by "complex regions"? What qualifies as such?
Response:
We thank the reviewer for requesting clarification. Here, "complex regions" meant genomic regions that are difficult to resolve with short reads, including repeated or highly similar sequences and regions affected by structural variation. We revised this sentence to avoid repeating the earlier discussion."By spanning genomic regions that are difficult to resolve with SRS, these platforms made complete genome assembly of isolates more feasible."(Lines 115-117)

L157: The approaches were not "focused" on drafts but there was simply no better solution readily available. The reference to "finished gapless" is redundant with what follows ("structural completeness").
Response:
We thank the reviewer for this helpful comment.We have revised this sentence to avoid implying that draft assemblies were an intentional preference and to remove the redundant phrase "finished, gapless assemblies." The revised sentence now reads: "Collectively, these studies supported a shift in genome quality standards from draft assemblies toward complete microbial reference genomes, reflecting the ability

of LRS to recover genome continuity at the chromosomal level and provide a stronger basis for functional and structural analysis." (Lines 134-137)

L204: Consider removing "where SRS alignment …" as it is redundant with all the rest.
Response:
We agree that this final clause is redundant, as the limitations of SRS have already been thoroughly established in the preceding text. The phrase has been removed.

L249: What do the authors mean by "inference" here?
Response:
We apologize that the original sentence was unclear. We meant that assigning blaKPC and Tn4401 to a particular plasmid based on partial sequence similarity or the detection of a known plasmid backbone in short-read data may be unreliable. We have revised the text to describe the point more explicitly: "Sheppard et al. applied LRS to 17 blaKPC-positive Enterobacteriaceae isolates. This enabled precise localization of blaKPC and its associated transposon Tn4401, clarifying the linked roles of blaKPC-bearing host strain transmission, plasmid transfer across strains and species, and Tn4401 transposition among plasmids in blaKPC dissemination."(Lines 198-201)

L267: Not sure what the "moving away beyond taxonomic inventories" brings in here. This applies to SRS as well as LRS.
Response:
Thank you for this comment. We agree that moving beyond taxonomic inventories is not unique to LRS and that the original sentence did not clearly identify the specific contribution of long-read metagenomics. We have removed this general statement and replaced it with the concrete applications presented in Figure 2 and Table 2, including genome recovery, strain resolution, and localization of functional loci and integrated mobile elements within assembled genomes. We have revised this sentence as "These developments enable long-read metagenomics to recover complete or near-complete genomes, distinguish closely related strains, and determine where functional loci and integrated mobile elements occur within assembled genomes (Figure 2 and Table 2)." (Lines 218-221)

L298: Why are SRS binning strategies "prone to missing low-abundance" but LRS binning strategies are not? In fact, LRS has a much lower sequencing depth, which is a considerable challenge for low-abundance organisms.
Response:
Thank you for identifying this unsupported comparison. After re-evaluating previous related studies, we agree that low-abundance taxa are not inherently more recoverable using LRS. Their recovery is primarily determined by species-specific sequencing depth, and the lower total yield of many long-read datasets may further limit the representation of rare community members. Although long reads can provide greater genomic context when a low-abundance taxon is sufficiently sampled, this should not be interpreted as greater sensitivity for detecting or recovering rare taxa. We have therefore deleted the original comparison between short- and long-read binning here.

L301: Long-read metagenomic sequencing does not "generate" "molecules"; it generates sequences.
Response:
Thanks very much for pointing this out. We have deleted this sentence.

L318: Not sure what is mean by "converted" in this context.
Response:
Thank you for pointing out this ambiguity. We agree that "converted" was imprecise because LRS does not convert marker-gene signals into genomes. Rather, the cited study recovered genome representatives for previously underrepresented taxa and associated complete functional and structural elements with those genomes. We have revised this sentence as "These assemblies also recovered thousands of complete rRNA operons, biosynthetic gene clusters (BGCs), and CRISPR-Cas systems". (Lines 259-261)

L326: "parsing" remains used.
Response:
Thank you for identifying this remaining inconsistency. We have now conducted a full-text search and

replaced all three remaining instances. Specifically, "high-resolution strain parsing" has been changed to "strain-level resolution" in the Abstract and Conclusion, and the Section 3.2 heading has been revised to "Strain-level resolution".

L328: Wrong capitalization at start of sentence.
Response:
Thanks for your comment. We have corrected it.

L425: I do not understand how LRS connects "function to mechanism". If at all, the authors show that mechanism is hard to get to, not least due to epigenetic/posttranscriptional/posttranslational modifications.
Response:
We agree that the phrase "connects function to mechanism" was imprecise and could imply that LRS alone demonstrates molecular mechanism. We have removed this wording and revised this sentence to describe the contribution more directly. The revised text is "Together, these studies show that LRS improves resistome and mobilome analysis by placing functional genes in their genomic background. In short-read datasets, detection of an ARG, virulence gene, or prophage often shows only that the sequence is present. LRS can further indicate whether these loci are located on chromosomes, plasmids, or transposons, which is essential for assessing their host association and potential mobility". (Lines 355-358)

L431: Formatting of heading missing.
Response:
Thank you for pointing this out. The formatting of the heading "3.4 Pathogen genomics and real-time detection" was inadvertently omitted. We have formatted this heading in bold to ensure consistency with the other subsection headings.

L435: Real-time sequencing is a particular feature of Oxford Nanopore Technologies approach, but not common to LRS. Please adjust accordingly.
Response:
We agree with the reviewer that real-time sequencing is a platform-specific feature of Oxford Nanopore Technologies rather than a general property of all LRS platforms. We have revised this sentence as "ONT-based LRS provides a culture-independent route from clinical specimens to pathogen information, making it an important translational extension of genome-resolved microbial genomics. Its distinctive advantage lies in the ability to generate analyzable reads during the run". (Lines 363-366)

L442: What does "mNGS" mean?
Response:
Thank you for pointing this out. We have defined this term by replacing "mNGS" with "metagenomic next-generation sequencing" in the relevant sentence. (Lines 372)

L486: Why is "core" needed here?
Response:
We thank the reviewer for pointing out that the term "core" may imply universal conservation or evolutionary indispensability among microbial epigenetic modifications. We have removed "core" and revised the sentence to refer more generally to microbial epigenetic modifications, including 6mA, 5mC, and 4mC. "Microbial epigenetic modifications, predominantly N6-methyladenine (6mA), 5-methylcytosine (5mC), and N4-methylcytosine (4mC), are associated with restriction-modification (R-M) systems, gene expression, and host-pathogen interactions"(Lines 415-418)

L491: Why is that argument brought up? SRS was never designed to provide single-molecule solution; at least no SRS solution got meaningful adoption/market share for that.
Response:
We appreciate the reviewer's insightful comment. We agree that SRS technologies were not designed to provide single-molecule resolution, and our original wording may incorrectly suggest this comparison. We have revised this section to clarify the distinction between conventional SRS based DNA methylation approaches, which often rely on bisulfite chemical conversion and generally provide population level methylation profiles, and LRS approaches, which preserve native DNA molecules and enable direct modification detection. The revised text can be found in Lines 418-428 as: "Historically, SRS based

approaches for DNA methylation detection have often relied on bisulfite chemical conversion, which can cause DNA degradation and sequence bias. Because methylation profiles are inferred from aggregated sequencing reads, these approaches generally provide population-level methylation profiles rather than direct measurements of individual DNA molecules. In contrast, LRS preserves native DNA molecules and enables direct modification detection: PacBio SMRT utilizes polymerase kinetic characteristics, while ONT detects changes in nanopore ionic current. Both platforms enable simultaneous DNA sequencing and single-molecule methylation detection without PCR amplification or chemical conversion, allowing direct linkage of epigenetic modifications to their genomic context on individual DNA molecules."

L515: "HMW" not introduced.
Response:
Thank you, We have added the full term "high-molecular-weight (HMW) DNA" at its first appearance in the revised manuscript. (Lines 445)

L534: "TSS" and "TTS" not introduced.
Response:
Thank you for pointing this out. We have introduced the full terms "transcriptional start sites (TSSs)" and "transcriptional termination sites (TTSs)" at their first appearance (Lines 467) in the revised manuscript.

L540: What is meant by "defined" here?
Response:
Thank you for pointing this out. We agree that the term "defined" was ambiguous and could be misinterpreted. To improve clarity, we have revised the sentence to emphasize that ONT direct RNA sequencing refined the transcriptional boundaries of previously identified operons, rather than suggesting that the operons themselves were "defined" The revised text in lines 473-475: "For instance, ONT direct RNA sequencing refined the transcriptional boundaries of previously identified operons, revealing extended boundaries for 225 operons in E. coli and 89 operons in S. aureus."

L569: Why "intact", when it already include "HMW" in the sentence? Importantly, how likely is it to recover fully intact DNA, i.e., a full genome as a single, intact molecule?
Response:
We thank the reviewer for raising this point. Our intention was to indicate high-molecular-weight DNA with minimal fragmentation, not recovery of a complete genome as a single intact DNA molecule. To avoid this misunderstanding, we have removed "intact" from the sentence and now refer only to high purity and high molecular weight DNA.

L691: Please use "cMAGs" to be consistent.
Response:
Thanks very much for your suggestion. We have made the corrections and checked it throughout the manuscript.(Lines 277 and 623)

L699: "AI" not introduced. Importantly, assuming "Artificial Intelligence" is meant here, this is a very generic term.
Response:
We agree that "AI" was too generic and had not been introduced. We have revised this sentence as "Machine-learning and deep-learning methods are likely to contribute to this process". (Lines 631)

L766f: Not sure why "gapless" is needed in addition to "completeness".
Response:
We thank the reviewer for pointing out this redundancy. We agree that "gapless" was unnecessary in addition to "completeness," and we have removed it from the sentence.

L768: Why is "microbial" added here when referring to metagenomics?
Response:
We thank the reviewer for pointing this out. The review already focuses on microbial genomics and metagenomics, the word "microbial" was redundant in this sentence. We have therefore removed it.

Cookie Preference Center

We use cookies which are necessary to make our site work. We may also use additional cookies to analyze, improve and personalize our content and your digital experience. For more information, see our Cookie Policy.

You may choose not to allow some types of cookies. However, blocking some types may impact your experience of our site and the services we are able to offer. See the different category headings below to find out more or change your settings.

Allow all

Manage Consent Preferences

Strictly Necessary Cookies

Always active

These cookies are necessary for the website to function and cannot be switched off in our systems. They are usually only set in response to actions made by you which amount to a request for services, such as setting your privacy preferences, logging in or filling in forms. You can set your browser to block or alert you about these cookies, but some parts of the site will not then work.

Functional Cookies

These cookies enable the website to provide enhanced functionality and personalisation. They may be set by us or by third party providers whose services we have added to our pages. If you do not allow these cookies then some or all of these services may not function properly.

Performance Cookies

These cookies allow us to count visits and traffic sources so we can measure and improve the performance of our site. They help us to know which pages are the most and least popular and see how visitors move around the site.

Targeting Cookies

These cookies may be set through our site by our advertising partners. They may be used by those companies to build a profile of your interests and show you relevant adverts on other sites. If you do not allow these cookies, you will experience less targeted advertising.

Cookie List

Clear

Apply Cancel
Consent Leg.Interest

Confirm my choices

Powe
