# Supplementary material for: Promises and pitfalls of long-read sequencing for resolving microbial complexity
Source: Gigascience. 2026 Aug 17;15:giag087. doi: 10.1093/gigascience/giag087 (PMC13543314; doi:10.1093/gigascience/giag087)

GigaScience

Promises and Pitfalls of Long-Read Sequencing for Resolving Microbial Complexity

--Manuscript Draft--

**Manuscript Number:**	GIGA-D-26-00124
**Full Title:**	Promises and Pitfalls of Long-Read Sequencing for Resolving Microbial Complexity
**Article Type:**	Review
**Funding Information:**	Shenzhen Science and Technology Program (KCXFZ20240903094006009) — Dr. Yuanqiang Zou<br>Shenzhen Medical Research Fund (JCYJ20241202124801003) — Dr. Yuanqiang Zou
**Abstract:**	Long-Read Sequencing (LRS) has driven a fundamental transition in microbial genomics, decisively overcoming the assembly fragmentation inherent to short-read sequencing.<br>This review elucidates the transformative impact of LRS across isolate genomics, complex-community metagenomics, and multi-omics domains. By spanning extensive repetitive regions, LRS facilitates the reconstruction of gapless circular chromosomes and precisely resolves mobile genetic elements. In metagenomics, LRS enables high-resolution strain parsing, the recovery of circular metagenome-assembled genomes, and definitive physical linkages between accessory genes and host genomes, advancing research toward mechanistic ecology. Furthermore, the single-molecule, amplification-free properties of LRS provide unprecedented resolution of native epigenetic modifications and full-length transcriptomes. Despite these advancements, widespread implementation remains constrained by multidimensional challenges, including stringent high-molecular-weight DNA requirements, depth deficits, and computational overhead. Consequently, hybrid sequencing strategies remain the contemporary method.<br>As detection technologies and algorithms progress, LRS will establish a contiguous, multidimensional landscape for deciphering microbial ecosystems.
**Corresponding Author:**	Yuanqiang Zou<br>BGI-Shenzhen: BGI Group<br>Shenzhen, CHINA
**Corresponding Author Secondary Information:**	
**Corresponding Author's Institution:**	BGI-Shenzhen: BGI Group
**Corresponding Author's Secondary Institution:**	
**First Author:**	xing rao
**First Author Secondary Information:**	
**Order of Authors:**	xing rao
	Yuzheng Gu
	Gabriella
	Jiayi Ma
	Haoyu Wang
	Yuanqiang Zou
**Order of Authors Secondary Information:**	
**Additional Information:**	
**Question**	**Response**
Are you submitting this manuscript to a	No

Powered by Editorial Manager® and ProduXion Manager® from Aries Systems Corporation

special series or article collection?	
**Experimental design and statistics**<br><br>Full details of the experimental design and statistical methods used should be given in the Methods section, as detailed in our Minimum Standards Reporting Checklist. Information essential to interpreting the data presented should be made available in the figure legends.<br><br>Have you included all the information requested in your manuscript?	No
If not, please give reasons for any omissions below.<br><br>as follow-up to "**Experimental design and statistics**<br><br>Full details of the experimental design and statistical methods used should be given in the Methods section, as detailed in our Minimum Standards Reporting Checklist. Information essential to interpreting the data presented should be made available in the figure legends.<br><br>Have you included all the information requested in your manuscript?<br><br>"	This manuscript is a review article and does not present original experimental data. Therefore, no experimental design or statistical methods are applicable. The manuscript has been prepared in accordance with the journal's guidelines for reviews, and all relevant information has been included.
**Resources**<br><br>A description of all resources used, including antibodies, cell lines, animals and software tools, with enough information to allow them to be uniquely identified, should be included in the Methods section. Authors are strongly encouraged to cite Research Resource Identifiers (RRIDs) for antibodies, model organisms and tools, where possible.	No

Have you included the information requested as detailed in our Minimum Standards Reporting Checklist?	
If not, please give reasons for any omissions below.<br><br>as follow-up to "**Resources**<br><br>A description of all resources used, including antibodies, cell lines, animals and software tools, with enough information to allow them to be uniquely identified, should be included in the Methods section. Authors are strongly encouraged to cite Research Resource Identifiers (RRIDs) for antibodies, model organisms and tools, where possible.<br><br>Have you included the information requested as detailed in our Minimum Standards Reporting Checklist?<br><br>"	This manuscript is a review article and does not present original experimental data. Therefore, no experimental design or statistical methods are applicable. The manuscript has been prepared in accordance with the journal's guidelines for reviews, and all relevant information has been included.
**Availability of data and materials**<br><br>All datasets and code on which the conclusions of the paper rely must be either included in your submission or deposited in publicly available repositories (where available and ethically appropriate), referencing such data using a unique identifier in the references and in the "Availability of Data and Materials" section of your manuscript.<br><br>Have you have met the above requirement as detailed in our Minimum Standards Reporting Checklist?	No
If not, please give reasons for any omissions below.<br><br>as follow-up to "**Availability of data and materials**	This manuscript is a review article and does not present original experimental data. Therefore, no experimental design or statistical methods are applicable. The manuscript has been prepared in accordance with the journal's guidelines for reviews, and all relevant information has been included.

All datasets and code on which the conclusions of the paper rely must be either included in your submission or deposited in publicly available repositories (where available and ethically appropriate), referencing such data using a unique identifier in the references and in the "Availability of Data and Materials" section of your manuscript.<br><br>Have you have met the above requirement as detailed in our Minimum Standards Reporting Checklist?<br><br>"	
GigaScience has policies and guidelines in place for the use of generative AI-writing tools such as ChatGPT. If you have used such writing tools to assist with writing the manuscript this must be declared and cited in the text. Authors should not list AI-writing tools and other AI-assisted technologies as an author or co-author and should acknowledge that they are fully responsible for text generated or refined by AI-writing tools.<p><br><br>A summary of use (particularly in the introduction or among methods) needs to be included at the end of the paper, and the outputs should also be included as a supplementary file hosted in GigaDB or other open repositories. Please read our guidelines for more information. <p><br><br>By submitting to GigaScience, you are aware of the journal's AI-writing tools policy, and if you have declared use of such tools below, you have acknowledged this where appropriate in your manuscript and have made a summary of use and outputs available. <p><br><b>AI-assisted writing tools have been used in the preparation of this manuscript?	Yes

Manuscript Click here to access/download;Manuscript;manuscript_LRS.docx

Promises and Pitfalls of Long-Read Sequencing for Resolving Microbial Complexity

Xing Rao[1,2#], Yuzheng Gu[3#], Gabriella[1,4], Jiayi Ma[1,2], Haoyu Wang[1,2], Yuanqiang Zou[1,5]

1. State Key Laboratory of Genome and Multi-omics Technologies, BGI Research, Shenzhen 518083, China

2. College of Life Science, University of Chinese Academy of Sciences, Beijing 100049, China

3. Key Laboratory of Agricultural and Environmental Microbiology, Ministry of Agriculture and Rural Affairs, Department of Microbiology, College of Life Sciences, Nanjing Agricultural University, Nanjing 210095, China

4. Department of Food Science and Biotechnology, Faculty of Agricultural Technology, Universitas Brawijaya, Malang 65145, Indonesia

5. Shenzhen Key Laboratory of Human commensal microorganisms and Health Research, BGI Research, Shenzhen 518083, China

# These authors contributed equally.

Corresponding author:

Yuanqiang Zou.

Telephone: +86-13147016380

Fax: 0755-36352579

Address: State Key Laboratory of Genome and Multi-omics Technologies, BGI

Research, Shenzhen 518083, China

Email: zouyuanqiang@genomics.cn

Yuanqiang Zou will handle the correspondence of the article at all stages of the review and publication process, as well as post-publication.

Author's email: raoxing@genomics.cn, guyuzheng513@163.com, gabriella@genomics.cn, majiayi@genomics.cn, wanghaoyu2@genomics.cn , zouyuanqiang@genomics.cn

Abstract

Long-Read Sequencing (LRS) has driven a fundamental transition in microbial genomics, decisively overcoming the assembly fragmentation inherent to short-read sequencing.

This review elucidates the transformative impact of LRS across isolate genomics, complex-community metagenomics, and multi-omics domains. By spanning extensive repetitive regions, LRS facilitates the reconstruction of gapless circular chromosomes and precisely resolves mobile genetic elements. In metagenomics, LRS enables high-resolution strain parsing, the recovery of circular metagenome-assembled genomes, and definitive physical linkages between accessory genes and host genomes, advancing research toward mechanistic ecology. Furthermore, the single-molecule, amplification-free properties of LRS provide unprecedented resolution of native epigenetic modifications and full-length transcriptomes. Despite these advancements,

widespread implementation remains constrained by multidimensional challenges, including stringent high-molecular-weight DNA requirements, depth deficits, and computational overhead. Consequently, hybrid sequencing strategies remain the contemporary method.

As detection technologies and algorithms progress, LRS will establish a contiguous, multidimensional landscape for deciphering microbial ecosystems.

Keywords: Long-read sequencing, Microbial genomics, Metagenomics, Genome assembly.

1. Introduction

The evolution of sequencing technologies is required to resolve the structural complexity of microbial genomes at molecular resolution. From Sanger sequencing to single-molecule real-time platforms, each technical transition reflects improvements in throughput, cost efficiency, and analytical scope. Sanger sequencing, characterized by dideoxynucleotide chain termination, enabled the first genome draft assembly of a cultured microorganism, *Haemophilus influenzae*, marking the formal entry of microbiology into the genomic era[1, 2]. However, throughput constraints necessitated the development of short-read sequencing (SRS) platforms such as Illumina and DNA nanoball sequencing (DNBSEQ) [3, 4]. By integrating bridge amplification with sequencing-by-synthesis, LRS circumvented the need for microbial cultivation and supported landmark metagenomic initiatives like the Human Microbiome Project and METAgenomics of the Human Intestinal Tract[5-7]. Despite these advances, the short-read lengths often fail to resolve intricate genomic

architectures, resulting in fragmented assemblies[8]. LRS platforms, exemplified by PacBio, Oxford Nanopore Technologies (ONT) and CycloneSEQ, generate read lengths that exceed SRS capabilities by several orders of magnitude, effectively addressing the above assembly bottlenecks[9-11]. This progress enables the transition from fragmented draft assemblies to the reconstruction of high-quality, circularized chromosomes[12-14], surmounting the structural barriers that have historically hindered contiguous assembly in SRS workflows.

The inability of SRS to achieve gapless genome closure stems from the inherent scale disparity between short-read lengths and the structural complexity of microbial chromosomes. Central to this challenge is the high density of repetitive elements, which can constitute up to 10% of some bacterial genomes and thus present formidable barriers to computational reconstruction[15-17]. These regions, primarily composed of ribosomal RNA (rRNA) operons and insertion sequences (ISs), often exhibit sequence lengths and identities that exceed the disambiguation capacity of SRS algorithms[17-19]. Furthermore, the ubiquity of mobile genetic elements, such as plasmids and prophages, complicates reconstruction because short reads frequently fail to establish their physical linkage with the primary chromosome[20, 21]. Compositional challenges, including extreme guanine–cytosine(GC) content, further exacerbate assembly difficulties by introducing systematic coverage biases and stochastic gaps[22-24]. Collectively, these intrinsic genomic features result in highly fragmented draft assemblies that impede accurate phylogenetic inference and the comprehensive functional annotation of bacterial genomes.

In response to these structural bottlenecks, LRS has emerged as a transformative force, shifting the microbiological research paradigm from fragmented observations toward holistic genomic resolution. However, current reviews have typically centered on biomedical surveys or algorithmic analyses[25-27], frequently overlooking the pragmatic integration of multi-scale microbial research. Our analysis spans pivotal microbiological applications, encompassing the reconstruction of gapless isolate genomes, the resolution of complex metagenomic populations, and the characterization of epigenomic and full-length transcriptomic landscapes. In particular, we also offer a critical evaluation of the technical disadvantages of LRS relative to SRS. By synthesizing these integrative perspectives, this work establishes a strategic framework for microbial research, underscoring the necessity of LRS in resolving the intricate complexity of diverse microorganisms.

2. Genomics of single isolates: reshaping the standard of "reference genomes."

The standard for high-quality reference genomes has shifted from base accuracy to requiring structural completeness (Figure 1). This change is driven by broader access to analytical tools and reduced sequencing costs[28-31]. Historically, single-isolate microbial reference genomes relied heavily on SRS platforms[32]. In SRS, DNA strands are fragmented into short pieces, which are then sequenced individually and assembled into a genome using computational bioinformatic tools. This method generates draft assemblies with high read accuracy to the original DNA[28, 29, 33]. However, a high number of short fragments creates gaps and causes

loss of reads in complex repetitive regions that cannot be resolved unambiguously[34]. Algorithms for SRS, often based on De Bruijn graphs, struggle with these repetitive sequences. The result is gaps, misassemblies, and ambiguous contig ordering[35]. In microbial genomes, these problems are seen in loci such as rRNA operons, ISs, and duplicated elements. Supporting this, Utturkar et al. showed most SRS assembly gaps come from repetitive DNA, especially multiple rRNA operons[36]. The introduction of LRS has overcome this bottleneck. LRS platforms provide reads long enough to span megabase-scale regions, enabling single circularized chromosomes and full reconstruction of extrachromosomal elements[37].

Figure 1 Comparative assembly of single-isolate microbial genomes using SRS and LRS. Conventional SRS implementations generate highly fragmented draft genomes, inherently constrained by their inability to resolve complex repetitive elements, differentiate extrachromosomal plasmids, or capture SVs. Conversely, LRS harnesses extended read lengths to span these challenging structural complexities, yielding gapless, complete genomes that ensure precise spatial positioning, definitive plasmid resolution, and comprehensive SV capture for downstream genomic interpretations.

Alt text: A comparative flowchart on microbial genome assembly using SRS and LRS, illustrating the technical transition from fragmented draft genomes to gapless, complete circularized chromosomes and resolved genomic features.

2.1. Achieving gapless genomes: from fragmentation to continuity

The commercialization of LRS platforms, initiated by PacBio SMRT in 2011 and followed by ONT MinION in 2014, represented a milestone in third-generation sequencing technologies[38]. LRS platforms generate reads spanning tens to hundreds of kilobases, bridging repetitive regions. These capabilities did not simply improve microbial assemblies incrementally, but fundamentally updating the attainable standard for single-isolate reference genomes (Table 1). In SRS, even datasets with high-accuracy read per base frequently yielded fragmented draft assemblies since reads are too short to span complex regions[36].

Chin et al. showed that PacBio SMRT sequencing could resolve long repeat regions and produce *de novo* microbial genome assemblies with over 99.999% consensus accuracy[39]. This demonstrated that finished genomes no longer required labor-intensive finishing strategies. Koren et al. assembled six bacteria and analyzed repeat complexity across 2,267 complete bacterial and archaeal genomes[40]. They concluded that most microbial genomes should be closeable without gaps using a single PacBio RS library. The advantage of LRS was not limited to contiguity; ultra-long reads exceeding 32 kb enabled sequencing of entire adhesin-encoding genes (>6 kb) and antibiotic biosynthesis loci (>15 kb), regions previously difficult to reconstruct with short-read data[41]. As reductions in sequencing cost and improvements in LRS throughput expanded the potential of complete-genome reconstruction, these benefits became apparent at reference-collection scale. This has been illustrated by a recent human gut resource comprising 1,150 circular genomes

from 1,093 isolates across 199 species-level clusters[42]. In turn, the improved completeness of these genomes strengthened downstream functional inference, as metabolic models based on complete genomes recovered 4,764 additional reactions relative to draft-based models, most of them transport-related[43].

Collectively, these studies catalyzed a shift in genome quality standards—from a draft-focused approach to a new expectation of finished, gapless assemblies. This transition was anchored in the recent ability to recover genome continuity at the chromosomal level, thus redefining reference genome quality beyond conventional contiguity metrics. This shift established structural completeness as a defining feature of high-quality reference genomes and provides the basis for further discussion for high-confidence functional and structural analysis.

2.2. Beyond the chromosome: unveiling the full architecture of plasmids and mobile genetic elements

Although SRS platforms generate accurate reads of 100-300 bp that are well suited for identifying conserved genomic regions, a major drawback of reads produced by SRS is that they are too short to disentangle extrachromosomal sequences sharing homologous regions, insertion sequences, or transposon-associated repeats[44]. Short-read plasmid reconstruction remained unreliable in a benchmark of 42 complete bacterial genomes containing 148 plasmids, particularly for large plasmids with repetitive regions[45]. Microbial genomes extend beyond the chromosome; plasmids, transposons, ISs, integrons, integrative and conjugative

elements (ICEs), resistance islands, and prophages constitute the mobile genetic element (MGE) repertoire that drives horizontal gene transfer (HGT)[46]. As a result, accessory elements, such as antimicrobial resistance (AMRs), antibiotic resistance genes (ARGs), and virulence, may be fragmented, merged, or incorrectly assigned, which may lower the accuracy and credibility of the analysis results[44, 47].

LRS has enhanced the landscape by enabling direct assembly of entire plasmids as individual replicons, preserving the associated MGE context. Li et al. used PacBio LRS on 134 multidrug-resistant *Salmonella enterica* isolates, producing 285 plasmid sequences[48]. Of these, 245 were fully resolved as circular structures, demonstrating large-scale recovery of plasmid architecture with LRS. Jiang et al. reconstructed resistance plasmid architecture using PacBio SMRT sequencing, characterizing pooled conjugative plasmids from multidrug-resistant *Klebsiella pneumoniae*[49]. They identified 124 ARGs and 317 MGEs, including transposons, ISs, and integrons, and established a relationship network between MGEs and ARGs that described dissemination by mobile elements. Key resistance transposition elements included IS26, Tn3, IS903B, ISEcp1, and ISKpn19, and they illustrated an IS26-mediated multicopy composite transposition event. ONT R10.4.1 data recently showed that finished bacterial and plasmid genomes can be generated without SRS, achieving accuracies above 99.99% for a 5-Mb bacterial genome at 75× coverage[50]. Beyond structural resolution, reconstructed plasmids also revealed biological relevance. Wang et al. showed that resolved plasmid-borne genes included functions related to fructose metabolism, indicating that accessory replicons can contribute directly to

host-associated metabolic capacity[42].

2.3. Resolving microbial structural variations and evolutionary dynamics

The use of LRS technologies makes it easier to identify a broader spectrum of structural variations (SVs). In bacteria, these are genomic rearrangements exceeding 50 bp, many of which remain inaccessible or challenging to resolve through short-read methods alone[51, 52]. Although one of the clearest advantages of LRS lies in resolving large inversions, duplications and other structural variants, they also improve the detection and interpretation of smaller-scale variants such as SNVs. Recent ONT-based benchmarking showed that Illumina- and k-mer-based SNP typing failed to detect 9 to 68 SNPs per 1,000 sites in repetitive reference regions, whereas ONT long reads generated perfect SNP calls across the full genome in simulated bacterial read sets[53]. Capturing this broader view is essential as it shows that microbial evolution depends not just on massive structural shifts, but also on small, localized mutations and shifting repeat regions[54]. Recently, Waters argued that genome structure variation should be considered part of genome-based bacterial surveillance, but this requires complete circularized genomes[55]. In case studies of *Salmonella enterica* serovar Typhi, researchers confirmed genome rearrangements generated during long-term culture using LRS. All isolates with altered genome structures had changes in gene expression compared to the parental strain[55]. The most extreme rearrangement caused a stronger imbalance between the origin and terminus of replication and was associated with more pronounced transcriptional changes, showing that chromosome-scale structural variation can influence phenotype

even when nucleotide-level sequence changes are limited. Similarly, in *Bordetella pertussis*, LRS confirmed both duplications and a large inversion of approximately 1 Mbp between isolates from the same patient. This demonstrates that large rearrangements can arise and persist during both within-host and laboratory evolution[56]. LRS also helps detect microbial SVs by resolving genome-scale rearrangements and mobile-element-associated chromosomal changes that are difficult to reconstruct from fragmented assemblies. In *Salmonella enterica*, LRS of 134 multidrug-resistant isolates revealed variation in the presence and orientation of *Salmonella* pathogenicity islands, as well as chromosomal insertion of resistance plasmids and genomic islands. This highlights the value of LRS in resolving genome plasticity and resistance-associated structural changes[48].

On an evolutionary scale, the value of LRS is reinforced by population-level analyses. Canalda-Baltrons et al. constructed a pangenome reference graph from 859 high-quality LRS assemblies of *Mycobacterium tuberculosis* and showed that SVs are not marginal events, but an important component of genome evolution, virulence-associated deletions, and drug-resistance architecture[52]. Their subsequent genotyping across 41,134 isolates linked recurrent SVs to multiple first- and second-line drug resistance phenotypes, underscoring that long-read-enabled SV analysis contributes not only to structural characterization, but also to understanding clinically relevant adaptation. Taken together, these studies show that the key contribution of LRS is not merely improved contiguity, but the ability to recover chromosome-scale structural change with sufficient precision to study its functional

and evolutionary consequences.

2.4 Precise localization of genetic elements through complete genomes

The precise localization of MGEs provides a critical window into understanding of microbial organisms. Once chromosomes and accessory replicons are fully assembled, genetic elements that would otherwise appear as disconnected fragments in draft genomes can be assigned to a defined chromosomal or plasmid backbone. Sheppard et al. used a hybrid whole-genome sequencing (WGS) approach to analyze 281 blaKPC-positive Enterobacteriaceae isolates and showed that blaKPC dissemination occurred through multiple linked genetic levels, including movement of host strains between patients, plasmid transfer across strains and species, and transposition of Tn4401 between plasmids[57]. In this setting, complete plasmid structures revealed how resistance dissemination depends on the organization of elements across strains, plasmids and transposons, and showed why partial typing or reference-based inference can misrepresent the true pathways of gene spread. In a multidrug-resistant *Chryseobacterium indologenes* isolate, genome-resolved analysis localized 13 genomic islands together with an ICE, ISEIsp1, Tn5393 and In31 within the chromosome, clarifying the structural context of resistance- and virulence-associated loci[58]. At larger scale, Benler et al. delineated 6,549 Tn7-like transposons at base-pair resolution and showed that 96% carried cargo genes enriched not only in defense and antibiotic resistance functions but also in central carbon metabolism[59]. Han et al. further highlighted the importance of this organization in *Klebsiella pneumoniae*, where gene cassettes, integrons, transposons, and conjugative

elements are hierarchically embedded within one another[60]. Together, these findings show that complete genome assemblies both recover more sequence, and reveal the genomic context required to understand mobility, adaptation, and pathogenic potential.

3. Metagenomics of complex communities: from "fragments" to "panoramas"

Metagenomics has vastly expanded our understandings of the diversity and metabolic potential of uncultured microorganisms. However, analytical strategies based on short-read metagenomic sequencing have persistently faced a fundamental computational bottleneck. When assembly algorithms (e.g., De Bruijn graphs) process ubiquitous features in microbial genomes such as rRNA operons, long tandem repeats, ISs, and highly homologous regions shared among closely related taxa, assembly graphs tend to fragment or generate chimeric paths[61, 62]. As a result, metagenomic datasets are often reduced to fragmented contigs, and functional genes become detached from the chromosomal, plasmid or phage contexts that determine their biological significance[61]. Recent improvements in the throughput and raw-read accuracy of LRS, together with long-read-aware assemblers, polishers and binning frameworks, have begun to relax this constraint[62, 63]. Complex-community metagenomics is therefore moving beyond taxonomic inventories towards a more integrated framework in which complete genome architecture, DNA modifications, and ecological interactions can be interrogated within the same datasets (Figure 2).

Figure 2 Transformative applications of long-read metagenomics in complex microbial ecosystems. This schematic delineates the multidimensional analytical capacities unlocked by LRS to overcome the fragmentation limitations of conventional metagenomics. LRS facilitates the direct reconstruction of cMAGs, thereby significantly expanding the known phylogenetic diversity. Concurrently, the recovery of intact functional loci, such as BGCs and CRISPR systems, enables robust metabolic and functional profiling. High-resolution haplotypic parsing further allows for the dynamic monitoring of strains and SVs that drive microdiversity. Furthermore, LRS establishes definitive physical linkages between host chromosomes and MGEs (plasmids, phages, and ARGs), elucidating the interaction network of HGT.

Alt text: A four-panel schematic illustrating the transformative impact of LRS on metagenomics. It highlights the transition from fragmented data to cMAGs for diversity expansion, the recovery of intact functional loci for metabolic profiling, strain-level tracking of structural variations, and the establishment of physical host-element linkages to map horizontal gene transfer networks.

3.1 Circular metagenome-assembled genomes

One immediate consequence of this shift is the increasing feasibility of recovering complete or near-complete genomes directly from complex communities. High-quality metagenome-assembled genomes (MAGs) provide the structural basis for metabolic reconstruction, comparative genomics and ecological interpretation. However, in highly heterogeneous ecosystems, such as soils and aquatic environments,

species abundance spans a broad dynamic range. Under these conditions, short-read binning strategies are prone to missing low-abundance but ecologically informative taxa and often fail to reconstruct highly fragmented sequences into closed genomes[61, 64]. Long-read metagenomic sequencing addresses this limitation by generating contiguous molecules that span tens to hundreds of kilobases, thereby enabling recovery of c**ircular metagenome-assembled genomes (**cMAGs) and rescuing genomic regions that are routinely lost from short-read assemblies because of structural complexity.

Early nanopore-based studies showed that closed bacterial genomes could be recovered directly from human faecal metagenomes, indicating that complete community genomes were no longer restricted to isolate sequencing[65]. At population scale, this additional continuity translates directly into a more complete view of microbiome diversity. In a study of Southeast Asian gut microbiomes integrating LRS with Hi-C, more than 1,700 MAGs were absent from short-read-only analyses, assembly contiguity improved by more than 28-fold, and thousands of population-specific strains and biosynthetic gene clusters were recovered[64]. Similar gains have been reported outside the human gut. In activated sludge, hybrid long- and short-read approaches generated more than 1,000 high-quality MAGs, including dozens of circular genomes, and restored full-length rRNA genes that are typically absent from short-read bins[66]. More recently, deep long-read surveys across terrestrial habitats recovered genomes representing more than 15,000 previously undescribed genomes and expanded the known phylogenetic breadth of prokaryotic

diversity, indicating that long-read metagenomics contributes not only to improved assembly of known taxa, but also to recovery of lineages that remain poorly represented in short-read-centred reference frameworks[67]. Surprisingly, this study enabled the recovery of thousands of complete ribosomal RNA operons, biosynthetic gene clusters (BGCs) and CRISPR-Cas systems, highlighting that LRS at scale can now move beyond genome recovery and into systematic discovery of the repetitive, structurally complex, and functionally critical elements that define microbial adaptability and evolution.

3.2 High-resolution strain-level parsing

Long-read metagenomics is also redefining strain resolution in complex microbial communities. Natural microbiomes often contain multiple closely related lineages whose genomic differences are encoded by not only single-nucleotide variants, but also accessory gene cassettes, inversions, prophage carriage, tandem duplications and large insertions. Under such conditions, short reads frequently collapse related strains into species-level consensus representations or require statistical deconvolution that remains sensitive to reference bias and assembly ambiguity[62, 68]. LRS alleviates this problem because long molecules preserve haplotypic linkage and can traverse structurally variable loci that would otherwise disrupt assembly graphs.

In sheep faecal microbiomes, deep sequencing using long reads enabled recovery of lineage-resolved complete MAGs and phasing of closely related microbial

haplotypes across long genomic distances, thereby revealing hidden population structure and improving assignment of biosynthetic gene clusters and mobile elements to specific hosts[69]. In the human gut microbiome, long-read-enabled analyses further showed that SV is highly individualized and contributes substantially to within-species diversity[68]. This is important because many ecologically relevant phenotypes are encoded by multi-kilobase genomic segments that are poorly represented in short-read consensus assemblies.

The clinical relevance of this enhanced resolution is becoming increasingly apparent. In faecal microbiota transplantation (FMT), therapeutic interpretation depends not only on whether a species is detected after treatment, but also on which donor strain engrafts and persists in the recipient gut. Long-read metagenomics enables post-FMT strain tracking with substantially greater specificity than short-read approaches, particularly when multiple conspecific strains coexist in the same sample. In cohorts with recurrent *Clostridioides difficile* infection and inflammatory bowel disease, long-read analyses identified hundreds of engrafted donor strains and tracked their persistence for up to five years, while also capturing epigenetic stability and large-scale SVs acquired during adaptation to the recipient environment[70]. In a paediatric undernutrition cohort, Minich et al. used long-read metagenomics to follow the gut microbiota over 11 months and linked SV in commensal strains to host growth-associated phenotypes, suggesting that clinically relevant microbiome signals may depend on fine-scale genomic plasticity rather than taxonomic composition alone[71]. These findings indicate that strain resolution should not be viewed as a

technical refinement of species-level profiling, but as a shift towards a more appropriate biological scale. Many clinically and ecologically relevant processes, including engraftment, persistence, competition and adaptation, operate at the level of strains rather than species. Long-read metagenomics therefore offers a framework in which microbial population dynamics can be interpreted in terms of inheritance, selection and genome plasticity, rather than inferred indirectly from abundance changes alone.

3.3 Establishing host-element linkages

One of the most important contributions of long-read metagenomics to microbial ecology is its ability to bridge the assembly gap between MGEs and their host chromosomes. In short-read datasets, the flanking regions of ARGs, virulence factors, prophages and plasmids are often enriched in transposases and ISs, causing these loci to be isolated on short fragments within assembly graphs[61, 72]. Consequently, researchers can quantify the abundance of these elements, yet still lack direct evidence for their host range or transfer potential. LRS addresses this problem by spanning the repetitive boundaries that separate functional loci from their chromosomal or plasmid backbones[72, 73].

This capability has been particularly valuable in resistome analysis. In an early proof-of-principle study, combining long-read assembly with proximity ligation substantially increased recovery of ARGs and virus-host associations in the rumen microbiome relative to short-read assembly alone[72]. Works in wastewater treatment

plants, activated sludge systems and urban sewage networks showed that LRS can determine whether specific ARGs are stably chromosome-encoded or carried by conjugative plasmids, while resolving how host associations and mobility profiles change along environmental gradients[73-75]. Long-read datasets have likewise clarified the effects of local selection pressures on MGE dynamics. In hospital wastewater, chlorine disinfection was associated with altered community structure, increased ARG diversity and higher mobility potential within the resistome[76]. In microplastic-associated biofilms, plasmid-borne ARGs were enriched relative to surrounding water and natural rock substrates, suggesting that artificial polymer surfaces may act as hotspots for plasmid-mediated resistance transfer[77]. These works imply that LRS connects function to mechanism. In short-read datasets, the detection of an ARG, virulence determinant or prophage often supports only descriptive inference. Once these elements are placed onto specific chromosomes or plasmids, however, the same data begin to inform questions of mobility, host range, persistence, and transmission. Long-read metagenomics therefore shifts resistome and mobilome analysis from compositional surveillance towards mechanistic ecology.

LRS is also refining our understanding of the interplay between phages, plasmids and hosts in complex communities. In the human gut, long-read metagenomics enabled direct recovery of complete extrachromosomal mobile genetic elements, revealing plasmids and phage-associated DNA that are underrepresented in short-read catalogues[78]. In the oral microbiome, long-read assembly uncovered a family of giant plasmid-like extrachromosomal elements associated with *Streptococcus*,

indicating that mobile gene pools in host-associated communities have been substantially underestimated when inferred from fragmented assemblies alone[79]. Long reads have also sharpened analyses of phage ecology. In a two-year longitudinal study of the human gut microbiome, Wirbel et al. found that prophage-host relationships were more flexible than predicted by a strictly host-specific model: bacterial lineages with and without particular prophages could coexist stably, and some phages were detected in distinct host lineages across family boundaries[80]. The same study identified a class of phages termed "IScream" phages, which appear to exploit bacterial IS30 transposases to mediate their own mobility and integration. These findings demonstrate that the mobilome is more dynamic and cross-lineage than previously appreciated, and that long-read sequencing now can be the primary tool for resolving this complexity.

4. Extended applications: multi-omics beyond DNA sequences

LRS has thoroughly shattered the fragmentation bottleneck inherent to SRS, enabling complete single-isolate genome assemblies, cMAGs and the precise linkage of AMR and virulence genes to their host genomes[81]. However, microbial phenotypes—such as antibiotic resistance, virulence, and environmental adaptability—are not dictated solely by linear DNA sequences. Epigenetic modifications and dynamic transcriptomic regulation serve as crucial bridges connecting genotype to phenotype. SRS presents insurmountable natural barriers in these dimensions. In contrast, LRS, driven by its single-molecule, long-fragment, and amplification-free core properties, achieves breakthrough multi-omics resolution. This

section systematically elucidates the technical advantages and frontier applications of LRS in three core fields: microbial epigenomics, full-length transcriptomics and single-molecule proteomics (Figure 3).

Figure 3 Paradigm shifts in microbial multi-omics driven by single-molecule sequencing. This schematic contrasts the analytical mechanisms of conventional assays against single-molecule technologies across three core omics dimensions. Epigenomics: Conventional epigenetic profiling necessitates chemical conversions (e.g., bisulfite treatment), which induce severe DNA damage and sequence bias. In contrast, LRS enables the direct, amplification-free detection of diverse native modifications (such as 6mA, 5mC, and 4mC) via nanopore current disruptions. Transcriptomics: The inherent read-length constraints of SRS result in fragmented assemblies, precluding the recovery of contiguous physical linkage within polycistronic operons. Conversely, LRS achieves full-length transcript identification, capturing contiguous RNA molecules spanning intact multi-gene organizations from TSS to TTS. Proteomics: Compared with traditional mass spectrometry (MS), next-generation nanopore proteomics facilitates the direct sequencing of translocated peptide strands at single-molecule resolution.

Alt text: A three-panel schematic comparing conventional and single-molecule multi-omics. It illustrates the shift from chemical-based epigenetics to direct modification sensing, from fragmented transcripts to full-length operon sequencing,

and from mass-spectrometry-based proteomics to direct nanopore protein sequencing.

4.1 Microbial epigenomics: from native signals to regulatory landscapes

Microbial core epigenetic modifications—predominantly 6mA, 5mC, and 4mC—are fundamental mechanisms governing restriction-modification (R-M) systems, gene expression, and host-pathogen interactions. Historically, SRS relied on bisulfite chemical conversion, which frequently causes DNA degradation and severe GC bias. It fails to cover extreme GC regions and repetitive sequences (e.g., rRNA operons, IS elements, and plasmids), thus failing to achieve simultaneous "modification-sequence" detection on a single molecule. SRS only captures a population-averaged signal, masking strain-level epigenetic heterogeneity. LRS fundamentally resolves this issue: PacBio SMRT utilizes polymerase kinetic characteristics , while ONT detects nanopore ionic current disruptions. Both platforms accomplish simultaneous DNA sequencing and single-molecule methylation detection without PCR amplification or chemical conversion, thoroughly breaking the SRS barrier[82]. Today, LRS is extensively applied to methylation detection, complete R-M system resolution, and the linking of epigenetics to phenotypic regulation. Notably, the two platforms exhibit divergent strengths: PacBio HiFi approaches the gold standard for 6mA detection sensitivity, whereas recent updates like ONT's R10.4.1 pore have drastically improved 5mC and 4mC accuracy, making it highly suitable for cost-effective, large-scale screening[83, 84].

Compared to short reads, LRS precisely matches restriction

endonuclease-encoding genes with their corresponding methylation motifs[85]. This precise anchoring allows for the complete resolution of R-M systems carried by plasmids and prophages, revealing their core roles in HGT and phage defense[86, 87]. Furthermore, LRS maps methylation modifications onto specific functional elements (promoters, operons, and mobile genetic elements), elucidating the regulatory impact of single-base modifications on gene expression. In complex microbial communities, long-read meta-epigenomics reconstructs strain-level methylation profiles, precisely associating mobile elements with specific host strains and breaking the species-resolution bottleneck of short-read meta-epigenomics[88]. However, current technical limitations persist, such as the difficulty of extracting high-molecular-weight (HMW) DNA from complex environmental samples and ONT's insufficient accuracy in detecting very low-abundance modifications.

4.2 Full-length transcriptomics: reshaping operons and isoforms

The bacterial transcriptome is primarily orchestrated around polycistronic operons, encompassing an abundance of alternative transcripts, antisense RNAs, and non-coding RNAs that mediate responses to environmental stress and govern virulence. Traditional RNA sequencing based on SRS generates short fragments of 50 to 150 bases. To infer complete transcript structures, researchers must rely on complex bioinformatics algorithms to "stitch" these massive fragments together. This process is computationally intensive and highly prone to assembly errors, and susceptible to severe sequence biases[89]. Long-read RNA sequencing (e.g., PacBio Iso-Seq or ONT direct RNA/cDNA sequencing) fundamentally solves this by

generating reads spanning thousands of bases, covering intact RNA molecules in a single pass[87, 90]. This "read-is-transcript" characteristic eradicates the reliance on algorithmic patchwork, significantly enhancing annotation accuracy and providing unbiased quantification[91].

Accurate delineation of operon boundaries (i.e., transcriptional start sites, TSS; transcriptional termination sites, TTS), internal gene organization, and intra-operonic co-transcriptional relationships is critical for deciphering bacterial gene regulatory mechanisms. By sequencing the entire length of polycistronic mRNAs, LRS physically links multiple genes onto a single molecule. This structural precision replaces predictive models with definitive physical maps of active transcription units[92, 93]. Furthermore, capturing full-length transcripts systematically identifies diverse transcriptional isoforms driven by alternative TSS/TTS and internal promoters. Beyond operons, LRS drastically enhances the identification of non-coding regulatory elements, such as antisense RNAs and overlapping genes. LRS provides unambiguous strand-specific information to accurately annotate these genomic "regulatory dark matters" that typically confound short-read assemblers[94]. Notably, the direct RNA sequencing technology of ONT has also inaugurated the era of "Epitranscriptomics", enabling direct detection of chemical modifications (such as m6A) on native RNA molecules, adding an entirely new functional dimension to post-transcriptional regulation[95].

4.3 Single-molecule proteomics: direct nanopore protein sequencing

Proteins are the ultimate executors of biological activities. Achieving rapid and accurate high-resolution characterization of protein and their constituent peptides remains a critical challenge in biotechnology and microbiomics[96]. In recent years, nanopore-based protein characterization has emerged as one of the most promising next-generation analytical strategies. However, formidable technical bottlenecks, including difficulties in sample preparation, complex molecular manipulation, and signal decoding, have long restricted the practical application of nanopore technology to direct peptide detection[96, 97].

To transcend these limitations, the frontier of multi-omics is rapidly advancing toward direct, single-molecule protein sequencing using nanopore technologies[98]. Analogous to long-read DNA sequencing, this paradigm involves translocating unfolded protein or peptide strands through engineered nanopores to detect sequence-specific ionic current disruptions. Recent landmark breakthroughs have proven this historically elusive concept highly feasible. For instance, utilizing molecular unfoldases for the multi-pass, single-molecule nanopore reading of long protein strands has drastically improved amino acid resolution and reading accuracy[99]. Concurrently, innovations in terminal conjugation chemistry to control peptide translocation speeds[98], alongside massively parallel sensing arrays, are enabling high-throughput peptide profiling and *de novo* protein identification[98, 100].

Although direct nanopore proteomics is still in its nascent for complex metagenomic applications, its trajectory is clear, bypassing MS bottlenecks, allowing

for the direct reading of strain-specific metabolic enzymes, intact virulence factors, and dynamic post-translational modification landscapes at single-molecule resolution. Ultimately, this will close the multi-omics loop, providing accurate physical mapping from the microbial genome to its expressed phenome.

5. Multidimensional technical challenges in long-read sequencing

As delineated in preceding sections, LRS has catalyzed a profound paradigm shift across isolate genomics, metagenomics, and multi-omics dimensions, elevating the analytical resolution of microbiology from fragmented observations to holistic systemic insights. Nevertheless, despite its exceptional potential to reconstruct complex genomic architectures, the systematic application of LRS in large-scale sequencing studies remains severely impeded by cost-effectiveness limitations[25, 101]. Crucially, this economic burden extends far beyond direct sequencing expenses (such as reagents and instruments)[102], it is deeply entrenched in systemic technical barriers that permeate the entire experimental workflow (Table 2), spanning sample preparation, data acquisition, and bioinformatic assembly.

Table 2 Comparison of short- and long-read sequencing platforms

	SRS		LRS		
	Illumina (NovaSeq X)	MGI (DNBSEQ-T20)	PacBio (Revio)	ONT (PromethION 24)	MGI （CycloneSEQ-G400-ER）
DNA input requirements	100 ng	200 ng	500 ng	1 μg	500 ng

Read Length	2×150 bp	2×150 bp	15–20 kb	20 bp - >4 Mb	100 bp - >1Mb
Raw accuracy	>99.9%	>99.9%	~99.9%	~99%	~97%
Throughput	2 × (8-10.5)Tb	6 × 12 Tb	4 × (100-120)Gb	24 × (100-200)Gb	1 × 400 Gb
Runtime	48 h	24~30 h	24 h	72 h	72 h

5.1 Sample preparation constraints in long-read sequencing

By preserving native nucleic acid information, LRS fundamentally circumvents the sequence biases and errors introduced by PCR amplification in SRS[103], marking a significant leap in genomic research. However, while LRS drastically increases read lengths (Table 2), it simultaneously imposes exceedingly stringent requirements on upstream sample preparation[25, 104]. This exacting demand for high purity and intact HMW DNA is a primary technical barrier curtailing the accessibility of LRS for complex environmental niches.

First, LRS exhibits extreme sensitivity to co-purified impurities. In traditional SRS library construction, the PCR amplification process inherently dilutes easily co-precipitated inhibitors, such as polysaccharides and bile acids in gut samples, or residual extraction reagents[105]. Conversely, the amplification-free nature of LRS allows these contaminants to persist within the final library. These impurities directly inhibit single-molecule polymerase activity or irreversibly obstruct nanopores, leading to premature termination of the sequencing reaction[104, 105]. This issue is

particularly acute in complex environmental samples such as soil, where abundant humic acids, sharing high chemical structural similarity with nucleic acids, are readily co-purified. Attempts to remove these impurities through additional purification steps frequently result in irreversible fragmentation of the DNA molecules[106]. Second, the extraction of HMW DNA confronts a fundamental paradox. Even routine extraction procedures, including pipette aspiration, column purification, or mild vortexing, generate sufficient physical shear forces to induce the breakage of long DNA fragments[107, 108]. Within microbiome research, this challenge is substantially amplified. Due to the robust peptidoglycan architecture characteristic of many bacteria, gentle enzymatic lysis protocols designed to preserve the DNA backbone fail to efficiently lyse Gram-positive bacteria, resulting in severe bias and loss of taxonomic representation within the community[109]. Conversely, adopting bead-beating methods to ensure comprehensive and unbiased lysis inevitably leads to highly fragmented DNA extracts, significantly curtailing the long-read advantages of LRS[105].

Finally, low-biomass samples further exacerbate this predicament. For samples characterized by extremely low microbial abundance, the yield from conventional extraction methods struggles to meet the input requirements of LRS platforms (Table 2)[62, 110]. As explicitly demonstrated by Gweon et al. in skin microbiomes and George et al. in complex environmental soils, standard commercial kits often fail to balance yield and integrity in these niches, recovering only trace amounts of highly fragmented DNA[111, 112]. Determining how to secure adequate extraction recovery

rates from extremely low initial biomass while simultaneously preserving DNA length and purity represents a critical technical blind spot that urgently requires resolution within the field.

5.2 Inherent limitationsof data throughput and base-calling accuracy

Beyond stringent sample preparation prerequisites, the inherent limitations of LRS regarding data yield and sequencing depth also severely restrict its application. Compared to the terabase-scale single run throughput characteristic of SRS platforms, current LRS exhibit a pronounced disadvantage in data output per unit cost. For instance, the maximum throughput per flow cell for PacBio and ONT platforms is approximately 100 to 200 Gb, whereas Illumina systems can readily exceed 8 Tb (Table 2). This upper limit on throughput causes insufficient resolution for complex samples, precipitating a cascade of analytical challenges.

First, this throughput deficit amplifies the masking effect exerted by highly abundant species over low abundance taxa in the microbial communities. In complex habitats, a vast majority of sequencing reads are monopolized by a few dominant microbes. Consequently, due to insufficient overall depth, the probability of capturing species with relative abundances below 1% decreases exponentially[113]. Second, insufficient depth impedes the assembly of uncultured microorganisms and exacerbates information loss. For uncultured taxa lacking reference genomes, the inability to achieve the target coverage requisite for *de novo* assembly precludes the generation of high-quality closed genomes or extensive contiguous sequences. Consequently, a massive proportion of the sequencing data remains highly fragmented,

exhibiting extremely low completeness scores upon evaluation with algorithms such as CheckM2, and is ultimately discarded as invalid data during the binning phase[114].

Furthermore, the persistent systematic errors intrinsic to long-read technologies within homopolymer regions significantly exacerbate downstream analytical challenges. Constrained by resolution limits of nanopore electrical signals or the kinetic characteristics of single-molecule polymerases, long read platforms are highly susceptible to insertion or deletion errors across consecutive identical bases[104]. Notably, these specific inaccuracies account for over 35% of the total sequencing error profile[115]. In metagenomic samples characterized by low sequencing depth and lacking supplemental error correction from SRS data, these non random errors induce pervasive frameshift mutations within protein coding regions[116, 117]. Such structural disruptions severely impede accurate prediction of open reading frames and subsequent functional gene annotation.

5.3 Algorithmic burdens in long-read sequencing

The convergence of underlying algorithmic architectures and intrinsic sequencing error rates imposes substantial computational demands during the genomic assembly phase, particularly for complex metagenomes. Unlike the De Bruijn Graph algorithms prevalent in SRS, long-read assembly primarily relies on the Overlap Layout Consensus (OLC) framework. Although OLC demonstrates a higher tolerance for sequencing inaccuracies[118], establishing topological relationships between long reads necessitates exhaustive pairwise all-vs-all alignments. This

alignment mechanism significantly elevates computational overhead; for instance, during the assembly of the HG002 human genome, the alignment stage accounted for approximately 79% of the total execution time[119, 120].

Furthermore, when processing exceptionally intricate metagenomic samples, contemporary assembly tools struggle to circumvent sophisticated structural defects despite the allocation of massive computational resources. Evaluations indicate that prominent long-read metagenomic assemblers, including HiCanu, metaFlye, hifiasm-meta and metaMDBG[121-124], generate an average of over 40 errors per 100 megabases of contiguous sequences. These inaccuracies transcend simple base level substitutions and manifest as complex structural anomalies, such as chimeras, and prematurely circularized sequences[125].

6. Hybrid strategies and future perspectives

Hybrid sequencing remains the most practical strategy for long-read-based microbial genomics in both isolate and metagenomic sequencing (Figure 4). In isolate genomics, long reads now resolve chromosome structure, plasmids, inversions, and other repeat-rich regions with high continuity. Short reads are still often retained when coding-sequence integrity, consensus accuracy, and conservative variant interpretation are critical, especially in error-prone loci[108, 126-128]. In metagenomics, long reads preserve the genomic context needed to recover circular genomes, host-element linkages, and strain-resolved structures. Meanwhile, short reads still improve recovery in low-coverage regions and help capture rare community members[61, 129, 130]. Hybrid designs are therefore likely to remain important in the near term, even as

long-read-only workflows become increasingly reliable in selected settings[63, 127].

What is changing most rapidly now is the accuracy. PacBio HiFi sequencing showed that long reads can reach a level of accuracy suitable for both assembly and variant analysis[104]. Subsequent microbial studies showed that complete or near-complete genomes can often be recovered from isolates and microbiomes without mandatory short-read correction[63, 123, 127]. On the ONT side, R10.4.1 chemistry and improved basecalling have narrowed the historical gap in bacterial genome reconstruction, especially at sufficient coverage[128]. The field is therefore moving toward Q30-class long reads as a realistic standard in optimized workflows. Based on this, Q40-grade reads are also becoming plausible[108]. On the other hand, ultra-long reads matter not simply because they are longer, but because they can directly span the regions that still create ambiguity in microbial assembly, such as large repeats, long mobile elements, and extended recombination tracts[131, 132]. Once such regions are bridged by single molecules, some assembly problems no longer need to be solved indirectly through graph inference. Even so, this will depend on more than sequencing chemistry. It will also require reliable extraction of HMW DNA from difficult samples and further reduction of systematic error.

At the same time, LRS is now limited as much by interpretation as by data generation. This is especially clear in metagenomics, where assembly still has to accommodate extreme abundance variation, microdiversity, shared repeats, and very large datasets. Recent assemblers such as metaMDBG, nanoMDBG, and myloasm show that algorithmic redesign can still improve memory efficiency, runtime, and

recovery of circular MAGs, plasmids, and viruses[124, 133, 134]. Binning is evolving in the same way. LorBin and recent cross-platform benchmarks indicate that long-read-aware and hybrid-aware strategies can substantially improve the recovery of high-quality genomes from complex communities[135, 136]. However, continuity does not guarantee correctness. Long-read metagenome assemblies can still contain chimeras, false duplications, haplotyping artefacts, and premature circularization[125]. This point matters because future tools will need to not only produce longer contigs. but also expose uncertainty, support automated validation, and better distinguish true biological microdiversity from assembly artefact. AI is likely to become increasingly relevant in this domain. Deep learning already underlies modern nanopore basecalling, and recent studies show that model design can strongly affect both accuracy and computational cost[137, 138]. Similar approaches are likely to influence methylation calling, assembly validation, binning, and cross-omic integration. If LRS is to become routine rather than specialist, these analytical advances will also need to be matched by lower cost, more standardized workflows, and greater automation[139].

The broader significance of LRS may become clearest in multi-omics studies (Figure 4). Its main strength is that it preserves molecular context. That feature can connect genome structure with DNA methylation, transcription, and, indirectly, protein and metabolite profiles. In bacteria, long-read transcriptomics has already shown that transcript organization is more complex than suggested by fragment-based RNA sequencing, revealing extended operons and substantial transcriptional heterogeneity[87]. More recently, nanopore direct RNA sequencing has begun to

support joint analysis of the bacterial transcriptome and epitranscriptome within the same experiment[140]. LRS is also becoming increasingly important for epigenomic profiling and transcript-level analysis more broadly[91, 141]. The real opportunity for microbiology lies in combining these measurements with genome-resolved metagenomics, metatranscriptomics, metaproteomics, and metabolomics. Standardized multi-omics studies have already shown that linked analysis of genomes, functions, and metabolites can reveal ecological structure that is invisible to any single modality[142, 143]. In this setting, LRS could serve as the scaffold that anchors different measurements to the same genome or strain. That would make it easier to ask which lineages are active, which elements are mobilized, which methylation states accompany regulatory change, and how these properties shift over time or under perturbation. In the longer term, such a framework may support models that monitor strain turnover, improve prediction of engraftment or instability, and provide earlier warning of resistance emergence or community collapse. For this reason, the future value of LRS is unlikely to be defined by assembly alone. Its deeper contribution may be to help microbial genomics move from increasingly complete description toward a more mechanistic and predictive framework.

Figure 4 Future perspectives of long-read microbial genomics. (a) Technological and algorithmic evolution. Hybrid sequencing strategies and emerging ultra-long sequencing approaches (>100 kb) provide comprehensive genomic resolution for both microbial isolates and complex metagenomes. Concurrently, continuous

advancements in sequencing platforms are systematically driving raw accuracy towards Q30 and Q40 standards. To efficiently interpret these high-fidelity datasets, AI and deep learning algorithms are optimizing basecalling accuracy, enhancing binning performance, automating assembly validation, and reducing operational costs. (b) Towards multi-omics integration. LRS empowers applications across the epigenome, transcriptome, mobilome, proteome, and metabolome. This holistic integration drives a complete descriptions of genomic architectures, a mechanistic understanding of ecological interplay, and predictive models for microbial community dynamics.

Alt text: A two-panel conceptual diagram illustrating the future of long-read microbial genomics. Panel (a) shows hybrid and ultra-long sequencing technologies, rising raw accuracy, and AI-driven data optimization for basecalling, binning, and validation. Panel (b) depicts the integration of multiple omics layers to drive complete genomic descriptions, mechanistic understanding, and predictive models for microbial communities.

7. Conclusion

This review provides a systematic evaluation of the application potential and systemic barriers of LRS within microbial genomics. As a paradigm shift, LRS circumvents the inherent limitations of sequence fragmentation and amplification bias, driving a transition from simplistic taxonomic classification toward high fidelity

reconstruction of multi-dimensional ecological networks. However, its comprehensive translation across intricate systems remains constrained by multiple technical impediments. Currently, hybrid strategies integrating the structural frameworks of LRS with SRS data represent the primary methodology for balancing cost effectiveness and analytical resolution. Looking forward, the iterative refinement of lightweight bioinformatic strategies and breakthroughs in ultra-long read chemical systems are poised to definitively overcome assembly constraints. These advancements will establish a foundational framework for decoding global microbial diversity and mapping the physical architecture of microbial ecosystems.

Data Availability

Not applicable

List of abbreviations

AMR: antimicrobial resistance; ARG: antibiotic resistance gene; cDNA: complementary DNA; cMAGs: circular metagenome-assembled genomes; DNBSEQ: DNA nanoball sequencing; FMT: fecal microbiota transplantation; GC: guanine–cytosine; HGT: horizontal gene transfer; HMW DNA: high molecular weight DNA; ICE: integrative and conjugative elements; IS: insertion sequence; KPC: klebsiella pneumoniae carbapenemase; LRS: long-read sequencing; MAG: metagenome-assembled genome; MGE: mobile genetic element; NGS: next-generation sequencing; OLC: overlap layout consensus; ONT: Oxford Nanopore

Technologies; RM: restriction-modification; rRNA: ribosomal RNA; SRS: short-read sequencing; SV: structural variant; TSS: transcription start site; TTS: transcription termination site; WGS: whole-genome sequencing.

Competing interests

The authors declare that they have no competing interests.

Funding

This work was supported by the financial support from Shenzhen Science and Technology Program (No. KCXFZ20240903094006009) and Shenzhen Medical Research Fund (No. JCYJ20241202124801003).

Authors' contributions

Xing Rao (Investigation, Writing—original draft, Visualization, Writing—review & editing), Yuzheng Gu (Investigation, Writing—original draft, Visualization, Writing—review & editing), Gabriella (Writing—original draft), Jiayi Ma (Writing—original draft), Haoyu Wang (Investigation, Visualization), and Yuanqiang Zou (Conceptualization, Funding acquisition, Project administration, Supervision, Writing – review & editing).

Acknowledgements

This study is supported by grants from Shenzhen Science and Technology

Program (No.SYSPG20241211173845014).

Disclosure of use of AI-assisted tools including generative AI

During the preparation of this work, the authors used Gemini 3.1 Pro to improve the language and readability of the manuscript[144].

Reference

1. Sanger F, Nicklen S, Coulson AR. DNA sequencing with chain-terminating inhibitors. *Proc Natl Acad Sci U S A*. 1977; doi: 10.1073/pnas.74.12.5463.
2. Fleischmann RD, Adams MD, White O, et al. Whole-genome random sequencing and assembly of Haemophilus influenzae Rd. *Science*. 1995; doi: 10.1126/science.7542800.
3. van Dijk EL, Auger H, Jaszczyszyn Y, et al. Ten years of next-generation sequencing technology. *Trends Genet*. 2014; doi: 10.1016/j.tig.2014.07.001.
4. Mak SST, Gopalakrishnan S, Caroe C, et al. Comparative performance of the BGISEQ-500 vs Illumina HiSeq2500 sequencing platforms for palaeogenomic sequencing. *Gigascience*. 2017; doi: 10.1093/gigascience/gix049.
5. Previte MJ, Zhou C, Kellinger M, et al. DNA sequencing using polymerase substrate-binding kinetics. *Nat Commun*. 2015; doi: 10.1038/ncomms6936.
6. Human Microbiome Project C. A framework for human microbiome research. *Nature*. 2012; doi: 10.1038/nature11209.
7. Qin J, Li R, Raes J, et al. A human gut microbial gene catalogue established by

metagenomic sequencing. *Nature*. 2010; doi: 10.1038/nature08821.

8. Mandelker D, Schmidt RJ, Ankala A, et al. Navigating highly homologous genes in a molecular diagnostic setting: a resource for clinical next-generation sequencing. *Genet Med*. 2016; doi: 10.1038/gim.2016.58.

9. Korlach J, Bjornson KP, Chaudhuri BP, et al. Real-time DNA sequencing from single polymerase molecules. *Methods Enzymol*. 2010; doi: 10.1016/S0076-6879(10)72001-2.

10. Shafin K, Pesout T, Lorig-Roach R, et al. Nanopore sequencing and the Shasta toolkit enable efficient de novo assembly of eleven human genomes. *Nat Biotechnol*. 2020; doi: 10.1038/s41587-020-0503-6.

11. Liang H, Zou Y, Wang M, et al. Efficiently constructing complete genomes with CycloneSEQ to fill gaps in bacterial draft assemblies. *GigaByte*. 2025; doi: 10.46471/gigabyte.154.

12. Quick J, Loman NJ, Duraffour S, et al. Real-time, portable genome sequencing for Ebola surveillance. *Nature*. 2016; doi: 10.1038/nature16996.

13. Loman NJ, Quick J, Simpson JT. A complete bacterial genome assembled de novo using only nanopore sequencing data. *Nat Methods*. 2015; doi: 10.1038/nmeth.3444.

14. Quick J, Ashton P, Calus S, et al. Rapid draft sequencing and real-time nanopore sequencing in a hospital outbreak of Salmonella. *Genome Biol*. 2015; doi: 10.1186/s13059-015-0677-2.

15. Mrazek J, Guo X, Shah A. Simple sequence repeats in prokaryotic genomes.

Proc Natl Acad Sci U S A. 2007; doi: 10.1073/pnas.0702412104.

16. Brazda V, Fojta M, Bowater RP. Structures and stability of simple DNA repeats from bacteria. *Biochem J*. 2020; doi: 10.1042/BCJ20190703.

17. Schmid M, Frei D, Patrignani A, et al. Pushing the limits of de novo genome assembly for complex prokaryotic genomes harboring very long, near identical repeats. *Nucleic Acids Res*. 2018; doi: 10.1093/nar/gky726.

18. Achaz G, Rocha EP, Netter P, et al. Origin and fate of repeats in bacteria. *Nucleic Acids Res*. 2002; doi: 10.1093/nar/gkf391.

19. Hill CW. Large genomic sequence repetitions in bacteria: lessons from rRNA operons and Rhs elements. *Res Microbiol*. 1999; doi: 10.1016/s0923-2508(99)00125-4.

20. Huisman JS, Vaughan TG, Egli A, et al. The effect of sequencing and assembly on the inference of horizontal gene transfer on chromosomal and plasmid phylogenies. *Philos Trans R Soc Lond B Biol Sci*. 2022; doi: 10.1098/rstb.2021.0245.

21. George S, Pankhurst L, Hubbard A, et al. Resolving plasmid structures in Enterobacteriaceae using the MinION nanopore sequencer: assessment of MinION and MinION/Illumina hybrid data assembly approaches. *Microb Genom*. 2017; doi: 10.1099/mgen.0.000118.

22. Goldstein S, Beka L, Graf J, et al. Evaluation of strategies for the assembly of diverse bacterial genomes using MinION long-read sequencing. *BMC Genomics*. 2019; doi: 10.1186/s12864-018-5381-7.

23. Tilak MK, Botero-Castro F, Galtier N, et al. Illumina Library Preparation for Sequencing the GC-Rich Fraction of Heterogeneous Genomic DNA. *Genome Biol Evol*. 2018; doi: 10.1093/gbe/evy022.

24. International Chicken Genome Sequencing C. Sequence and comparative analysis of the chicken genome provide unique perspectives on vertebrate evolution. *Nature*. 2004; doi: 10.1038/nature03154.

25. Logsdon GA, Vollger MR, Eichler EE. Long-read human genome sequencing and its applications. *Nat Rev Genet*. 2020; doi: 10.1038/s41576-020-0236-x.

26. Wang Y, Zhao Y, Bollas A, et al. Nanopore sequencing technology, bioinformatics and applications. *Nat Biotechnol*. 2021; doi: 10.1038/s41587-021-01108-x.

27. Marx V. Method of the year: long-read sequencing. *Nat Methods*. 2023; doi: 10.1038/s41592-022-01730-w.

28. Fraser CM, Eisen JA, Nelson KE, et al. The value of complete microbial genome sequencing (you get what you pay for). *J Bacteriol*. 2002; doi: 10.1128/JB.184.23.6403-6405.2002.

29. Larsen MV, Cosentino S, Rasmussen S, et al. Multilocus sequence typing of total-genome-sequenced bacteria. *J Clin Microbiol*. 2012; doi: 10.1128/JCM.06094-11.

30. Shi W, Sun Q, Fan G, et al. gcType: a high-quality type strain genome database for microbial phylogenetic and functional research. *Nucleic Acids Res*. 2021; doi: 10.1093/nar/gkaa957.

31. Eisenhofer R, Odriozola I, Alberdi A. Impact of microbial genome completeness on metagenomic functional inference. *ISME Commun*. 2023; doi: 10.1038/s43705-023-00221-z.

32. Zou Y, Xue W, Luo G, et al. 1,520 reference genomes from cultivated human gut bacteria enable functional microbiome analyses. *Nat Biotechnol*. 2019; doi: 10.1038/s41587-018-0008-8.

33. Akintunde O, Tucker T, Carabetta VJ. The Evolution of Next-Generation Sequencing Technologies. *Methods Mol Biol*. 2025; doi: 10.1007/978-1-0716-4192-7_1.

34. Peng K, Yin Y, Li Y, et al. QitanTech Nanopore Long-Read Sequencing Enables Rapid Resolution of Complete Genomes of Multi-Drug Resistant Pathogens. *Front Microbiol*. 2022; doi: 10.3389/fmicb.2022.778659.

35. Chin FY, Leung HC, Yiu SM. Sequence assembly using next generation sequencing data--challenges and solutions. *Sci China Life Sci*. 2014; doi: 10.1007/s11427-014-4752-9.

36. Utturkar SM, Klingeman DM, Hurt RA, Jr., et al. A Case Study into Microbial Genome Assembly Gap Sequences and Finishing Strategies. *Front Microbiol*. 2017; doi: 10.3389/fmicb.2017.01272.

37. Kim C, Pongpanich M, Porntaveetus T. Unraveling metagenomics through long-read sequencing: a comprehensive review. *J Transl Med*. 2024; doi: 10.1186/s12967-024-04917-1.

38. Athanasopoulou K, Boti MA, Adamopoulos PG, et al. Third-Generation

Sequencing: The Spearhead towards the Radical Transformation of Modern Genomics. *Life (Basel)*. 2021; doi: 10.3390/life12010030.

39. Chin CS, Alexander DH, Marks P, et al. Nonhybrid, finished microbial genome assemblies from long-read SMRT sequencing data. *Nat Methods*. 2013; doi: 10.1038/nmeth.2474.

40. Koren S, Harhay GP, Smith TP, et al. Reducing assembly complexity of microbial genomes with single-molecule sequencing. *Genome Biol*. 2013; doi: 10.1186/gb-2013-14-9-r101.

41. Stanton NCKHJ-AL. Next-generation DNA sequencing of oral microbes at the Sir John Walsh Research Institute: technologies, tools and achievements. *Journal of the Royal Society of New Zealand*. 2019; doi: 10.1080/03036758.2019.1687530.

42. Wang H, Gu Y, He W, et al. Complete Genomes of Cultivated Gut Bacteria Reveal Mobile Genetic Element-Driven Functional Diversity with Therapeutic Implications. *bioRxiv*. 2026; doi: 10.64898/2026.03.25.714112.

43. Gu Y, Wang H, Yang J, et al. Complete genome-derived metabolic interactions reveal impact of gut ecology on human health. *bioRxiv*. 2026; doi: 10.64898/2026.03.25.714329.

44. Olsen NS, Riber L. Metagenomics as a Transformative Tool for Antibiotic Resistance Surveillance: Highlighting the Impact of Mobile Genetic Elements with a Focus on the Complex Role of Phages. *Antibiotics (Basel)*. 2025; doi: 10.3390/antibiotics14030296.

45. Arredondo-Alonso S, Willems RJ, van Schaik W, et al. On the (im)possibility of reconstructing plasmids from whole-genome short-read sequencing data. *Microb Genom*. 2017; doi: 10.1099/mgen.0.000128.

46. Carr VR, Shkoporov A, Hill C, et al. Probing the Mobilome: Discoveries in the Dynamic Microbiome. *Trends Microbiol*. 2021; doi: 10.1016/j.tim.2020.05.003.

47. Martinez JL, Coque TM, Lanza VF, et al. Genomic and metagenomic technologies to explore the antibiotic resistance mobilome. *Ann N Y Acad Sci*. 2017; doi: 10.1111/nyas.13282.

48. Li C, Tyson GH, Hsu CH, et al. Long-Read Sequencing Reveals Evolution and Acquisition of Antimicrobial Resistance and Virulence Genes in Salmonella enterica. *Front Microbiol*. 2021; doi: 10.3389/fmicb.2021.777817.

49. Jiang Y, Wang Y, Hua X, et al. Pooled Plasmid Sequencing Reveals the Relationship Between Mobile Genetic Elements and Antimicrobial Resistance Genes in Clinically Isolated Klebsiella pneumoniae. *Genomics Proteomics Bioinformatics*. 2020; doi: 10.1016/j.gpb.2020.12.002.

50. Zhao W, Zeng W, Pang B, et al. Oxford nanopore long-read sequencing enables the generation of complete bacterial and plasmid genomes without short-read sequencing. *Front Microbiol*. 2023; doi: 10.3389/fmicb.2023.1179966.

51. West PT, Chanin RB, Bhatt AS. From genome structure to function: insights into structural variation in microbiology. *Curr Opin Microbiol*. 2022; doi:

10.1016/j.mib.2022.102192.

52. Canalda-Baltrons A, Silcocks M, Hall MB, et al. Genome graphs reveal the importance of structural variation in Mycobacterium tuberculosis evolution and drug resistance. *Nat Commun*. 2025; doi: 10.1038/s41467-025-65779-9.

53. Cottingham H, Judd LM, Harshegyi-Hand T, et al. Nanopore sequencing enables highly accurate genotyping and identification of resistance determinants in key nosocomial pathogens. *Microb Genom*. 2026; doi: 10.1099/mgen.0.001657.

54. Wick RR, Judd LM, Stinear TP, et al. Are reads required? High-precision variant calling from bacterial genome assemblies. *Access Microbiol*. 2025; doi: 10.1099/acmi.0.001025.v3.

55. Waters EV, Tucker LA, Ahmed JK, et al. Impact of Salmonella genome rearrangement on gene expression. *Evol Lett*. 2022; doi: 10.1002/evl3.305.

56. Bouda E, Peng Y, Williams MM, et al. Bordetella pertussis exhibits genomic diversity within patients and laboratory culture. *mSystems*. 2025; doi: 10.1128/msystems.01717-24.

57. Sheppard AE, Stoesser N, Wilson DJ, et al. Nested Russian Doll-Like Genetic Mobility Drives Rapid Dissemination of the Carbapenem Resistance Gene blaKPC. *Antimicrob Agents Chemother*. 2016; doi: 10.1128/AAC.00464-16.

58. Damas MSF, Ferreira RL, Campanini EB, et al. Whole genome sequencing of the multidrug-resistant Chryseobacterium indologenes isolated from a patient in Brazil. *Front Med (Lausanne)*. 2022; doi: 10.3389/fmed.2022.931379.

59. Benler S, Faure G, Altae-Tran H, et al. Cargo Genes of Tn7-Like Transposons Comprise an Enormous Diversity of Defense Systems, Mobile Genetic Elements, and Antibiotic Resistance Genes. *mBio*. 2021; doi: 10.1128/mBio.02938-21.

60. Han B, Feng C, Jiang Y, et al. Mobile genetic elements encoding antibiotic resistance genes and virulence genes in Klebsiella pneumoniae: important pathways for the acquisition of virulence and resistance. *Front Microbiol*. 2025; doi: 10.3389/fmicb.2025.1529157.

61. Brown CL, Keenum IM, Dai D, et al. Critical evaluation of short, long, and hybrid assembly for contextual analysis of antibiotic resistance genes in complex environmental metagenomes. *Sci Rep*. 2021; doi: 10.1038/s41598-021-83081-8.

62. Agustinho DP, Fu Y, Menon VK, et al. Unveiling microbial diversity: harnessing long-read sequencing technology. *Nat Methods*. 2024; doi: 10.1038/s41592-024-02262-1.

63. Kim CY, Ma J, Lee I. HiFi metagenomic sequencing enables assembly of accurate and complete genomes from human gut microbiota. *Nat Commun*. 2022; doi: 10.1038/s41467-022-34149-0.

64. Gounot JS, Chia M, Bertrand D, et al. Genome-centric analysis of short and long read metagenomes reveals uncharacterized microbiome diversity in Southeast Asians. *Nat Commun*. 2022; doi: 10.1038/s41467-022-33782-z.

65. Moss EL, Maghini DG, Bhatt AS. Complete, closed bacterial genomes from

microbiomes using nanopore sequencing. *Nat Biotechnol*. 2020; doi: 10.1038/s41587-020-0422-6.

66. Singleton CM, Petriglieri F, Kristensen JM, et al. Connecting structure to function with the recovery of over 1000 high-quality metagenome-assembled genomes from activated sludge using long-read sequencing. *Nat Commun*. 2021; doi: 10.1038/s41467-021-22203-2.

67. Sereika M, Mussig AJ, Jiang C, et al. Genome-resolved long-read sequencing expands known microbial diversity across terrestrial habitats. *Nat Microbiol*. 2025; doi: 10.1038/s41564-025-02062-z.

68. Chen L, Zhao N, Cao J, et al. Short- and long-read metagenomics expand individualized structural variations in gut microbiomes. *Nat Commun*. 2022; doi: 10.1038/s41467-022-30857-9.

69. Bickhart DM, Kolmogorov M, Tseng E, et al. Generating lineage-resolved, complete metagenome-assembled genomes from complex microbial communities. *Nat Biotechnol*. 2022; doi: 10.1038/s41587-021-01130-z.

70. Fan Y, Ni M, Aggarwala V, et al. Long-read metagenomics for strain tracking after faecal microbiota transplant. *Nat Microbiol*. 2025; doi: 10.1038/s41564-025-02164-8.

71. Minich JJ, Allsing N, Din MO, et al. Culture-independent meta-pangenomics enabled by long-read metagenomics reveals associations with pediatric undernutrition. *Cell*. 2025; doi: 10.1016/j.cell.2025.08.020.

72. Bickhart DM, Watson M, Koren S, et al. Assignment of virus and

antimicrobial resistance genes to microbial hosts in a complex microbial community by combined long-read assembly and proximity ligation. *Genome Biol*. 2019; doi: 10.1186/s13059-019-1760-x.

73. Dai D, Brown C, Burgmann H, et al. Long-read metagenomic sequencing reveals shifts in associations of antibiotic resistance genes with mobile genetic elements from sewage to activated sludge. *Microbiome*. 2022; doi: 10.1186/s40168-021-01216-5.

74. Ding J, Wang M, Xu X, et al. Highly Resolved Community Sewage Metagenomics Unveiling Landscape and Transmission Patterns of Antibiotic Resistome in Hong Kong Populations. *Adv Sci (Weinh)*. 2026; doi: 10.1002/advs.202508389.

75. Mao X, Yin X, Yang Y, et al. Longitudinal metagenomic analysis on antibiotic resistome, mobilome, and microbiome of river ecosystems in a sub-tropical metropolitan city. *Water Res*. 2025; doi: 10.1016/j.watres.2025.123102.

76. Rolbiecki D, Paukszto L, Krawczyk K, et al. Chlorine disinfection modifies the microbiome, resistome and mobilome of hospital wastewater - A nanopore long-read metagenomic approach. *J Hazard Mater*. 2023; doi: 10.1016/j.jhazmat.2023.132298.

77. Zhang Q, Fan Y, Qian X, et al. Unraveling the role of microplastics in antibiotic resistance: Insights from long-read metagenomics on ARG mobility and host dynamics. *J Hazard Mater*. 2025; doi: 10.1016/j.jhazmat.2025.137804.

78. Suzuki Y, Nishijima S, Furuta Y, et al. Long-read metagenomic exploration of extrachromosomal mobile genetic elements in the human gut. *Microbiome*. 2019; doi: 10.1186/s40168-019-0737-z.

79. Kiguchi Y, Hamamoto N, Kashima Y, et al. Giant extrachromosomal element "Inocle" potentially expands the adaptive capacity of the human oral microbiome. *Nat Commun*. 2025; doi: 10.1038/s41467-025-62406-5.

80. Wirbel J, Hickey AS, Chang D, et al. Long-read metagenomics reveals phage dynamics in the human gut microbiome. *Nature*. 2026; doi: 10.1038/s41586-025-09786-2.

81. Monzó C, Liu T, Conesa A. Transcriptomics in the era of long-read sequencing. *Nat Rev Genet*. 2025; doi: 10.1038/s41576-025-00828-z.

82. Lucas MC, Novoa EM. Long-read sequencing in the era of epigenomics and epitranscriptomics. *Nat Methods*. 2023; doi: 10.1038/s41592-022-01724-8.

83. Jakubickova M, Sabatova K, Zbudilova M, et al. MethylomeMiner: A novel tool for high-resolution analysis of bacterial methylation patterns from nanopore sequencing. *Comput Struct Biotechnol J*. 2025; doi: 10.1016/j.csbj.2025.10.047.

84. Tourancheau A, Mead EA, Zhang XS, et al. Discovering multiple types of DNA methylation from bacteria and microbiome using nanopore sequencing. *Nat Methods*. 2021; doi: 10.1038/s41592-021-01109-3.

85. Sigurpalsdottir BD, Stefansson OA, Holley G, et al. A comparison of methods for detecting DNA methylation from long-read sequencing of human genomes.

Genome Biol. 2024; doi: 10.1186/s13059-024-03207-9.

86. Nass NM, Zaher KA. From Methylomes to CRISPR Epigenetic Editing: New Paths in Antibiotic Resistance. *Pathogens*. 2025; doi: 10.3390/pathogens14121267.

87. Yan B, Boitano M, Clark TA, et al. SMRT-Cappable-seq reveals complex operon variants in bacteria. *Nat Commun*. 2018; doi: 10.1038/s41467-018-05997-6.

88. Fu Y, Timp W, Sedlazeck FJ. Computational analysis of DNA methylation from long-read sequencing. *Nat Rev Genet*. 2025; doi: 10.1038/s41576-025-00822-5.

89. De Maio N, Shaw LP, Hubbard A, et al. Comparison of long-read sequencing technologies in the hybrid assembly of complex bacterial genomes. *Microb Genom*. 2019; doi: 10.1099/mgen.0.000294.

90. Rodger G, Lipworth S, Barrett L, et al. Comparison of direct cDNA and PCR-cDNA Nanopore sequencing of RNA from Escherichia coli isolates. *Microb Genom*. 2024; doi: 10.1099/mgen.0.001296.

91. Monzo C, Liu T, Conesa A. Transcriptomics in the era of long-read sequencing. *Nat Rev Genet*. 2025; doi: 10.1038/s41576-025-00828-z.

92. Conte MI, Fuentes-Trillo A, Dominguez Conde C. Opportunities and tradeoffs in single-cell transcriptomic technologies. *Trends Genet*. 2024; doi: 10.1016/j.tig.2023.10.003.

93. Wen L, Tang F. Single-cell omics sequencing technologies: the long-read

generation. *Trends Genet*. 2026; doi: 10.1016/j.tig.2025.07.012.

94. Lim CS, Gibbon AK, Tran Nguyen AT, et al. RIBOSS detects novel translational events by combining long- and short-read transcriptome and translatome profiling. *Brief Bioinform*. 2025; doi: 10.1093/bib/bbaf164.

95. Riquelme-Barrios S, Vasquez-Camus L, Cusack SA, et al. Direct RNA sequencing of the Escherichia coli epitranscriptome uncovers alterations under heat stress. *Nucleic Acids Res*. 2025; doi: 10.1093/nar/gkaf175.

96. Dorey A, Howorka S. Nanopore DNA sequencing technologies and their applications towards single-molecule proteomics. *Nat Chem*. 2024; doi: 10.1038/s41557-023-01322-x.

97. Alfaro JA, Bohlander P, Dai M, et al. The emerging landscape of single-molecule protein sequencing technologies. *Nat Methods*. 2021; doi: 10.1038/s41592-021-01143-1.

98. Ritmejeris J, Chen X, Albada B, et al. Terminal Conjugation Enables Nanopore Sequencing of Peptides. *J Am Chem Soc*. 2026; doi: 10.1021/jacs.5c18813.

99. Motone K, Kontogiorgos-Heintz D, Wee J, et al. Multi-pass, single-molecule nanopore reading of long protein strands. *Nature*. 2024; doi: 10.1038/s41586-024-07935-7.

100. Wang J, Chen J, Pan H, et al. Nanopore-based massively parallel sensing for peptide profiling and protein identification. *Nat Commun*. 2026; doi: 10.1038/s41467-026-69628-1.

101. Kolmogorov M, Billingsley KJ, Mastoras M, et al. Scalable Nanopore sequencing of human genomes provides a comprehensive view of haplotype-resolved variation and methylation. *Nat Methods*. 2023; doi: 10.1038/s41592-023-01993-x.

102. Giordano F, Aigrain L, Quail MA, et al. De novo yeast genome assemblies from MinION, PacBio and MiSeq platforms. *Sci Rep*. 2017; doi: 10.1038/s41598-017-03996-z.

103. Kozarewa I, Ning Z, Quail MA, et al. Amplification-free Illumina sequencing-library preparation facilitates improved mapping and assembly of (G+C)-biased genomes. *Nat Methods*. 2009; doi: 10.1038/nmeth.1311.

104. Wenger AM, Peluso P, Rowell WJ, et al. Accurate circular consensus long-read sequencing improves variant detection and assembly of a human genome. *Nat Biotechnol*. 2019; doi: 10.1038/s41587-019-0217-9.

105. Maghini DG, Moss EL, Vance SE, et al. Improved high-molecular-weight DNA extraction, nanopore sequencing and metagenomic assembly from the human gut microbiome. *Nat Protoc*. 2021; doi: 10.1038/s41596-020-00424-x.

106. Gand M, Bloemen B, Vanneste K, et al. Comparison of 6 DNA extraction methods for isolation of high yield of high molecular weight DNA suitable for shotgun metagenomics Nanopore sequencing to detect bacteria. *BMC Genomics*. 2023; doi: 10.1186/s12864-023-09537-5.

107. Jain M, Koren S, Miga KH, et al. Nanopore sequencing and assembly of a human genome with ultra-long reads. *Nat Biotechnol*. 2018; doi:

10.1038/nbt.4060.

108. Wick RR, Judd LM, Holt KE. Assembling the perfect bacterial genome using Oxford Nanopore and Illumina sequencing. *PLoS Comput Biol*. 2023; doi: 10.1371/journal.pcbi.1010905.

109. Costea PI, Zeller G, Sunagawa S, et al. Towards standards for human fecal sample processing in metagenomic studies. *Nat Biotechnol*. 2017; doi: 10.1038/nbt.3960.

110. De Coster W, Weissensteiner MH, Sedlazeck FJ. Towards population-scale long-read sequencing. *Nat Rev Genet*. 2021; doi: 10.1038/s41576-021-00367-3.

111. Serghiou IR, Baker D, Evans R, et al. An efficient method for high molecular weight bacterial DNA extraction suitable for shotgun metagenomics from skin swabs. *Microb Genom*. 2023; doi: 10.1099/mgen.0.001058.

112. Child HT, Wierzbicki L, Joslin GR, et al. Comparative evaluation of soil DNA extraction kits for long read metagenomic sequencing. *Access Microbiol*. 2024; doi: 10.1099/acmi.0.000868.v3.

113. Slizovskiy IB, Oliva M, Settle JK, et al. Target-enriched long-read sequencing (TELSeq) contextualizes antimicrobial resistance genes in metagenomes. *Microbiome*. 2022; doi: 10.1186/s40168-022-01368-y.

114. Chklovski A, Parks DH, Woodcroft BJ, et al. CheckM2: a rapid, scalable and accurate tool for assessing microbial genome quality using machine learning. *Nat Methods*. 2023; doi: 10.1038/s41592-023-01940-w.

115. Weirather JL, de Cesare M, Wang Y, et al. Comprehensive comparison of Pacific Biosciences and Oxford Nanopore Technologies and their applications to transcriptome analysis. *F1000Res*. 2017; doi: 10.12688/f1000research.10571.2.

116. Watson M, Warr A. Errors in long-read assemblies can critically affect protein prediction. *Nat Biotechnol*. 2019; doi: 10.1038/s41587-018-0004-z.

117. Pop M, Salzberg SL. Bioinformatics challenges of new sequencing technology. *Trends Genet*. 2008; doi: 10.1016/j.tig.2007.12.006.

118. Koren S, Walenz BP, Berlin K, et al. Canu: scalable and accurate long-read assembly via adaptive k-mer weighting and repeat separation. *Genome Res*. 2017; doi: 10.1101/gr.215087.116.

119. Berlin K, Koren S, Chin CS, et al. Assembling large genomes with single-molecule sequencing and locality-sensitive hashing. *Nat Biotechnol*. 2015; doi: 10.1038/nbt.3238.

120. Rautiainen M, Nurk S, Walenz BP, et al. Telomere-to-telomere assembly of diploid chromosomes with Verkko. *Nat Biotechnol*. 2023; doi: 10.1038/s41587-023-01662-6.

121. Nurk S, Walenz BP, Rhie A, et al. HiCanu: accurate assembly of segmental duplications, satellites, and allelic variants from high-fidelity long reads. *Genome Res*. 2020; doi: 10.1101/gr.263566.120.

122. Kolmogorov M, Bickhart DM, Behsaz B, et al. metaFlye: scalable long-read metagenome assembly using repeat graphs. *Nat Methods*. 2020; doi:

10.1038/s41592-020-00971-x.

123. Feng X, Cheng H, Portik D, et al. Metagenome assembly of high-fidelity long reads with hifiasm-meta. *Nat Methods*. 2022; doi: 10.1038/s41592-022-01478-3.

124. Benoit G, Raguideau S, James R, et al. High-quality metagenome assembly from long accurate reads with metaMDBG. *Nat Biotechnol*. 2024; doi: 10.1038/s41587-023-01983-6.

125. Trigodet F, Sachdeva R, Banfield JF, et al. Troubleshooting common errors in assemblies of long-read metagenomes. *Nat Biotechnol*. 2026; doi: 10.1038/s41587-025-02971-8.

126. Wick RR, Judd LM, Cerdeira LT, et al. Trycycler: consensus long-read assemblies for bacterial genomes. *Genome Biol*. 2021; doi: 10.1186/s13059-021-02483-z.

127. Sereika M, Kirkegaard RH, Karst SM, et al. Oxford Nanopore R10.4 long-read sequencing enables the generation of near-finished bacterial genomes from pure cultures and metagenomes without short-read or reference polishing. *Nat Methods*. 2022; doi: 10.1038/s41592-022-01539-7.

128. Sanderson ND, Hopkins KMV, Colpus M, et al. Evaluation of the accuracy of bacterial genome reconstruction with Oxford Nanopore R10.4.1 long-read-only sequencing. *Microb Genom*. 2024; doi: 10.1099/mgen.0.001246.

129. Bertrand D, Shaw J, Kalathiyappan M, et al. Hybrid metagenomic assembly

enables high-resolution analysis of resistance determinants and mobile elements in human microbiomes. *Nat Biotechnol*. 2019; doi: 10.1038/s41587-019-0191-2.

130. Jin H, You L, Zhao F, et al. Hybrid, ultra-deep metagenomic sequencing enables genomic and functional characterization of low-abundance species in the human gut microbiome. *Gut Microbes*. 2022; doi: 10.1080/19490976.2021.2021790.

131. Jain C, Rodriguez RL, Phillippy AM, et al. High throughput ANI analysis of 90K prokaryotic genomes reveals clear species boundaries. *Nat Commun*. 2018; doi: 10.1038/s41467-018-07641-9.

132. Koren S, Bao Z, Guarracino A, et al. Gapless assembly of complete human and plant chromosomes using only nanopore sequencing. *Genome Res*. 2024; doi: 10.1101/gr.279334.124.

133. Benoit G, James R, Raguideau S, et al. High-quality metagenome assembly from nanopore reads with nanoMDBG. *Nat Commun*. 2026; doi: 10.1038/s41467-026-69760-y.

134. Shaw J, Marin MG, Li H. High-resolution metagenome assembly for modern long reads with myloasm. *Nat Biotechnol*. 2026; doi: 10.1038/s41587-026-03053-z.

135. Xue W, Liu Z, Zhang Y, et al. LorBin: efficient binning of long-read metagenomes by multiscale adaptive clustering and evaluation. *Nat Commun*. 2025; doi: 10.1038/s41467-025-64916-8.

136. Han H, Wang Z, Zhu S. Benchmarking metagenomic binning tools on real datasets across sequencing platforms and binning modes. *Nat Commun*. 2025; doi: 10.1038/s41467-025-57957-6.

137. Pages-Gallego M, de Ridder J. Comprehensive benchmark and architectural analysis of deep learning models for nanopore sequencing basecalling. *Genome Biol*. 2023; doi: 10.1186/s13059-023-02903-2.

138. Singh G, Alser M, Denolf K, et al. RUBICON: a framework for designing efficient deep learning-based genomic basecallers. *Genome Biol*. 2024; doi: 10.1186/s13059-024-03181-2.

139. Child HT, Wierzbicki L, Joslin GR, et al. Automated environmental metagenomics using Oxford nanopore sequencing. *BMC Genomics*. 2025; doi: 10.1186/s12864-025-11989-w.

140. Tan L, Guo Z, Shao Y, et al. Analysis of bacterial transcriptome and epitranscriptome using nanopore direct RNA sequencing. *Nucleic Acids Res*. 2024; doi: 10.1093/nar/gkae601.

141. Liu T, Conesa A. Profiling the epigenome using long-read sequencing. *Nat Genet*. 2025; doi: 10.1038/s41588-024-02038-5.

142. Shaffer JP, Nothias LF, Thompson LR, et al. Standardized multi-omics of Earth's microbiomes reveals microbial and metabolite diversity. *Nat Microbiol*. 2022; doi: 10.1038/s41564-022-01266-x.

143. Go D, Yeon GH, Park SJ, et al. Integration of metabolomics and other omics: from microbes to microbiome. *Appl Microbiol Biotechnol*. 2024; doi:

10.1007/s00253-024-13384-z.

144. Google (2026). Gemini (3.0 Pro) [Large language model]. Response to query made by Xing Rao, Yuzheng Gu, Gabriella, Jiayi Ma. 04/2026. https://gemini.google.com

Table 1. Studies demonstrating the implementation of LRS for single-isolate microbial genome reconstruction (2013–2026)

**Year**	**Study**	**Platformstrategy**	**Isolate(s) / dataset**	**Main contribution to genome quality**
2013	Koren et al.	PacBio RS	6 bacterial genomes + repeat analysis of 2,267 finished bacterial/archaeal genomes	Showed that long reads greatly reduce assembly complexity and suggested that more than 70% of known bacterial and archaeal genomes could be closed at finished-grade quality using a single PacBio library.
2013	Chin et al.	PacBio SMRT	*Escherichia coli* K-12 MG1655, *Meiothermus ruber* DSM1279,*Pedobacter heparinus* DSM2366	Demonstrated non-hybrid finishing from a single long-insert library, successful resolution of long repeats, and consensus accuracy exceeding 99.999%.
2015	Loman et al.	ONT MinION	*Escherichia coli* K-12 MG1655	Produced a single 4.6 Mb contig with 99.5% nucleotide identity, providing early proof that nanopore-only data could recover complete

				chromosome structure.
2017	Wick et al.	ONT MinION + Illumina (Hybrid)	12 *Klebsiella pneumoniae* isolates	Showed that multiplex MinION sequencing plus hybrid assembly could generate fully resolved assemblies with little to no manual intervention, making complete bacterial genome closure more scalable.
2017	Teng et al.	PacBio RS II using P6-C4	*Burkholderia pseudomallei*	PacBio completely resolved the high-GC, two-chromosome genome without gaps or misassemblies and with >99.9% identity, whereas Illumina assemblies remained fragmented into 201–366 contigs.
2017	George et al.	ONT MinION + Illumina (Hybrid)	8 *Enterobacteriaceae* isolates	Showedchromosomal contigs matching >95% of the reference chromosomal sequence length. Improve plasmid reconstruction where assembler recovered 70% of plasmids in complete contigs, increased to 78% with combined assembler.
2019	De Maio et al.	Illumina + ONT or SMRT PacBio (Hybrid)	20 *Enterobacteriaceae* isolates	Hybrid assembly with either ONT or PacBio increased accuracy and completeness. ONT+Illumina fully resolved most genomes and had lower consumable cost per isolate in their setting.

2019	Sydenham et al.	Illumina MiSeq + ONT MinION (Hybrid)	6 multidrug-resistant *Bacteroides fragilis* isolates	Generated complete circular assemblies of all isolates, identified 11 circular plasmids, and resolved complete insertion sequences upstream of AMR genes.
2022	Sereika et al.	ONT R10.4	Pure cultures and metagenomes	Showed that R10.4 could generate near-finished microbial genomes without short-read or reference polishing, with near-finished quality obtained at roughly 40× coverage and read accuracy of 99%.
2024	Sanderson et al.	ONT R10.4.1/kit14 flowcell/chemistry	13 bacterial isolates	Demonstrated that updated chemistry plus methylation-aware basecalling produced accurate long-read-only assemblies at ≥40× depth, bringing ONT-only bacterial reconstruction closer to routine use.
2024	Luan et al.	Oxford Nanopore GridION, Illumina MiSeq	15 *Salmonella enterica serovar*	Showed that long-read assemblies could reconstruct complete genomes at about 99.95% accuracy, while 99.9999% accuracy required combined long- and short-read polishing, highlighting the present upper boundary of “near-perfect” reconstruction.
2025	Fan et al.	DNBSEQ-T5 + CycloneSEQ-WT02	*Lactiplantibacillus*	A hybrid of SRS and LRS a complete genome assembly with 100% completeness.

		(Hybrid)	*plantarum* BGI-J9	
2025	Wang et al.	DNBSEQ-T5 + CycloneSEQ-WT02 (Hybrid)	*Lacticplantibacillus plantarum* BGI-N6	Achieved a complete genome assembly, replacing fragmented draft structure with a fully resolved genome.
2025	Wang et al.	DNBSEQ-T5 + CycloneSEQ-WT02 (Hybrid)	*Lacticaseibacillus rhamnosus* OF44	Achieved a complete genome assembly of a single-contig with 99.99% completeness and 0.19% contamination.
2026	Rao et al.	DNBSEQ-T5 + CycloneSEQ-WT02 (Hybrid)	*Lacticaseibacillus paracasei* BGI-N2	Achieved a complete single-contig genome assembly of 3,102,337 bp, with 46.4% GC content and 2,937 CDSs.

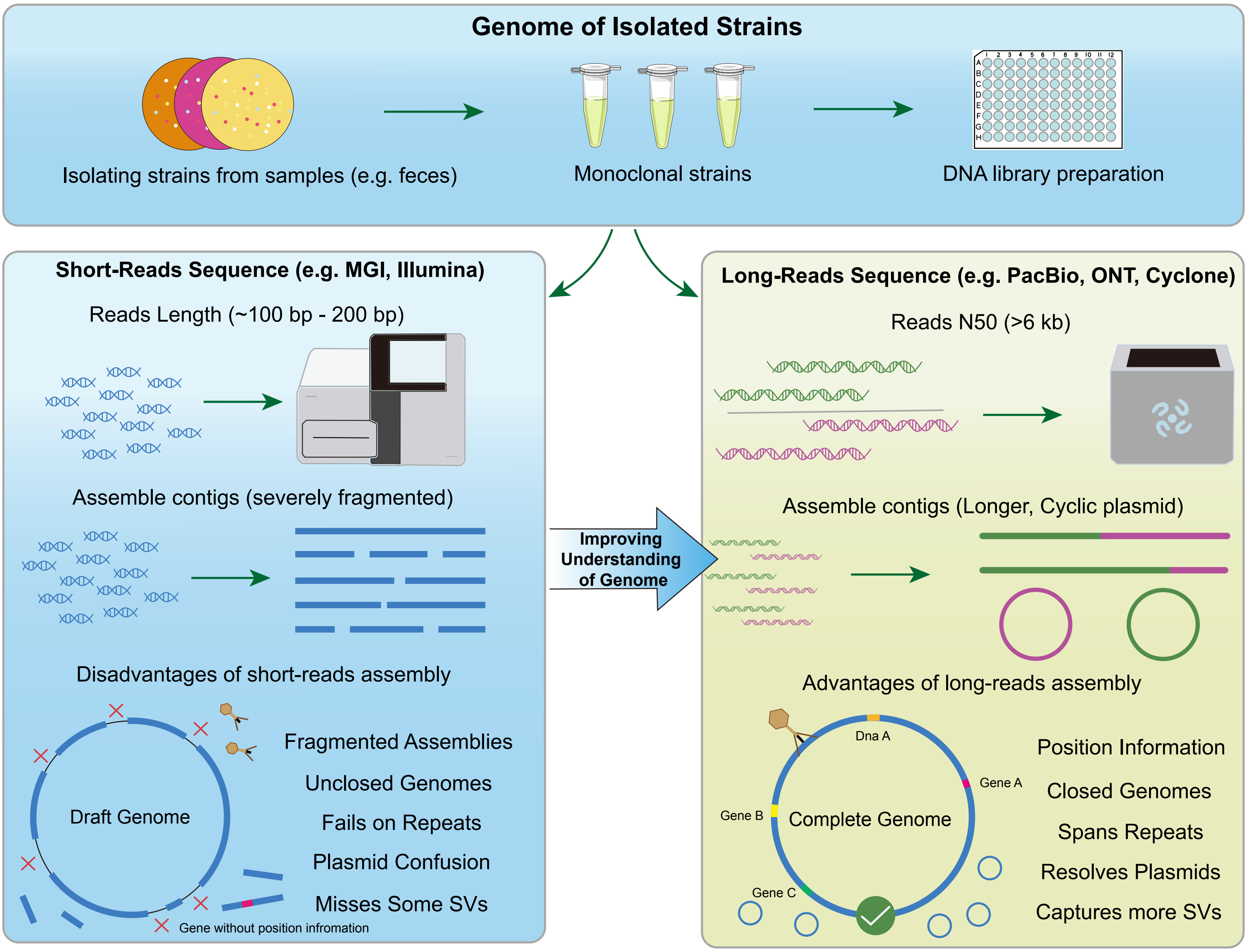
Genome of Isolated Strains
Isolating strains from samples (e.g. feces)
Monoclonal strains
DNA library preparation
Short-Reads Sequence (e.g. MGI, Illumina)
Reads Length (~100 bp - 200 bp)
Assemble contigs (severely fragmented)
Disadvantages of short-reads assembly
Draft Genome
Fragmented Assemblies
Unclosed Genomes
Fails on Repeats
Plasmid Confusion
Misses Some SVs
Gene without position infromation
Improving Understanding of Genome
Long-Reads Sequence (e.g. PacBio, ONT, Cyclone)
Reads N50 (>6 kb)
Assemble contigs (Longer, Cyclic plasmid)
Advantages of long-reads assembly
Dna A
Gene A
Gene B
Gene C
Complete Genome
Position Information
Closed Genomes
Spans Repeats
Resolves Plasmids
Captures more SVs

Figure

Click here to access/download;Figure;FIgure2.pdf

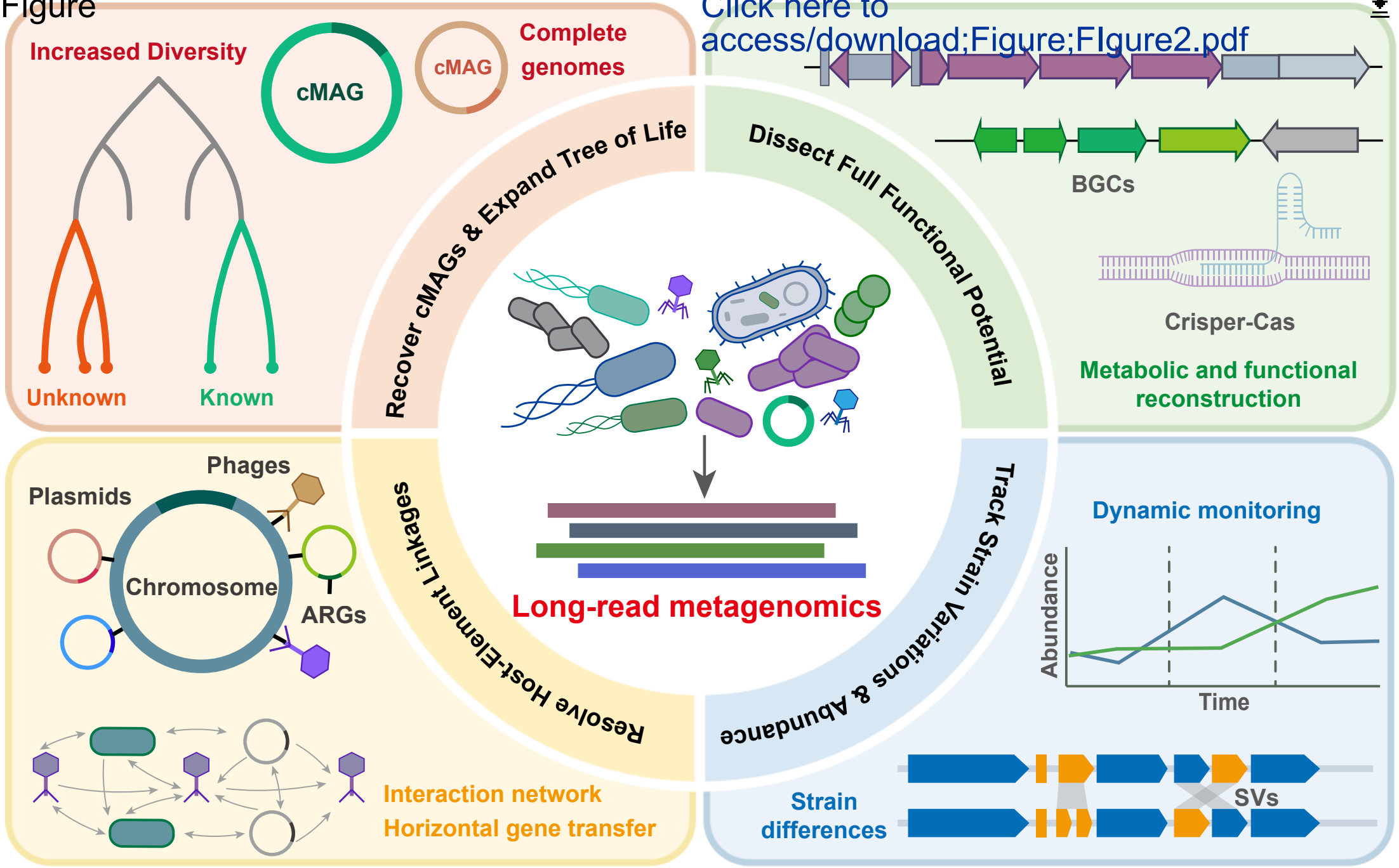

 Click here to access/download;Figure;Figure3.pdf

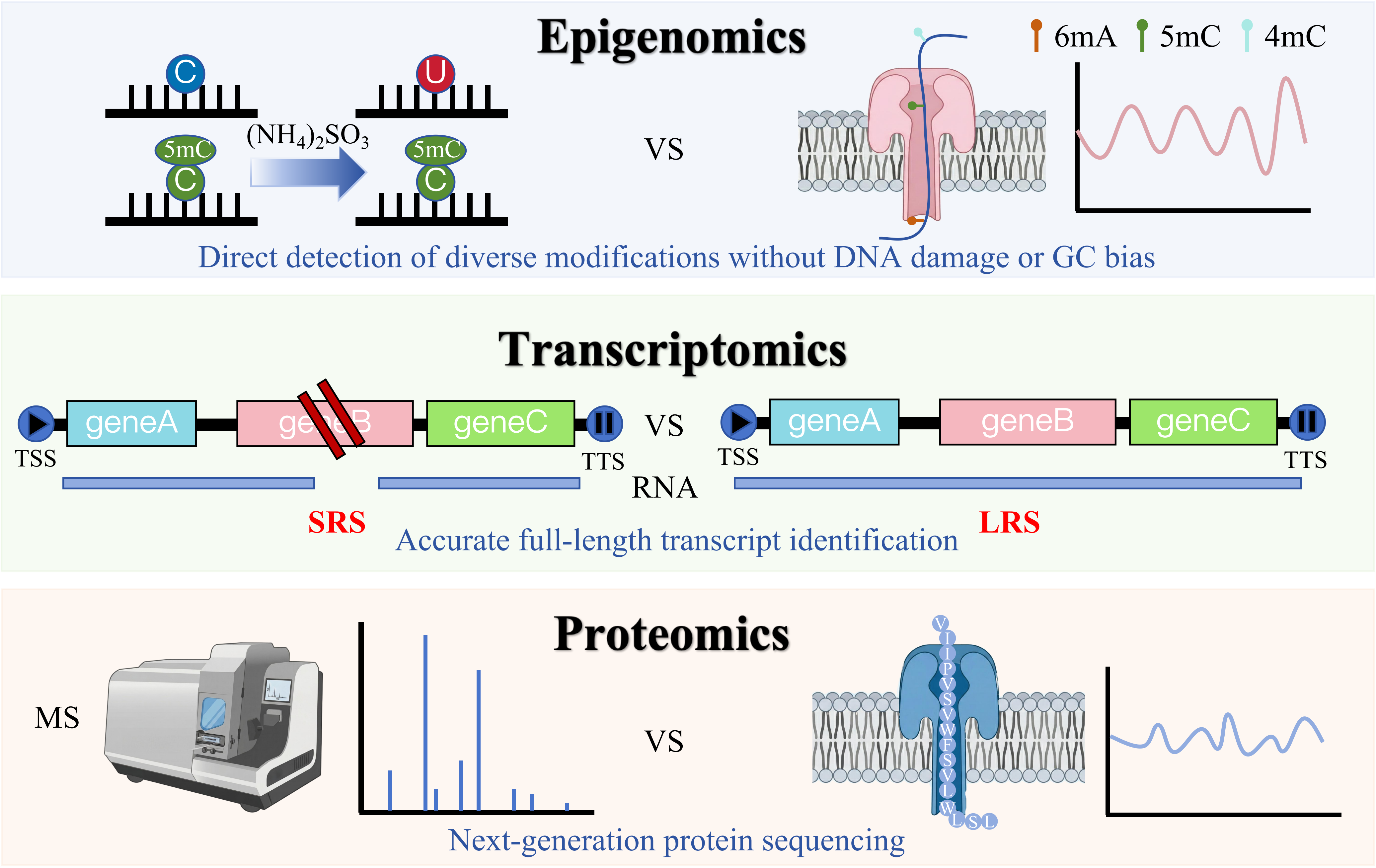

Figure Click here to access/download;Figure;FIgure4.pdf

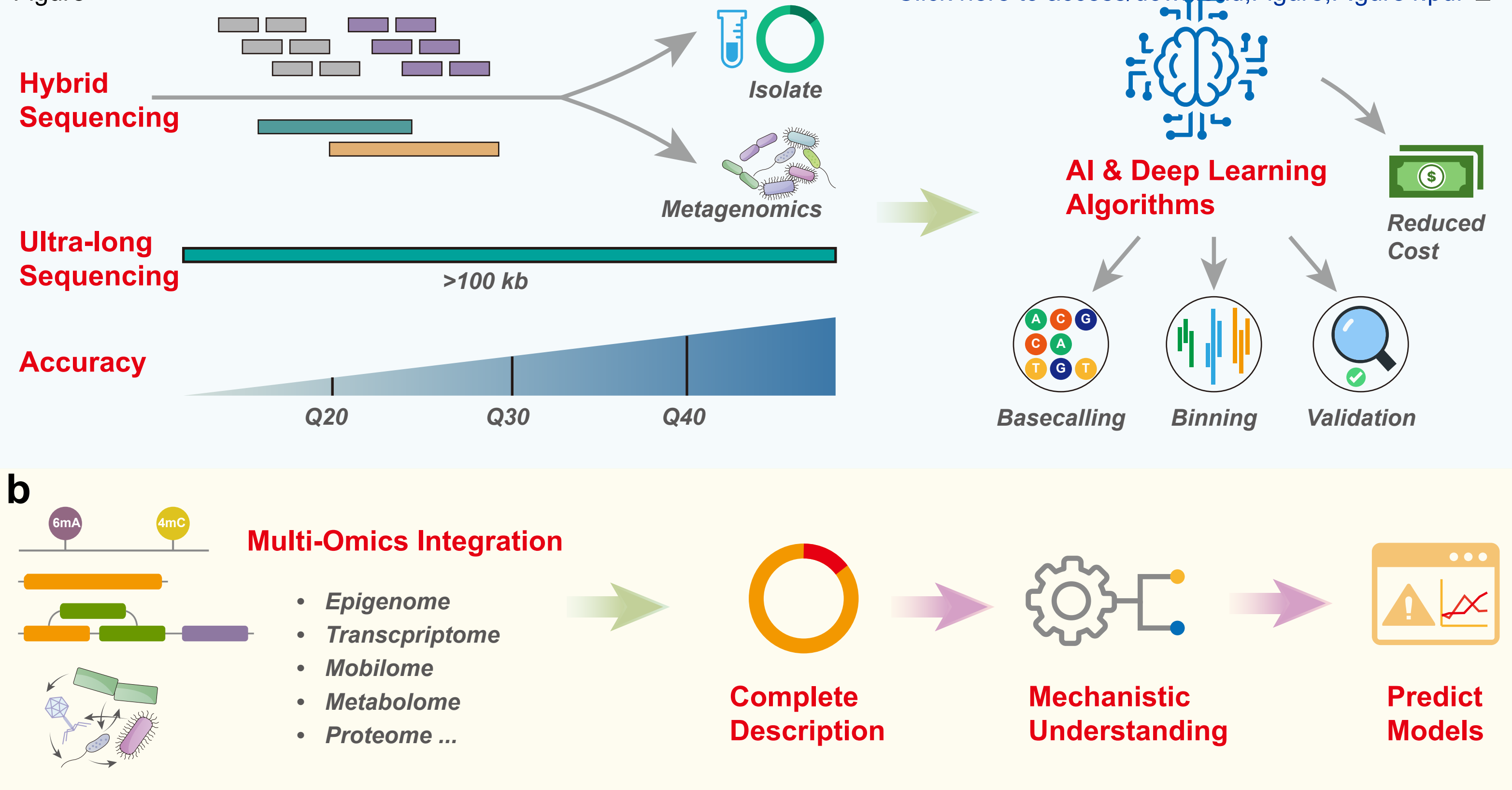

Supplement: Supplementary file 3 [file giag087_giga-d-26-00124_original_submission.pdf]
