# Supplementary material for: Promises and pitfalls of long-read sequencing for resolving microbial complexity
Source: Gigascience. 2026 Aug 17;15:giag087. doi: 10.1093/gigascience/giag087 (PMC13543314; doi:10.1093/gigascience/giag087)

GigaScience

Promises and Pitfalls of Long-Read Sequencing for Resolving Microbial Complexity

--Manuscript Draft--

**Manuscript Number:**	GIGA-D-26-00124R1
**Full Title:**	Promises and Pitfalls of Long-Read Sequencing for Resolving Microbial Complexity
**Article Type:**	Review
**Funding Information:**	Shenzhen Science and Technology Program (KCXFZ20240903094006009) — Dr. Yuanqiang Zou<br>Shenzhen Medical Research Fund (JCYJ20241202124801003) — Dr. Yuanqiang Zou
**Abstract:**	Long-Read Sequencing (LRS) has driven a transition in microbial genomics, overcoming the assembly fragmentation inherent to short-read sequencing.<br>This review elucidates the impact of LRS across isolate genomics, metagenomics, and multi-omics domains. By spanning extensive repetitive regions, LRS facilitates the reconstruction of gapless circular chromosomes and precisely resolves mobile genetic elements (MGEs). In metagenomics, LRS enables high-resolution strain parsing, the recovery of circular metagenome-assembled genomes, and the precise localization of MGEs within host replicons. Furthermore, the single-molecule, amplification-free properties of LRS provide enhanced resolution of native epigenetic modifications and full-length transcriptomes. Despite these advancements, widespread implementation remains constrained by multidimensional challenges, including stringent high-molecular-weight DNA requirements, depth deficits, and computational overhead. Nevertheless, LRS is increasingly becoming the method of choice for isolate genomics and metagenomics.<br>As detection technologies and algorithms progress, LRS will establish a contiguous, multidimensional landscape for deciphering microbial ecosystems.
**Corresponding Author:**	Yuanqiang Zou<br>BGI-Shenzhen: BGI Group<br>Shenzhen, CHINA
**Corresponding Author Secondary Information:**	
**Corresponding Author's Institution:**	BGI-Shenzhen: BGI Group
**Corresponding Author's Secondary Institution:**	
**First Author:**	xing rao
**First Author Secondary Information:**	
**Order of Authors:**	xing rao
	Yuzheng Gu
	Gabriella
	Jiayi Ma
	Haoyu Wang
	Yuanqiang Zou
**Order of Authors Secondary Information:**	
**Response to Reviewers:**	Dear editor and Reviewers,<br><br>We thank all of you for taking the time to edit or review this manuscript. We appreciate all the professional comments and suggestions, which are very helpful in improving this manuscript. We have carefully considered these critical comments and thoughtful suggestions. The point-to-point responses to the reviewers' concerns are listed below. Besides, a marked-up version of the revised manuscript with the changes has also

been submitted.

Regards,
Yuanqiang Zou, PhD

Responses to reviewers:

#Reviewer 1:

This review provides a comprehensive overview of the paradigm shift that long-read sequencing (LRS) has brought to microbial genomics and appropriately discusses its current technical limitations. A particular strength of the manuscript is the unusually rich set of case studies focused on pathogenic bacteria and antibiotic resistance genes, which the authors discuss from several complementary angles. This emphasis is, in my view, the most distinctive contribution of the review. Based on this strength, I would like to suggest the following improvements to further enhance the manuscript:
* Addition of a schematic figure that specifically illustrates how LRS has benefited pathogenic bacterial genomics would substantially help readers situate the technological gains in a translational context.
* The long-read metagenomics sections would benefit from additional content connecting LRS to pathogenic bacterial genomics. For example, the current status of real-time pathogen detection enabled by long-read metagenomics is a particularly impactful topic and would be a natural extension.
* If the authors retain the current emphasis on pathogenic bacteria throughout the manuscript, a corresponding revision of the title to more accurately reflect this scope would be warranted.

Response:
We sincerely thank the reviewer for the positive and encouraging evaluation of our work. We also greatly appreciate the constructive feedback and insightful concerns you have raised to further improve the manuscript. We have carefully considered each of your points and have made comprehensive revisions accordingly.
We are pleased that the pathogenic-bacteria and antibiotic-resistance case studies were viewed as a distinctive strength of the review. We agree that the translational implications of LRS should be made clearer, especially in the metagenomics section. In response, we have added a new subsection entitled “Pathogenic bacterial genomics and real-time detection,” focusing on culture-independent long-read metagenomics from clinical specimens. This new section discusses real-time nanopore pathogen detection, AMR-gene screening, and the possibility of partial genome recovery when pathogen load and sequencing depth are sufficient. We cite primary studies demonstrating rapid nanopore metagenomic diagnosis of lower respiratory infection, real-time pathogen detection from infected body fluids, and a recent unified host-depletion nanopore workflow for detection of bacteria, fungi and viruses from respiratory samples. The added text can be found in Lines 432-457 as “Long-read metagenomics provides a culture-independent route from clinical specimens to pathogen information, making it an important translational extension of genome-resolved microbial genomics. Its distinctive advantage in this setting is the combination of long molecular reads and real-time nanopore analysis. Reads can be classified during sequencing, allowing pathogen detection and resistance-gene screening to begin before the sequencing run is complete. In lower respiratory infection, Charalampous et al. developed a host-depleted nanopore metagenomic workflow that produced results within 6 h from sample to result, detected bacterial pathogens with 96.6% sensitivity compared with culture, and accurately detected antibiotic-resistance genes[84]. In infected body fluids, Gu et al. showed that nanopore mNGS coupled with real-time computational analysis enabled pathogen identification with a median 50 min of sequencing and a 6 h sample-to-answer time[85]. More recently, Alcolea-Medina et al. developed a unified host-depletion and nanopore workflow for respiratory samples that detected RNA and DNA viruses, bacteria, atypical bacteria and fungi, generated first automated reports after 30 min of sequencing within a 7 h workflow, and achieved prospective bacterial detection sensitivity and specificity of 90% and 100% after 2 h of sequencing[86]. This study also showed that genome recovery was feasible for a subset of reported organisms after longer sequencing, supporting the potential of clinical long-read metagenomics to move beyond detection toward strain typing and resistance or virulence analysis when pathogen load and read depth are sufficient[86].

Powered by Editorial Manager® and ProduXion Manager® from Aries Systems Corporation

These studies indicate that LRS-based metagenomics is most relevant when culture is slow, antibiotic exposure may reduce culture sensitivity, or rapid resistance information is needed. Clinical implementation nevertheless remains constrained by host-background DNA, contamination control, pathogen-load thresholds, database interpretation, genotype-phenotype concordance for AMR, and the need for prospective validation."

Furthermore, we carefully considered the suggestion to add a dedicated schematic figure and revise the title. However, because the review still covers microbial genomics broadly, including isolate genome closure, MAG/cMAG recovery, mobile genetic elements, strain variation, epigenomics, transcriptomics, and metagenomic ecology across both pathogenic and non-pathogenic contexts, we have decided not to add a pathogen-specific figure or narrow the title. We understand that the pathogenic examples in Section 2 may created a misleading impression. To correct this imbalance and better reflect our original intent, we have revised as follows. We removed the pathogenic cases of Chryseobacterium indologenes and Klebsiella pneumoniae, and added probiotic examples to explicitly demonstrate the broad microbial relevance. The added text can be found in Lines 146-150 as "Beyond genome completeness, LRS also enhanced functional recovery. For Lactiplantibacillus pentosus LP309, LRS resolved its complete chromosome and eight plasmids, enabling the prediction of over 150 probiotic functional genes involved in acid resistance and vitamin synthesis[44].", Lines 188-190 "For instance, McLeod et al. resolved the extensive plasmid complement of Lactiplantibacillus plantarum MF1298, yielding 14 complete plasmid sequences, one of which carried functional genes associated with vitamin B12 transformation[54].", and Lines 250-253 "Beyond resistance spread, complete genome resolution of the probiotic Lactobacillus crispatus precisely anchored Tn7088, a novel integrative mobile element encoding a class I bacteriocin biosynthetic gene cluster that drives antimicrobial competition and probiotic fitness[62]."

References:

[44]Marin-Gordillo A et al. Hybrid genome 942 assembly and comprehensive genomic analysis of Lactiplantibacillus pentosus 943 LP309 reveal its probiotic and technological potential. BMC Genomics. 2026; 944 .doi: 10.1186/s12864-026-12678-y.
[54]McLeod A et al. Large Plasmid Complement Resolved: Complete Genome Sequencing of Lactobacillus plantarum MF1298, a Candidate Probiotic Strain Associated with Unfavorable Effect. Microorganisms. 2019; doi: 10.3390/microorganisms7080262.
[62] Colombini L et al. The mobilome of Lactobacillus crispatus M247 includes two novel genetic elements: Tn7088 coding for a putative bacteriocin and the siphovirus prophage PhiM247. Microb Genom. 2023; doi: 10.1099/mgen.0.001150.
[84] Charalampous et al. Nanopore metagenomics enables rapid clinical diagnosis of bacterial lower respiratory infection. Nature Biotechnology. 2019. doi: 10.1038/s41587-019-0156-5.
[85] Gu et al. Rapid pathogen detection by metagenomic next-generation sequencing of infected body fluids. Nature Medicine. 2021. doi: 10.1038/s41591-020-1105-z.
[86] Alcolea-Medina et al. Unified metagenomic method for rapid detection of microorganisms in clinical samples. Communications Medicine. 2024. doi: 10.1038/s43856-024-00554-3.

Major comments

1. Section 2.3 — Claim that long reads outperform short reads in SNP detection (Lines 205-208, p.10)

The current text states that ONT long reads outperform short reads for SNP calling. Given that, on a per-base basis, short reads still have a substantially lower error rate than long reads, this conclusion may strike many readers as counter-intuitive. In all likelihood, the long-read advantage is restricted to specific genomic contexts (for example, repetitive regions and loci embedded in structural variants or mobile genetic elements) where short reads cannot be uniquely mapped. I recommend that the authors explicitly clarify the underlying reason why long reads outperform short reads at SNP calling in these specific contexts. This framing will help prevent readers from overestimating the general per-base SNP accuracy of LRS platforms.

Response:

We thank the reviewer for this important and valuable guidance. We agree that the raw per-base consensus accuracy of short reads retains a lower per-base error rate and remains highly reliable for genomic study. Our original phrasing inadvertently

overstated the generalized accuracy of long reads which overshadowed the contribution of SRS. To address this, we have explicitly mentioned at Line 196-204 “Although one of the clearest advantages of LRS lies in resolving large inversions, duplications and other structural variants, they also improve the detection and interpretation of smaller-scale variants such as single nucleotide variants (SNVs), when these variants occur within complex genomic architecture. Recent ONT-based benchmarking showed that Illumina- and k-mer-based SNP typing failed to detect 9 to 68 SNPs per 1,000 sites in repetitive reference regions. Conversely, by spanning these complex regions on one long single molecule, ONT long reads improved SNP calls across the previously inaccessible genomic regions, where SRS alignment is at disadvantage.” to clarify the advantage of LRS in SNV calling is localized to repetitive regions, duplications, and structurally variable loci and these were the result of LRS ability to produce a single molecule with enough length (10 to 100kb) that span across genomic architecture. In these specific contexts, short reads struggle with multi-mapping ambiguities due to fragmented reads were mentioned.
Accordingly, we revised words and sentence selection in Section 2 to prevent overstating and misinterpretation. Exaggerated claim in Line 199 "perfect full SNP calls" was replaced with a more nuanced language emphasizing LRS contribution in improving variant resolution in intricate regions.

2. Figure 2 / Section 3 — Why does cMAG construction expand phylogenetic diversity? (Lines 288-289, p.14)
The current sentence — "LRS facilitates the direct reconstruction of cMAGs, thereby significantly expanding the known phylogenetic diversity" — is asserted without an explicit rationale. If the sole goal were to expand phylogenetic diversity, conventional 16S rRNA gene surveys would, in principle, already suffice. I recommend that the authors clarify (and cite supporting primary literature, both in the main text and in the Figure 2 caption) the specific reasons why cMAG construction is required to expand phylogenetic diversity beyond what marker-gene approaches can achieve.
Response:
Thanks very much for this helpful comment. We agree that the original wording did not clearly explain why cMAG construction expands phylogenetic diversity beyond what marker-gene surveys can provide. Although 16S rRNA gene surveys can detect divergent lineages, they do not provide complete genome representatives, phylogenomic placement based on conserved single-copy genes, or linkage between taxonomic identity and functional elements such as metabolic pathways, BGCs, CRISPR-Cas systems and MGEs. In contrast, cMAGs enable uncultivated lineages to be represented as genomes and interpreted in both evolutionary and functional contexts. We have added supporting citations to primary literature showing that genome-resolved metagenomics expands the microbial genome tree and that long-read terrestrial metagenomics recovered >15,000 previously undescribed genomes together with complete rRNA operons, BGCs, and CRISPR-Cas systems. We have therefore revised both Section 3.1 as “More recently, deep long-read surveys across terrestrial habitats recovered genomes representing more than 15,000 previously undescribed genomes and expanded the known phylogenetic breadth of prokaryotic diversity. Meanwhile, LRS converted divergent marker-gene signals or fragmented metagenomic assemblies into high-quality genome representatives that can be placed in phylogenomic frameworks and linked to complete ribosomal RNA operons, BGCs, and CRISPR-Cas systems[69]. This indicates that long-read metagenomics contributes not only to improved assembly of known taxa and recovery of lineages that remain poorly represented in genome-centred reference frameworks, but also to help explore repetitive, structurally complex, and functionally critical elements that define microbial adaptability and evolution.” in Lines 315-325 and revised Figure 2 caption as “LRS facilitates the reconstruction of cMAGs, expanding genome-resolved phylogenetic diversity by generating complete or near-complete genome representatives that can be placed in phylogenomic frameworks and linked to functional elements” in Lines 274-278.

References:
[69] Sereika M, Mussig AJ, Jiang C, et al. Genome-resolved long-read sequencing expands known microbial diversity across terrestrial habitats. Nat Microbiol. 2025; doi: 10.1038/s41564-025-02062-z.

3. Chromosome-MGE linkage in metagenomic contexts (Lines 292-294, p.14)

I agree with the statement that long reads enable the separation of chromosomal contigs and MGE contigs in single-isolate assemblies. However, in a true metagenomic context, identifying the chromosomal host of an MGE remains difficult even with long reads alone. The current wording risks overstating the present capability. I suggest the authors add a sentence describing which complementary strategies can (at least partially) establish chromosome-MGE linkages in metagenomes with appropriate citation, such as proximity-ligation methods (Hi-C / 3C), methylation-based host assignment, and ultra-long reads that physically span the integration site between the MGE and its chromosomal flanking sequence.
Response:
Thanks the reviewer for this important clarification. We agree that the previous wording overstated host assignment as a general capability of long-read metagenomics. For integrated MGEs, including prophages, LRS can provide direct sequence evidence when reads or contigs span the element-chromosome junction. Long-read assemblies can also improve phage-host association because longer viral and bacterial contigs improve CRISPR-based matching and host taxonomic assignment. However, extrachromosomal plasmids remain more difficult to assign because they usually lack a fixed chromosomal junction, often have mosaic sequence composition, and may move across taxonomic boundaries. We therefore added complementary strategies, including Hi-C/3C proximity ligation and methylation-based binning, as orthogonal evidence for plasmid-host and broader MGE-host assignment in metagenomic datasets.
We have revised Section 3.3 to distinguish different types of MGE-host evidence as "LRS can strengthen host-MGE analysis in two distinct ways. For integrated MGEs, including prophages, reads or contigs that span the element-chromosome junction provide direct sequence evidence for the host genome[76]. In addition, longer viral and bacterial contigs can improve phage-host association by recovering more complete viral genomes, CRISPR arrays and host taxonomic signals. For example, viralFlye showed that long-read assemblies increased CRISPR-based virus-host predictions and improved host assignment precision relative to short-read assemblies[77]. Extrachromosomal plasmids are often harder to assign because they lack obligatory chromosomal junctions, have mosaic sequence composition and may circulate across multiple hosts. Therefore, plasmid-host and ARG-bearing MGE assignments in metagenomes often require orthogonal evidence, such as Hi-C/3C proximity ligation, methylation-aware binning, or ultra-long reads when they span integration or cointegration junctions[78]." in Lines 380-392.
We also have revise the Figure 2 caption as "Furthermore, LRS supports evidence-based host-MGE association by resolving integration junctions and improving phage-host inference, while complementary strategies provide evidence for assigning plasmids, phages and ARG-bearing elements to likely hosts" in Lines 281-284.

References:
[76] Yahara K, Suzuki M, Hirabayashi A, et al. Long-read metagenomics using PromethION uncovers oral bacteriophages and their interaction with host bacteria. Nat Commun. 2021; doi: 10.1038/s41467-020-20199-9.
[77] Antipov D, Raiko M, Lapidus A, Pevzner PA. viralFlye: assembling viruses and identifying their hosts from long-read metagenomics data. Genome Biology. 2022; doi: 10.1186/s13059-021-02566-x.
[76] Beaulaurier J, Zhu S, Deikus G, et al. Metagenomic binning and association of plasmids with bacterial host genomes using DNA methylation. Nat Biotechnol. 2018; doi: 10.1038/nbt.4037.

4. Section 3.2 — Mechanism of strain-resolution long-read metagenomics, and recent tooling (p.16)
The emphasis on strain resolution as a key strength of long-read metagenomics is well taken and highly valuable for the field. To strengthen this section further, please add a concise explanation of how strain resolution is actually achieved with long reads (e.g., preservation of haplotypic linkage on a single molecule, phasing of SNVs/SVs across long stretches, and traversal of structurally variable accessory loci). In addition, the recent release of myloasm (Shaw J, Marin MG, Li H. High-resolution metagenome assembly for modern long reads with myloasm. Nat Biotechnol. 2026; https://doi.org/10.1038/s41587-026-03053-z) makes strain-resolution long-read metagenomic assembly substantially more accessible in practice, and I encourage the authors to mention this development in this section.

	Response:<br>Thanks very much for this constructive suggestion. We agree that Section 3.2 should explain not only that long-read metagenomics improves strain resolution, but also how this resolution is achieved. LRS preserve haplotypic linkage on individual DNA molecules, allowing single-nucleotide variants, indels, and SV breakpoints to be phased across long genomic intervals. They also traverse structurally variable accessory loci, including prophages, BGCs, CRISPR arrays, and insertion-sequence-flanked islands, that are often fragmented or collapsed in short-read assemblies. We also added the recent myloasm study to this section, noting that modern long-read assemblers are making strain-resolved metagenome assembly more accessible by improving graph resolution in metagenomic samples.<br>We have revised the text as "Long-read metagenomics is also redefining strain resolution in microbial communities. particularly when multiple closely related lineages coexist within the same sample. Their differences are encoded not only by SNVs, but also by accessory gene cassettes, inversions, prophage carriage, tandem duplications and large insertions. Mechanistically, long reads improve strain resolution because individual molecules preserve haplotypic linkage across distant variants, allowing SNVs, small indels and SV breakpoints to be phased over long genomic intervals rather than inferred from unlinked short-read fragments. Long molecules can also traverse structurally variable accessory loci, including BGCs, prophages, CRISPR arrays and IS-flanked islands, that would otherwise fragment assembly graphs or collapse related strains into consensus representations. Recent algorithmic advances further improve practical access to this resolution: myloasm uses polymorphic k-mers and high-resolution string graphs for modern HiFi and ONT R10.4 long reads, improving the recovery of complete circular contigs and previously inaccessible within-species diversity in metagenomic datasets[72]" in Lines 327-341.<br><br>References:<br>[72] Shaw J, Marin MG, Li H. High-resolution metagenome assembly for modern long reads with myloasm. Nat Biotechnol. 2026; doi: 10.1038/s41587-026-03053-z.<br><br>5. Section 4.1 — HMW DNA, low-abundance modifications and sequencing depth (Lines 488-491, p.23)<br>Two related points require clarification here. First, please explain explicitly why HMW DNA extraction improves the accuracy of methylation calling. Second, the statement that ONT "lacks sufficient accuracy in detecting very low-abundance modifications" is essentially equivalent to saying that sequencing depth is insufficient — and this is not a limitation specific to methylation analysis. At current depths, low-abundance chromosomes and MGEs frequently fail to assemble at all. I therefore recommend either reframing this limitation more precisely or, ideally, if possible, providing a practical description for the sequencing depth required to recover reliable methylation profiles from representative samples such as the human gut microbiome.<br>Response:<br>Thank you for this insightful comment. We fully agree with your comments and have revised the text accordingly. First, regarding HMW DNA extraction: we have now explicitly explained that its role in improving methylation calling accuracy lies in enabling longer reads and more contiguous assemblies, which are essential for reliably assigning methylation motifs to specific genomic elements. Second, you correctly pointed out that the original wording was imprecise and that the core issue is sequencing depth, a limitation not unique to methylation analysis. We have therefore reframed this statement to focus explicitly on depth, and we have added a practical depth benchmark with a note that even deeper sequencing may be needed for rare taxa in complex samples such as the human gut microbiome. The revised paragraph is as follows: "However, several challenges remain. Accurate long-read metagenomic methylation analysis depends on the recovery of HMW DNA, which is required to generate long reads and contiguous assemblies that enable reliable assignment of methylation motifs to chromosomes, plasmids, and phages. In addition, the methylation characterization of low-abundance taxa remains limited by sequencing depth. Ding et al. found that at the 25× sequencing depth recommended by PacBio, only 16.24% of phage genomes met this requirement, and the number of detected methylation sites increased with deeper sequencing[96]. Furthermore, different types of methylation modifications exhibit distinct depth requirements. For instance, 40× depth is sufficient to profile most 6mA sites, whereas 4mC resolution requires at least 60× depth to achieve comparable sensitivity[97]."(lines 514-524)

	References:<br>[96] Ding Y, Zhao L, Wang G, et al. PacBio sequencing of human fecal samples uncovers the DNA methylation landscape of 22 673 gut phages. Nucleic Acids Res. 2023; doi: 10.1093/nar/gkad977.<br>[97] Wilbanks EG, Dore H, Ashby MH, et al. Metagenomic methylation patterns resolve bacterial genomes of unusual size and structural complexity. ISME J. 2022; doi: 10.1038/s41396-022-01242-7.<br><br>6. Table 1 / Section 6 — Over-representation of CycloneSEQ studies and disclosure of competing interests<br>In Table 1, every entry from 2025-2026 uses a CycloneSEQ-WT02 + DNBSEQ-T5 hybrid strategy and is contributed by BGI-affiliated groups. Given that several of the authors of this review are affiliated with BGI Research, the present selection risks the appearance of a non-representative emphasis on a single platform that is commercially associated with the authors' institution. I recommend two adjustments: (i) explicitly add a Competing Interests statement disclosing the authors' affiliation with BGI and the relationship to the CycloneSEQ platform; and (ii) balance the recent rows of Table 1 (and the corresponding narrative in Section 6) with comparable 2024-2026 studies using ONT or PacBio platforms. This will substantially strengthen the reader's confidence that platform recommendations in this review reflect the broader field rather than the authors' in-house technology.<br>Response:<br>Thanks very much for these important suggestions. We agree that the previous version of the table over-represented recent CycloneSEQ-WT02 + DNBSEQ-T5 hybrid studies, which could create the impression of platform imbalance given our institutional affiliation. We have therefore revised the table to better represent recent developments across ONT, PacBio, and CycloneSEQ/DNBSEQ platforms. Specifically, we added recent ONT and PacBio studies evaluating LRS or hybrid bacterial genome reconstruction, including Abdel-Glil et al (2025, Microb Genom), Xu et al (2025, Nat Commun), Adams et al (2025, Microbiol Resour Announc), Nagy et al (2026, Microb Genom). We also added a Competing Interests statement disclosing the authors' affiliation with BGI Research and the institutional relationship to BGI-associated sequencing platforms as “The CycloneSEQ was initially developed by BGI Research and is now being marketed as an advanced technology in MGI. All authors except Yuzheng Gu are employees of BGI Research. The authors declare that this affiliation did not influence the selection, interpretation, or presentation of the literature reviewed in this article.” at Lines 797-800.<br>In Section 6, we revised the related text as “However, SRS alone often fail to resolve complete chromosomes, plasmids, long repeats, and complex structural variants. For isolated bacterial genomes, recent PacBio HiFi and ONT R10/R10.4.1 workflows have shown that complete or near-complete chromosomes and plasmids can often be reconstructed without mandatory short-read polishing, provided that coverage, read length, and quality-control metrics are adequate” at Lines 656-661.<br>Furthermore, we would like to respectfully point out that many studies in the original manuscript, including those by Zhao et al., Jiang et al., Canalda-Baltrons et al., Moss et al., Bickhart et al., Singleton et al., Sereika et al., Chin et al., Koren et al., Li et al., Fan et al., and Yan et al., were performed using ONT and PacBio platforms, and we presented their advantages in a balanced and objective manner. Thus, the manuscript has not exhibited a systematic bias toward the CycloneSEQ platform.<br><br>7. Section 6 / Abstract / Conclusion — Tone of the "hybrid remains the most practical strategy" recommendation should be revised<br>The manuscript's top-line recommendation — that "hybrid sequencing strategies remain the contemporary method" (Abstract line 47; Conclusion line 753) — no longer reflects the current state of the field. As the authors themselves note in Section 6, complete or near-complete genomes can often be recovered from isolates and microbiomes without mandatory short-read correction (Lines 667-669), and the field is moving toward Q30-class long reads as a realistic standard (Lines 671-672). In practice, for the most common use case — isolated genome sequencing — LRS-only workflows are already sufficient, and hybrid sequencing is only required in specific situations such as low-coverage regions, capture of rare community members in metagenomes, and applications where the highest possible consensus accuracy or conservative variant interpretation is critical (as the authors describe in Lines 657-662).

Powered by Editorial Manager® and ProduXion Manager® from Aries Systems Corporation

	I recommend that the authors revise the overall tone of Section 6, the Abstract, and the Conclusion to reflect this reality: namely, that LRS-only should now be presented as the default for isolate genomics and increasingly for metagenomics, with hybrid sequencing positioned as a targeted strategy for the specific conditions in which it remains clearly justified. Without this rebalancing, the current top-line statement risks understating the rapid accuracy gains of the past two years and inadvertently steering readers away from workflows that the same Section 6 acknowledges as already viable.<br>Response:<br>We thank the reviewer for this important comment. We agree that the original statement that "hybrid sequencing strategies remain the contemporary method" was too broad and could understate recent improvements in long-read accuracy. We have revised the Abstract , Section 6, Figure 4 caption and Conclusion to present sequencing strategy as application-dependent rather than as a single universal hierarchy as follows:<br>"Nevertheless, LRS is increasingly becoming the method of choice for isolate genomics and metagenomics" in the Abstract in Lines 45-46.<br>"In this applied context, the optimal choice among SRS, LRS, and hybrid sequencing is now best defined by the biological question, sample complexity, and accuracy requirement. SRS remains widely used in microbial genomics because it provides high-throughput, cost-effective depth, and high per-base accuracy, particularly for taxonomic profiling, abundance estimation, and large cohort studies. However, SRS alone often fail to resolve complete chromosomes, plasmids, long repeats, and complex structural variants. For isolated bacterial genomes, recent PacBio HiFi and ONT R10/R10.4.1 workflows have shown that complete or near-complete chromosomes and plasmids can often be reconstructed without mandatory short-read polishing, provided that coverage, read length, and quality-control metrics are adequate. This does not make hybrid sequencing obsolete. Instead, hybrid approaches remain particularly useful when the study requires maximal consensus accuracy, conservative SNP or indel interpretation, recovery of difficult plasmids, validation of problematic low-coverage, and homopolymer-rich regions. In metagenomics, LRS-only approaches are increasingly powerful for cMAG recovery and structural resolution, but uneven species abundance, rare community members, strain mixtures and assembly artefacts still make short-read supplementation or other orthogonal data valuable in selected settings." in the Section 6 in Lines 651-669.<br>"High-accuracy LRS and emerging ultra-long sequencing approaches (>100 kb) are expanding comprehensive resolution across isolate genomics and metagenomics, with hybrid sequencing retained for depth-limited or accuracy-critical applications." in the Figure 4 caption in Lines 743-746.<br>"Currently, while high-accuracy LRS is increasingly preferred for isolate genomics and metagenomics, hybrid sequencing is retained for depth-limited, difficult-replicon, rare-taxon, or accuracy-critical applications." in the Conclusion in Lines 773-776.<br><br>8. Sections 4.2 and 4.3 — Unclear metagenomic relevance of full-length transcriptomics and single-molecule proteomics<br>Sections 4.2 (full-length transcriptomics) and 4.3 (single-molecule proteomics) are written essentially as general technology overviews of long-read transcriptome / proteome sequencing, without demonstrating how either technique is actually applied — or could plausibly be applied — in a microbial metagenomic context. Given that the central theme of this review is the resolution of microbial complexity in complex communities, it is unclear what additional value these two sections contribute in their current form. Specifically: (i) Section 4.2 discusses bacterial isolate transcriptomics in detail, but does not address the metatranscriptomic challenges that would actually be relevant to community-scale analysis (e.g., full-length RNA recovery from complex communities, rRNA depletion, assignment of transcripts back to specific lineages or strains); (ii) Section 4.3 contains essentially no microbial application examples, as refs 96-100 are technology-development papers, and the connection to "microbial complexity" is not made explicit. I therefore recommend either substantially expanding these sections to include concrete metagenomic / community-level use cases (even at the pilot or proof-of-concept stage), or, if such examples are scarce at present, relocating this material to an Outlook box or a subsection of Section 6 (Future perspectives) and tightening the body of the review to its actual scope.<br>Response:<br>We thank the reviewer for this constructive and insightful feedback. We agree that the previous version of these sections focused too heavily on technical principles rather

Powered by Editorial Manager® and ProduXion Manager® from Aries Systems Corporation

than their direct relevance to resolving microbial complexity in community-level analyses. Accordingly, we have substantially revised Sections 4.2 and 4.3 as detailed below.

1.For Section 4.2: In the revised manuscript, we have added concrete application examples regarding community-scale analysis in lines 539-546 “For instance, ONT direct RNA sequencing extended the boundaries of 225 known E. coli operons and 89 defined S. aureus operons. Moreover, direct RNA sequencing enables the detection of native RNA modifications, such as m5C and m6A, providing new insights into the complexity of post-transcriptional regulation in bacteria. In a community context, integrating metatranscriptomics with nanopore long-read metagenomics enabled the reconstruction of trimethylamine metabolic networks and the identification of key microbial taxa[104]”. Furthermore, we also discussed technical challenges of applying LRS in microbiomes, such as full-length RNA recovery from complex communities, rRNA depletion, the difficulty of assigning transcripts to specific strains.“Nevertheless, applying LRS in the microbiome faces several technical challenges. Full-length RNA recovery is difficult due to rapid RNA degradation and inhibitory compounds (e.g., humic acids in soil)[105]. rRNA depletion is another essential step, as rRNA typically constitutes more than 90% of total bacterial RNA[106]. Assignment of transcripts to specific strains represents a third barrier, owing to high sequence similarity among closely related species and the lack of reference genomes[107].” in lines 546-552.

2.For Section 4.3: The reviewer correctly noted that this section contained no microbial application examples and that references 96–100 are technology-development papers. We agree with the reviewer's assessment, as direct nanopore protein sequencing has indeed not yet been validated on bacterial isolates or complex metaproteomic samples. Therefore, we have moved the entire original Section 4.3 into Section 6. This paragraph highlights recent technological breakthroughs, potential advantages over mass spectrometry, and prospective applications in microbial communities. “Recent advances in nanopore protein sequencing have demonstrated its feasibility for single-molecule proteomics, including repeated reading of single polypeptides, single-amino acid sensitivity, and peptide discrimination for protein identification. This platform offers potential advantages over conventional mass spectrometry, as it does not require enzymatic digestion, enabling the direct characterization of proteoforms and post-translational modifications at single-molecule resolution[148]. Although direct nanopore proteomics has not yet been applied to isolated bacterial strains or complex metagenomic samples, its prospective utility for microbiomics is substantial. In community settings, reading complete polypeptides could resolve the pervasive challenge of peptide sharing, where identical short sequences cannot be uniquely assigned among closely related strains.”in lines 706-716.

Reference:

[104] Simo C, Mamani-Huanca M, Hernandez-Hernandez O, et al. Application of nanopore long-read sequencing and metabolomics in an in vitro dynamic intestinal digestion model: A genome-centric metatranscriptomic approach to investigating microbial TMA and SCFA metabolism. J Pharm Biomed Anal. 2025; doi: 10.1016/j.jpba.2025.116896.

[105] Barber DG, Davies CA, Hartley IP, et al. Evaluation of commercial RNA extraction kits for long-read metatranscriptomics in soil. Microb Genom. 2024; doi: 10.1099/mgen.0.001298.

[106] Culviner PH, Guegler CK, Laub MT. A Simple, Cost-Effective, and Robust Method for rRNA Depletion in RNA-Sequencing Studies. mBio. 2020; doi: 10.1128/mBio.00010-20.

[107] Zhang W, Li XJ, Liu F, et al. Fungen: clustering and correcting long-read metatranscriptomic data for exploring eukaryotic microorganisms. Sci China Life Sci. 2026; doi: 10.1007/s11427-024-2853-x.

[148] Liu X, Dong Z, Timp G. Calling the amino acid sequence of a protein/peptide from the nanospectrum produced by a sub-nanometer diameter pore. Sci Rep. 2022; doi: 10.1038/s41598-022-22305-x.

Minor comments

1. p.4, Lines 82-83 — Additional citation suggested

The discussion of prophages confounding short-read assembly would benefit from an additional citation:

Wirbel J, Hickey AS, Chang D, Enright NJ, Dvorak M, Chanin RB, Schmidtke DT, Bhatt AS. Long-read metagenomics reveals phage dynamics in the human gut microbiome. Nature. 2025; 1-9.

Response:
We thank the reviewer for the specific citation suggestion. As recommended, we have added the reference (Wirbel et al., Nature, 2026) in the relevant section (Lines 81), where we discuss that short reads frequently fail to establish linkage between mobile genetic elements (including prophages) and the chromosome.

2. Insufficient in-text citation of figures
Although the manuscript contains four figures, the figures are not adequately cited at the relevant points in the main text. Please add explicit in-text citations at every point where the corresponding figure panel supports the narrative, so that readers can map the discussion to the visual summary.
Response:
We thank the reviewer for this careful observation. We agree that clear in-text citations help readers connect the narrative with the visual summaries. Accordingly, we have now added explicit citations of the figures at all relevant points throughout the manuscript. Specifically, “line131 – added citation of Figure1; line196 – added citation of Figure1; line303 – added citation of Figure2; line374 – added citation of Figure2; line498– added citation of Figure3; line531 – added citation of Figure3.” We believe these additions will improve the clarity and usability of the manuscript. Thank you again for this helpful suggestion.

Reviewer #2 - Major Revision
In this work, Rao and Gu et al. review the application of long read-sequencing to reconstruct microbial genomes from microbial isolate culture and metagenome-assembled genomes from microbiome samples. They provide an overview of the main long read-sequencing approaches and the analytical challenges they improve on, notably the resolution of extended repeats, genome rearrangements, and the detection of epigenetic modifications. While the authors cover the recent literature, I have the following comments:

Major comments:
1.The authors cite the work by Agustinho et al. (2024) Nature Methods. I found the overlap between the 2024 review and the present review quite large. Please specify what novel information the present review provides compared to previous reviews on long read-sequencing in the context of isolate genomes and/or metagenomes. It might be advisable to adjust the text accordingly for the reader to better appreciate what sets this review apart from other, very recent works.
Response:
Thank you for raising this important point. We have carefully read the 2024 review by Agustinho et al. in Nature Methods and fully acknowledge its value as a comprehensive methodological resource. In our revised manuscript, we have explicitly cited this work and clarified our distinct positioning in the Introduction (Lines 85-92): "For instance, Agustinho et al. provided a comprehensive synthesis of computational pipelines for long-read metagenomic assembly and taxonomic characterization, serving as an invaluable methodological reference. Nevertheless, that review was primarily methodological and centered on metagenomics. In contrast, our review systematically covers the reconstruction of gapless isolate genomes, the resolution of metagenomic populations with host–element linkages, and the characterization of native epigenomic and full-length transcriptomic landscapes." and at the beginning of the Section 6 (Lines 649-651): "While recent reviews, such as the work by Agustinho et al., focus primarily on computational workflows and software tools used for long-read data analysis, our review centers on practical applications across biological scales." Although both reviews address LRS and metagenomics, we believe that our review makes distinct contributions in the following aspects:
Divergent focus: The review by Agustinho et al. primarily focuses on metagenomic analysis pipelines (assembly, binning, taxonomic profiling, variant detection, etc.) and compares different bioinformatics tools, such as MetaMaps, MEGAN-LR, MMseqs2, CDKAM, and BugSeq for taxonomic classification. In contrast, our review focuses more on what biological questions LRS can help to answer. For example, in the context of isolate genomes, LRS enables precise localization of mobile genetic elements and allows investigation of their transfer between hosts (Section 2.4).
Expanded metagenomics content: For the metagenomics section that both reviews cover, we offer a more comprehensive discussion than Agustinho et al. Specifically, Section 3.3 (Lines 371-430) systematically discusses the use of LRS in establishing

host-element linkages, detailing how long molecular reads bridge assembly gaps to track mobile genetic elements, plasmids, phages, and antibiotic resistance genes. Furthermore, Section 3.4 (Lines 431-451) focuses on pathogen genomics and real-time detection, highlighting the clinical application of nanopore sequencing for rapid, culture-independent pathogen identification. These two parts are not detailed in their publication.

Broader scope: The review by Agustinho et al. is mainly centered on metagenomics. Our review covers three major application areas of LRS: isolate genomics, metagenomics, and multi-omics. Agustinho et al. barely discuss isolate genome assembly, whereas we provide an in-depth discussion in Section 2 on the contributions of LRS to microbial isolates. For instance, LRS enables the construction of complete, gapless bacterial genomes, which in turn allows the prediction of more functional genes. Furthermore, regarding extended applications, although both reviews touch upon methylation detection, we further discuss in Section 4 the use of LRS in transcriptomics and in Section 6 the emerging field of nanopore protein sequencing, these topics not covered in Agustinho et al. These unique sections constitute a substantial proportion of our total manuscript.

2.I found the text to read repetitive frequently, such as about fragmented assemblies using short reads and the possibility to sequence free of prior amplification with long reads. The authors refer to improved assembly using long reads but only introduce in section 5.3 how assembly actually takes place; a different order would help reduce redundancy.

Response：

We sincerely appreciate the reviewer’s careful reading and constructive feedback. We agree that our initial manuscript, in an effort to emphasize the limitations of SRS and the advantages of LRS, inadvertently introduced some repetitiveness. Section 1 already provides a comprehensive account of SRS shortcomings (repetitive regions, mobile genetic elements, GC bias, etc.). In later sections, we have eliminated redundant elaboration while preserving the key message. Following the reviewer’s suggestion, we have moved the comparison of SRS and LRS assembly strategies (including the explanation of De Bruijn graphs for SRS and OLC for LRS) from the original Section 5.3 to Section 2, where this earlier introduction helps readers understand the fundamental assembly differences, thereby reducing later redundancy and improving overall coherence. The specific modifications are as follows:

“SRS assembly typically employs De Bruijn graphs, which decompose short reads (typically 100–300bp) into short k-mers. As a result, short reads frequently fail to resolve complex repetitive regions, leading to gaps, misassemblies, and ambiguous contig ordering. In contrast, LRS adopts an overlap layout consensus (OLC) framework, where long reads (ranging from 10kb to 30kb or more) can span repetitive elements, enabling single circularized chromosomes and full reconstruction of extrachromosomal elements.” in lines 106-113.

“Although SRS platforms generate accurate reads that are well suited for identifying conserved genomic regions, these reads are too short to disentangle extrachromosomal sequences with repetitive regions.” in lines 168-170.

“However, short-read metagenomic sequencing often yields fragmented assemblies, which detach functional genes from their chromosomal, plasmid, or phage contexts.”in lines 262-264.

“Historically, SRS relied on bisulfite chemical conversion, which frequently caused DNA degradation and severe GC bias, thus failing to simultaneously detect DNA sequence and native modifications at single-molecule resolution.” in lines 488-491.

3.The authors declare no competing interests. However, Rao and Zou are both affiliated to BGI Research, with BGI producing the CycloneSeq platform. I think that in the interest of transparency, this should be disclosed by the authors.

Response:

We thank the reviewer for raising this important point regarding transparency. We fully agree that any potential affiliation with a commercial platform developer should be explicitly disclosed. In light of this suggestion, we have added the following statement to the Competing interests section (lines 797-800):“The CycloneSEQ was initially developed by BGI Research and is now being marketed as an advanced technology in MGI. All authors except Yuzheng Gu are employees of BGI Research. The authors declare that this affiliation did not influence the selection, interpretation, or presentation of the literature reviewed in this article.” We believe this addition appropriately ensures

	full transparency.<br><br>4.The authors repeatedly refer to "physical linkage". Please carefully review this and appropriate use of respective phrasing in the text. While long read-sequencing can produce circular chromosomes and circular plasmids, physical linkage of these genetic elements is only established through complementary approaches, e.g., proximity ligation approaches. Put differently, circular chromosomes and circular plasmids are not automatically put into their shared organismal context through long read-sequencing.<br>Response:<br>We thank the reviewer for this rigorous comment. We fully agree that in its strict sense, “physical linkage” implies direct molecular connection, which LRS cannot establish across different replicons. Our intended meaning throughout the manuscript was to describe the ability of LRS to resolve the localization of MGEs, such as determining whether an MGE is located on a chromosome or a plasmid, and its precise position within a replicon. We realize that our original wording “physical linkage” was imprecise and could mislead readers, and we appreciate the reviewer for pointing this out.<br>To address this concern, we have thoroughly revised the manuscript to replace ambiguous uses of “physical linkage” with more accurate terminology. Specifically, “In metagenomics, LRS enables high-resolution strain parsing, the recovery of circular metagenome-assembled genomes, and the precise localization of MGEs within host replicons.”in Lines 38-40. In the Introduction, we revised the related text as“Furthermore, the ubiquity of MGEs, such as plasmids and prophages, complicates reconstruction because short reads frequently fail to establish their integration sites within the primary chromosome” in Lines 79-81.<br><br>5. In the interest of consistency, please include a table like Table 1 but for metagenome-assembled genomes. Importantly, the use of long read-sequencing for isolate genomes is largely an accepted standard by now, while the use for the recovery of metagenome-assembled genomes remains a challenge.<br>Response:<br>Good suggestion! We agree that the manuscript should provide a metagenome-focused counterpart to the isolate-genome table, particularly because LRS-based isolate genome closure is now comparatively mature whereas MAG/cMAG recovery from metagenomes remains technically more challenging. We have added a new table as Table 2 summarizing representative long-read and hybrid studies for MAG/cMAG recovery, including the sequencing strategy, sample type, and main genome-recovery output.<br>Table 2 Representative LRS studies for MAG and cMAG recovery from metagenomes.<br>YearStudyPlatform and strategyMetagenomic datasetMAG/cMAG recovery<br>2019Bertrand et al.Illumina + ONT nanopore; OPERA-MS hybrid assemblyMock communities and 28 human gut metagenomesImproved contiguity ~200-fold; recovered rare-species genomes and >80 closed plasmid/phage sequences.<br>2020Moss et al.ONT MinION R9.4; Lathe workflow with short-read polishing13 human stool metagenomesRecovered 20 circular bacterial genomes directly from microbiomes.<br>2021Singleton et al.ONT PromethION + Illumina; long-read assembly with short-read supportActivated sludge from 23 Danish WWTPsRecovered 1,083 HQ MAGs, including 57 closed circular genomes.<br>2022Bickhart et al.PacBio HiFi + Hi-C binning; MAGPhase haplotype phasingSheep faecal metagenomeRecovered 428 MAGs, including 44 single-contig circular MAGs and 220 lineage-resolved MAGs.<br>2022Feng et al.PacBio HiFi; hifiasm-meta assemblerSeven empirical metagenomic datasetsReconstructed tens to hundreds of complete circular...
**Additional Information:**	
**Question**	**Response**
Are you submitting this manuscript to a special series or article collection?	No
**Experimental design and statistics**	No

Full details of the experimental design and statistical methods used should be given in the Methods section, as detailed in our Minimum Standards Reporting Checklist. Information essential to interpreting the data presented should be made available in the figure legends.<br><br>Have you included all the information requested in your manuscript?	
If not, please give reasons for any omissions below.<br><br>as follow-up to "**Experimental design and statistics**<br><br>Full details of the experimental design and statistical methods used should be given in the Methods section, as detailed in our Minimum Standards Reporting Checklist. Information essential to interpreting the data presented should be made available in the figure legends.<br><br>Have you included all the information requested in your manuscript?<br><br>"	This manuscript is a review article and does not present original experimental data. Therefore, no experimental design or statistical methods are applicable. The manuscript has been prepared in accordance with the journal's guidelines for reviews, and all relevant information has been included.
**Resources**<br><br>A description of all resources used, including antibodies, cell lines, animals and software tools, with enough information to allow them to be uniquely identified, should be included in the Methods section. Authors are strongly encouraged to cite Research Resource Identifiers (RRIDs) for antibodies, model organisms and tools, where possible.<br><br>Have you included the information requested as detailed in our Minimum Standards Reporting Checklist?	No

If not, please give reasons for any omissions below.<br><br>as follow-up to "**Resources**<br><br>A description of all resources used, including antibodies, cell lines, animals and software tools, with enough information to allow them to be uniquely identified, should be included in the Methods section. Authors are strongly encouraged to cite Research Resource Identifiers (RRIDs) for antibodies, model organisms and tools, where possible.<br><br>Have you included the information requested as detailed in our Minimum Standards Reporting Checklist?<br><br>"	This manuscript is a review article and does not present original experimental data. Therefore, no experimental design or statistical methods are applicable. The manuscript has been prepared in accordance with the journal's guidelines for reviews, and all relevant information has been included.
**Availability of data and materials**<br><br>All datasets and code on which the conclusions of the paper rely must be either included in your submission or deposited in publicly available repositories (where available and ethically appropriate), referencing such data using a unique identifier in the references and in the "Availability of Data and Materials" section of your manuscript.<br><br>Have you have met the above requirement as detailed in our Minimum Standards Reporting Checklist?	No
If not, please give reasons for any omissions below.<br><br>as follow-up to "**Availability of data and materials**<br><br>All datasets and code on which the conclusions of the paper rely must be	This manuscript is a review article and does not present original experimental data. Therefore, no experimental design or statistical methods are applicable. The manuscript has been prepared in accordance with the journal's guidelines for reviews, and all relevant information has been included.

Powered by Editorial Manager® and ProduXion Manager® from Aries Systems Corporation

either included in your submission or deposited in publicly available repositories (where available and ethically appropriate), referencing such data using a unique identifier in the references and in the “Availability of Data and Materials” section of your manuscript.<br><br>Have you have met the above requirement as detailed in our Minimum Standards Reporting Checklist?<br><br>"	
GigaScience has policies and guidelines in place for the use of generative AI-writing tools such as ChatGPT. If you have used such writing tools to assist with writing the manuscript this must be declared and cited in the text. Authors should not list AI-writing tools and other AI-assisted technologies as an author or co-author and should acknowledge that they are fully responsible for text generated or refined by AI-writing tools.<p><br><br>A summary of use (particularly in the introduction or among methods) needs to be included at the end of the paper, and the outputs should also be included as a supplementary file hosted in GigaDB or other open repositories. Please read our guidelines for more information. <p><br><br>By submitting to GigaScience, you are aware of the journal's AI-writing tools policy, and if you have declared use of such tools below, you have acknowledged this where appropriate in your manuscript and have made a summary of use and outputs available. <p><br><b>AI-assisted writing tools have been used in the preparation of this manuscript?	Yes

Manuscript

Click here to access/download;Manuscript;Giga_manuscript_revised.docx

Promises and Pitfalls of Long-Read Sequencing for Resolving Microbial Complexity

Xing Rao (饶兴)[1,2#], Yuzheng Gu (顾雨铮)[3#], Gabriella[1,4], Jiayi Ma (马嘉仪)[1,2], Haoyu Wang (王浩宇)[1,2], Yuanqiang Zou (邹远强)[1,5]

1. State Key Laboratory of Genome and Multi-omics Technologies, BGI Research, Shenzhen 518083, China

2. College of Life Science, University of Chinese Academy of Sciences, Beijing 100049, China

3. Key Laboratory of Agricultural and Environmental Microbiology, Ministry of Agriculture and Rural Affairs, Department of Microbiology, College of Life Sciences, Nanjing Agricultural University, Nanjing 210095, China

4. Department of Food Science and Biotechnology, Faculty of Agricultural Technology, Universitas Brawijaya, Malang 65145, Indonesia

5. Shenzhen Key Laboratory of Human commensal microorganisms and Health Research, BGI Research, Shenzhen 518083, China

# These authors contributed equally.

Corresponding author:

Yuanqiang Zou.

Telephone: +86-13147016380

Fax: 0755-36352579

Address: State Key Laboratory of Genome and Multi-omics Technologies, BGI

Research, Shenzhen 518083, China

Email: zouyuanqiang@genomics.cn

Yuanqiang Zou will handle the correspondence of the article at all stages of the review and publication process, as well as post-publication.

Author's email: raoxing@genomics.cn, guyuzheng513@163.com, gabriella@genomics.cn, majiayi@genomics.cn, wanghaoyu2@genomics.cn , zouyuanqiang@genomics.cn

Abstract

Long-Read Sequencing (LRS) has driven a transition in microbial genomics, overcoming the assembly fragmentation inherent to short-read sequencing.

This review elucidates the impact of LRS across isolate genomics, metagenomics, and multi-omics domains. By spanning extensive repetitive regions, LRS facilitates the reconstruction of gapless circular chromosomes and precisely resolves mobile genetic elements (MGEs). In metagenomics, LRS enables high-resolution strain parsing, the recovery of circular metagenome-assembled genomes, and the precise localization of MGEs within host replicons. Furthermore, the single-molecule, amplification-free properties of LRS provide enhanced resolution of native epigenetic modifications and full-length transcriptomes. Despite these advancements, widespread implementation remains constrained by multidimensional challenges, including stringent high-molecular-weight DNA requirements, depth deficits, and

computational overhead. Nevertheless, LRS is increasingly becoming the method of choice for isolate genomics and metagenomics.

As detection technologies and algorithms progress, LRS will establish a contiguous, multidimensional landscape for deciphering microbial ecosystems.

Keywords: Long-read sequencing, Microbial genomics, Metagenomics, Genome assembly.

1. Introduction

The evolution of sequencing technologies has facilitated the resolution of structural complexity in microbial genomes. From Sanger sequencing to single-molecule real-time platforms, each technical transition reflects improvements in throughput, cost efficiency, and analytical scope. Sanger sequencing, characterized by dideoxynucleotide chain termination, enabled the first genome draft assembly of a cultured microorganism, *Haemophilus influenzae*, marking the formal entry of microbiology into the genomic era[1, 2]. However, throughput constraints necessitated the development of short-read sequencing (SRS) platforms such as Illumina and DNA nanoball sequencing (DNBSEQ) [3, 4]. By integrating bridge amplification with sequencing-by-synthesis, SRS circumvented the need for microbial cultivation and supported landmark metagenomic initiatives like the Human Microbiome Project and METAgenomics of the Human Intestinal Tract[5-7]. Despite these advances, the short-read lengths often fail to resolve intricate genomic architectures, resulting in fragmented assemblies[8]. LRS platforms, exemplified by PacBio, Oxford Nanopore Technologies (ONT) and CycloneSEQ, generate read

lengths that exceed SRS capabilities by several orders of magnitude, effectively addressing the above assembly challenges[9-11]. This progress enables the transition from fragmented draft assemblies to the reconstruction of high-quality, circularized chromosomes[12-14], surmounting the structural constraints that have historically hindered contiguous assembly in SRS workflows.

The inability of SRS to achieve gapless genome closure stems from the inherent disparity between short-read lengths and the structural complexity of microbial chromosomes. Central to this challenge is the high density of repetitive elements, which can constitute up to 10% of some bacterial genomes and thus present formidable challenges to computational reconstruction[15-17]. These regions, primarily composed of ribosomal RNA (rRNA) operons and insertion sequences (ISs), often exhibit sequence lengths and identities that exceed the disambiguation capacity of SRS algorithms[17-19]. Furthermore, the ubiquity of MGEs, such as plasmids and prophages, complicates reconstruction because short reads frequently fail to establish their integration sites within the primary chromosome[20-22]. Compositional challenges, including extreme guanine–cytosine(GC) content, further exacerbate assembly difficulties due to coverage biases and the underrepresentation or loss of such regions[23-25]. Collectively, these intrinsic genomic features result in fragmented draft assemblies that impede accurate phylogenetic inference and the comprehensive functional annotation of bacterial genomes.

In response to these challenges, LRS has emerged as an important tool, shifting the microbiological research from fragmented observations toward holistic genomic

resolution. However, current reviews have focused on biomedical surveys or algorithmic analyses[26-28]. For instance, Agustinho et al. provided a comprehensive synthesis of computational pipelines for long-read metagenomic assembly and taxonomic characterization, serving as an invaluable methodological reference[29]. Nevertheless, that review was primarily methodological and centered on metagenomics. Our review systematically covers the reconstruction of gapless isolate genomes, the resolution of metagenomic populations with host–element linkages, and the characterization of native epigenomic and full-length transcriptomic landscapes. In particular, we also offer an evaluation of the technical disadvantages of LRS relative to SRS. By synthesizing these integrative perspectives, this work establishes a strategic framework for microbial research, underscoring the necessity of LRS in resolving the intricate complexity of diverse microorganisms.

2. Genomics of single isolates: reshaping the standard of "reference genomes."

The standard for high-quality reference genomes has expanded beyond base accuracy to requiring structural completeness (Figure 1). This change is driven by broader access to analytical tools and reduced sequencing costs[30-33]. Historically, single-isolate microbial reference genomes relied heavily on SRS platforms[34]. SRS assembly typically employs De Bruijn graphs, which decompose short reads (typically 100–300 bp) into short k-mers[30, 31, 35]. As a result, short reads frequently fail to resolve complex repetitive regions, leading to gaps, misassemblies, and ambiguous contig ordering[36-38]. In contrast, LRS adopts an overlap layout

consensus (OLC) framework, where long reads (ranging from 10 kb to 30 kb or more) can span repetitive elements, enabling single circularized chromosomes and full reconstruction of extrachromosomal elements[39].

Figure 1 Comparative assembly of single-isolate microbial genomes using SRS and LRS. Conventional SRS implementations generate highly fragmented draft genomes, inherently constrained by their inability to resolve complex repetitive elements, differentiate extrachromosomal plasmids, or capture structural variations (SVs). Conversely, LRS harnesses extended read lengths to span these challenging structural complexities, yielding gapless, complete genomes that improve spatial positioning, support plasmid resolution, and enable comprehensive SV detection for downstream genomic interpretations.

Alt text: A comparative flowchart on microbial genome assembly using SRS and LRS, illustrating the technical transition from fragmented draft genomes to gapless, complete circularized chromosomes and resolved genomic features.

2.1. Achieving gapless genomes: from fragmentation to continuity

The commercialization of LRS platforms, initiated by PacBio SMRT in 2011 and followed by ONT MinION in 2014, represented a milestone in third-generation sequencing technologies[40]. LRS platforms generate reads spanning tens to hundreds of kilobases, bridging repetitive regions (Figure 1). These capabilities did not simply

improve microbial assemblies incrementally, but fundamentally updating the attainable standard for single-isolate reference genomes (Table 1). In SRS, even datasets with high-accuracy read per base frequently yielded fragmented draft assemblies since reads are too short to span complex regions[38].

Chin et al. showed that PacBio SMRT sequencing was able to resolve long repeat regions and produce *de novo* microbial genome assemblies with over 99.999% consensus accuracy[41]. This demonstrated that complete genomes no longer required labor-intensive finishing strategies. Koren et al. demonstrated LRS capability by assembling six bacteria genomes and analyzed repeat complexity across 2,267 complete bacterial and archaeal genomes, including plasmid and repetitive regions[42]. They concluded that most microbial genomes should be closeable without gaps using a single PacBio RS library. The advantage of LRS was not limited to contiguity; ultra-long reads exceeding 32 kb enabled sequencing of entire adhesin-encoding genes (>6 kb) and antibiotic biosynthesis loci (>15 kb), regions previously difficult to reconstruct with short-read data[43]. Beyond genome completeness, LRS also enhanced functional recovery. For *Lactiplantibacillus pentosus* LP309, LRS resolved its complete chromosome and eight plasmids, enabling the prediction of over 150 probiotic functional genes involved in acid resistance and vitamin synthesis[44]. A recent human gut genome collection of 1,150 circular genomes across 199 species-level clusters further demonstrated that complete assemblies substantially improve downstream functional inference[45]. Specifically, metabolic models constructed from these complete genomes recovered 4,764

additional reactions relative to draft-based models, the majority of which were transport-related[46].

Collectively, these studies catalyzed a shift in expension of genome quality standards—from a draft-focused approach to a new expectation of finished, gapless assemblies. This transition was anchored in the recent ability to recover genome continuity at the chromosomal level, thus redefining reference genome quality beyond conventional contiguity metrics. This shift established structural completeness as a defining feature of high-quality reference genomes and provides the basis for further discussion for high-confidence functional and structural analysis.

2.2. Beyond the chromosome: unveiling the full architecture of plasmids and mobile genetic elements

Microbial genomes harbor a diverse repertoire of mobile genetic elements (MGEs), including plasmids, transposons, ISs, integrons, integrative and conjugative elements (ICEs), and prophages, that drive horizontal gene transfer (HGT)[47]. Although SRS platforms generate accurate reads that are suited for identifying conserved genomic regions, these reads are too short to disentangle extrachromosomal sequences sharing homologous regions with the chromosome[48]. SRS plasmid reconstruction remains unreliable in a benchmark of 42 complete bacterial genomes containing 148 plasmids, particularly for large plasmids with repetitive regions[49]. As a result, accessory genes encoded within these structures, such as antibiotic resistance genes (ARGs) and virulence factors, are fragmented or

incorrectly assigned, which may lower the accuracy of the analysis results[50, 51].

LRS has enhanced the landscape by enabling direct assembly of entire plasmids as individual replicons, preserving the associated MGE context. Jiang et al. reconstructed resistance plasmid architecture using PacBio SMRT sequencing, characterizing pooled conjugative plasmids from multidrug-resistant *Klebsiella pneumoniae*[52]. They identified 124 ARGs and 317 MGEs, including transposons, ISs, and integrons, and established a relationship network between MGEs and ARGs that described dissemination by mobile elements. Key resistance transposition elements included IS26, Tn3, IS903B, ISEcp1, and ISKpn19, and they illustrated an IS26-mediated multicopy composite transposition event. ONT R10.4.1 data recently showed that finished bacterial and plasmid genomes can be generated without SRS, achieving accuracies above 99.99% for a 5-Mb bacterial genome at 75× coverage[53]. Beyond structural resolution, reconstructed plasmids also revealed biological relevance. For instance, McLeod et al. resolved the extensive plasmid complement of *Lactiplantibacillus plantarum* MF1298, yielding 14 complete plasmid sequences, one of which carried functional genes associated with vitamin B12 transformation[54]. Wang et al. showed that resolved plasmid-borne genes included functions related to fructose metabolism, indicating that accessory replicons can contribute directly to host-associated metabolic capacity[45].

2.3. Resolving microbial variations and evolutionary dynamics

The use of LRS technologies facilitates the identification of a broader spectrum of variations (Figure 1). Although one of the clearest advantages of LRS lies in

resolving large inversions, duplications and other structural variants, they also improve the detection and interpretation of smaller-scale variants such as single nucleotide variants (SNVs), when these variants occur within complex genomic architecture. Recent ONT-based benchmarking showed that Illumina- and k-mer-based SNP typing failed to detect 9 to 68 SNPs per 1,000 sites in repetitive reference regions. Conversely, by spanning these complex regions on one long single molecule, ONT long reads improved SNP calls across the previously inaccessible genomic regions, where SRS alignment is at disadvantage[55]. Capturing this broader view is essential as it shows that microbial evolution depends not just on massive structural shifts, but also on small, localized mutations and shifting repeat regions[56]. Recently, Waters argued that genome structure variation should be considered part of genome-based bacterial surveillance, but this requires complete circularized genomes[57]. In case studies of *Salmonella enterica* serovar Typhi, researchers confirmed genome rearrangements generated during long-term culture using LRS. All isolates with altered genome structures had changes in gene expression compared to the parental strain[57]. The most extreme rearrangement caused a stronger imbalance between the origin and terminus of replication and was associated with more pronounced transcriptional changes, showing that chromosome-scale structural variation can influence phenotype even when nucleotide-level sequence changes are limited. Similarly, in *Bordetella pertussis*, LRS confirmed both duplications and a large inversion of approximately 1 Mbp between isolates from the same patient. This demonstrates that large rearrangements can arise and persist during both within-host

and laboratory evolution[58]. LRS also helps detect microbial SVs by resolving genome-scale rearrangements and mobile-element-associated chromosomal changes that are difficult to reconstruct from fragmented assemblies. In *Salmonella enterica*, LRS of 134 multidrug-resistant isolates revealed variation in the presence and orientation of *Salmonella* pathogenicity islands, as well as chromosomal insertion of resistance plasmids and genomic islands. This highlights the value of LRS in resolving genome plasticity and resistance-associated structural changes[59].

On an evolutionary scale, the value of LRS is reinforced by population-level analyses. Canalda-Baltrons et al. constructed a pangenome reference graph from 859 high-quality LRS assemblies of *Mycobacterium tuberculosis* and showed that SVs are not marginal events, but an important component of genome evolution, virulence-associated deletions, and drug-resistance architecture[60]. Their subsequent genotyping across 41,134 isolates linked recurrent SVs to multiple first- and second-line drug resistance phenotypes, underscoring that long-read-enabled SV analysis contributes not only to structural characterization, but also to understanding clinically relevant adaptation. Taken together, these studies show that the key contribution of LRS is not merely improved contiguity, but the ability to recover chromosome-scale structural change with sufficient precision to study its functional and evolutionary consequences.

2.4 Precise localization of genetic elements through complete genomes

The precise localization of MGEs provides a critical window into understanding microbial organisms. Once chromosomes and accessory replicons are fully assembled,

genetic elements that would otherwise appear as disconnected fragments in draft genomes can be assigned to a defined chromosomal or plasmid backbone. Sheppard et al. used a hybrid whole-genome sequencing approach to analyze 281 blaKPC-positive *Enterobacteriaceae* isolates and showed that blaKPC dissemination occurred through multiple linked genetic levels, including movement of host strains, plasmid transfer across strains and species, and transposition between plasmids[61]. In this setting, complete plasmid structures revealed how resistance dissemination depends on the organization of elements across strains, plasmids and transposons, and showed why partial typing or reference-based inference can misrepresent the true pathways of gene spread. Beyond resistance spread, complete genome resolution of the probiotic *Lactobacillus crispatus* precisely anchored Tn7088, a novel integrative mobile element encoding a class I bacteriocin biosynthetic gene cluster that drives antimicrobial competition and probiotic fitness[62]. At a larger scale, Benler et al. delineated 6,549 Tn7-like transposons at base-pair resolution and showed that 96% carried cargo genes enriched not only in defense and antibiotic resistance functions but also in central carbon metabolism[63]. Together, these findings show that complete genome assemblies both recover more sequence, and reveal the genomic context required to understand mobility and adaptation.

3. Metagenomics of microbial communities: from "fragments" to "panoramas"

Metagenomics has vastly expanded our understandings of the diversity and metabolic potential of uncultured microorganisms. However, short-read metagenomic

sequencing often yields fragmented assemblies, which detach functional genes from their chromosomal, plasmid, or phage contexts[29, 64]. Recent improvements in the throughput and raw-read accuracy of LRS, together with long-read-aware assemblers, polishers and binning frameworks, have begun to relax this constraint[29, 65]. Long-read metagenomics is therefore moving beyond taxonomic inventories towards a more integrated framework in which complete genome architecture, DNA modifications, and ecological interactions can be interrogated within the same datasets (Figure 2 and Table 2).

Figure 2 Applications of long-read metagenomics in microbial ecosystems. This schematic delineates the multidimensional analytical capacities unlocked by LRS to overcome the fragmentation limitations of conventional metagenomics. LRS facilitates the reconstruction of circular metagenome-assembled genomes (cMAGs), expanding genome-resolved phylogenetic diversity by generating complete or near-complete genome representatives that can be placed in phylogenomic frameworks and linked to functional elements. Concurrently, the recovery of intact functional loci, such as biosynthetic gene clusters (BGCs) and CRISPR systems, enables robust metabolic and functional profiling. High-resolution haplotype phasing further allows for the dynamic monitoring of strains. Furthermore, LRS supports evidence-based host-MGE association by resolving integration junctions and improving phage-host inference, while complementary strategies provide evidence for assigning plasmids, phages and ARG-bearing elements to likely hosts.

Alt text: A four-panel schematic illustrating the impact of LRS on metagenomics. It highlights the transition from fragmented data to cMAGs for diversity expansion, the recovery of intact functional loci for metabolic profiling, strain-level tracking of structural variations, and the establishment of host-element linkages to map horizontal gene transfer networks.

3.1 Circular metagenome-assembled genomes

One immediate consequence of this shift is the increasing feasibility of recovering complete or near-complete genomes directly from microbial communities. High-quality metagenome-assembled genomes (MAGs) provide the structural basis for metabolic reconstruction, comparative genomics and ecological interpretation. However, in highly heterogeneous ecosystems, such as soils and aquatic environments, species abundance spans a broad dynamic range. Under these conditions, short-read binning strategies are prone to missing low-abundance but ecologically informative taxa and often fail to reconstruct highly fragmented sequences into closed genomes[64, 66]. Long-read metagenomic sequencing addresses this limitation by generating contiguous molecules that span tens to hundreds of kilobases, thereby enabling recovery of cMAGs and rescuing genomic regions that are routinely lost from short-read assemblies because of structural complexity (Figure 2).

Early nanopore-based studies showed that closed bacterial genomes could be recovered directly from human faecal metagenomes, indicating that complete community genomes were no longer restricted to isolate sequencing[67]. At

population scale, this additional continuity translates directly into a more complete view of microbiome diversity. In a study of Southeast Asian gut microbiomes integrating LRS with Hi-C, more than 1,700 MAGs were absent from short-read-only analyses, assembly contiguity improved by more than 28-fold, and thousands of population-specific strains and biosynthetic gene clusters were recovered[66]. Similar gains have been reported outside the human gut. In activated sludge, hybrid long- and short-read approaches generated more than 1,000 high-quality MAGs, including dozens of circular genomes, and restored full-length rRNA genes that are typically absent from short-read bins[68]. More recently, deep long-read surveys across terrestrial habitats recovered genomes representing more than 15,000 previously undescribed genomes and expanded the known phylogenetic breadth of prokaryotic diversity. Meanwhile, LRS converted divergent marker-gene signals or fragmented metagenomic assemblies into high-quality genome representatives that can be placed in phylogenomic frameworks and linked to complete ribosomal RNA operons, BGCs, and CRISPR-Cas systems[69]. This indicates that long-read metagenomics contributes not only to improved assembly of known taxa and recovery of lineages that remain poorly represented in genome-centred reference frameworks, but also to help explore repetitive, structurally complex, and functionally critical elements that define microbial adaptability and evolution.

3.2 High-resolution strain-level parsing

Long-read metagenomics is also redefining strain resolution in microbial communities. particularly when multiple closely related lineages coexist within the

same sample. Their differences are encoded not only by SNVs, but also by accessory gene cassettes, inversions, prophage carriage, tandem duplications and large insertions. Mechanistically, long reads improve strain resolution because individual molecules preserve haplotypic linkage across distant variants, allowing SNVs, small indels and SV breakpoints to be phased over long genomic intervals rather than inferred from unlinked short-read fragments. Long molecules can also traverse structurally variable accessory loci, including BGCs, prophages, CRISPR arrays and IS-flanked islands, that would otherwise fragment assembly graphs or collapse related strains into consensus representations[29, 70, 71]. Recent algorithmic advances further improve practical access to this resolution: myloasm uses polymorphic k-mers and high-resolution string graphs for modern HiFi and ONT R10.4 long reads, improving the recovery of complete circular contigs and previously inaccessible within-species diversity in metagenomic datasets[72]. These principles have been demonstrated in animal and human gut microbiomes. In sheep faecal microbiomes, LRS recovered lineage-resolved complete MAGs and revealed hidden population structure, while improving assignment of BGCs and mobile elements to specific hosts[71]. In the human gut microbiome, long-read analyses further showed that SV is highly individualized and contributes substantially to within-species diversity[70]. Together, these studies show that strain-level interpretation often depends on multi-kilobase genomic segments that are poorly represented in short-read consensus assemblies.

The clinical relevance of this enhanced resolution is becoming increasingly apparent. In faecal microbiota transplantation (FMT), therapeutic interpretation

depends not only on whether a species is detected after treatment, but also on which donor strain engrafts and persists in the recipient gut. Long-read metagenomics enables post-FMT strain tracking with substantially greater specificity than short-read approaches, particularly when multiple conspecific strains coexist in the same sample. In cohorts with recurrent *Clostridioides difficile* infection and inflammatory bowel disease, long-read analyses identified hundreds of engrafted donor strains and tracked their persistence for up to five years, while also capturing epigenetic stability and large-scale SVs acquired during adaptation to the recipient environment[73]. In a paediatric undernutrition cohort, Minich et al. used long-read metagenomics to follow the gut microbiota over 11 months and linked SV in commensal strains to host growth-associated phenotypes, suggesting that clinically relevant microbiome signals may depend on fine-scale genomic plasticity rather than taxonomic composition alone[74]. These findings indicate that strain resolution should not be viewed as a technical refinement of species-level profiling, but as a shift towards a more appropriate biological scale. Many clinically and ecologically relevant processes, including engraftment, persistence, competition and adaptation, operate at the level of strains rather than species. Long-read metagenomics therefore offers a framework in which microbial population dynamics can be interpreted in terms of inheritance, selection and genome plasticity, rather than inferred indirectly from abundance changes alone.

3.3 Establishing host-element linkages

One of the most important contributions of LRS to microbial ecology is its

ability to bridge the assembly gap between MGEs and their host chromosomes (Figure 2). In short-read datasets, the flanking regions of ARGs, virulence factors, and prophages are often enriched in transposases and ISs, causing these loci to be isolated on short fragments within assembly graphs, while shared backbone regions, repeats, and embedded transposable elements can prevent complete plasmid reconstruction and obscure their host associations[64, 75]. Consequently, researchers can quantify the abundance of these elements, yet still lack direct evidence for their host range or transfer potential. LRS can strengthen host-MGE analysis in two distinct ways. For integrated MGEs, including prophages, reads or contigs that span the element-chromosome junction provide direct sequence evidence for the host genome[29, 76]. In addition, longer viral and bacterial contigs can improve phage-host association by recovering more complete viral genomes, CRISPR arrays and host taxonomic signals. For example, viralFlye showed that long-read assemblies increased CRISPR-based virus-host predictions and improved host assignment precision relative to short-read assemblies[77]. Extrachromosomal plasmids are often harder to assign because they lack obligatory chromosomal junctions, have mosaic sequence composition and may circulate across multiple hosts. Therefore, plasmid-host and ARG-bearing MGE assignments in metagenomes often require orthogonal evidence, such as Hi-C/3C proximity ligation, methylation-aware binning, or ultra-long reads when they span integration or cointegration junctions[29, 78].

Beyond host assignment, LRS is reshaping mobilome discovery by recovering complete extrachromosomal elements and resolving their structural diversity. In the

human gut, long-read metagenomics enabled direct recovery of complete extrachromosomal mobile genetic elements, revealing plasmids and phage-associated DNA that are underrepresented in short-read catalogues[79]. In the oral microbiome, long-read assembly uncovered a family of giant plasmid-like extrachromosomal elements associated with Streptococcus, indicating that mobile gene pools in host-associated communities have been substantially underestimated when inferred from fragmented assemblies alone[80]. Long reads have also sharpened analyses of phage ecology. In a two-year longitudinal study of the human gut microbiome, Wirbel et al. found that prophage-host relationships were more flexible than predicted by a strictly host-specific model: bacterial lineages with and without particular prophages could coexist stably, and some phages were detected in distinct host lineages across family boundaries[22]. The same study identified a class of phages termed “IScream” phages, which appear to exploit bacterial IS30 transposases to mediate their own mobility and integration. These findings demonstrate that the mobilome is more dynamic and cross-lineage than previously appreciated, and that LRS now can be the primary tool for resolving this complexity.

The advantage of LRS is particularly valuable in resistome analysis. In an early proof-of-principle study, combining long-read assembly with proximity ligation substantially increased recovery of ARGs and virus-host associations in the rumen microbiome relative to short-read assembly alone[75]. Works in wastewater treatment plants, activated sludge systems and urban sewage networks showed that LRS can determine whether specific ARGs are stably chromosome-encoded or carried by

conjugative plasmids, while resolving how host associations and mobility profiles change along environmental gradients[81-83]. Long-read datasets have likewise clarified the effects of local selection pressures on MGE dynamics. In hospital wastewater, chlorine disinfection was associated with altered community structure, increased ARG diversity and higher mobility potential within the resistome[84]. In microplastic-associated biofilms, plasmid-borne ARGs were enriched relative to surrounding water and natural rock substrates, suggesting that artificial polymer surfaces may act as hotspots for plasmid-mediated resistance transfer[85]. These works imply that LRS connects function to mechanism. In short-read datasets, the detection of an ARG, virulence determinant or prophage often supports only descriptive inference. Once these elements are placed onto specific chromosomes or plasmids, however, the same data begin to inform questions of mobility, host range, persistence, and transmission. Long-read metagenomics therefore shifts resistome and mobilome analysis from compositional surveillance towards mechanistic ecology.

3.4 Pathogen genomics and real-time detection

Long-read metagenomics provides a culture-independent route from clinical specimens to pathogen information, making it an important translational extension of genome-resolved microbial genomics. Its distinctive advantage in this setting is the combination of long molecular reads and real-time nanopore analysis. Reads can be classified during sequencing, allowing pathogen detection and resistance-gene screening to begin before the sequencing run is complete. In lower respiratory infection, Charalampous et al. developed a host-depleted nanopore metagenomic

workflow that produced results within 6 h from sample to result, detected bacterial pathogens with 96.6% sensitivity compared with culture, and accurately detected antibiotic-resistance genes[86]. In infected body fluids, Gu et al. showed that nanopore mNGS coupled with real-time computational analysis enabled pathogen identification with a median 50 min of sequencing and a 6 h sample-to-answer time[87]. More recently, Alcolea-Medina et al. developed a unified host-depletion and nanopore workflow for respiratory samples that detected RNA and DNA viruses, bacteria, atypical bacteria and fungi, generated first automated reports after 30 min of sequencing within a 7 h workflow, and achieved prospective bacterial detection sensitivity and specificity of 90% and 100% after 2 h of sequencing[88]. This study also showed that genome recovery was feasible for a subset of reported organisms after longer sequencing, supporting the potential of clinical long-read metagenomics to move beyond detection toward strain typing and resistance or virulence analysis when pathogen load and read depth are sufficient[88]. These studies indicate that LRS-based metagenomics is most relevant when culture is slow, antibiotic exposure may reduce culture sensitivity, or rapid resistance information is needed. Clinical implementation nevertheless remains constrained by host-background DNA, contamination control, pathogen-load thresholds, database interpretation, genotype-phenotype concordance for AMR, and the need for prospective validation.

4. Extended applications: multi-omics beyond DNA sequences

Although LRS has significantly alleviated the fragmentation limitations inherent to SRS, microbial phenotypes, such as antibiotic resistance, virulence, and

environmental adaptability, are not dictated solely by linear DNA sequences. Epigenetic modifications and dynamic transcriptomic regulation serve as crucial bridges connecting genotype to phenotype. SRS presents inherent limitations in these dimensions. This section systematically elucidates the technical advantages and frontier applications of LRS in microbial epigenomics and full-length transcriptomics (Figure 3).

Figure 3 Paradigm shifts in microbial multi-omics driven by single-molecule sequencing. This schematic contrasts the analytical mechanisms of conventional assays against single-molecule technologies across three core omics dimensions. Epigenomics: Conventional epigenetic profiling necessitates chemical conversions (e.g., bisulfite treatment), which induce severe DNA damage and sequence bias. In contrast, LRS enables the direct detection of diverse native modifications (such as 6mA, 5mC, and 4mC) via nanopore current disruptions. Transcriptomics: The inherent read-length constraints of SRS result in fragmented assemblies, precluding the recovery of contiguous linkage within polycistronic operons. Conversely, LRS achieves full-length transcript identification, capturing contiguous RNA molecules spanning intact multi-gene organizations from transcriptional start sites (TSS) to transcriptional termination sites (TTS).

Alt text: A two-panel schematic comparing conventional and single-molecule multi-omics. It illustrates the shift from chemical-based epigenetics to direct

modification sensing, and from fragmented transcripts to full-length operon sequencing.

4.1 Microbial epigenomics: from native signals to regulatory landscapes

Microbial core epigenetic modifications, predominantly 6mA, 5mC, and 4mC, are fundamental mechanisms governing restriction-modification (R-M) systems, gene expression, and host-pathogen interactions. Historically, SRS relied on bisulfite chemical conversion, which frequently caused DNA degradation and severe GC bias, thus failing to simultaneously detect DNA sequence and native modifications at single-molecule resolution. SRS only captures a population-averaged signal, masking strain-level epigenetic heterogeneity. LRS fundamentally resolves this issue: PacBio SMRT utilizes polymerase kinetic characteristics, while ONT detects nanopore ionic current disruptions. Both platforms accomplish simultaneous DNA sequencing and single-molecule methylation detection without PCR amplification or chemical conversion, enabling direct linkage of epigenetic modifications to their genomic context on individual DNA molecules and overcoming key limitations of SRS-based approaches[89] (Figure 3). Today, LRS is extensively applied to methylation detection, complete R-M system resolution, and the linking of epigenetics to phenotypic regulation. Notably, the two platforms exhibit divergent strengths: PacBio HiFi currently provides the highest sensitivity and confidence for 6mA detection, whereas recent developments including the R10.4.1 pore and improved basecalling models, have drastically improved 5mC and 4mC detection, making it particularly attractive

for cost-effective, large-scale meta-epigenomic studies[90, 91].

Compared with SRS, LRS can associate methylation motifs with cognate methyltransferases across the same long DNA molecules[92]. This precise anchoring allows for the resolution of R-M systems carried by plasmids, revealing their core roles in restriction of HGT and phage defense[93, 94]. Furthermore, LRS maps methylation modifications onto specific functional elements (promoters, operons, and MGEs), identifying potential regulatory sites of single-base modifications on gene expression. In microbial communities, long-read metagenomic methylation profiling improves strain-level resolution and assignment of plasmids and phages to their hosts through shared methylation signatures[95].

However, several challenges remain. Accurate long-read metagenomic methylation analysis depends on the recovery of HMW DNA, which is required to generate long reads and contiguous assemblies that enable reliable assignment of methylation motifs to chromosomes, plasmids, and phages. In addition, the methylation characterization of low-abundance taxa remains limited by sequencing depth. Ding et al. found that at the 25× sequencing depth recommended by PacBio, only 16.24% of phage genomes met this requirement, and the number of detected methylation sites increased with deeper sequencing[96]. Furthermore, different types of methylation modifications exhibit distinct depth requirements. For instance, 40× depth is sufficient to profile most 6mA sites, whereas 4mC resolution requires at least 60× depth to achieve comparable sensitivity[97].

4.2 Full-length transcriptomics: reshaping operons and isoforms

Bacterial transcriptomes are substantially organized around polycistronic operons and contain a rich repertoire of alternative transcripts, antisense RNAs (asRNAs), and non-coding RNAs that collectively coordinate environmental adaptation, stress responses, and virulence. Traditional short-read RNA sequencing often fragments transcripts, complicating accurate reconstruction of complete transcriptional units (Figure 3). LRS can generate reads that span entire RNA molecules in a single pass[94, 98].

A major advantage of LRS is its ability to precisely delineate operon architecture, including TSSs, TTSs, internal promoters and co-transcribed gene clusters. By linking multiple genes within individual polycistronic transcripts, LRS transforms inferred operon models into experimentally validated maps of active transcription units[99, 100]. Furthermore, LRS provides unambiguous information to accurately annotate regulatory elements such as asRNAs, as well as overlapping genes, which typically pose challenges for short‑read‑based assembly[101]. For instance, ONT direct RNA sequencing extended the boundaries of 225 known *E. coli* operons and 89 defined *S. aureus* operons[102]. Moreover, direct RNA sequencing enables the detection of native RNA modifications, such as m5C and m6A, providing new insights into the complexity of post-transcriptional regulation in bacteria[103]. In a community context, integrating metatranscriptomics with nanopore long-read metagenomics enabled the reconstruction of trimethylamine metabolic networks and the identification of key microbial taxa[104]. Nevertheless, applying LRS in the microbiome faces several

technical challenges. Full‑length RNA recovery is difficult due to rapid RNA degradation and inhibitory compounds (e.g., humic acids in soil)[105]. rRNA depletion is another essential step, as rRNA typically constitutes more than 90% of total bacterial RNA[106]. Assignment of transcripts to specific strains represents a third barrier, owing to high sequence similarity among closely related species and the lack of reference genomes[107].

5. Multidimensional technical challenges in LRS

As delineated in preceding sections, LRS has promoted a shift across isolate genomics, metagenomics, and multi-omics dimensions, elevating the analytical resolution of microbiology from fragmented observations to holistic systemic insights. Nevertheless, despite its exceptional potential to reconstruct genomic architectures, the systematic application of LRS in large-scale sequencing studies remains impeded by cost-effectiveness limitations[26, 108]. Crucially, this economic burden extends far beyond direct sequencing expenses (such as reagents and instruments)[109], it is rooted in systemic technical limitations that affect the entire experimental workflow (Table 3), spanning sample preparation, data acquisition, and bioinformatic assembly.

Table 3 Comparison of short- and long-read sequencing platforms

	SRS		LRS		
	Illumina (NovaSeq X)	MGI (DNBSEQ-T20)	PacBio (Revio)	ONT (PromethION 24)	MGI （CycloneSEQ-G400-ER）
DNA input	100 ng	200 ng	500 ng	1 µg	500 ng

requirements					
Read Length	2×150 bp	2×150 bp	15–20 kb	20 bp - >4 Mb	100 bp - >1Mb
Raw accuracy	>99.9%	>99.9%	~99.9%	~99%	~97%
Throughput	2 × (8-10.5)Tb	6 × 12 Tb	4 × (100-120)Gb	24 × (100-200)Gb	1 × 400 Gb
Runtime	48 h	24~30 h	24 h	72 h	72 h

5.1 Sample preparation constraints in LRS

By preserving native nucleic acid information, LRS circumvents the sequence biases and errors introduced by PCR amplification in SRS[110], representing an important advance in genomic research. However, while LRS increases read lengths (Table 3), it simultaneously imposes stringent requirements on upstream sample preparation[26, 111]. This requirement for high purity and intact HMW DNA is a major technical challenge limiting the accessibility of LRS for complex environmental niches.

First, LRS is sensitive to co-purified impurities. In traditional SRS library construction, the PCR amplification process dilutes easily co-precipitated inhibitors, such as polysaccharides and bile acids in gut samples, or residual extraction reagents[112]. Conversely, the amplification-free nature of LRS allows these contaminants to persist in the final library. These impurities inhibit single-molecule polymerase activity or obstruct nanopores, leading to premature termination of the

sequencing reaction[111, 112]. This issue is pronounced in complex environmental samples such as soil, where abundant humic acids, sharing chemical structural similarity with nucleic acids, are readily co-purified. Attempts to remove these impurities through additional purification steps may result in fragmentation of the DNA molecules[113]. Second, the extraction of HMW DNA confronts a fundamental paradox. Even routine extraction procedures, including pipette aspiration, column purification, or mild vortexing, generate physical shear forces to induce the breakage of long DNA fragments[114, 115]. Within microbiome research, this challenge is substantially amplified. Due to the robust peptidoglycan architecture characteristic of many bacteria, gentle enzymatic lysis protocols designed to preserve the DNA backbone fail to lyse Gram-positive bacteria, resulting in bias and loss of taxonomic representation within the community[116]. Conversely, adopting bead-beating methods to ensure comprehensive and unbiased lysis tend to produce fragmented DNA extracts, which limits the advantages of LRS[112].

Finally, low-biomass samples present an additional challenge. For samples characterized by low microbial abundance, the yield from conventional extraction methods may not meet the input requirements of LRS platforms (Table 3)[29, 117]. As demonstrated by Gweon et al. in skin microbiomes and George et al. in environmental soils, standard commercial kits often fail to balance yield and integrity in these niches, recovering only small amounts of fragmented DNA[118, 119]. Determining how to secure adequate extraction recovery rates from low initial biomass while simultaneously preserving DNA length and purity remains a technical

issue in the field.

5.2 Inherent limitations of data throughput and base-calling accuracy

Beyond sample preparation requirements, the limitations of LRS regarding data yield and sequencing depth also restrict its application. Compared to the terabase-scale single run throughput characteristic of SRS platforms, current LRS exhibit a disadvantage in data output per unit cost. For instance, the maximum throughput per flow cell for PacBio and ONT platforms is approximately 100 to 200 Gb, whereas Illumina systems can exceed 8 Tb (Table 3). This throughput limitation causes insufficient resolution for complex samples, which leads to subsequent analytical challenges.

First, this throughput deficit increases the dominance of highly abundant species over low abundance taxa in the microbial communities. In complex habitats, a majority of sequencing reads are originate from dominant microbes. Consequently, due to insufficient overall depth, the probability of capturing species with relative abundances below 1% is substantially reduced[120]. Second, insufficient depth limits the assembly of uncultured microorganisms and leads to information loss. For uncultured taxa lacking reference genomes, the inability to achieve the target coverage requisite for *de novo* assembly makes it difficult to generate high-quality closed genomes or extensive contiguous sequences. Consequently, a massive proportion of the sequencing data remains fragmented, exhibiting low completeness scores upon evaluation with algorithms such as CheckM2, and is ultimately discarded as invalid data during the binning phase[121].

Furthermore, the errors associated with LRS in homopolymer regions also lead to downstream analytical challenges. Constrained by resolution limits of nanopore electrical signals or the kinetic characteristics of single-molecule polymerases, LRS are prone to insertion or deletion errors across consecutive identical bases[111]. Notably, these specific inaccuracies account for over 35% of the total sequencing error profile[122]. In metagenomic samples characterized by low sequencing depth and lacking supplemental error correction from SRS data, these non random errors may induce frameshift mutations within protein coding regions[123, 124]. Such sequence alterations affect accurate prediction of open reading frames and subsequent functional gene annotation.

5.3 Algorithmic burdens in LRS

The convergence of algorithmic architectures and intrinsic sequencing error rates leads to substantial computational demands during the genomic assembly phase, particularly for metagenomes. Although OLC has a higher tolerance for sequencing inaccuracies[125], establishing topological relationships between long reads requires pairwise all-vs-all alignments. This alignment mechanism increases computational overhead; for instance, during the assembly of the HG002 human genome, the alignment stage accounted for approximately 79% of the total execution time[126, 127].

Furthermore, when processing metagenomic samples, contemporary assembly tools generate structural defects despite the allocation of massive computational resources. Evaluations indicate that prominent long-read metagenomic assemblers,

including HiCanu, metaFlye, hifiasm-meta and metaMDBG[128-131], generate an average of over 40 errors per 100 megabases of contiguous sequences. These inaccuracies transcend simple base level substitutions and manifest as complex structural anomalies, such as chimeras and prematurely circularized sequences[132].

6. Current sequencing choices and future perspectives

While recent reviews, such as the work by Agustinho et al., focus primarily on computational workflows and software tools used for long-read data analysis, our review centers on practical applications across biological scales. In this applied context, the optimal choice among SRS, LRS, and hybrid sequencing is now best defined by the biological question, sample complexity, and accuracy requirement. SRS remains widely used in microbial genomics because it provides high-throughput, cost-effective depth, and high per-base accuracy, particularly for taxonomic profiling, abundance estimation, and large cohort studies. However, SRS alone often fail to resolve complete chromosomes, plasmids, long repeats, and complex structural variants. For isolated bacterial genomes, recent PacBio HiFi and ONT R10/R10.4.1 workflows have shown that complete or near-complete chromosomes and plasmids can often be reconstructed without mandatory short-read polishing, provided that coverage, read length, and quality-control metrics are adequate[53, 111, 133, 134]. This does not make hybrid sequencing obsolete. Instead, hybrid approaches remain particularly useful when the study requires maximal consensus accuracy, conservative SNP or indel interpretation, recovery of difficult plasmids, validation of problematic low-coverage, and homopolymer-rich regions[115, 133-135]. In metagenomics,

LRS-only approaches are increasingly powerful for cMAG recovery and structural resolution, but uneven species abundance, rare community members, strain mixtures and assembly artefacts still make short-read supplementation or other orthogonal data valuable in selected settings[63, 64, 130-132, 136, 137].

What is changing most rapidly now is the accuracy. PacBio HiFi sequencing showed that long reads can reach a level of accuracy suitable for both assembly and variant analysis[111]. Subsequent microbial studies showed that complete or near-complete genomes can often be recovered from isolates and microbiomes without mandatory short-read correction[65, 130, 134]. On the ONT side, R10.4.1 chemistry and improved basecalling have narrowed the historical gap in bacterial genome reconstruction, especially at sufficient coverage[133]. The field is therefore moving toward Q30-class long reads as a realistic standard in optimized workflows. Based on this, Q40-grade reads are also becoming plausible[115]. On the other hand, ultra-long reads matter not simply because they are longer, but because they can directly span the regions that still create ambiguity in microbial assembly, such as large repeats, long mobile elements, and extended recombination tracts[138, 139]. Once such regions are bridged by single molecules, some assembly problems no longer need to be solved indirectly through graph inference. Even so, this will depend on more than sequencing chemistry. It will also require reliable extraction of HMW DNA from difficult samples and further reduction of systematic error.

At the same time, LRS is now limited as much by interpretation as by data generation. This is especially clear in metagenomics, where assembly still has to

accommodate extreme abundance variation, microdiversity, shared repeats, and very large datasets. Recent assemblers such as metaMDBG, nanoMDBG, and myloasm show that algorithmic redesign can still improve memory efficiency, runtime, and recovery of circular MAGs, plasmids, and viruses[72, 131, 140]. Binning is evolving in the same way. LorBin and recent cross-platform benchmarks indicate that long-read-aware and hybrid-aware strategies can substantially improve the recovery of high-quality genomes from communities[141, 142]. However, continuity does not guarantee correctness. Long-read metagenome assemblies can still contain chimeras, false duplications, haplotyping artefacts, and premature circularization[132]. This point matters because future tools will need to not only produce longer contigs. but also expose uncertainty, support automated validation, and better distinguish true biological microdiversity from assembly artefact. AI is likely to become increasingly relevant in this domain. Deep learning already underlies modern nanopore basecalling, and recent studies show that model design can strongly affect both accuracy and computational cost[143, 144]. Similar approaches are likely to influence methylation calling, assembly validation, binning, and cross-omic integration. If LRS is to become routine rather than specialist, these analytical advances will also need to be matched by lower cost, more standardized workflows, and greater automation[145].

Recent advances in nanopore protein sequencing have demonstrated its feasibility for single-molecule proteomics, including repeated reading of single polypeptides, single-amino acid sensitivity, and peptide discrimination for protein identification[146, 147]. This platform offers potential advantages over conventional

mass spectrometry, as it does not require enzymatic digestion, enabling the direct characterization of proteoforms and post-translational modifications at single-molecule resolution[146, 148]. Although direct nanopore proteomics has not yet been applied to isolated bacterial strains or complex metagenomic samples, its prospective utility for microbiomics is substantial. In community settings, reading complete polypeptides could resolve the pervasive challenge of peptide sharing, where identical short sequences cannot be uniquely assigned among closely related strains.

The broader significance of LRS may become clearest in multi-omics studies (Figure 4). Its main strength is that it preserves molecular context. That feature can connect genome structure with DNA methylation, transcription, and, indirectly, protein and metabolite profiles. In bacteria, long-read transcriptomics has already shown that transcript organization is more complex than suggested by fragment-based RNA sequencing, revealing extended operons and substantial transcriptional heterogeneity[94]. More recently, nanopore direct RNA sequencing has begun to support joint analysis of the bacterial transcriptome and epitranscriptome within the same experiment[102]. LRS is also becoming increasingly important for epigenomic profiling and transcript-level analysis more broadly[149, 150]. The real opportunity for microbiology lies in combining these measurements with genome-resolved metagenomics, metatranscriptomics, metaproteomics, and metabolomics. Standardized multi-omics studies have already shown that linked analysis of genomes, functions, and metabolites can reveal ecological structure that is invisible to any single modality[151, 152]. In this setting, LRS could serve as the scaffold that anchors

different measurements to the same genome or strain. That would make it easier to ask which lineages are active, which elements are mobilized, which methylation states accompany regulatory change, and how these properties shift over time or under perturbation. In the longer term, such a framework may support models that monitor strain turnover, improve prediction of engraftment or instability, and provide earlier warning of resistance emergence or community collapse. For this reason, the future value of LRS is unlikely to be defined by assembly alone. Its deeper contribution may be to help microbial genomics move from increasingly complete description toward a more mechanistic and predictive framework.

Figure 4 Future perspectives of long-read microbial genomics. (a) Technological and algorithmic evolution. High-accuracy LRS and emerging ultra-long sequencing approaches (>100 kb) are expanding comprehensive resolution across isolate genomics and metagenomics, with hybrid sequencing retained for depth-limited or accuracy-critical applications.. Concurrently, continuous advancements in sequencing platforms are systematically driving raw accuracy towards Q30 and Q40 standards. To efficiently interpret these high-fidelity datasets, AI and deep learning algorithms are optimizing basecalling accuracy, enhancing binning performance, automating assembly validation, and reducing operational costs. (b) Towards multi-omics integration. LRS empowers applications across the epigenome, transcriptome, mobilome, proteome, and metabolome. This holistic integration drives a complete descriptions of genomic architectures, a mechanistic understanding of ecological

interplay, and predictive models for microbial community dynamics.

Alt text: A two-panel conceptual diagram illustrating the future of long-read microbial genomics. Panel (a) shows hybrid and ultra-long sequencing technologies, rising raw accuracy, and AI-driven data optimization for basecalling, binning, and validation. Panel (b) depicts the integration of multiple omics layers to drive complete genomic descriptions, mechanistic understanding, and predictive models for microbial communities.

7. Conclusion

This review provides a systematic evaluation of the application potential and challenges of LRS within microbial genomics. LRS overcomes the inherent limitations of sequence fragmentation and amplification bias. This technological advancement not only facilitates the reconstruction of isolate genomes with gapless completeness and precise localization of genetic elements, but also enhances microbial metagenomics by enabling the recovery of cMAGs, high-resolution strain parsing, and the linkage of MGEs to their specific replicons. Furthermore, LRS extends microbial analysis into multi-omics dimensions by directly detecting epigenetic modifications and capturing full-length transcripts. However, LRS remains constrained by challenges, primarily the requirements for HMW DNA extraction, sequencing depth deficits, and substantial computational burdens. Currently, while high-accuracy LRS is increasingly preferred for isolate genomics and metagenomics,

hybrid sequencing is retained for depth-limited, difficult-replicon, rare-taxon, or accuracy-critical applications. Looking forward, ongoing advancements in sequencing accuracy, efficient bioinformatic tools, and multi-omics integration will further propel LRS to decode global microbial diversity and map the architecture of microbial ecosystems.

Data Availability

Not applicable

List of abbreviations

AMR: antimicrobial resistance; ARG: antibiotic resistance gene; BGCs: biosynthetic gene clusters; cMAGs: circular metagenome-assembled genomes; DNBSEQ: DNA nanoball sequencing; FMT: fecal microbiota transplantation; GC: guanine–cytosine; HGT: horizontal gene transfer; HMW DNA: high molecular weight DNA; ICE: integrative and conjugative elements; IS: insertion sequence; LRS: long-read sequencing; MAG: metagenome-assembled genome; MGEs: mobile genetic elements; OLC: overlap layout consensus; ONT: Oxford Nanopore Technologies; RM: restriction-modification; rRNA: ribosomal RNA; SNVs: single nucleotide variants; SRS: short-read sequencing; SVs: structural variants; TSS: transcription start site; TTS: transcription termination site.

Competing interests

The CycloneSEQ was initially developed by BGI Research and is now being marketed as an advanced technology in MGI. All authors except Yuzheng Gu are employees of BGI Research. The authors declare that this affiliation did not influence the selection, interpretation, or presentation of the literature reviewed in this article.

Funding

This work was supported by the financial support from Shenzhen Science and Technology Program (No. KCXFZ20240903094006009) and Shenzhen Medical Research Fund (No. JCYJ20241202124801003).

Authors' contributions

Xing Rao (Investigation, Writing—original draft, Visualization, Writing—review & editing), Yuzheng Gu (Investigation, Writing—original draft, Visualization, Writing—review & editing), Gabriella (Writing—original draft), Jiayi Ma (Writing—original draft), Haoyu Wang (Investigation, Visualization), and Yuanqiang Zou (Conceptualization, Funding acquisition, Project administration, Supervision, Writing – review & editing).

Acknowledgements

This study is supported by grants from Shenzhen Science and Technology Program (No.SYSPG20241211173845014).

Disclosure of use of AI-assisted tools including generative AI

During the preparation of this work, the authors used Gemini 3.1 Pro to improve the language and readability of the manuscript[153].

Reference

1. Sanger F, Nicklen S, Coulson AR. DNA sequencing with chain-terminating inhibitors. *Proc Natl Acad Sci U S A*. 1977; doi: 10.1073/pnas.74.12.5463.
2. Fleischmann RD, Adams MD, White O, et al. Whole-genome random sequencing and assembly of Haemophilus influenzae Rd. *Science*. 1995; doi: 10.1126/science.7542800.
3. van Dijk EL, Auger H, Jaszczyszyn Y, et al. Ten years of next-generation sequencing technology. *Trends Genet*. 2014; doi: 10.1016/j.tig.2014.07.001.
4. Mak SST, Gopalakrishnan S, Caroe C, et al. Comparative performance of the BGISEQ-500 vs Illumina HiSeq2500 sequencing platforms for palaeogenomic sequencing. *Gigascience*. 2017; doi: 10.1093/gigascience/gix049.
5. Previte MJ, Zhou C, Kellinger M, et al. DNA sequencing using polymerase substrate-binding kinetics. *Nat Commun*. 2015; doi: 10.1038/ncomms6936.
6. Human Microbiome Project C. A framework for human microbiome research. *Nature*. 2012; doi: 10.1038/nature11209.
7. Qin J, Li R, Raes J, et al. A human gut microbial gene catalogue established by metagenomic sequencing. *Nature*. 2010; doi: 10.1038/nature08821.
8. Mandelker D, Schmidt RJ, Ankala A, et al. Navigating highly homologous genes in a molecular diagnostic setting: a resource for clinical next-generation sequencing. *Genet Med*. 2016; doi: 10.1038/gim.2016.58.
9. Korlach J, Bjornson KP, Chaudhuri BP, et al. Real-time DNA sequencing from single polymerase molecules. *Methods Enzymol*. 2010; doi: 10.1016/S0076-6879(10)72001-2.
10. Shafin K, Pesout T, Lorig-Roach R, et al. Nanopore sequencing and the Shasta toolkit enable efficient de novo assembly of eleven human genomes. *Nat Biotechnol*. 2020; doi: 10.1038/s41587-020-0503-6.
11. Liang H, Zou Y, Wang M, et al. Efficiently constructing complete genomes with CycloneSEQ to fill gaps in bacterial draft assemblies. *GigaByte*. 2025; doi: 10.46471/gigabyte.154.
12. Quick J, Loman NJ, Duraffour S, et al. Real-time, portable genome sequencing for Ebola surveillance. *Nature*. 2016; doi: 10.1038/nature16996.
13. Loman NJ, Quick J, Simpson JT. A complete bacterial genome assembled de novo using only nanopore sequencing data. *Nat Methods*. 2015; doi: 10.1038/nmeth.3444.
14. Quick J, Ashton P, Calus S, et al. Rapid draft sequencing and real-time

nanopore sequencing in a hospital outbreak of Salmonella. *Genome Biol*. 2015; doi: 10.1186/s13059-015-0677-2.
15. Mrazek J, Guo X, Shah A. Simple sequence repeats in prokaryotic genomes. *Proc Natl Acad Sci U S A*. 2007; doi: 10.1073/pnas.0702412104.
16. Brazda V, Fojta M, Bowater RP. Structures and stability of simple DNA repeats from bacteria. *Biochem J*. 2020; doi: 10.1042/BCJ20190703.
17. Schmid M, Frei D, Patrignani A, et al. Pushing the limits of de novo genome assembly for complex prokaryotic genomes harboring very long, near identical repeats. *Nucleic Acids Res*. 2018; doi: 10.1093/nar/gky726.
18. Achaz G, Rocha EP, Netter P, et al. Origin and fate of repeats in bacteria. *Nucleic Acids Res*. 2002; doi: 10.1093/nar/gkf391.
19. Hill CW. Large genomic sequence repetitions in bacteria: lessons from rRNA operons and Rhs elements. *Res Microbiol*. 1999; doi: 10.1016/s0923-2508(99)00125-4.
20. Huisman JS, Vaughan TG, Egli A, et al. The effect of sequencing and assembly on the inference of horizontal gene transfer on chromosomal and plasmid phylogenies. *Philos Trans R Soc Lond B Biol Sci*. 2022; doi: 10.1098/rstb.2021.0245.
21. George S, Pankhurst L, Hubbard A, et al. Resolving plasmid structures in Enterobacteriaceae using the MinION nanopore sequencer: assessment of MinION and MinION/Illumina hybrid data assembly approaches. *Microb Genom*. 2017; doi: 10.1099/mgen.0.000118.
22. Wirbel J, Hickey AS, Chang D, et al. Long-read metagenomics reveals phage dynamics in the human gut microbiome. *Nature*. 2026; doi: 10.1038/s41586-025-09786-2.
23. Goldstein S, Beka L, Graf J, et al. Evaluation of strategies for the assembly of diverse bacterial genomes using MinION long-read sequencing. *BMC Genomics*. 2019; doi: 10.1186/s12864-018-5381-7.
24. Tilak MK, Botero-Castro F, Galtier N, et al. Illumina Library Preparation for Sequencing the GC-Rich Fraction of Heterogeneous Genomic DNA. *Genome Biol Evol*. 2018; doi: 10.1093/gbe/evy022.
25. International Chicken Genome Sequencing C. Sequence and comparative analysis of the chicken genome provide unique perspectives on vertebrate evolution. *Nature*. 2004; doi: 10.1038/nature03154.
26. Logsdon GA, Vollger MR, Eichler EE. Long-read human genome sequencing and its applications. *Nat Rev Genet*. 2020; doi: 10.1038/s41576-020-0236-x.
27. Wang Y, Zhao Y, Bollas A, et al. Nanopore sequencing technology, bioinformatics and applications. *Nat Biotechnol*. 2021; doi: 10.1038/s41587-021-01108-x.
28. Marx V. Method of the year: long-read sequencing. *Nat Methods*. 2023; doi: 10.1038/s41592-022-01730-w.
29. Agustinho DP, Fu Y, Menon VK, et al. Unveiling microbial diversity: harnessing long-read sequencing technology. *Nat Methods*. 2024; doi: 10.1038/s41592-024-02262-1.

30. Fraser CM, Eisen JA, Nelson KE, et al. The value of complete microbial genome sequencing (you get what you pay for). *J Bacteriol*. 2002; doi: 10.1128/JB.184.23.6403-6405.2002.
31. Larsen MV, Cosentino S, Rasmussen S, et al. Multilocus sequence typing of total-genome-sequenced bacteria. *J Clin Microbiol*. 2012; doi: 10.1128/JCM.06094-11.
32. Shi W, Sun Q, Fan G, et al. gcType: a high-quality type strain genome database for microbial phylogenetic and functional research. *Nucleic Acids Res*. 2021; doi: 10.1093/nar/gkaa957.
33. Eisenhofer R, Odriozola I, Alberdi A. Impact of microbial genome completeness on metagenomic functional inference. *ISME Commun*. 2023; doi: 10.1038/s43705-023-00221-z.
34. Zou Y, Xue W, Luo G, et al. 1,520 reference genomes from cultivated human gut bacteria enable functional microbiome analyses. *Nat Biotechnol*. 2019; doi: 10.1038/s41587-018-0008-8.
35. Akintunde O, Tucker T, Carabetta VJ. The Evolution of Next-Generation Sequencing Technologies. *Methods Mol Biol*. 2025; doi: 10.1007/978-1-0716-4192-7_1.
36. Peng K, Yin Y, Li Y, et al. QitanTech Nanopore Long-Read Sequencing Enables Rapid Resolution of Complete Genomes of Multi-Drug Resistant Pathogens. *Front Microbiol*. 2022; doi: 10.3389/fmicb.2022.778659.
37. Chin FY, Leung HC, Yiu SM. Sequence assembly using next generation sequencing data--challenges and solutions. *Sci China Life Sci*. 2014; doi: 10.1007/s11427-014-4752-9.
38. Utturkar SM, Klingeman DM, Hurt RA, Jr., et al. A Case Study into Microbial Genome Assembly Gap Sequences and Finishing Strategies. *Front Microbiol*. 2017; doi: 10.3389/fmicb.2017.01272.
39. Kim C, Pongpanich M, Porntaveetus T. Unraveling metagenomics through long-read sequencing: a comprehensive review. *J Transl Med*. 2024; doi: 10.1186/s12967-024-04917-1.
40. Athanasopoulou K, Boti MA, Adamopoulos PG, et al. Third-Generation Sequencing: The Spearhead towards the Radical Transformation of Modern Genomics. *Life (Basel)*. 2021; doi: 10.3390/life12010030.
41. Chin CS, Alexander DH, Marks P, et al. Nonhybrid, finished microbial genome assemblies from long-read SMRT sequencing data. *Nat Methods*. 2013; doi: 10.1038/nmeth.2474.
42. Koren S, Harhay GP, Smith TP, et al. Reducing assembly complexity of microbial genomes with single-molecule sequencing. *Genome Biol*. 2013; doi: 10.1186/gb-2013-14-9-r101.
43. Stanton NCKHJ-AL. Next-generation DNA sequencing of oral microbes at the Sir John Walsh Research Institute: technologies, tools and achievements. *Journal of the Royal Society of New Zealand*. 2019; doi: 10.1080/03036758.2019.1687530.
44. Marin-Gordillo A, Lopez-Garcia E, Romero-Gil V, et al. Hybrid genome

assembly and comprehensive genomic analysis of Lactiplantibacillus pentosus LP309 reveal its probiotic and technological potential. *BMC Genomics*. 2026; doi: 10.1186/s12864-026-12678-y.
45. Wang H, Gu Y, He W, et al. Complete Genomes of Cultivated Gut Bacteria Reveal Mobile Genetic Element-Driven Functional Diversity with Therapeutic Implications. *bioRxiv*. 2026; doi: 10.64898/2026.03.25.714112.
46. Gu Y, Wang H, Yang J, et al. Complete genome-derived metabolic interactions reveal impact of gut ecology on human health. *bioRxiv*. 2026; doi: 10.64898/2026.03.25.714329.
47. Carr VR, Shkoporov A, Hill C, et al. Probing the Mobilome: Discoveries in the Dynamic Microbiome. *Trends Microbiol*. 2021; doi: 10.1016/j.tim.2020.05.003.
48. . !!! INVALID CITATION !!! [45].
49. Arredondo-Alonso S, Willems RJ, van Schaik W, et al. On the (im)possibility of reconstructing plasmids from whole-genome short-read sequencing data. *Microb Genom*. 2017; doi: 10.1099/mgen.0.000128.
50. Martinez JL, Coque TM, Lanza VF, et al. Genomic and metagenomic technologies to explore the antibiotic resistance mobilome. *Ann N Y Acad Sci*. 2017; doi: 10.1111/nyas.13282.
51. Olsen NS, Riber L. Metagenomics as a Transformative Tool for Antibiotic Resistance Surveillance: Highlighting the Impact of Mobile Genetic Elements with a Focus on the Complex Role of Phages. *Antibiotics (Basel)*. 2025; doi: 10.3390/antibiotics14030296.
52. Jiang Y, Wang Y, Hua X, et al. Pooled Plasmid Sequencing Reveals the Relationship Between Mobile Genetic Elements and Antimicrobial Resistance Genes in Clinically Isolated Klebsiella pneumoniae. *Genomics Proteomics Bioinformatics*. 2020; doi: 10.1016/j.gpb.2020.12.002.
53. Zhao W, Zeng W, Pang B, et al. Oxford nanopore long-read sequencing enables the generation of complete bacterial and plasmid genomes without short-read sequencing. *Front Microbiol*. 2023; doi: 10.3389/fmicb.2023.1179966.
54. McLeod A, Fagerlund A, Rud I, et al. Large Plasmid Complement Resolved: Complete Genome Sequencing of Lactobacillus plantarum MF1298, a Candidate Probiotic Strain Associated with Unfavorable Effect. *Microorganisms*. 2019; doi: 10.3390/microorganisms7080262.
55. Cottingham H, Judd LM, Harshegyi-Hand T, et al. Nanopore sequencing enables highly accurate genotyping and identification of resistance determinants in key nosocomial pathogens. *Microb Genom*. 2026; doi: 10.1099/mgen.0.001657.
56. Wick RR, Judd LM, Stinear TP, et al. Are reads required? High-precision variant calling from bacterial genome assemblies. *Access Microbiol*. 2025; doi: 10.1099/acmi.0.001025.v3.
57. Waters EV, Tucker LA, Ahmed JK, et al. Impact of Salmonella genome rearrangement on gene expression. *Evol Lett*. 2022; doi: 10.1002/evl3.305.

58. Bouda E, Peng Y, Williams MM, et al. Bordetella pertussis exhibits genomic diversity within patients and laboratory culture. *mSystems*. 2025; doi: 10.1128/msystems.01717-24.
59. Li C, Tyson GH, Hsu CH, et al. Long-Read Sequencing Reveals Evolution and Acquisition of Antimicrobial Resistance and Virulence Genes in Salmonella enterica. *Front Microbiol*. 2021; doi: 10.3389/fmicb.2021.777817.
60. Canalda-Baltrons A, Silcocks M, Hall MB, et al. Genome graphs reveal the importance of structural variation in Mycobacterium tuberculosis evolution and drug resistance. *Nat Commun*. 2025; doi: 10.1038/s41467-025-65779-9.
61. Sheppard AE, Stoesser N, Wilson DJ, et al. Nested Russian Doll-Like Genetic Mobility Drives Rapid Dissemination of the Carbapenem Resistance Gene blaKPC. *Antimicrob Agents Chemother*. 2016; doi: 10.1128/AAC.00464-16.
62. Colombini L, Santoro F, Tirziu M, et al. The mobilome of Lactobacillus crispatus M247 includes two novel genetic elements: Tn7088 coding for a putative bacteriocin and the siphovirus prophage PhiM247. *Microb Genom*. 2023; doi: 10.1099/mgen.0.001150.
63. Benler S, Faure G, Altae-Tran H, et al. Cargo Genes of Tn7-Like Transposons Comprise an Enormous Diversity of Defense Systems, Mobile Genetic Elements, and Antibiotic Resistance Genes. *mBio*. 2021; doi: 10.1128/mBio.02938-21.
64. Brown CL, Keenum IM, Dai D, et al. Critical evaluation of short, long, and hybrid assembly for contextual analysis of antibiotic resistance genes in complex environmental metagenomes. *Sci Rep*. 2021; doi: 10.1038/s41598-021-83081-8.
65. Kim CY, Ma J, Lee I. HiFi metagenomic sequencing enables assembly of accurate and complete genomes from human gut microbiota. *Nat Commun*. 2022; doi: 10.1038/s41467-022-34149-0.
66. Gounot JS, Chia M, Bertrand D, et al. Genome-centric analysis of short and long read metagenomes reveals uncharacterized microbiome diversity in Southeast Asians. *Nat Commun*. 2022; doi: 10.1038/s41467-022-33782-z.
67. Moss EL, Maghini DG, Bhatt AS. Complete, closed bacterial genomes from microbiomes using nanopore sequencing. *Nat Biotechnol*. 2020; doi: 10.1038/s41587-020-0422-6.
68. Singleton CM, Petriglieri F, Kristensen JM, et al. Connecting structure to function with the recovery of over 1000 high-quality metagenome-assembled genomes from activated sludge using long-read sequencing. *Nat Commun*. 2021; doi: 10.1038/s41467-021-22203-2.
69. Sereika M, Mussig AJ, Jiang C, et al. Genome-resolved long-read sequencing expands known microbial diversity across terrestrial habitats. *Nat Microbiol*. 2025; doi: 10.1038/s41564-025-02062-z.
70. Chen L, Zhao N, Cao J, et al. Short- and long-read metagenomics expand individualized structural variations in gut microbiomes. *Nat Commun*. 2022; doi: 10.1038/s41467-022-30857-9.
71. Bickhart DM, Kolmogorov M, Tseng E, et al. Generating lineage-resolved,

complete metagenome-assembled genomes from complex microbial communities. *Nat Biotechnol*. 2022; doi: 10.1038/s41587-021-01130-z.

72. Shaw J, Marin MG, Li H. High-resolution metagenome assembly for modern long reads with myloasm. *Nat Biotechnol*. 2026; doi: 10.1038/s41587-026-03053-z.

73. Fan Y, Ni M, Aggarwala V, et al. Long-read metagenomics for strain tracking after faecal microbiota transplant. *Nat Microbiol*. 2025; doi: 10.1038/s41564-025-02164-8.

74. Minich JJ, Allsing N, Din MO, et al. Culture-independent meta-pangenomics enabled by long-read metagenomics reveals associations with pediatric undernutrition. *Cell*. 2025; doi: 10.1016/j.cell.2025.08.020.

75. Bickhart DM, Watson M, Koren S, et al. Assignment of virus and antimicrobial resistance genes to microbial hosts in a complex microbial community by combined long-read assembly and proximity ligation. *Genome Biol*. 2019; doi: 10.1186/s13059-019-1760-x.

76. Yahara K, Suzuki M, Hirabayashi A, et al. Long-read metagenomics using PromethION uncovers oral bacteriophages and their interaction with host bacteria. *Nat Commun*. 2021; doi: 10.1038/s41467-020-20199-9.

77. Antipov D, Rayko M, Kolmogorov M, et al. viralFlye: assembling viruses and identifying their hosts from long-read metagenomics data. *Genome Biol*. 2022; doi: 10.1186/s13059-021-02566-x.

78. Beaulaurier J, Zhu S, Deikus G, et al. Metagenomic binning and association of plasmids with bacterial host genomes using DNA methylation. *Nat Biotechnol*. 2018; doi: 10.1038/nbt.4037.

79. Suzuki Y, Nishijima S, Furuta Y, et al. Long-read metagenomic exploration of extrachromosomal mobile genetic elements in the human gut. *Microbiome*. 2019; doi: 10.1186/s40168-019-0737-z.

80. Kiguchi Y, Hamamoto N, Kashima Y, et al. Giant extrachromosomal element "Inocle" potentially expands the adaptive capacity of the human oral microbiome. *Nat Commun*. 2025; doi: 10.1038/s41467-025-62406-5.

81. Dai D, Brown C, Burgmann H, et al. Long-read metagenomic sequencing reveals shifts in associations of antibiotic resistance genes with mobile genetic elements from sewage to activated sludge. *Microbiome*. 2022; doi: 10.1186/s40168-021-01216-5.

82. Ding J, Wang M, Xu X, et al. Highly Resolved Community Sewage Metagenomics Unveiling Landscape and Transmission Patterns of Antibiotic Resistome in Hong Kong Populations. *Adv Sci (Weinh)*. 2026; doi: 10.1002/advs.202508389.

83. Mao X, Yin X, Yang Y, et al. Longitudinal metagenomic analysis on antibiotic resistome, mobilome, and microbiome of river ecosystems in a sub-tropical metropolitan city. *Water Res*. 2025; doi: 10.1016/j.watres.2025.123102.

84. Rolbiecki D, Paukszto L, Krawczyk K, et al. Chlorine disinfection modifies the microbiome, resistome and mobilome of hospital wastewater - A nanopore long-read metagenomic approach. *J Hazard Mater*. 2023; doi:

10.1016/j.jhazmat.2023.132298.

85. Zhang Q, Fan Y, Qian X, et al. Unraveling the role of microplastics in antibiotic resistance: Insights from long-read metagenomics on ARG mobility and host dynamics. *J Hazard Mater*. 2025; doi: 10.1016/j.jhazmat.2025.137804.

86. Charalampous T, Kay GL, Richardson H, et al. Nanopore metagenomics enables rapid clinical diagnosis of bacterial lower respiratory infection. *Nat Biotechnol*. 2019; doi: 10.1038/s41587-019-0156-5.

87. Gu W, Deng X, Lee M, et al. Rapid pathogen detection by metagenomic next-generation sequencing of infected body fluids. *Nat Med*. 2021; doi: 10.1038/s41591-020-1105-z.

88. Alcolea-Medina A, Alder C, Snell LB, et al. Unified metagenomic method for rapid detection of microorganisms in clinical samples. *Commun Med (Lond)*. 2024; doi: 10.1038/s43856-024-00554-3.

89. Lucas MC, Novoa EM. Long-read sequencing in the era of epigenomics and epitranscriptomics. *Nat Methods*. 2023; doi: 10.1038/s41592-022-01724-8.

90. Jakubickova M, Sabatova K, Zbudilova M, et al. MethylomeMiner: A novel tool for high-resolution analysis of bacterial methylation patterns from nanopore sequencing. *Comput Struct Biotechnol J*. 2025; doi: 10.1016/j.csbj.2025.10.047.

91. Tourancheau A, Mead EA, Zhang XS, et al. Discovering multiple types of DNA methylation from bacteria and microbiome using nanopore sequencing. *Nat Methods*. 2021; doi: 10.1038/s41592-021-01109-3.

92. Sigurpalsdottir BD, Stefansson OA, Holley G, et al. A comparison of methods for detecting DNA methylation from long-read sequencing of human genomes. *Genome Biol*. 2024; doi: 10.1186/s13059-024-03207-9.

93. Nass NM, Zaher KA. From Methylomes to CRISPR Epigenetic Editing: New Paths in Antibiotic Resistance. *Pathogens*. 2025; doi: 10.3390/pathogens14121267.

94. Yan B, Boitano M, Clark TA, et al. SMRT-Cappable-seq reveals complex operon variants in bacteria. *Nat Commun*. 2018; doi: 10.1038/s41467-018-05997-6.

95. Fu Y, Timp W, Sedlazeck FJ. Computational analysis of DNA methylation from long-read sequencing. *Nat Rev Genet*. 2025; doi: 10.1038/s41576-025-00822-5.

96. Ding Y, Zhao L, Wang G, et al. PacBio sequencing of human fecal samples uncovers the DNA methylation landscape of 22 673 gut phages. *Nucleic Acids Res*. 2023; doi: 10.1093/nar/gkad977.

97. Wilbanks EG, Dore H, Ashby MH, et al. Metagenomic methylation patterns resolve bacterial genomes of unusual size and structural complexity. *ISME J*. 2022; doi: 10.1038/s41396-022-01242-7.

98. Rodger G, Lipworth S, Barrett L, et al. Comparison of direct cDNA and PCR-cDNA Nanopore sequencing of RNA from Escherichia coli isolates. *Microb Genom*. 2024; doi: 10.1099/mgen.0.001296.

99. Conte MI, Fuentes-Trillo A, Dominguez Conde C. Opportunities and tradeoffs in single-cell transcriptomic technologies. *Trends Genet*. 2024; doi: 10.1016/j.tig.2023.10.003.
100. Wen L, Tang F. Single-cell omics sequencing technologies: the long-read generation. *Trends Genet*. 2026; doi: 10.1016/j.tig.2025.07.012.
101. Lim CS, Gibbon AK, Tran Nguyen AT, et al. RIBOSS detects novel translational events by combining long- and short-read transcriptome and translatome profiling. *Brief Bioinform*. 2025; doi: 10.1093/bib/bbaf164.
102. Tan L, Guo Z, Shao Y, et al. Analysis of bacterial transcriptome and epitranscriptome using nanopore direct RNA sequencing. *Nucleic Acids Res*. 2024; doi: 10.1093/nar/gkae601.
103. Riquelme-Barrios S, Vasquez-Camus L, Cusack SA, et al. Direct RNA sequencing of the Escherichia coli epitranscriptome uncovers alterations under heat stress. *Nucleic Acids Res*. 2025; doi: 10.1093/nar/gkaf175.
104. Simo C, Mamani-Huanca M, Hernandez-Hernandez O, et al. Application of nanopore long-read sequencing and metabolomics in an in vitro dynamic intestinal digestion model: A genome-centric metatranscriptomic approach to investigating microbial TMA and SCFA metabolism. *J Pharm Biomed Anal*. 2025; doi: 10.1016/j.jpba.2025.116896.
105. Barber DG, Davies CA, Hartley IP, et al. Evaluation of commercial RNA extraction kits for long-read metatranscriptomics in soil. *Microb Genom*. 2024; doi: 10.1099/mgen.0.001298.
106. Culviner PH, Guegler CK, Laub MT. A Simple, Cost-Effective, and Robust Method for rRNA Depletion in RNA-Sequencing Studies. *mBio*. 2020; doi: 10.1128/mBio.00010-20.
107. Zhang W, Li XJ, Liu F, et al. Fungen: clustering and correcting long-read metatranscriptomic data for exploring eukaryotic microorganisms. *Sci China Life Sci*. 2026; doi: 10.1007/s11427-024-2853-x.
108. Kolmogorov M, Billingsley KJ, Mastoras M, et al. Scalable Nanopore sequencing of human genomes provides a comprehensive view of haplotype-resolved variation and methylation. *Nat Methods*. 2023; doi: 10.1038/s41592-023-01993-x.
109. Giordano F, Aigrain L, Quail MA, et al. De novo yeast genome assemblies from MinION, PacBio and MiSeq platforms. *Sci Rep*. 2017; doi: 10.1038/s41598-017-03996-z.
110. Kozarewa I, Ning Z, Quail MA, et al. Amplification-free Illumina sequencing-library preparation facilitates improved mapping and assembly of (G+C)-biased genomes. *Nat Methods*. 2009; doi: 10.1038/nmeth.1311.
111. Wenger AM, Peluso P, Rowell WJ, et al. Accurate circular consensus long-read sequencing improves variant detection and assembly of a human genome. *Nat Biotechnol*. 2019; doi: 10.1038/s41587-019-0217-9.
112. Maghini DG, Moss EL, Vance SE, et al. Improved high-molecular-weight DNA extraction, nanopore sequencing and metagenomic assembly from the human gut microbiome. *Nat Protoc*. 2021; doi: 10.1038/s41596-020-00424-x.

113. Gand M, Bloemen B, Vanneste K, et al. Comparison of 6 DNA extraction methods for isolation of high yield of high molecular weight DNA suitable for shotgun metagenomics Nanopore sequencing to detect bacteria. *BMC Genomics*. 2023; doi: 10.1186/s12864-023-09537-5.
114. Jain M, Koren S, Miga KH, et al. Nanopore sequencing and assembly of a human genome with ultra-long reads. *Nat Biotechnol*. 2018; doi: 10.1038/nbt.4060.
115. Wick RR, Judd LM, Holt KE. Assembling the perfect bacterial genome using Oxford Nanopore and Illumina sequencing. *PLoS Comput Biol*. 2023; doi: 10.1371/journal.pcbi.1010905.
116. Costea PI, Zeller G, Sunagawa S, et al. Towards standards for human fecal sample processing in metagenomic studies. *Nat Biotechnol*. 2017; doi: 10.1038/nbt.3960.
117. De Coster W, Weissensteiner MH, Sedlazeck FJ. Towards population-scale long-read sequencing. *Nat Rev Genet*. 2021; doi: 10.1038/s41576-021-00367-3.
118. Serghiou IR, Baker D, Evans R, et al. An efficient method for high molecular weight bacterial DNA extraction suitable for shotgun metagenomics from skin swabs. *Microb Genom*. 2023; doi: 10.1099/mgen.0.001058.
119. Child HT, Wierzbicki L, Joslin GR, et al. Comparative evaluation of soil DNA extraction kits for long read metagenomic sequencing. *Access Microbiol*. 2024; doi: 10.1099/acmi.0.000868.v3.
120. Slizovskiy IB, Oliva M, Settle JK, et al. Target-enriched long-read sequencing (TELSeq) contextualizes antimicrobial resistance genes in metagenomes. *Microbiome*. 2022; doi: 10.1186/s40168-022-01368-y.
121. Chklovski A, Parks DH, Woodcroft BJ, et al. CheckM2: a rapid, scalable and accurate tool for assessing microbial genome quality using machine learning. *Nat Methods*. 2023; doi: 10.1038/s41592-023-01940-w.
122. Weirather JL, de Cesare M, Wang Y, et al. Comprehensive comparison of Pacific Biosciences and Oxford Nanopore Technologies and their applications to transcriptome analysis. *F1000Res*. 2017; doi: 10.12688/f1000research.10571.2.
123. Watson M, Warr A. Errors in long-read assemblies can critically affect protein prediction. *Nat Biotechnol*. 2019; doi: 10.1038/s41587-018-0004-z.
124. Pop M, Salzberg SL. Bioinformatics challenges of new sequencing technology. *Trends Genet*. 2008; doi: 10.1016/j.tig.2007.12.006.
125. Koren S, Walenz BP, Berlin K, et al. Canu: scalable and accurate long-read assembly via adaptive k-mer weighting and repeat separation. *Genome Res*. 2017; doi: 10.1101/gr.215087.116.
126. Berlin K, Koren S, Chin CS, et al. Assembling large genomes with single-molecule sequencing and locality-sensitive hashing. *Nat Biotechnol*. 2015; doi: 10.1038/nbt.3238.
127. Rautiainen M, Nurk S, Walenz BP, et al. Telomere-to-telomere assembly of diploid chromosomes with Verkko. *Nat Biotechnol*. 2023; doi:

10.1038/s41587-023-01662-6.

128. Nurk S, Walenz BP, Rhie A, et al. HiCanu: accurate assembly of segmental duplications, satellites, and allelic variants from high-fidelity long reads. *Genome Res*. 2020; doi: 10.1101/gr.263566.120.

129. Kolmogorov M, Bickhart DM, Behsaz B, et al. metaFlye: scalable long-read metagenome assembly using repeat graphs. *Nat Methods*. 2020; doi: 10.1038/s41592-020-00971-x.

130. Feng X, Cheng H, Portik D, et al. Metagenome assembly of high-fidelity long reads with hifiasm-meta. *Nat Methods*. 2022; doi: 10.1038/s41592-022-01478-3.

131. Benoit G, Raguideau S, James R, et al. High-quality metagenome assembly from long accurate reads with metaMDBG. *Nat Biotechnol*. 2024; doi: 10.1038/s41587-023-01983-6.

132. Trigodet F, Sachdeva R, Banfield JF, et al. Troubleshooting common errors in assemblies of long-read metagenomes. *Nat Biotechnol*. 2026; doi: 10.1038/s41587-025-02971-8.

133. Sanderson ND, Hopkins KMV, Colpus M, et al. Evaluation of the accuracy of bacterial genome reconstruction with Oxford Nanopore R10.4.1 long-read-only sequencing. *Microb Genom*. 2024; doi: 10.1099/mgen.0.001246.

134. Sereika M, Kirkegaard RH, Karst SM, et al. Oxford Nanopore R10.4 long-read sequencing enables the generation of near-finished bacterial genomes from pure cultures and metagenomes without short-read or reference polishing. *Nat Methods*. 2022; doi: 10.1038/s41592-022-01539-7.

135. Wick RR, Judd LM, Cerdeira LT, et al. Trycycler: consensus long-read assemblies for bacterial genomes. *Genome Biol*. 2021; doi: 10.1186/s13059-021-02483-z.

136. Bertrand D, Shaw J, Kalathiyappan M, et al. Hybrid metagenomic assembly enables high-resolution analysis of resistance determinants and mobile elements in human microbiomes. *Nat Biotechnol*. 2019; doi: 10.1038/s41587-019-0191-2.

137. Jin H, You L, Zhao F, et al. Hybrid, ultra-deep metagenomic sequencing enables genomic and functional characterization of low-abundance species in the human gut microbiome. *Gut Microbes*. 2022; doi: 10.1080/19490976.2021.2021790.

138. Jain C, Rodriguez RL, Phillippy AM, et al. High throughput ANI analysis of 90K prokaryotic genomes reveals clear species boundaries. *Nat Commun*. 2018; doi: 10.1038/s41467-018-07641-9.

139. Koren S, Bao Z, Guarracino A, et al. Gapless assembly of complete human and plant chromosomes using only nanopore sequencing. *Genome Res*. 2024; doi: 10.1101/gr.279334.124.

140. Benoit G, James R, Raguideau S, et al. High-quality metagenome assembly from nanopore reads with nanoMDBG. *Nat Commun*. 2026; doi: 10.1038/s41467-026-69760-y.

141. Xue W, Liu Z, Zhang Y, et al. LorBin: efficient binning of long-read metagenomes by multiscale adaptive clustering and evaluation. *Nat Commun*. 2025; doi: 10.1038/s41467-025-64916-8.
142. Han H, Wang Z, Zhu S. Benchmarking metagenomic binning tools on real datasets across sequencing platforms and binning modes. *Nat Commun*. 2025; doi: 10.1038/s41467-025-57957-6.
143. Pages-Gallego M, de Ridder J. Comprehensive benchmark and architectural analysis of deep learning models for nanopore sequencing basecalling. *Genome Biol*. 2023; doi: 10.1186/s13059-023-02903-2.
144. Singh G, Alser M, Denolf K, et al. RUBICON: a framework for designing efficient deep learning-based genomic basecallers. *Genome Biol*. 2024; doi: 10.1186/s13059-024-03181-2.
145. Child HT, Wierzbicki L, Joslin GR, et al. Automated environmental metagenomics using Oxford nanopore sequencing. *BMC Genomics*. 2025; doi: 10.1186/s12864-025-11989-w.
146. Motone K, Kontogiorgos-Heintz D, Wee J, et al. Multi-pass, single-molecule nanopore reading of long protein strands. *Nature*. 2024; doi: 10.1038/s41586-024-07935-7.
147. Wang J, Chen J, Pan H, et al. Nanopore-based massively parallel sensing for peptide profiling and protein identification. *Nat Commun*. 2026; doi: 10.1038/s41467-026-69628-1.
148. Liu X, Dong Z, Timp G. Calling the amino acid sequence of a protein/peptide from the nanospectrum produced by a sub-nanometer diameter pore. *Sci Rep*. 2022; doi: 10.1038/s41598-022-22305-x.
149. Liu T, Conesa A. Profiling the epigenome using long-read sequencing. *Nat Genet*. 2025; doi: 10.1038/s41588-024-02038-5.
150. Monzo C, Liu T, Conesa A. Transcriptomics in the era of long-read sequencing. *Nat Rev Genet*. 2025; doi: 10.1038/s41576-025-00828-z.
151. Shaffer JP, Nothias LF, Thompson LR, et al. Standardized multi-omics of Earth's microbiomes reveals microbial and metabolite diversity. *Nat Microbiol*. 2022; doi: 10.1038/s41564-022-01266-x.
152. Go D, Yeon GH, Park SJ, et al. Integration of metabolomics and other omics: from microbes to microbiome. *Appl Microbiol Biotechnol*. 2024; doi: 10.1007/s00253-024-13384-z.
153. Google (2026). Gemini (3.0 Pro) [Large language model]. Response to query made by Xing Rao, Yuzheng Gu, Gabriella, Jiayi Ma. 04/2026. https://gemini.google.com

Table 1. Studies demonstrating the implementation of LRS for single-isolate microbial genome reconstruction (2013–2026)

**Year**	**Study**	**Platform**	**Isolate(s) / dataset**	**Main contribution to genome quality**

		**strategy**		
2013	Koren et al.	PacBio RS	6 bacterial genomes + 2,267 reference genomes	LRS reduce assembly complexity, showing over 70% of known bacterial and archaeal genomes could be finished using a single PacBio library
2013	Chin et al.	PacBio RS SMRT	*Escherichia coli* K-12 MG1655, *Meiothermus ruber* DSM1279, *Pedobacter heparinus* DSM2366	Demonstrated non-hybrid finishing from a single long-insert library improved resolution of long repeats, and consensus accuracy exceeding 99.999%
2015	Loman et al.	ONT MinION	*Escherichia coli* K-12 MG1655	Produced a single 4.6 Mb contig with 99.5% nucleotide identity, providing early proof that nanopore-only data could recover complete chromosome structure
2017	Wick et al.	ONT MinION + Illumina HiSeq	12 *Klebsiella pneumoniae* isolates	Multiplex MinION sequencing with hybrid assembly could enable a scalable, automated bacterial genome closure with fully resolved assemblies
2017	Teng et al.	PacBio RS II + Illumina HiSeq	*Burkholderia pseudomallei*	PacBio resolved the high-GC, two-chromosome genome with >99.9% identity, while Illumina assemblies remained fragmented
2017	George et al.	ONT MinION +	8 *Enterobacteriaceae*	Chromosomal contigs matched >95% of the

		Illumina	isolates	reference chromosomal sequence length. Plasmid reconstruction recovered 70% and increased to 78% with combined assembler strategy
2019	De Maio et al.	Illumina + ONT MinION or PacBio RS	20 *Enterobacteriaceae* isolates	LRS improves the reads on repeats, plasmid, and complex regions. In hybrid workflows with Illumina data, it enables more complete, accurate, and automation-friendly bacterial genome assemblies
2019	Sydenham et al.	Illumina MiSeq + ONT MinION	6 *Bacteroides fragilis* isolates	Generated complete circular assemblies of all isolates, identified 11 circular plasmids, and resolved complete insertion sequences upstream of AMR genes.
2022	Sereika et al.	ONT R10.4	Pure cultures	Assembled near-finished microbial genomes without polishing, reaching 99% accuracy
2024	Sanderson et al.	ONT R10.4.1	13 bacterial isolates	Updated chemistry and ≥40× coverage enabled accurate bacterial reconstruction using just LRS reads
2024	Luan et al.	ONT R9.4.1 + Illumina MiSeq	15 *Salmonella enterica serovar*	LRS reached 99.95% accuracy for complete genomes, but "near-perfect" 99.9999% required hybrid approach
2025	Fan et al.	DNBSEQ-T5 + CycloneSEQ-W	*Lactiplantibacillus plantarum* BGI-J9	A hybrid of SRS and LRS a complete genome assembly with 100% completeness.

		T02		
2025	Abdel-Glil et al	ONT R10.4.1 + Illumina MiSeq	*Y. Similis* DSM 18211, *K. pneumonia* DSM 30104, *E. coli* ATCC 25922, *E. coli* ATCC 35218, and *A. baumannii* DSM 30007	Demonstrated that nanopore-only assemblies are highly reproducible, though short-read polishing further refines consensus accuracy.
2025	Wang et al.	DNBSEQ-T5 + CycloneSEQ-W T02	*Lacticplantibacillus plantarum* BGI-N6	Achieved a complete genome assembly, replacing fragmented draft structure with a fully resolved genome.
2025	Xu et al.	ONT R10.4.1	*E. coli* isolates	Provided a high-quality reconstruction of genomes, plasmids, and ARG contexts, yielding 1016 genomes and 2647 circular plasmids
2025	Wang et al.	DNBSEQ-T5 + CycloneSEQ-W T02	*Lacticaseibacillus rhamnosus* OF44	Achieved a complete genome assembly of a single-contig with 99.99% completeness and 0.19% contamination.
2025	Adams et al	PacBio HiFi + Illumina NextSeq	*Staphylococcus aureus* 63-2498	Produced a closed single‑contig chromosome assembly (98.62% complete) along with three plasmids
2026	Nagy et al	ONT R10.4.1 + Illumina	92 *Enterobacterales* isolates	Demonstrated similar results from LRS only assemblies to hybrid assemblies when using

		NovaSeq		Autocycler+Medaka assembler.
2026	Rao et al.	DNBSEQ-T5 + CycloneSEQ-WT02 (Hybrid)	*Lacticaseibacillus paracasei* BGI-N2	Achieved a complete single-contig genome assembly of 3,102,337 bp, with 46.4% GC content and 2,937 CDs.

Table 2. Representative LRS studies for MAG and cMAG recovery from metagenomes.

Year	Study	Platform and strategy	Metagenomic dataset	MAG/cMAG recovery
2019	Bertrand et al.	Illumina + ONT nanopore; OPERA-MS hybrid assembly	Mock communities and 28 human gut metagenomes	Improved contiguity ~200-fold; recovered rare-species genomes and >80 closed plasmid/phage sequences.
2020	Moss et al.	ONT MinION R9.4; Lathe workflow with short-read polishing	13 human stool metagenomes	Recovered 20 circular bacterial genomes directly from microbiomes.
2021	Singleton et al.	ONT PromethION + Illumina; long-read assembly with short-read support	Activated sludge from 23 Danish WWTPs	Recovered 1,083 HQ MAGs, including 57 closed circular genomes.
2022	Bickhart et al.	PacBio HiFi + Hi-C binning; MAGPhase haplotype phasing	Sheep faecal metagenome	Recovered 428 MAGs, including 44 single-contig circular MAGs and 220 lineage-resolved MAGs.
2022	Feng et al.	PacBio HiFi; hifiasm-meta assembler	Seven empirical metagenomic datasets	Reconstructed tens to hundreds of complete circular bacterial genomes per dataset.
2022	Kim et al.	PacBio HiFi, including Sequel II data; metaFlye/HiCanu/hifiasm-meta plus cMAGfilter	Five human faecal metagenomes	Recovered 102 accurate cMAGs, including uncultured gut taxa.
2022	Gounot et al.	Illumina HiSeq4K + ONT MinION/GridION/PromethION; OPERA-MS; Hi-C subset	109 Southeast Asian gut metagenomes	Recovered 4,497 medium/HQ MAGs; 1,708 were absent from short-read-only analysis.
2024	Benoit et al.	PacBio HiFi; metaMDBG assembler	Human gut, anaerobic digester and sheep	Increased HQ circular MAG recovery, with improved plasmid

			rumen datasets	and virus assembly.
2025	Sereika et al.	ONT P2/P24, SQK-LSK114/FLO-PRO114M; Flye/Medaka + mmlong2 workflow	154 soil and sediment samples	Recovered 23,843 MAGs, dereplicated into 15,640 species-level MAGs.
2026	Benoit et al.	ONT R10.4.1; nanoMDBG assembler	Human gut, Zymo Fecal Reference and soil metagenomes	Recovered more near-complete MAGs than ONT comparators; showed ONT can approach HiFi MAG recovery at matched depth.
2026	Shaw et al.	PacBio HiFi and ONT R10.4; myloasm assembler	Gut, oral and other metagenomic datasets	Improved strain-resolved assembly and recovery of complete circular contigs.

Figure

Click here to access/download;Figure;Figure1.pdf

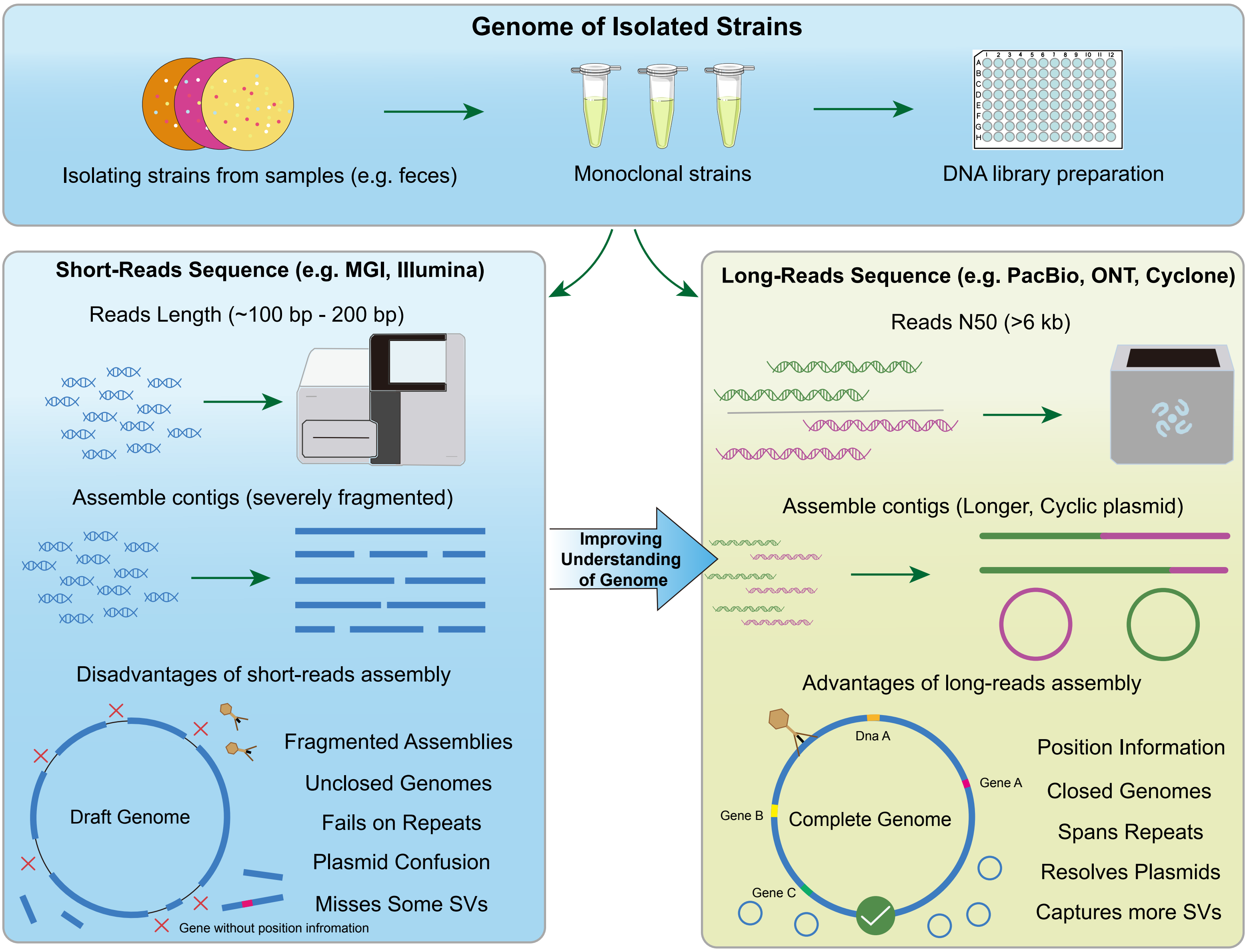

Figure

Click here to access/download;Figure;FIgure2.pdf

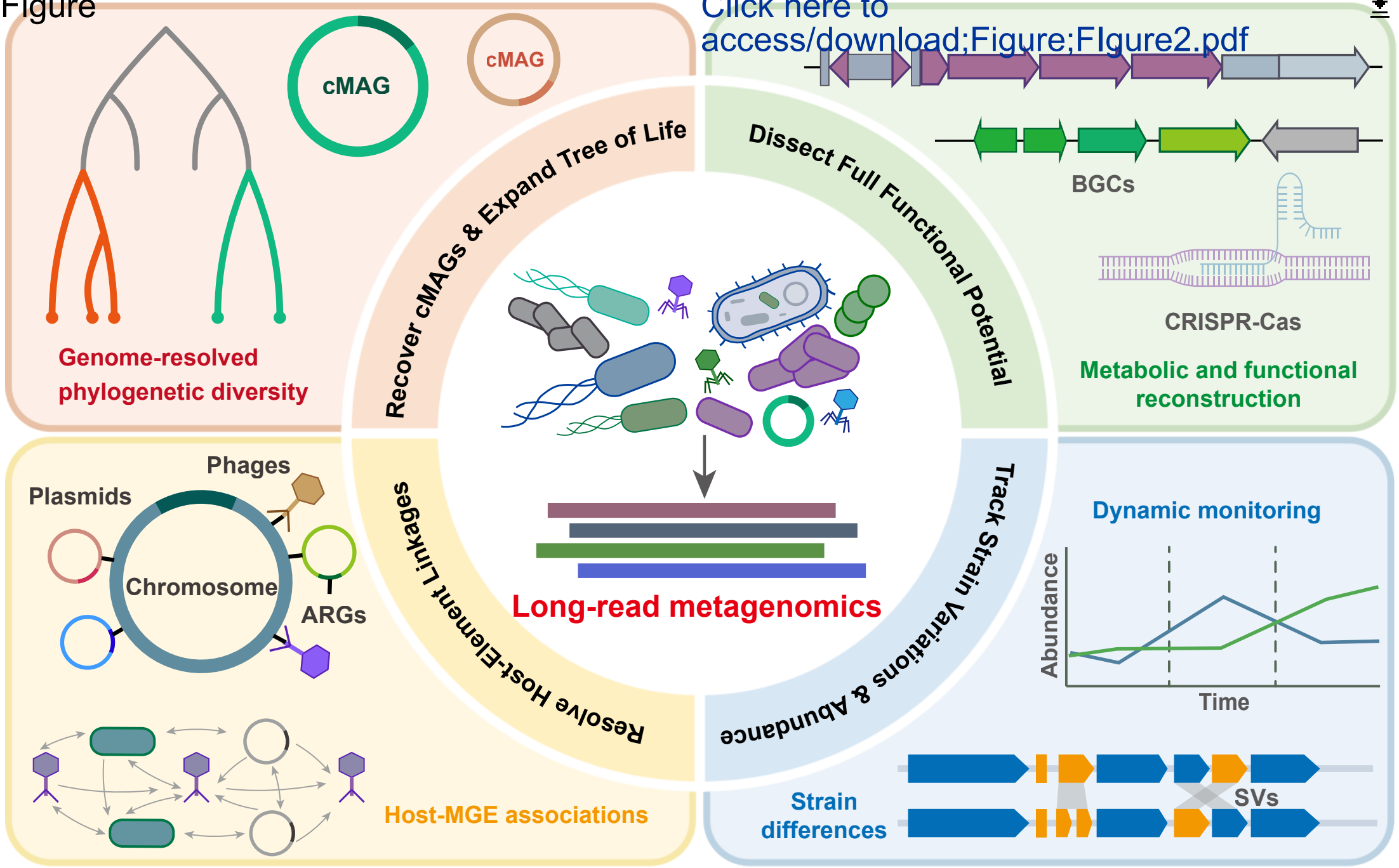

Figure
Click here to access/download;Figure;Figure3.pdf

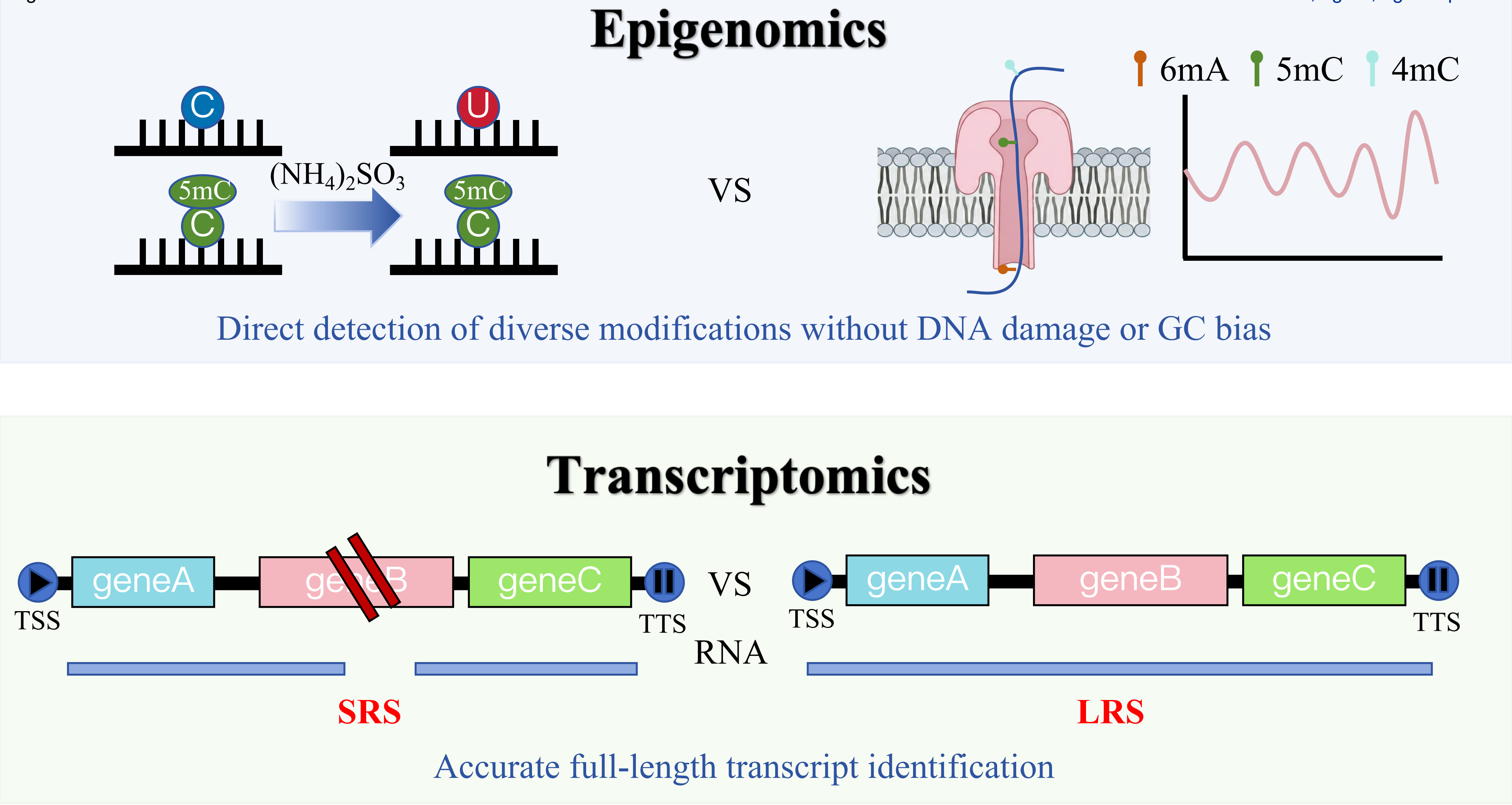

Figure
Click here to access/download;Figure;FIgure4.pdf

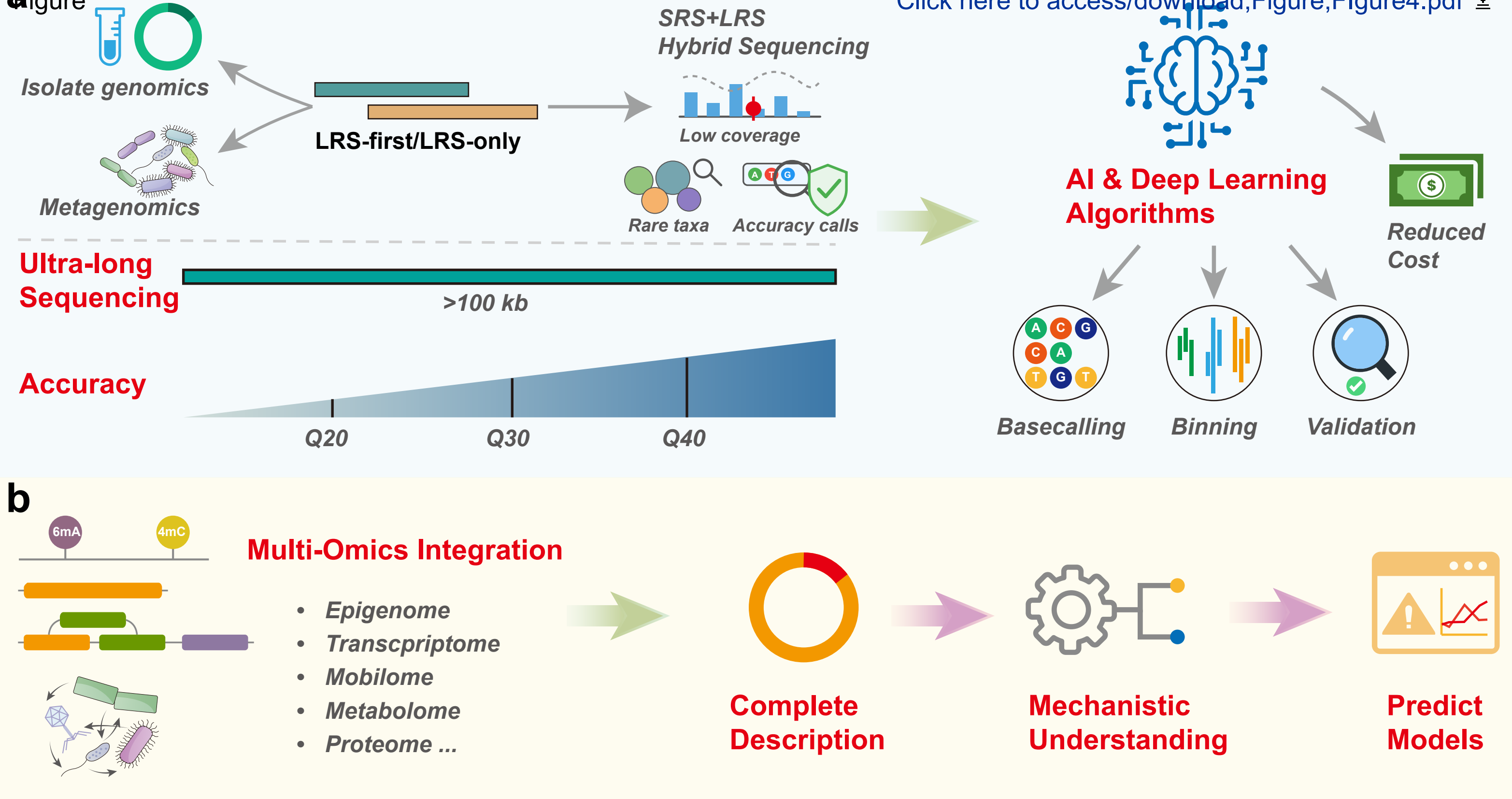

Supplement: Supplementary file 4 [file giag087_giga-d-26-00124_revision_1.pdf]
