# Supplementary material for: Promises and pitfalls of long-read sequencing for resolving microbial complexity
Source: Gigascience. 2026 Aug 17;15:giag087. doi: 10.1093/gigascience/giag087 (PMC13543314; doi:10.1093/gigascience/giag087)
Supplement: Supplementary file 5 [file giag087_giga-d-26-00124_revision_2.pdf]

GigaScience

Promises and Pitfalls of Long-Read Sequencing for Resolving Microbial Complexity

--Manuscript Draft--

**Manuscript Number:**	GIGA-D-26-00124R2
**Full Title:**	Promises and Pitfalls of Long-Read Sequencing for Resolving Microbial Complexity
**Article Type:**	Review
**Funding Information:**	Shenzhen Science and Technology Program (KCXFZ20240903094006009) — Dr. Yuanqiang Zou<br>Shenzhen Medical Research Fund (JCYJ20241202124801003) — Dr. Yuanqiang Zou
**Abstract:**	Long-read sequencing (LRS) has driven a transition in microbial genomics, overcoming the assembly fragmentation inherent to short-read sequencing.<br>This review elucidates the impact of LRS across isolate genomics, metagenomics, and multi-omics domains. By spanning extensive repetitive regions, LRS facilitates the reconstruction of circular chromosomes and precisely resolves mobile genetic elements (MGEs). In metagenomics, LRS enables strain-level resolution, the recovery of circular metagenome-assembled genomes, and the precise localization of MGEs within host replicons. Furthermore, the single-molecule, amplification-free properties of LRS provide enhanced resolution of native epigenetic modifications and full-length transcriptomes. Despite these advancements, widespread implementation remains constrained by multidimensional challenges, including stringent high-molecular-weight DNA requirements, depth deficits, and computational overhead. Nevertheless, LRS is increasingly becoming the method of choice for isolate genomics and metagenomics. As detection technologies and algorithms progress, LRS will further improve our ability to decipher the structural and functional diversity of microbial ecosystems.
**Corresponding Author:**	Yuanqiang Zou<br>BGI-Shenzhen: BGI Group<br>Shenzhen, CHINA
**Corresponding Author Secondary Information:**	
**Corresponding Author's Institution:**	BGI-Shenzhen: BGI Group
**Corresponding Author's Secondary Institution:**	
**First Author:**	Xing Rao
**First Author Secondary Information:**	
**Order of Authors:**	Xing Rao<br>Yuzheng Gu<br>Gabriella<br>Jiayi Ma<br>Haoyu Wang<br>Yuanqiang Zou
**Order of Authors Secondary Information:**	
**Response to Reviewers:**	Reviewer reports:<br><br>Reviewer #1: The authors provided appropriate responses to each comment and improved the manuscript, so I think it is possible to publish this review covering the current possibilities of long-read sequencing in microbial genomics.<br>Response:<br>We sincerely thank the reviewer for the positive evaluation of our revised manuscript and for recognizing the improvements made in response to the previous comments.

Powered by Editorial Manager® and ProduXion Manager® from Aries Systems Corporation

We are also grateful for the reviewer's constructive suggestions, which helped us clarify the scope and strengthen the discussion of current applications of long-read sequencing in microbial genomics.

Reviewer #2: Summary: In this revised version of their review manuscript on long-read sequencing for resolving microbial complexity, Rao et al. implemented my comments to a limited extent.

Major comments:
1. Despite the authors' claim to have reduced redundancy, many parts still read repetitive. Notably, the point about reduced fragmentation due to longer reads being used in genomic assembly is coming up again and again. Often even as the main point, thus adding little new information. After all, this is what long read sequencing was developed for. This point also only differs marginally between isolate cultures and microbiomes. Meta-assemblers provide additional solutions for the difference in coverage but otherwise are highly similar to "non-meta" assemblers. Moreover, long reads hold potential beyond contigs, i.e., due to their length, relevant functional information covering neighboring genes could be recovered. As one early concrete example of repetitiveness, take L64+L65 and L72ff. Basically, L72ff are examples of "intricate genomic architecture". However, the reading flow is disrupted and comes back on a previous point. This is persistent throughout the manuscript.
Response:
We thank the reviewer for this important comment. We agree that the previous version still repeated the point that long reads reduce assembly fragmentation. In the revised manuscript, we therefore performed a more systematic revision to reduce redundancy and improve the flow of the review.
First, we reorganized the Introduction so that the genomic features that challenge SRS, including repetitive elements, MGEs, and GC-biased regions, are discussed together before LRS is introduced. This avoids returning to the same argument after the role of LRS has already been presented. "By integrating bridge amplification with sequencing-by-synthesis, SRS circumvented the need for microbial cultivation and supported landmark metagenomic initiatives like the Human Microbiome Project and METAgenomics of the Human Intestinal Tract. Despite these advances, SRS reads remain short relative to many microbial genomic features. This limitation is particularly evident in three contexts. First, repetitive elements, including ribosomal RNA (rRNA) operons and insertion sequences (ISs), can constitute up to 10% of some bacterial genomes, and their length may exceed the resolving capacity of SRS algorithms. Second, for mobile genetic elements (MGEs), such as plasmids and prophages, shared sequences and integration sites are difficult to assign from short reads alone. Third, extreme guanine–cytosine (GC) content may introduce coverage bias and lead to the underrepresentation or loss of affected regions. In response to these challenges, LRS platforms such as PacBio, Oxford Nanopore Technologies (ONT) and CycloneSEQ generate substantially longer reads and preserve broader genomic context. These features have expanded the analytical scope of microbial genomics. "(Lines 61-76)
Second, we revised repetitive descriptions that contrasted fragmented drafts with complete assemblies. For example, we removed the sentence "As a result, short reads frequently fail to resolve complex repetitive regions, leading to gaps, misassemblies, and ambiguous contig ordering". We also condensed the discussion of Koren et al. to focus on the main finding "Through an analysis of 2,267 complete bacterial and archaeal genomes, Koren et al. found that most microbial genomes could be closed using LRS" (Lines 121-122). In addition to these examples, we made revisions in other parts of the manuscript (Lines 122-126, 146-149, 214-216, 485-487, and 585-586) and made several smaller wording changes throughout the text to further reduce redundancy. Across these revisions, we shifted the emphasis from contig length and reduced fragmentation alone to the biological information enabled by LRS, including plasmid and phage context, ARG localization, and strain variation.

2. I find that the points I raised have been addressed rather superficially. My comment on "haplotype parsing" was apparently only considered for Figure 2. However, uses in the text remain unchanged. Capitalization is inconsistent, notably in Figure 1 and Figure 4. As listed below several minor comments remain. The references seem not to have been checked thoroughly, e.g., L958 "48. . !!! INVALID CITATION !!! [45]."
Response:

Thanks very much for pointing this out. We have made the corrections and checked these issues throughout the manuscript. First, we reviewed "haplotype parsing" and related wording throughout the manuscript. We replaced the remaining uses of "strain parsing" or ambiguous "parsing" terminology with more precise terms, such as "strain resolved analysis". Second, we checked the capitalization in the figures and revised them for consistency. Third, we carefully rechecked the reference list and in text citations. The invalid citation noted by the reviewer has been corrected, and the reference formatting has been reviewed to remove citation errors and inconsistencies. Finally, we also addressed the remaining minor comments listed below and revised the corresponding text throughout the manuscript. We appreciate the reviewer's careful reading, which helped us improve the consistency, accessibility, and accuracy of the revised review.

3. The review requires a certain domain knowledge to be accessible to the readers. The authors refer to several things that are not explained, e.g., de Bruijn graph-based assembly vs. OLC assembly. This limits the accessibility to the uninformed reader.
Response:
Good suggestion. We revised the Section 2 to clarify the distinction between assembly based on de Bruijn graphs and OLC assembly. Specifically, we added the following explanatory text: "SRS assembly commonly uses approaches based on de Bruijn graphs, in which short reads are decomposed into k-mers and reconstructed by connecting overlapping k-mers. This strategy is computationally efficient, but it becomes ambiguous when complex regions exceed the length of short reads. In contrast, LRS assembly commonly adopts an overlap layout consensus (OLC) approach, in which longer reads are compared directly and ordered according to overlaps between reads to generate a consensus sequence. This enables more reliable reconstruction of complete chromosomal genomes and extrachromosomal elements, including plasmids and circular phage DNA." (Lines 92-101)
In addition, we reviewed the manuscript for other terms that may be difficult for readers who are not specialists in LRS and added brief explanations for SNVs, SVs, Q30 and Q40. Specifically, we added: "This includes single nucleotide variants (SNVs), which are single base differences at specific genomic positions, especially when these variants occur within complex genomic architecture." (Lines 164-166) We also added: "LRS is particularly valuable for resolving structural variants (SVs), which are genomic changes larger than 50 bp, including inversions, duplications, insertions, deletions and translocations." (Lines 171-174). We clarified the notation for microbial epigenetic modifications by revising the text to "Microbial epigenetic modifications, predominantly N6-methyladenine (6mA), 5-methylcytosine (5mC), and N4-methylcytosine (4mC)…" (Lines 415-417). Finally, we clarified sequencing quality scores as follows: "The field is therefore moving toward Q30-class long reads, corresponding to approximately one error per 1,000 bases, as a realistic standard in optimized workflows. Based on this, Q40-grade reads, corresponding to approximately one error per 10,000 bases, are also becoming plausible." (Lines 604-608)

4. This is up to the journal, but I could not find where the authors comply with the requirement of "A summary of use (particularly in the introduction or among methods) needs to be included at the end of the paper, and the outputs should also be included as a supplementary file hosted in GigaDB or other open repositories." The authors only mention that Gemini 3.1 Pro and what for, but not how or what the outputs were.
Response:
We thank the reviewer for noting this requirement. We have provided a more detailed statement in the "Disclosure of use of AI-assisted tools including generative AI" section at the end of the manuscript. The revised disclosure states: "The authors used Gemini 3.1 Pro as an auxiliary tool for language checking and wording suggestions during manuscript preparation. No AI-generated content was directly incorporated into this study without author review and revision." (Lines 744-747) Accordingly, we understand that there is no corresponding AI generated content to submit as a supplementary file.

L60: Why is DNBSEQ listed here, representing the arguably most recent technology in the field? Other approaches, such as by SOLID or IonTorrent, predate DNBSEQ by many years. Hence presenting it in this way is not logical to me.
Response:
We thank the reviewer for pointing this out.Our intention was not to present DNBSEQ as one of the earliest SRS technologies, but to give examples of representative SRS

platforms. We have revised the sentence to present these platforms as examples of SRS technologies rather than as a chronological sequence: “However, throughput constraints necessitated the development of short-read sequencing (SRS) technologies, such as Illumina, DNA nanoball sequencing (DNBSEQ), and Ion Torrent.”(Lines 58-60)

L79: Where is "MGE" introduced?
Response:
We thank the reviewer for pointing this out. We have revised the Introduction to define mobile genetic elements (MGEs) at their first occurrence in the main text.

L88: Remove "the" from "the microbiological"
Response:
We thank the reviewer for pointing this out. Because we reorganized the Introduction, the original sentence containing “the microbiological” has been removed from the revised manuscript. Therefore, the problematic phrase no longer appears in the text.

L98: What do the authors mean by "integrative perspectives"?
Response:
We apologize that the original wording was not sufficiently clear. Our intention was to refer to the combined discussion of isolate genomics, metagenomic host element linkages, epigenomics and full-length transcriptomics, and technical limitations. To improve clarity, we have revised the sentence and replaced "integrative perspectives" with a more explicit statement: "Together, these perspectives clarify the role of LRS in resolving the genomic and functional diversity of microorganisms." (Lines 85-86)

L106: Not sure the "single" is necessary.
Response:
We thank the reviewer for this comment. We intended to refer to genomes obtained from pure cultured isolates, but agree that “single” overlaps in meaning with “isolate.” We have therefore revised “single-isolate” to “isolate” throughout manuscript for consistency.

L134: Not sure what is meant by "high accuracy read per base"?
Response:
We thank the reviewer for pointing this out. We intended to refer to the high nucleotide accuracy of SRS reads, but the sentence repeated an earlier point about the limitation of SRS. We have therefore removed this wording and revised the sentence. “By spanning genomic regions that are difficult to resolve with SRS, these platforms made complete genome assembly of isolates more feasible.” (Lines 115-117)

L135: What is specifically meant by "complex regions"? What qualifies as such?
Response:
We thank the reviewer for requesting clarification. Here, "complex regions" meant genomic regions that are difficult to resolve with short reads, including repeated or highly similar sequences and regions affected by structural variation. We revised this sentence to avoid repeating the earlier discussion.“By spanning genomic regions that are difficult to resolve with SRS, these platforms made complete genome assembly of isolates more feasible.”(Lines 115-117)

L157: The approaches were not "focused" on drafts but there was simply no better solution readily available. The reference to "finished gapless" is redundant with what follows ("structural completeness").
Response:
We thank the reviewer for this helpful comment.We have revised this sentence to avoid implying that draft assemblies were an intentional preference and to remove the redundant phrase “finished, gapless assemblies.” The revised sentence now reads: "Collectively, these studies supported a shift in genome quality standards from draft assemblies toward complete microbial reference genomes, reflecting the ability of LRS to recover genome continuity at the chromosomal level and provide a stronger basis for functional and structural analysis." (Lines 134-137)

L204: Consider removing "where SRS alignment …" as it is redundant with all the rest.
Response:

We agree that this final clause is redundant, as the limitations of SRS have already been thoroughly established in the preceding text. The phrase has been removed.

L249: What do the authors mean by "inference" here?
Response:
We apologize that the original sentence was unclear. We meant that assigning blaKPC and Tn4401 to a particular plasmid based on partial sequence similarity or the detection of a known plasmid backbone in short-read data may be unreliable. We have revised the text to describe the point more explicitly: "Sheppard et al. applied LRS to 17 blaKPC-positive Enterobacteriaceae isolates. This enabled precise localization of blaKPC and its associated transposon Tn4401, clarifying the linked roles of blaKPC-bearing host strain transmission, plasmid transfer across strains and species, and Tn4401 transposition among plasmids in blaKPC dissemination."(Lines 198-201)

L267: Not sure what the "moving away beyond taxonomic inventories" brings in here. This applies to SRS as well as LRS.
Response:
Thank you for this comment. We agree that moving beyond taxonomic inventories is not unique to LRS and that the original sentence did not clearly identify the specific contribution of long-read metagenomics. We have removed this general statement and replaced it with the concrete applications presented in Figure 2 and Table 2, including genome recovery, strain resolution, and localization of functional loci and integrated mobile elements within assembled genomes. We have revised this sentence as "These developments enable long-read metagenomics to recover complete or near-complete genomes, distinguish closely related strains, and determine where functional loci and integrated mobile elements occur within assembled genomes (Figure 2 and Table 2)." (Lines 218-221)

L298: Why are SRS binning strategies "prone to missing low-abundance" but LRS binning strategies are not? In fact, LRS has a much lower sequencing depth, which is a considerable challenge for low-abundance organisms.
Response:
Thank you for identifying this unsupported comparison. After re-evaluating previous related studies, we agree that low-abundance taxa are not inherently more recoverable using LRS. Their recovery is primarily determined by species-specific sequencing depth, and the lower total yield of many long-read datasets may further limit the representation of rare community members. Although long reads can provide greater genomic context when a low-abundance taxon is sufficiently sampled, this should not be interpreted as greater sensitivity for detecting or recovering rare taxa. We have therefore deleted the original comparison between short- and long-read binning here.

L301: Long-read metagenomic sequencing does not "generate" "molecules"; it generates sequences.
Response:
Thanks very much for pointing this out. We have deleted this sentence.

L318: Not sure what is mean by "converted" in this context.
Response:
Thank you for pointing out this ambiguity. We agree that "converted" was imprecise because LRS does not convert marker-gene signals into genomes. Rather, the cited study recovered genome representatives for previously underrepresented taxa and associated complete functional and structural elements with those genomes. We have revised this sentence as "These assemblies also recovered thousands of complete rRNA operons, biosynthetic gene clusters (BGCs), and CRISPR-Cas systems". (Lines 259-261)

L326: "parsing" remains used.
Response:
Thank you for identifying this remaining inconsistency. We have now conducted a full-text search and replaced all three remaining instances. Specifically, "high-resolution strain parsing" has been changed to "strain-level resolution" in the Abstract and Conclusion, and the Section 3.2 heading has been revised to "Strain-level resolution".

L328: Wrong capitalization at start of sentence.

Response:
Thanks for your comment. We have corrected it.

L425: I do not understand how LRS connects "function to mechanism". If at all, the authors show that mechanism is hard to get to, not least due to epigenetic/posttranscriptional/posttranslational modifications.
Response:
We agree that the phrase "connects function to mechanism" was imprecise and could imply that LRS alone demonstrates molecular mechanism. We have removed this wording and revised this sentence to describe the contribution more directly. The revised text is "Together, these studies show that LRS improves resistome and mobilome analysis by placing functional genes in their genomic background. In short-read datasets, detection of an ARG, virulence gene, or prophage often shows only that the sequence is present. LRS can further indicate whether these loci are located on chromosomes, plasmids, or transposons, which is essential for assessing their host association and potential mobility". (Lines 355-358)

L431: Formatting of heading missing.
Response:
Thank you for pointing this out. The formatting of the heading "3.4 Pathogen genomics and real-time detection" was inadvertently omitted. We have formatted this heading in bold to ensure consistency with the other subsection headings.

L435: Real-time sequencing is a particular feature of Oxford Nanopore Technologies approach, but not common to LRS. Please adjust accordingly.
Response:
We agree with the reviewer that real-time sequencing is a platform-specific feature of Oxford Nanopore Technologies rather than a general property of all LRS platforms. We have revised this sentence as "ONT-based LRS provides a culture-independent route from clinical specimens to pathogen information, making it an important translational extension of genome-resolved microbial genomics. Its distinctive advantage lies in the ability to generate analyzable reads during the run". (Lines 363-366)

L442: What does "mNGS" mean?
Response:
Thank you for pointing this out. We have defined this term by replacing "mNGS" with "metagenomic next-generation sequencing" in the relevant sentence. (Lines 372)

L486: Why is "core" needed here?
Response:
We thank the reviewer for pointing out that the term "core" may imply universal conservation or evolutionary indispensability among microbial epigenetic modifications. We have removed "core" and revised the sentence to refer more generally to microbial epigenetic modifications, including 6mA, 5mC, and 4mC. “Microbial epigenetic modifications, predominantly N6-methyladenine (6mA), 5-methylcytosine (5mC), and N4-methylcytosine (4mC), are associated with restriction-modification (R-M) systems, gene expression, and host-pathogen interactions”(Lines 415-418)

L491: Why is that argument brought up? SRS was never designed to provide single-molecule solution; at least no SRS solution got meaningful adoption/market share for that.
Response:
We appreciate the reviewer’s insightful comment. We agree that SRS technologies were not designed to provide single-molecule resolution, and our original wording may incorrectly suggest this comparison. We have revised this section to clarify the distinction between conventional SRS based DNA methylation approaches, which often rely on bisulfite chemical conversion and generally provide population level methylation profiles, and LRS approaches, which preserve native DNA molecules and enable direct modification detection. The revised text can be found in Lines 418-428 as: "Historically, SRS based approaches for DNA methylation detection have often relied on bisulfite chemical conversion, which can cause DNA degradation and sequence bias. Because methylation profiles are inferred from aggregated sequencing reads, these approaches generally provide population-level methylation profiles rather than direct measurements of individual DNA molecules. In contrast, LRS preserves

	native DNA molecules and enables direct modification detection: PacBio SMRT utilizes polymerase kinetic characteristics, while ONT detects changes in nanopore ionic current. Both platforms enable simultaneous DNA sequencing and single-molecule methylation detection without PCR amplification or chemical conversion, allowing direct linkage of epigenetic modifications to their genomic context on individual DNA molecules."<br><br>L515: "HMW" not introduced.<br>Response:<br>Thank you, We have added the full term "high-molecular-weight (HMW) DNA" at its first appearance in the revised manuscript. (Lines 445)<br><br>L534: "TSS" and "TTS" not introduced.<br>Response:<br>Thank you for pointing this out. We have introduced the full terms "transcriptional start sites (TSSs)" and "transcriptional termination sites (TTSs)" at their first appearance (Lines 467) in the revised manuscript.<br><br>L540: What is meant by "defined" here?<br>Response:<br>Thank you for pointing this out. We agree that the term "defined" was ambiguous and could be misinterpreted. To improve clarity, we have revised the sentence to emphasize that ONT direct RNA sequencing refined the transcriptional boundaries of previously identified operons, rather than suggesting that the operons themselves were "defined" The revised text in lines 473-475: "For instance, ONT direct RNA sequencing refined the transcriptional boundaries of previously identified operons, revealing extended boundaries for 225 operons in E. coli and 89 operons in S. aureus."<br><br>L569: Why "intact", when it already include "HMW" in the sentence? Importantly, how likely is it to recover fully intact DNA, i.e., a full genome as a single, intact molecule?<br>Response:<br>We thank the reviewer for raising this point. Our intention was to indicate high-molecular-weight DNA with minimal fragmentation, not recovery of a complete genome as a single intact DNA molecule. To avoid this misunderstanding, we have removed "intact" from the sentence and now refer only to high purity and high molecular weight DNA.<br><br>L691: Please use "cMAGs" to be consistent.<br>Response:<br>Thanks very much for your suggestion. We have made the corrections and checked it throughout the manuscript.(Lines 277 and 623)<br><br>L699: "AI" not introduced. Importantly, assuming "Artificial Intelligence" is meant here, this is a very generic term.<br>Response:<br>We agree that "AI" was too generic and had not been introduced. We have revised this sentence as "Machine-learning and deep-learning methods are likely to contribute to this process". (Lines 631)<br><br>L766f: Not sure why "gapless" is needed in addition to "completeness".<br>Response:<br>We thank the reviewer for pointing out this redundancy. We agree that "gapless" was unnecessary in addition to "completeness," and we have removed it from the sentence.<br><br>L768: Why is "microbial" added here when referring to metagenomics?<br>Response:<br>We thank the reviewer for pointing this out. The review already focuses on microbial genomics and metagenomics, the word "microbial" was redundant in this sentence. We have therefore removed it.
**Additional Information:**	
**Question**	**Response**
Are you submitting this manuscript to a	No

special series or article collection?	
**Experimental design and statistics**<br><br>Full details of the experimental design and statistical methods used should be given in the Methods section, as detailed in our Minimum Standards Reporting Checklist. Information essential to interpreting the data presented should be made available in the figure legends.<br><br>Have you included all the information requested in your manuscript?	No
If not, please give reasons for any omissions below.<br><br>as follow-up to "**Experimental design and statistics**<br><br>Full details of the experimental design and statistical methods used should be given in the Methods section, as detailed in our Minimum Standards Reporting Checklist. Information essential to interpreting the data presented should be made available in the figure legends.<br><br>Have you included all the information requested in your manuscript?<br><br>"	This manuscript is a review article and does not present original experimental data. Therefore, no experimental design or statistical methods are applicable. The manuscript has been prepared in accordance with the journal's guidelines for reviews, and all relevant information has been included.
**Resources**<br><br>A description of all resources used, including antibodies, cell lines, animals and software tools, with enough information to allow them to be uniquely identified, should be included in the Methods section. Authors are strongly encouraged to cite Research Resource Identifiers (RRIDs) for antibodies, model organisms and tools, where possible.	No

Have you included the information requested as detailed in our Minimum Standards Reporting Checklist?	
If not, please give reasons for any omissions below.<br><br>as follow-up to "**Resources**<br><br>A description of all resources used, including antibodies, cell lines, animals and software tools, with enough information to allow them to be uniquely identified, should be included in the Methods section. Authors are strongly encouraged to cite Research Resource Identifiers (RRIDs) for antibodies, model organisms and tools, where possible.<br><br>Have you included the information requested as detailed in our Minimum Standards Reporting Checklist?<br><br>"	This manuscript is a review article and does not present original experimental data. Therefore, no experimental design or statistical methods are applicable. The manuscript has been prepared in accordance with the journal's guidelines for reviews, and all relevant information has been included.
**Availability of data and materials**<br><br>All datasets and code on which the conclusions of the paper rely must be either included in your submission or deposited in publicly available repositories (where available and ethically appropriate), referencing such data using a unique identifier in the references and in the "Availability of Data and Materials" section of your manuscript.<br><br>Have you have met the above requirement as detailed in our Minimum Standards Reporting Checklist?	No
If not, please give reasons for any omissions below.<br><br>as follow-up to "**Availability of data and materials**	This manuscript is a review article and does not present original experimental data. Therefore, no experimental design or statistical methods are applicable. The manuscript has been prepared in accordance with the journal's guidelines for reviews, and all relevant information has been included.

All datasets and code on which the conclusions of the paper rely must be either included in your submission or deposited in publicly available repositories (where available and ethically appropriate), referencing such data using a unique identifier in the references and in the "Availability of Data and Materials" section of your manuscript.<br><br>Have you have met the above requirement as detailed in our Minimum Standards Reporting Checklist?<br><br>"	
GigaScience has policies and guidelines in place for the use of generative AI-writing tools such as ChatGPT. If you have used such writing tools to assist with writing the manuscript this must be declared and cited in the text. Authors should not list AI-writing tools and other AI-assisted technologies as an author or co-author and should acknowledge that they are fully responsible for text generated or refined by AI-writing tools.<p><br><br>A summary of use (particularly in the introduction or among methods) needs to be included at the end of the paper, and the outputs should also be included as a supplementary file hosted in GigaDB or other open repositories. Please read our guidelines for more information. <p><br><br>By submitting to GigaScience, you are aware of the journal's AI-writing tools policy, and if you have declared use of such tools below, you have acknowledged this where appropriate in your manuscript and have made a summary of use and outputs available. <p><br><b>AI-assisted writing tools have been used in the preparation of this manuscript?	Yes

Manuscript

Click here to access/download;Manuscript;Giga-manuscript_R3.docx

Promises and Pitfalls of Long-Read Sequencing for Resolving Microbial Complexity

Xing Rao (饶兴)[1,2#], Yuzheng Gu (顾雨铮)[3#], Gabriella[1,4], Jiayi Ma (马嘉仪)[1,2], Haoyu Wang (王浩宇)[1,2], Yuanqiang Zou (邹远强)[1,5]

1. State Key Laboratory of Genome and Multi-omics Technologies, BGI Research, Shenzhen 518083, China

2. College of Life Science, University of Chinese Academy of Sciences, Beijing 100049, China

3. Key Laboratory of Agricultural and Environmental Microbiology, Ministry of Agriculture and Rural Affairs, Department of Microbiology, College of Life Sciences, Nanjing Agricultural University, Nanjing 210095, China

4. Department of Food Science and Biotechnology, Faculty of Agricultural Technology, Universitas Brawijaya, Malang 65145, Indonesia

5. Shenzhen Key Laboratory of Human commensal microorganisms and Health Research, BGI Research, Shenzhen 518083, China

# These authors contributed equally.

Corresponding author:

Yuanqiang Zou.

Telephone: +86-13147016380

Fax: 0755-36352579

Address: State Key Laboratory of Genome and Multi-omics Technologies, BGI

Research, Shenzhen 518083, China

Email: zouyuanqiang@genomics.cn

Yuanqiang Zou will handle the correspondence of the article at all stages of the review and publication process, as well as post-publication.

Author's email: raoxing@genomics.cn, guyuzheng513@163.com, gabriella@genomics.cn, majiayi@genomics.cn, wanghaoyu2@genomics.cn, zouyuanqiang@genomics.cn

Abstract

Long-read sequencing (LRS) has driven a transition in microbial genomics, overcoming the assembly fragmentation inherent to short-read sequencing.

This review elucidates the impact of LRS across isolate genomics, metagenomics, and multi-omics domains. By spanning extensive repetitive regions, LRS facilitates the reconstruction of circular chromosomes and precisely resolves mobile genetic elements (MGEs). In metagenomics, LRS enables strain-level resolution, the recovery of circular metagenome-assembled genomes, and the precise localization of MGEs within host replicons. Furthermore, the single-molecule, amplification-free properties of LRS provide enhanced resolution of native epigenetic modifications and full-length transcriptomes. Despite these advancements, widespread implementation remains constrained by multidimensional challenges, including stringent high-molecular-weight DNA requirements, depth deficits, and computational

overhead. Nevertheless, LRS is increasingly becoming the method of choice for isolate genomics and metagenomics.

As detection technologies and algorithms progress, LRS will further improve our ability to decipher the structural and functional diversity of microbial ecosystems.

Keywords: Long-read sequencing, Microbial genomics, Metagenomics, Genome assembly.

1. Introduction

The evolution of sequencing technologies has improved our ability to resolve structural complexity in microbial genomes. From Sanger sequencing to single-molecule real-time platforms, each technical transition reflects improvements in throughput, cost efficiency, and analytical scope. Sanger sequencing, characterized by dideoxynucleotide chain termination, enabled the first genome draft assembly of a cultured microorganism, *Haemophilus influenzae*, marking the formal entry of microbiology into the genomic era[1, 2]. However, throughput constraints necessitated the development of short-read sequencing (SRS) technologies, such as Illumina, DNA nanoball sequencing (DNBSEQ), and Ion Torrent[3-5]. By integrating bridge amplification with sequencing-by-synthesis, SRS circumvented the need for microbial cultivation and supported landmark metagenomic initiatives like the Human Microbiome Project and METAgenomics of the Human Intestinal Tract[6-8].

Despite these advances, SRS reads remain short relative to many microbial genomic features[9]. This limitation is particularly evident in three contexts. First, repetitive elements, including ribosomal RNA (rRNA) operons and insertion

sequences (ISs), can constitute up to 10% of some bacterial genomes, and their length may exceed the resolving capacity of SRS algorithms[10-12]. Second, for mobile genetic elements (MGEs), such as plasmids and prophages, shared sequences and integration sites are difficult to assign from short reads alone[13-15]. Third, extreme guanine–cytosine (GC) content may introduce coverage bias and lead to the underrepresentation or loss of affected regions[16-18].

In response to these challenges, LRS platforms such as PacBio, Oxford Nanopore Technologies (ONT) and CycloneSEQ generate substantially longer reads and preserve broader genomic context[19-21]. These features have expanded the analytical scope of microbial genomics. However, current reviews of LRS have focused mainly on biomedical surveys or algorithmic analyses[22-24]. For instance, Agustinho et al. provided a comprehensive synthesis of computational pipelines for long-read metagenomic assembly and taxonomic characterization, serving as an invaluable methodological reference[25]. Nevertheless, that review was primarily methodological and centered on metagenomics. Our review systematically covers the reconstruction of complete isolate genomes, the resolution of metagenomic populations with host–element linkages, and the characterization of native epigenomic and full-length transcriptomic landscapes. We also offer an evaluation of the technical limitations of LRS relative to SRS. Together, these perspectives clarify the role of LRS in resolving the genomic and functional diversity of microorganisms.

2. Genomics of isolates: reshaping the standard of "reference genomes."

The standard for high-quality reference genomes has expanded beyond base accuracy to requiring structural completeness (Figure 1). This change is driven by broader access to analytical tools and reduced sequencing costs[26-29]. Historically, isolate microbial reference genomes relied heavily on SRS platforms[30]. SRS assembly commonly uses approaches based on de Bruijn graphs, in which short reads are decomposed into k-mers and reconstructed by connecting overlapping k-mers[26, 27, 31]. This strategy is computationally efficient, but it becomes ambiguous when complex regions exceed the length of short reads. In contrast, LRS assembly commonly adopts an overlap layout consensus (OLC) approach, in which longer reads are compared directly and ordered according to overlaps between reads to generate a consensus sequence. This enables more reliable reconstruction of complete chromosomal genomes and extrachromosomal elements, including plasmids and circular phage DNA[32].

Figure 1 Comparative assembly of isolate microbial genomes using SRS and LRS. Conventional SRS assemblies may incompletely resolve complex repetitive elements, extrachromosomal plasmids, and structural variations (SVs). Conversely, LRS provides longer reads to yield complete genomes supporting plasmid resolution, and comprehensive SV detection for downstream genomic interpretation.

Alt text: A comparative flowchart on microbial genome assembly using SRS and LRS, illustrating the technical transition from fragmented draft genomes to gapless, complete circularized chromosomes and resolved genomic features.

2.1. Achieving gapless genomes: from fragmentation to continuity

The commercialization of LRS platforms, initiated by PacBio SMRT in 2011 and followed by ONT MinION in 2014, represented a milestone in third-generation sequencing technologies[33]. By spanning genomic regions that are difficult to resolve with SRS, these platforms made complete genome assembly of isolates more feasible (Figure 1 and Table 1).

Chin et al. showed that PacBio SMRT sequencing was able to produce *de novo* microbial genome assemblies with over 99.999% consensus accuracy, demonstrating that complete genomes no longer required labor-intensive finishing strategies[34]. Through an analysis of 2,267 complete bacterial and archaeal genomes, Koren et al. found that most microbial genomes could be closed using LRS[35]. The advantage of LRS was not limited to contiguity; ultra-long reads exceeding 32 kb enabled sequencing of entire adhesin-encoding genes (>6 kb) and antibiotic biosynthesis loci (>15 kb), preserving the genomic context of functional loci that was difficult to reconstruct with short read data[36]. In *Lactiplantibacillus pentosus* LP309, LRS recovered the complete chromosome and eight plasmids, enabling the prediction of over 150 probiotic functional genes involved in acid resistance and vitamin synthesis[37]. Similarly, a recent human gut genome collection of 1,150 circular genomes across 199 species-level clusters further demonstrated that complete assemblies substantially improve downstream functional inference[38]. Metabolic models constructed from these complete genomes recovered 4,764 additional

reactions relative to draft-based models, the majority of which were transport-related[39]. Collectively, these studies supported a shift in genome quality standards from draft assemblies toward complete microbial reference genomes, reflecting the ability of LRS to recover genome continuity at the chromosomal level and provide a stronger basis for functional and structural analysis.

2.2. Beyond the chromosome: unveiling the full architecture of plasmids and mobile genetic elements

Microbial genomes harbor a diverse repertoire of MGEs that drive horizontal gene transfer (HGT), including plasmids, prophages, transposons, ISs, integrons, and integrative and conjugative elements[40]. SRS can identify many MGE markers, but reconstructing the architecture of plasmids and other accessory replicons remains difficult, especially for large plasmids with shared or repetitive regions. A benchmark of 42 bacterial genomes containing 148 plasmids showed that SRS-based approaches frequently failed to recover complete plasmid structures[41, 42]. As a result, accessory genes encoded within these structures, such as antibiotic resistance genes (ARGs) and virulence factors, may be assigned to incomplete or incorrect genomic contexts, reducing the accuracy of downstream analysis[41, 43].

LRS enables plasmids to be reconstructed as individual replicons while preserving the associated MGE context. Jiang et al. reconstructed resistance plasmid architecture using PacBio SMRT sequencing, characterizing pooled conjugative plasmids from multidrug-resistant *Klebsiella pneumoniae*[44]. They identified 124

ARGs and 317 MGEs, and established a relationship network between MGEs and ARGs that described dissemination by mobile elements. Beyond structural resolution, reconstructed plasmids also revealed biological relevance. For instance, McLeod et al. resolved the extensive plasmid complement of *Lactiplantibacillus plantarum* MF1298, yielding 14 complete plasmid sequences, one of which carried functional genes associated with vitamin B12 transformation[45]. Wang et al. showed that resolved plasmid-borne genes included functions related to fructose metabolism, indicating that accessory replicons can contribute directly to host-associated metabolic capacity[38].

2.3. Resolving microbial variations and evolutionary dynamics

LRS technologies facilitate the identification of a broader spectrum of variations (Figure 1). This includes single nucleotide variants (SNVs), which are single base differences at specific genomic positions, especially when these variants occur within complex genomic architecture. ONT-based benchmarking showed that SRS failed to detect 9 to 68 SNPs per 1,000 sites in repetitive reference regions. Conversely, by spanning these complex regions, ONT long reads improved SNP calls across previously inaccessible genomic regions[46]. Capturing this broader view is essential because microbial evolution depends not just on massive structural shifts, but also on small, localized mutations and shifting repeat regions[47]. Beyond SNVs, LRS is particularly valuable for resolving structural variants (SVs), which are genomic changes larger than 50 bp, including inversions, duplications, insertions, deletions and translocations. In *Bordetella pertussis*, LRS confirmed both duplications and a large inversion of approximately 1 Mbp between isolates from the same patient[48].

Similarly, in *Salmonella enterica* serovar Typhi, LRS confirmed large genome structure rearrangements generated during long-term culture, and isolates with altered genome structures showed transcriptional changes relative to the parental strain[49].

On an evolutionary scale, the value of LRS is reinforced by population-level analyses. In 134 multidrug-resistant *Salmonella enterica* isolates, LRS revealed resistance-associated structural changes, including differences in the presence and orientation of Salmonella pathogenicity islands, and chromosomal insertion of resistance plasmids[50]. Canalda-Baltrons et al. constructed a pangenome reference graph from 859 high-quality LRS assemblies of *Mycobacterium tuberculosis* and showed that SVs are not marginal events, but an important component of genome evolution, virulence-associated deletions, and drug-resistance architecture[51]. Their subsequent genotyping across 41,134 isolates linked recurrent SVs to multiple first- and second-line drug resistance phenotypes, underscoring that long-read-enabled SV analysis contributes not only to structural characterization, but also to understanding clinically relevant adaptation. Taken together, these studies show that the key contribution of LRS is not merely improved contiguity, but the ability to recover chromosome-scale structural change with sufficient precision to study its functional and evolutionary consequences.

2.4 Precise localization of genetic elements through complete genomes

The precise localization of MGEs provides a critical window into understanding microbial organisms. Once chromosomes and accessory replicons are fully assembled, genetic elements can be assigned to a defined chromosomal or plasmid backbone.

Sheppard et al. applied LRS to 17 blaKPC-positive Enterobacteriaceae isolates. This enabled precise localization of blaKPC and its associated transposon Tn4401, clarifying the linked roles of blaKPC-bearing host strain transmission, plasmid transfer across strains and species, and Tn4401 transposition among plasmids in blaKPC dissemination[52]. Beyond resistance spread, complete genome resolution of the probiotic *Lactobacillus crispatus* precisely anchored Tn7088, a novel integrative mobile element encoding a class I bacteriocin biosynthetic gene cluster that drives antimicrobial competition and probiotic fitness[53]. At a larger scale, Benler et al. delineated 6,549 Tn7-like transposons at base-pair resolution and showed that 96% carried cargo genes enriched not only in defense and antibiotic resistance functions but also in central carbon metabolism[54]. Together, these findings show that complete genome assemblies both recover more sequence, and reveal the genomic context required to understand mobility and adaptation.

3. Genome-resolved metagenomics of microbial communities

Metagenomics has vastly expanded our understanding of the diversity and metabolic potential of uncultured microorganisms. Yet many ecological and functional questions require more than detecting genes or taxa alone. They depend on knowing which genome carries a given functional locus and what neighboring genes or mobile elements surround it. Recent improvements in the throughput and raw-read accuracy of LRS, together with long-read-aware assemblers, polishers and binning frameworks, have begun to relax this constraint[25, 55]. These developments enable long-read metagenomics to recover complete or near-complete genomes, distinguish

closely related strains, and determine where functional loci and integrated mobile elements occur within assembled genomes (Figure 2 and Table 2).

Figure 2 Applications of long-read metagenomics in microbial ecosystems. This schematic delineates the multidimensional analytical capacities unlocked by LRS to overcome the limitations of conventional metagenomics. LRS facilitates the reconstruction of circular metagenome-assembled genomes (cMAGs), expanding genome-resolved phylogenetic diversity by generating complete or near-complete genome representatives that can be placed in phylogenomic frameworks and linked to functional elements. Concurrently, the recovery of intact functional loci, such as biosynthetic gene clusters (BGCs) and CRISPR systems, enables robust metabolic and functional profiling. Strain-level resolution further allows for the dynamic monitoring of strains. Furthermore, LRS supports evidence-based host-MGE association by resolving integration junctions and improving phage-host inference, while complementary strategies provide evidence for assigning plasmids, phages and ARG-bearing elements to likely hosts.

Alt text: A four-panel schematic illustrating the impact of LRS on metagenomics. It highlights the transition from contigs to cMAGs for diversity expansion, the recovery of intact functional loci for metabolic profiling, strain-level tracking of structural variations, and the establishment of host-element linkages to map horizontal gene transfer networks.

3.1 Circular metagenome-assembled genomes

Complete or near-complete metagenome-assembled genomes (MAGs) recovered directly from microbial communities provide genome-resolved references for metabolic reconstruction, comparative genomics, and ecological interpretation. Early nanopore-based studies showed that closed bacterial genomes could be recovered directly from human faecal metagenomes, indicating that complete community genomes were no longer restricted to isolate sequencing[56]. At population scale, this additional continuity translates directly into a more complete view of microbiome diversity. In a study of Southeast Asian gut microbiomes integrating LRS with Hi-C, a proximity ligation method used to link DNA fragments from the same cell, more than 1,700 MAGs were absent from short-read-only analyses, assembly contiguity improved by more than 28-fold, and thousands of population-specific strains and biosynthetic gene clusters were recovered[57]. Similar gains have been reported outside the human gut. In activated sludge, hybrid long- and short-read approaches generated more than 1,000 high-quality MAGs, including dozens of circular genomes, and restored full-length rRNA genes that are typically absent from short-read MAGs[58]. More recently, deep long-read surveys across terrestrial habitats recovered genomes from 15,314 previously undescribed microbial species, expanding the phylogenetic diversity of the prokaryotic tree of life by 8%. These assemblies also recovered thousands of complete rRNA operons, biosynthetic gene clusters (BGCs), and CRISPR-Cas systems[59]. Together, these findings indicate that long-read

metagenomics can improve assembly of known taxa, recover poorly represented lineages, and support systematic discovery of repetitive and functionally important elements involved in microbial adaptability and evolution.

3.2 Strain-level resolution

Long-read metagenomics is also redefining strain resolution in microbial communities, particularly when multiple closely related lineages coexist within the same sample. Mechanistically, individual long reads link SNVs, small indels, and SVs over long genomic intervals, thereby preserving haplotypic linkage and improving strain resolution. Long reads can also preserve the genomic context of structurally variable accessory loci, including BGCs, prophages, CRISPR arrays and IS-flanked islands[25, 60, 61]. Recent algorithmic advances further improve practical access to this resolution. Myloasm uses polymorphic k-mers and high-resolution string graphs for modern PacBio HiFi and ONT R10.4 long reads, improving the recovery of complete circular contigs and previously inaccessible within-species diversity in metagenomic datasets[62]. These principles have been demonstrated in animal and human gut microbiomes. In sheep faecal microbiomes, LRS recovered lineage-resolved cMAGs and revealed hidden population structure, while improving assignment of BGCs and mobile elements to specific hosts[61]. In the human gut microbiome, long-read analyses further showed that SV is highly individualized and contributes substantially to within-species diversity[60]. Together, these studies show that strain-level interpretation often depends on multi-kilobase genomic segments that are poorly represented in short-read consensus assemblies.

The clinical relevance of this enhanced resolution is becoming increasingly apparent. In faecal microbiota transplantation (FMT), therapeutic interpretation depends not only on whether a species is detected after treatment, but also on which donor strain engrafts and persists in the recipient gut. Long-read metagenomics enables post-FMT strain tracking with substantially greater specificity than short-read approaches, particularly when multiple conspecific strains coexist in the same sample. In cohorts with recurrent *Clostridioides difficile* infection and inflammatory bowel disease, long-read analyses identified hundreds of engrafted donor strains and tracked their persistence for up to five years, while also capturing epigenetic stability and large-scale SVs acquired during adaptation to the recipient environment[63]. In a paediatric undernutrition cohort, Minich et al. used long-read metagenomics to follow the gut microbiota over 11 months and linked SV in commensal strains to host growth-associated phenotypes, suggesting that clinically relevant microbiome signals may depend on fine-scale genomic plasticity rather than taxonomic composition alone[64]. These findings indicate that strain resolution should not be viewed as a technical refinement of species-level profiling, but as a shift towards a more appropriate biological scale. Many clinically and ecologically relevant processes, including engraftment, persistence, competition and adaptation, operate at the level of strains rather than species. Long-read metagenomics therefore offers a framework in which microbial population dynamics can be interpreted in terms of inheritance, selection and genome plasticity, rather than inferred indirectly from abundance changes alone.

3.3 Establishing host-element linkages

One of the most important contributions of LRS to microbial ecology is its ability to bridge the assembly gap between MGEs and their host chromosomes (Figure 2). In short-read datasets, researchers can quantify the abundance of these elements, yet still lack direct evidence for their host range or transfer potential. LRS can strengthen host-MGE analysis in two distinct ways. For integrated MGEs, including prophages, reads or contigs that span the element-chromosome junction provide direct sequence evidence for the host genome[25, 65]. In addition, longer viral and bacterial contigs can improve phage-host association by recovering more complete viral genomes, CRISPR arrays and host taxonomic signals. For example, viralFlye showed that long-read assemblies increased CRISPR-based virus-host predictions and improved host assignment precision relative to short-read assemblies[66]. Extrachromosomal plasmids are often harder to assign because they lack obligatory chromosomal junctions, have mosaic sequence composition and may circulate across multiple hosts. Therefore, plasmid-host and ARG-bearing MGE assignments in metagenomes often require orthogonal evidence, such as Hi-C/3C proximity ligation, methylation-aware binning, or ultra-long reads when they span integration or cointegration junctions[25, 67].

Beyond host assignment, LRS is reshaping mobilome discovery by recovering complete extrachromosomal elements and resolving their structural diversity. In the human gut, long-read metagenomics enabled direct recovery of complete extrachromosomal MGEs, revealing plasmids and phage-associated DNA that are

underrepresented in short-read catalogues[68]. In the oral microbiome, long-read assembly uncovered a family of giant plasmid-like extrachromosomal elements associated with *Streptococcus*, indicating that mobile gene pools in host-associated communities have been substantially underestimated[69]. Long reads have also sharpened analyses of phage ecology. In a two-year longitudinal study of the human gut microbiome, Wirbel et al. found that prophage-host relationships were more flexible than predicted by a strictly host-specific model: bacterial lineages with and without particular prophages could coexist stably, and some phages were detected in distinct host lineages across family boundaries[15]. The same study identified a class of phages termed "IScream" phages, which appear to exploit bacterial IS30 transposases to mediate their own mobility and integration. These findings demonstrate that the mobilome is more dynamic and cross-lineage than previously appreciated, and that LRS is becoming an important tool for resolving this complexity.

The advantage of LRS is particularly valuable in resistome analysis. In an early proof-of-principle study, combining long-read assembly with proximity ligation substantially increased recovery of ARGs and virus-host associations in the rumen microbiome relative to short-read assembly alone[70]. Works in wastewater treatment plants, activated sludge systems and urban sewage networks showed that LRS can determine whether specific ARGs are stably chromosome-encoded or carried by conjugative plasmids, while resolving how host associations and mobility profiles change along environmental gradients[71-73]. Long-read datasets have likewise

clarified the effects of local selection pressures on MGE dynamics. In hospital wastewater, chlorine disinfection was associated with altered community structure, increased ARG diversity and higher mobility potential within the resistome[74]. In microplastic-associated biofilms, plasmid-borne ARGs were enriched relative to surrounding water and natural rock substrates, suggesting that artificial polymer surfaces may act as hotspots for plasmid-mediated resistance transfer[75]. Together, these studies show that LRS improves resistome and mobilome analysis by placing functional genes in their genomic background. In short-read datasets, detection of an ARG, virulence gene, or prophage often shows only that the sequence is present. LRS can further indicate whether these loci are located on chromosomes, plasmids, or transposons, which is essential for assessing their host association and potential mobility.

3.4 Pathogen genomics and real-time detection

ONT-based LRS provides a culture-independent route from clinical specimens to pathogen information, making it an important translational extension of genome-resolved microbial genomics. Its distinctive advantage lies in the ability to generate analyzable reads during the run. Reads can be classified during sequencing, allowing pathogen detection and resistance-gene screening to begin before the sequencing run is complete. In lower respiratory infection, Charalampous et al. developed a host-depleted nanopore metagenomic workflow that produced results within 6 h from sample to result, detected bacterial pathogens with 96.6% concordance with culture, and accurately detected antibiotic-resistance genes[76]. In

infected body fluids, Gu et al. evaluated clinical metagenomic next-generation sequencing using both Illumina and nanopore platforms. With the nanopore platform and real-time computational analysis, pathogens were identified after a median of 50 min of sequencing within a 6 h sample-to-answer workflow[77]. More recently, Alcolea-Medina et al. developed a unified host-depletion and nanopore workflow for respiratory samples that detected RNA and DNA viruses, bacteria, atypical bacteria and fungi, generated first automated reports after 30 min of sequencing within a 7 h workflow, and achieved prospective bacterial detection sensitivity and specificity of 90% and 100% after 2 h of sequencing[78]. This study also showed that genome recovery was feasible for a subset of reported organisms after longer sequencing, supporting the potential of nanopore metagenomics to move beyond detection toward strain typing and resistance or virulence analysis when pathogen load and read depth are sufficient[78]. These studies indicate that nanopore-based LRS is most relevant when culture is slow, antibiotic exposure may reduce culture sensitivity, or rapid resistance information is needed. Clinical implementation nevertheless remains constrained by host-background DNA, contamination control, pathogen-load thresholds, database interpretation, genotype-phenotype concordance for antimicrobial resistance, and the need for prospective validation.

4. Extended applications: multi-omics beyond DNA sequences

Genome continuity alone is insufficient to explain microbial phenotypes such as antibiotic resistance, virulence, and environmental adaptability. Epigenetic modifications and dynamic transcriptomic regulation provide additional layers linking

genotype to phenotype. SRS presents inherent limitations in these dimensions. This section systematically elucidates the technical advantages and frontier applications of LRS in microbial epigenomics and full-length transcriptomics (Figure 3).

Figure 3 Paradigm shifts in microbial multi-omics driven by single-molecule sequencing. This schematic contrasts the analytical mechanisms of conventional assays against single-molecule technologies across two core omics dimensions. Epigenomics: Conventional epigenetic profiling necessitates chemical conversions (e.g., bisulfite treatment), which induce severe DNA damage and sequence bias. In contrast, LRS enables the direct detection of diverse native modifications (such as 6mA, 5mC, and 4mC) via nanopore current disruptions. Transcriptomics: SRS makes it difficult to recover contiguous linkage within polycistronic operons. Conversely, LRS achieves full-length transcript identification, capturing contiguous RNA molecules spanning intact multi-gene organizations from transcriptional start sites (TSSs) to transcriptional termination sites (TTSs).

Alt text: A two-panel schematic comparing conventional and single-molecule multi-omics. It illustrates the shift from chemical-based epigenetics to direct modification sensing, and from fragmented transcripts to full-length operon sequencing.

4.1 Microbial epigenomics: from native signals to regulatory landscapes

Microbial epigenetic modifications, predominantly N6-methyladenine (6mA), 5-methylcytosine (5mC), and N4-methylcytosine (4mC), are associated with restriction-modification (R-M) systems, gene expression, and host-pathogen interactions. Historically, SRS based approaches for DNA methylation detection have often relied on bisulfite chemical conversion, which can cause DNA degradation and sequence bias. Because methylation profiles are inferred from aggregated sequencing reads, these approaches generally provide population-level methylation profiles rather than direct measurements of individual DNA molecules. In contrast, LRS preserves native DNA molecules and enables direct modification detection: PacBio SMRT utilizes polymerase kinetic characteristics, while ONT detects changes in nanopore ionic current. Both platforms enable simultaneous DNA sequencing and single-molecule methylation detection without PCR amplification or chemical conversion, allowing direct linkage of epigenetic modifications to their genomic context on individual DNA molecules[79, 80] (Figure 3). Today, LRS is extensively applied to methylation detection, complete R-M system resolution, and the linking of epigenetics to phenotypic regulation. Notably, the two platforms exhibit divergent strengths: PacBio HiFi currently provides high sensitivity and confidence for 6mA detection, whereas recent developments including the R10.4.1 pore and improved basecalling models have drastically improved 5mC and 4mC detection, making it particularly attractive for cost-effective, large-scale meta-epigenomic studies[81, 82].

Compared with SRS, LRS can associate methylation motifs with cognate methyltransferases across the same long DNA molecules[83]. This precise anchoring

allows for the resolution of R-M systems carried by plasmids, revealing their core roles in restriction of HGT and phage defense[84, 85]. Furthermore, LRS maps methylation modifications onto specific functional elements (promoters, operons, and MGEs), identifying potential regulatory sites of single-base modifications on gene expression. In microbial communities, long-read metagenomic methylation profiling improves strain-level resolution and assignment of plasmids and phages to their hosts through shared methylation signatures[86].

However, several challenges remain. Accurate long-read metagenomic methylation analysis depends on the recovery of high-molecular-weight (HMW) DNA, which is required to generate long reads and contiguous assemblies that enable reliable assignment of methylation motifs to chromosomes, plasmids, and phages. In addition, the methylation characterization of low-abundance taxa remains limited by sequencing depth. Ding et al. found that at the 25× sequencing depth recommended by PacBio, only 16.24% of phage genomes met this requirement, and the number of detected methylation sites increased with deeper sequencing[87]. Furthermore, different types of methylation modifications exhibit distinct depth requirements. For instance, 40× depth is sufficient to profile most 6mA sites, whereas 4mC resolution requires at least 60× depth to achieve comparable sensitivity[88].

4.2 Full-length transcriptomics: reshaping operons and isoforms

Bacterial transcriptomes are substantially organized around polycistronic operons and contain a rich repertoire of alternative transcripts, antisense RNAs

(asRNAs), and non-coding RNAs that collectively coordinate environmental adaptation, stress responses, and virulence. Traditional short-read RNA sequencing often fragments transcripts, hindering accurate reconstruction of complete transcriptional units (Figure 3). LRS can generate reads that span entire RNA molecules in a single pass[85, 89].

A major advantage of LRS is its ability to precisely delineate operon architecture, including transcriptional start sites (TSSs), transcriptional termination sites (TTSs), internal promoters and co-transcribed gene clusters. By linking multiple genes within individual polycistronic transcripts, LRS transforms inferred operon models into experimentally validated maps of active transcription units[90, 91]. Furthermore, LRS provides unambiguous information to accurately annotate regulatory elements such as asRNAs, as well as overlapping genes, which typically pose challenges for short‑read‑based assembly[92]. For instance, ONT direct RNA sequencing refined the transcriptional boundaries of previously identified operons, revealing extended boundaries for 225 operons in *E. coli* and 89 operons in *S. aureus*[93]. Moreover, direct RNA sequencing enables the detection of native RNA modifications, such as m5C and m6A, providing new insights into the complexity of post-transcriptional regulation in bacteria[94]. In a community context, integrating metatranscriptomics with nanopore long-read metagenomics enabled the reconstruction of trimethylamine metabolic networks and the identification of key microbial taxa[95]. Nevertheless, applying LRS in the microbiome faces several technical challenges. Full‑length RNA recovery is difficult due to rapid RNA degradation and inhibitory compounds (e.g.,

humic acids in soil)[96]. rRNA depletion is another essential step, as rRNA typically constitutes more than 90% of total bacterial RNA[97]. Assignment of transcripts to specific strains represents a third barrier, owing to high sequence similarity among closely related species and the lack of reference genomes[98].

5. Multidimensional technical challenges in LRS

As delineated in preceding sections, LRS has promoted a shift across isolate genomics, metagenomics, and multi-omics dimensions, elevating the analytical resolution of microbiology. Nevertheless, despite its exceptional potential to reconstruct genomic architectures, the systematic application of LRS in large-scale sequencing studies remains impeded by cost-effectiveness limitations[22, 99]. Crucially, this economic burden extends far beyond direct sequencing expenses (such as reagents and instruments)[100], it is rooted in systemic technical limitations that affect the entire experimental workflow (Table 3), spanning sample preparation, data acquisition, and bioinformatic assembly.

Table 3 Comparison of short- and long-read sequencing platforms

	SRS		LRS		
	Illumina (NovaSeq X)	MGI (DNBSEQ-T20)	PacBio (Revio)	ONT (PromethION 24)	MGI (CycloneSEQ-G400-ER)
DNA input requirements	100 ng	200 ng	500 ng	1 μg	500 ng
Read Length	2×150 bp	2×150 bp	15–20 kb	20 bp - >4	100 bp

				Mb	- >1Mb
Raw accuracy	>99.9%	>99.9%	~99.9%	~99%	~97%
Throughput	2 × (8-10.5)Tb	6 × 12 Tb	4 × (100-120)Gb	24 × (100-200)Gb	1 × 400 Gb
Runtime	48 h	24~30 h	24 h	72 h	72 h

5.1 Sample preparation constraints in LRS

By preserving native nucleic acid information, LRS circumvents the sequence biases and errors introduced by PCR amplification in SRS[101], representing an important advance in genomic research. However, while LRS increases read lengths (Table 3), it simultaneously imposes stringent requirements on upstream sample preparation[22, 102]. This requirement for high purity and HMW DNA is a major technical challenge limiting the accessibility of LRS for complex environmental niches.

First, LRS is sensitive to co-purified impurities. In traditional SRS library construction, the PCR amplification process dilutes easily co-precipitated inhibitors, such as polysaccharides and bile acids in gut samples, or residual extraction reagents[103]. Conversely, the amplification-free nature of LRS allows these contaminants to persist in the final library. These impurities inhibit single-molecule polymerase activity or obstruct nanopores, leading to premature termination of the sequencing reaction[102, 103]. This issue is pronounced in complex environmental samples such as soil, where abundant humic acids, sharing chemical structural

similarity with nucleic acids, are readily co-purified. Attempts to remove these impurities through additional purification steps may result in fragmentation of the DNA molecules[104]. Second, the extraction of HMW DNA confronts a fundamental paradox. Even routine extraction procedures, including pipette aspiration, column purification, or mild vortexing, generate physical shear forces to induce the breakage of long DNA fragments[105, 106]. Within microbiome research, this challenge is substantially amplified. Due to the robust peptidoglycan architecture characteristic of many bacteria, gentle enzymatic lysis protocols designed to preserve the DNA backbone fail to lyse Gram-positive bacteria, resulting in bias and loss of taxonomic representation within the community[107]. Conversely, adopting bead-beating methods to ensure comprehensive and unbiased lysis tend to produce fragmented DNA extracts, which limits the advantages of LRS[103].

Finally, low-biomass samples present an additional challenge. For samples characterized by low microbial abundance, the yield from conventional extraction methods may not meet the input requirements of LRS platforms (Table 3)[25, 108]. As demonstrated by Serghiou et al. in skin microbiomes and Child et al. in environmental soils, standard commercial kits often fail to balance yield and integrity in these niches, recovering only small amounts of fragmented DNA[109, 110]. Determining how to secure adequate extraction recovery rates from low initial biomass while simultaneously preserving DNA length and purity remains a technical issue in the field.

5.2 Inherent limitations of data throughput and base-calling accuracy

Beyond sample preparation requirements, the limitations of LRS regarding data yield and sequencing depth also restrict its application. Compared to the terabase-scale single run throughput characteristic of SRS platforms, current LRS platforms exhibit a disadvantage in data output per unit cost. For instance, the maximum throughput per flow cell for PacBio and ONT platforms is approximately 100 to 200 Gb, whereas Illumina systems can exceed 8 Tb (Table 3). This throughput limitation causes insufficient resolution for complex samples, which leads to subsequent analytical challenges.

First, this throughput deficit increases the dominance of highly abundant species over low abundance taxa in the microbial communities. In complex habitats, most sequencing reads originate from dominant microbes. Consequently, due to insufficient overall depth, the probability of capturing species with relative abundances below 1% is substantially reduced[111]. Second, insufficient depth limits the assembly of uncultured microorganisms and leads to information loss. For uncultured taxa lacking reference genomes, the inability to achieve the target coverage requisite for *de novo* assembly makes it difficult to generate high-quality closed genomes or extensive contiguous sequences. Consequently, a massive proportion of the sequencing data remains fragmented, exhibiting low completeness scores upon evaluation with algorithms such as CheckM2, and is ultimately discarded as invalid data during the binning phase[112].

Furthermore, the errors associated with LRS in homopolymer regions also lead to downstream analytical challenges. Constrained by resolution limits of nanopore

electrical signals or the kinetic characteristics of single-molecule polymerases, LRS are prone to insertion or deletion errors across consecutive identical bases[102]. Notably, these specific inaccuracies account for over 35% of the total sequencing error profile[113]. In metagenomic samples characterized by low sequencing depth and lacking supplemental error correction from SRS data, these non-random errors may induce frameshift mutations within protein coding regions[114, 115]. Such sequence alterations affect accurate prediction of open reading frames and subsequent functional gene annotation.

5.3 Algorithmic burdens in LRS

The convergence of algorithmic architectures and intrinsic sequencing error rates leads to substantial computational demands during the genomic assembly phase, particularly for metagenomes. Although OLC has a higher tolerance for sequencing inaccuracies[116], establishing topological relationships between long reads requires pairwise all-vs-all alignments. This alignment mechanism increases computational overhead; for instance, during the assembly of the HG002 human genome, the alignment stage accounted for approximately 79% of the total execution time[117, 118].

Furthermore, when processing metagenomic samples, contemporary assembly tools generate structural defects despite the allocation of massive computational resources. Evaluations indicate that prominent long-read metagenomic assemblers, including HiCanu, metaFlye, hifiasm-meta and metaMDBG[119-122], generate an average of over 40 errors per 100 megabases of contiguous sequences. These

inaccuracies transcend simple base level substitutions and manifest as complex structural anomalies, such as chimeras and prematurely circularized sequences[123].

6. Current sequencing choices and future perspectives

While recent reviews, such as the work by Agustinho et al., focus primarily on computational workflows and software tools used for long-read data analysis, our review centers on practical applications across biological scales. In this applied context, the optimal choice among SRS, LRS, and hybrid sequencing is now best defined by the biological question, sample complexity, and accuracy requirement. SRS remains widely used in taxonomic profiling, abundance estimation, and large cohort studies. For isolated bacterial genomes, recent PacBio HiFi and ONT R10/R10.4.1 workflows have shown that complete or near-complete chromosomes and plasmids can often be reconstructed without mandatory short-read polishing, provided that coverage, read length, and quality-control metrics are adequate[102, 124-126]. This does not make hybrid sequencing obsolete. Instead, hybrid approaches remain particularly useful when the study requires maximal consensus accuracy, conservative SNP or indel interpretation, recovery of difficult plasmids, validation of problematic low-coverage, and homopolymer-rich regions[106, 125-127]. In metagenomics, LRS-only approaches are increasingly powerful for cMAG recovery and structural resolution, but uneven species abundance, rare community members, strain mixtures and assembly artefacts still make short-read supplementation or other orthogonal data valuable in selected settings[54, 121-123, 128-130].

What is changing most rapidly now is the accuracy. PacBio HiFi sequencing

showed that long reads can reach a level of accuracy suitable for both assembly and variant analysis[102]. Subsequent microbial studies showed that complete or near-complete genomes can often be recovered from isolates and microbiomes without mandatory short-read correction[55, 121, 126]. On the ONT side, R10.4.1 chemistry and improved basecalling have narrowed the historical gap in bacterial genome reconstruction, especially at sufficient coverage[125]. The field is therefore moving toward Q30-class long reads, corresponding to approximately one error per 1,000 bases, as a realistic standard in optimized workflows. Based on this, Q40-grade reads, corresponding to approximately one error per 10,000 bases, are also becoming plausible[106]. On the other hand, ultra-long reads matter not simply because they are longer, but because they can span the regions that still create ambiguity in microbial assembly[131, 132]. Once such regions are bridged by single molecules, some assembly problems no longer need to be solved indirectly through graph inference. Even so, this will depend on more than sequencing chemistry. It will also require reliable extraction of HMW DNA from difficult samples and further reduction of systematic error.

At the same time, LRS is now limited as much by interpretation as by data generation. This is especially clear in metagenomics, where assembly still has to accommodate extreme abundance variation, microdiversity, shared repeats, and very large datasets. Recent assemblers such as metaMDBG, nanoMDBG, and myloasm show that algorithmic redesign can still improve memory efficiency, runtime, and recovery of cMAGs, plasmids, and viruses[62, 122, 133]. Binning is evolving in the

same way. LorBin and recent cross-platform benchmarks indicate that long-read-aware and hybrid-aware strategies can substantially improve the recovery of high-quality genomes from communities[134, 135]. However, continuity does not guarantee correctness. Long-read metagenome assemblies can still contain chimeras, false duplications, haplotyping artefacts, and premature circularization[123]. This point matters because future tools will need to support automated validation, and better distinguish true biological microbial diversity from assembly artefact. Machine-learning and deep-learning methods are likely to contribute to this process. For instance, recent studies show that deep learning underlies modern nanopore basecalling, affects read accuracy and computational cost, and can further support microbial viability inference from raw nanopore signals[136-138]. Similar approaches are likely to influence methylation calling, assembly validation, binning, and cross-omic integration. If LRS is to become routine rather than specialist, these analytical advances will also need to be matched by lower cost, more standardized workflows, and greater automation[139].

Recent advances in nanopore protein sequencing have demonstrated its feasibility for single-molecule proteomics, including repeated reading of single polypeptides, single-amino acid sensitivity, and peptide discrimination for protein identification[140, 141]. This platform offers potential advantages over conventional mass spectrometry, as it does not require enzymatic digestion, enabling the direct characterization of proteoforms and post-translational modifications at single-molecule resolution[140, 142]. Although direct nanopore proteomics has not yet been

applied to isolated bacterial strains or complex metagenomic samples, its prospective utility for microbiomics is substantial. In community settings, reading complete polypeptides could resolve the pervasive challenge of peptide sharing, where identical short sequences cannot be uniquely assigned among closely related strains.

The broader significance of LRS may become clearest in multi-omics studies (Figure 4). Its main strength is that it preserves molecular context, allowing genome structure to be linked with DNA methylation, transcription, and, indirectly, protein and metabolite profiles. In bacteria, long-read transcriptomics has already shown that transcript organization is more complex than suggested by fragment-based RNA sequencing, revealing extended operons and substantial transcriptional heterogeneity[85]. More recently, nanopore direct RNA sequencing has begun to support joint analysis of the bacterial transcriptome and epitranscriptome within the same experiment[93]. LRS is also becoming increasingly important for epigenomic profiling and transcript-level analysis more broadly[143, 144]. The real opportunity for microbiology lies in combining these measurements with genome-resolved metagenomics, metatranscriptomics, metaproteomics, and metabolomics. Standardized multi-omics studies have already shown that linked analysis of genomes, functions, and metabolites can reveal ecological structure that is invisible to any single modality[145-147]. In this setting, LRS could serve as the scaffold that anchors different measurements to the same genome or strain. That would make it easier to ask which lineages are active, which elements are mobilized, which methylation states accompany regulatory change, and how these properties shift over time or under

perturbation. In the longer term, such a framework may support models that monitor strain turnover, improve prediction of engraftment or instability, and provide earlier warning of resistance emergence or community collapse. For this reason, the future value of LRS is unlikely to be defined by assembly alone. Its deeper contribution may be to help microbial genomics move from increasingly complete description toward a more mechanistic and predictive framework.

Figure 4 Future perspectives of long-read microbial genomics. (a) Technological and algorithmic evolution. High-accuracy LRS and emerging ultra-long sequencing approaches (>100 kb) are expanding comprehensive resolution across isolate genomics and metagenomics, with hybrid sequencing retained for depth-limited or accuracy-critical applications. Concurrently, continuous advancements in sequencing platforms are systematically driving raw accuracy towards Q30 and Q40 standards. Machine learning and deep learning methods are improving basecalling accuracy, genome binning, assembly validation, and computational efficiency. (b) Towards multi-omics integration. LRS empowers applications across the epigenome, transcriptome, mobilome, proteome, and metabolome. This integration supports more complete descriptions of genomic architectures, a mechanistic understanding of ecological interplay, and predictive models for microbial community dynamics.

Alt text: A two-panel conceptual diagram illustrating the future of long-read microbial genomics. Panel (a) shows hybrid and ultra-long sequencing technologies, rising raw

accuracy, and machine-learning and deep-learning approaches for basecalling, binning, and validation. Panel (b) depicts the integration of multiple omics layers to drive complete genomic descriptions, mechanistic understanding, and predictive models for microbial communities.

7. Conclusion

This review provides a systematic evaluation of the application potential and challenges of LRS within microbial genomics. LRS overcomes the inherent limitations of sequence fragmentation and amplification bias. This technological advancement not only facilitates the reconstruction of complete isolate genomes and precise localization of genetic elements, but also enhances metagenomics by enabling the recovery of cMAGs, strain resolved analysis, and the linkage of MGEs to their specific replicons. Furthermore, LRS extends microbial analysis into multi-omics dimensions by directly detecting epigenetic modifications and capturing full-length transcripts. However, LRS remains constrained by challenges, primarily the requirements for HMW DNA extraction, sequencing depth deficits, and substantial computational burdens. Currently, while high-accuracy LRS is increasingly preferred for isolate genomics and metagenomics, hybrid sequencing is retained for depth-limited, difficult-replicon, rare-taxon, or accuracy-critical applications. Looking forward, ongoing advancements in sequencing accuracy, efficient bioinformatic tools, and multi-omics integration will further propel LRS to decode global microbial diversity and map the architecture of microbial ecosystems.

Data Availability

Not applicable

List of abbreviations

ARG: antibiotic resistance gene; BGC: biosynthetic gene cluster; cMAG: circular metagenome-assembled genome; DNBSEQ: DNA nanoball sequencing; FMT: fecal microbiota transplantation; GC: guanine–cytosine; HGT: horizontal gene transfer; HMW DNA: high-molecular-weight DNA; IS: insertion sequence; LRS: long-read sequencing; MAG: metagenome-assembled genome; MGE: mobile genetic element; OLC: overlap layout consensus; ONT: Oxford Nanopore Technologies; R-M: restriction-modification; rRNA: ribosomal RNA; SNV: single nucleotide variant; SRS: short-read sequencing; SV: structural variant; TSS: transcription start site; TTS: transcription termination site.

Competing interests

The CycloneSEQ was initially developed by BGI Research and is now being marketed as an advanced technology in MGI. All authors except Yuzheng Gu are employees of BGI Research. The authors declare that this affiliation did not influence the selection, interpretation, or presentation of the literature reviewed in this article.

Funding

This work was supported by the financial support from Shenzhen Science and Technology Program (No. KCXFZ20240903094006009) and Shenzhen Medical Research Fund (No. JCYJ20241202124801003).

Authors' contributions

Xing Rao (Investigation, Writing—original draft, Visualization, Writing—review & editing), Yuzheng Gu (Investigation, Writing—original draft, Visualization, Writing—review & editing), Gabriella (Writing—original draft), Jiayi Ma (Writing—original draft), Haoyu Wang (Investigation, Visualization), and Yuanqiang Zou (Conceptualization, Funding acquisition, Project administration, Supervision, Writing – review & editing).

Acknowledgements

This study is supported by grants from Shenzhen Science and Technology Program (No. SYSPG20241211173845014).

Disclosure of use of AI-assisted tools including generative AI

The authors used Gemini 3.1 Pro as an auxiliary tool during manuscript preparation in April 2026[148]. The tool assisted with grammar checking and wording suggestions. It was not used to generate original scientific content, figures, tables, references, or conclusions. All AI-assisted suggestions were reviewed and revised by the authors.

References

1. Sanger F, Nicklen S, Coulson AR. DNA sequencing with chain-terminating inhibitors. *Proc Natl Acad Sci U S A*. 1977; doi: 10.1073/pnas.74.12.5463.
2. Fleischmann RD, Adams MD, White O, et al. Whole-genome random sequencing and assembly of Haemophilus influenzae Rd. *Science*. 1995; doi: 10.1126/science.7542800.
3. van Dijk EL, Auger H, Jaszczyszyn Y, et al. Ten years of next-generation sequencing technology. *Trends Genet*. 2014; doi: 10.1016/j.tig.2014.07.001.
4. Mak SST, Gopalakrishnan S, Caroe C, et al. Comparative performance of the BGISEQ-500 vs Illumina HiSeq2500 sequencing platforms for palaeogenomic sequencing. *Gigascience*. 2017; doi: 10.1093/gigascience/gix049.
5. Merriman B, Ion Torrent R, Team D, et al. Progress in ion torrent semiconductor chip based sequencing. *Electrophoresis*. 2012; doi: 10.1002/elps.201200424.
6. Previte MJ, Zhou C, Kellinger M, et al. DNA sequencing using polymerase substrate-binding kinetics. *Nat Commun*. 2015; doi: 10.1038/ncomms6936.
7. Human Microbiome Project C. A framework for human microbiome research. *Nature*. 2012; doi: 10.1038/nature11209.
8. Qin J, Li R, Raes J, et al. A human gut microbial gene catalogue established by metagenomic sequencing. *Nature*. 2010; doi: 10.1038/nature08821.
9. Mandelker D, Schmidt RJ, Ankala A, et al. Navigating highly homologous genes in a molecular diagnostic setting: a resource for clinical next-generation sequencing. *Genet Med*. 2016; doi: 10.1038/gim.2016.58.
10. Schmid M, Frei D, Patrignani A, et al. Pushing the limits of de novo genome assembly for complex prokaryotic genomes harboring very long, near identical repeats. *Nucleic Acids Res*. 2018; doi: 10.1093/nar/gky726.
11. Achaz G, Rocha EP, Netter P, et al. Origin and fate of repeats in bacteria. *Nucleic Acids Res*. 2002; doi: 10.1093/nar/gkf391.
12. Hill CW. Large genomic sequence repetitions in bacteria: lessons from rRNA operons and Rhs elements. *Res Microbiol*. 1999; doi: 10.1016/s0923-2508(99)00125-4.
13. Huisman JS, Vaughan TG, Egli A, et al. The effect of sequencing and assembly on the inference of horizontal gene transfer on chromosomal and plasmid phylogenies. *Philos Trans R Soc Lond B Biol Sci*. 2022; doi: 10.1098/rstb.2021.0245.
14. George S, Pankhurst L, Hubbard A, et al. Resolving plasmid structures in Enterobacteriaceae using the MinION nanopore sequencer: assessment of MinION and MinION/Illumina hybrid data assembly approaches. *Microb Genom*. 2017; doi: 10.1099/mgen.0.000118.
15. Wirbel J, Hickey AS, Chang D, et al. Long-read metagenomics reveals phage dynamics in the human gut microbiome. *Nature*. 2026; doi:

10.1038/s41586-025-09786-2.
16. Goldstein S, Beka L, Graf J, et al. Evaluation of strategies for the assembly of diverse bacterial genomes using MinION long-read sequencing. *BMC Genomics*. 2019; doi: 10.1186/s12864-018-5381-7.
17. Tilak MK, Botero-Castro F, Galtier N, et al. Illumina Library Preparation for Sequencing the GC-Rich Fraction of Heterogeneous Genomic DNA. *Genome Biol Evol*. 2018; doi: 10.1093/gbe/evy022.
18. International Chicken Genome Sequencing C. Sequence and comparative analysis of the chicken genome provide unique perspectives on vertebrate evolution. *Nature*. 2004; doi: 10.1038/nature03154.
19. Korlach J, Bjornson KP, Chaudhuri BP, et al. Real-time DNA sequencing from single polymerase molecules. *Methods Enzymol*. 2010; doi: 10.1016/S0076-6879(10)72001-2.
20. Shafin K, Pesout T, Lorig-Roach R, et al. Nanopore sequencing and the Shasta toolkit enable efficient de novo assembly of eleven human genomes. *Nat Biotechnol*. 2020; doi: 10.1038/s41587-020-0503-6.
21. Liang H, Zou Y, Wang M, et al. Efficiently constructing complete genomes with CycloneSEQ to fill gaps in bacterial draft assemblies. *GigaByte*. 2025; doi: 10.46471/gigabyte.154.
22. Logsdon GA, Vollger MR, Eichler EE. Long-read human genome sequencing and its applications. *Nat Rev Genet*. 2020; doi: 10.1038/s41576-020-0236-x.
23. Wang Y, Zhao Y, Bollas A, et al. Nanopore sequencing technology, bioinformatics and applications. *Nat Biotechnol*. 2021; doi: 10.1038/s41587-021-01108-x.
24. Marx V. Method of the year: long-read sequencing. *Nat Methods*. 2023; doi: 10.1038/s41592-022-01730-w.
25. Agustinho DP, Fu Y, Menon VK, et al. Unveiling microbial diversity: harnessing long-read sequencing technology. *Nat Methods*. 2024; doi: 10.1038/s41592-024-02262-1.
26. Fraser CM, Eisen JA, Nelson KE, et al. The value of complete microbial genome sequencing (you get what you pay for). *J Bacteriol*. 2002; doi: 10.1128/JB.184.23.6403-6405.2002.
27. Larsen MV, Cosentino S, Rasmussen S, et al. Multilocus sequence typing of total-genome-sequenced bacteria. *J Clin Microbiol*. 2012; doi: 10.1128/JCM.06094-11.
28. Shi W, Sun Q, Fan G, et al. gcType: a high-quality type strain genome database for microbial phylogenetic and functional research. *Nucleic Acids Res*. 2021; doi: 10.1093/nar/gkaa957.
29. Eisenhofer R, Odriozola I, Alberdi A. Impact of microbial genome completeness on metagenomic functional inference. *ISME Commun*. 2023; doi: 10.1038/s43705-023-00221-z.
30. Zou Y, Xue W, Luo G, et al. 1,520 reference genomes from cultivated human gut bacteria enable functional microbiome analyses. *Nat Biotechnol*. 2019; doi: 10.1038/s41587-018-0008-8.

31. Akintunde O, Tucker T, Carabetta VJ. The Evolution of Next-Generation Sequencing Technologies. *Methods Mol Biol*. 2025; doi: 10.1007/978-1-0716-4192-7_1.
32. Kim C, Pongpanich M, Porntaveetus T. Unraveling metagenomics through long-read sequencing: a comprehensive review. *J Transl Med*. 2024; doi: 10.1186/s12967-024-04917-1.
33. Athanasopoulou K, Boti MA, Adamopoulos PG, et al. Third-Generation Sequencing: The Spearhead towards the Radical Transformation of Modern Genomics. *Life (Basel)*. 2021; doi: 10.3390/life12010030.
34. Chin CS, Alexander DH, Marks P, et al. Nonhybrid, finished microbial genome assemblies from long-read SMRT sequencing data. *Nat Methods*. 2013; doi: 10.1038/nmeth.2474.
35. Koren S, Harhay GP, Smith TP, et al. Reducing assembly complexity of microbial genomes with single-molecule sequencing. *Genome Biol*. 2013; doi: 10.1186/gb-2013-14-9-r101.
36. Heng NCK, Stanton J-AL. Next-generation DNA sequencing of oral microbes at the Sir John Walsh Research Institute: technologies, tools and achievements. *Journal of the Royal Society of New Zealand*. 2019; doi: 10.1080/03036758.2019.1687530.
37. Marin-Gordillo A, Lopez-Garcia E, Romero-Gil V, et al. Hybrid genome assembly and comprehensive genomic analysis of Lactiplantibacillus pentosus LP309 reveal its probiotic and technological potential. *BMC Genomics*. 2026; doi: 10.1186/s12864-026-12678-y.
38. Wang H, Gu Y, He W, et al. Complete Genomes of Cultivated Gut Bacteria Reveal Mobile Genetic Element-Driven Functional Diversity with Therapeutic Implications. *bioRxiv*. 2026; doi: 10.64898/2026.03.25.714112.
39. Gu Y, Wang H, Yang J, et al. Complete genome-derived metabolic interactions reveal impact of gut ecology on human health. *bioRxiv*. 2026; doi: 10.64898/2026.03.25.714329.
40. Carr VR, Shkoporov A, Hill C, et al. Probing the Mobilome: Discoveries in the Dynamic Microbiome. *Trends Microbiol*. 2021; doi: 10.1016/j.tim.2020.05.003.
41. Olsen NS, Riber L. Metagenomics as a Transformative Tool for Antibiotic Resistance Surveillance: Highlighting the Impact of Mobile Genetic Elements with a Focus on the Complex Role of Phages. *Antibiotics (Basel)*. 2025; doi: 10.3390/antibiotics14030296.
42. Arredondo-Alonso S, Willems RJ, van Schaik W, et al. On the (im)possibility of reconstructing plasmids from whole-genome short-read sequencing data. *Microb Genom*. 2017; doi: 10.1099/mgen.0.000128.
43. Martinez JL, Coque TM, Lanza VF, et al. Genomic and metagenomic technologies to explore the antibiotic resistance mobilome. *Ann N Y Acad Sci*. 2017; doi: 10.1111/nyas.13282.
44. Jiang Y, Wang Y, Hua X, et al. Pooled Plasmid Sequencing Reveals the Relationship Between Mobile Genetic Elements and Antimicrobial Resistance

Genes in Clinically Isolated Klebsiella pneumoniae. *Genomics Proteomics Bioinformatics*. 2020; doi: 10.1016/j.gpb.2020.12.002.

45. McLeod A, Fagerlund A, Rud I, et al. Large Plasmid Complement Resolved: Complete Genome Sequencing of Lactobacillus plantarum MF1298, a Candidate Probiotic Strain Associated with Unfavorable Effect. *Microorganisms*. 2019; doi: 10.3390/microorganisms7080262.

46. Cottingham H, Judd LM, Harshegyi-Hand T, et al. Nanopore sequencing enables highly accurate genotyping and identification of resistance determinants in key nosocomial pathogens. *Microb Genom*. 2026; doi: 10.1099/mgen.0.001657.

47. Wick RR, Judd LM, Stinear TP, et al. Are reads required? High-precision variant calling from bacterial genome assemblies. *Access Microbiol*. 2025; doi: 10.1099/acmi.0.001025.v3.

48. Bouda E, Peng Y, Williams MM, et al. Bordetella pertussis exhibits genomic diversity within patients and laboratory culture. *mSystems*. 2025; doi: 10.1128/msystems.01717-24.

49. Waters EV, Tucker LA, Ahmed JK, et al. Impact of Salmonella genome rearrangement on gene expression. *Evol Lett*. 2022; doi: 10.1002/evl3.305.

50. Li C, Tyson GH, Hsu CH, et al. Long-Read Sequencing Reveals Evolution and Acquisition of Antimicrobial Resistance and Virulence Genes in Salmonella enterica. *Front Microbiol*. 2021; doi: 10.3389/fmicb.2021.777817.

51. Canalda-Baltrons A, Silcocks M, Hall MB, et al. Genome graphs reveal the importance of structural variation in Mycobacterium tuberculosis evolution and drug resistance. *Nat Commun*. 2025; doi: 10.1038/s41467-025-65779-9.

52. Sheppard AE, Stoesser N, Wilson DJ, et al. Nested Russian Doll-Like Genetic Mobility Drives Rapid Dissemination of the Carbapenem Resistance Gene blaKPC. *Antimicrob Agents Chemother*. 2016; doi: 10.1128/AAC.00464-16.

53. Colombini L, Santoro F, Tirziu M, et al. The mobilome of Lactobacillus crispatus M247 includes two novel genetic elements: Tn7088 coding for a putative bacteriocin and the siphovirus prophage PhiM247. *Microb Genom*. 2023; doi: 10.1099/mgen.0.001150.

54. Benler S, Faure G, Altae-Tran H, et al. Cargo Genes of Tn7-Like Transposons Comprise an Enormous Diversity of Defense Systems, Mobile Genetic Elements, and Antibiotic Resistance Genes. *mBio*. 2021; doi: 10.1128/mBio.02938-21.

55. Kim CY, Ma J, Lee I. HiFi metagenomic sequencing enables assembly of accurate and complete genomes from human gut microbiota. *Nat Commun*. 2022; doi: 10.1038/s41467-022-34149-0.

56. Moss EL, Maghini DG, Bhatt AS. Complete, closed bacterial genomes from microbiomes using nanopore sequencing. *Nat Biotechnol*. 2020; doi: 10.1038/s41587-020-0422-6.

57. Gounot JS, Chia M, Bertrand D, et al. Genome-centric analysis of short and long read metagenomes reveals uncharacterized microbiome diversity in Southeast Asians. *Nat Commun*. 2022; doi: 10.1038/s41467-022-33782-z.

58. Singleton CM, Petriglieri F, Kristensen JM, et al. Connecting structure to function with the recovery of over 1000 high-quality metagenome-assembled genomes from activated sludge using long-read sequencing. *Nat Commun*. 2021; doi: 10.1038/s41467-021-22203-2.
59. Sereika M, Mussig AJ, Jiang C, et al. Genome-resolved long-read sequencing expands known microbial diversity across terrestrial habitats. *Nat Microbiol*. 2025; doi: 10.1038/s41564-025-02062-z.
60. Chen L, Zhao N, Cao J, et al. Short- and long-read metagenomics expand individualized structural variations in gut microbiomes. *Nat Commun*. 2022; doi: 10.1038/s41467-022-30857-9.
61. Bickhart DM, Kolmogorov M, Tseng E, et al. Generating lineage-resolved, complete metagenome-assembled genomes from complex microbial communities. *Nat Biotechnol*. 2022; doi: 10.1038/s41587-021-01130-z.
62. Shaw J, Marin MG, Li H. High-resolution metagenome assembly for modern long reads with myloasm. *Nat Biotechnol*. 2026; doi: 10.1038/s41587-026-03053-z.
63. Fan Y, Ni M, Aggarwala V, et al. Long-read metagenomics for strain tracking after faecal microbiota transplant. *Nat Microbiol*. 2025; doi: 10.1038/s41564-025-02164-8.
64. Minich JJ, Allsing N, Din MO, et al. Culture-independent meta-pangenomics enabled by long-read metagenomics reveals associations with pediatric undernutrition. *Cell*. 2025; doi: 10.1016/j.cell.2025.08.020.
65. Yahara K, Suzuki M, Hirabayashi A, et al. Long-read metagenomics using PromethION uncovers oral bacteriophages and their interaction with host bacteria. *Nat Commun*. 2021; doi: 10.1038/s41467-020-20199-9.
66. Antipov D, Rayko M, Kolmogorov M, et al. viralFlye: assembling viruses and identifying their hosts from long-read metagenomics data. *Genome Biol*. 2022; doi: 10.1186/s13059-021-02566-x.
67. Beaulaurier J, Zhu S, Deikus G, et al. Metagenomic binning and association of plasmids with bacterial host genomes using DNA methylation. *Nat Biotechnol*. 2018; doi: 10.1038/nbt.4037.
68. Suzuki Y, Nishijima S, Furuta Y, et al. Long-read metagenomic exploration of extrachromosomal mobile genetic elements in the human gut. *Microbiome*. 2019; doi: 10.1186/s40168-019-0737-z.
69. Kiguchi Y, Hamamoto N, Kashima Y, et al. Giant extrachromosomal element "Inocle" potentially expands the adaptive capacity of the human oral microbiome. *Nat Commun*. 2025; doi: 10.1038/s41467-025-62406-5.
70. Bickhart DM, Watson M, Koren S, et al. Assignment of virus and antimicrobial resistance genes to microbial hosts in a complex microbial community by combined long-read assembly and proximity ligation. *Genome Biol*. 2019; doi: 10.1186/s13059-019-1760-x.
71. Dai D, Brown C, Burgmann H, et al. Long-read metagenomic sequencing reveals shifts in associations of antibiotic resistance genes with mobile genetic elements from sewage to activated sludge. *Microbiome*. 2022; doi:

10.1186/s40168-021-01216-5.

72. Ding J, Wang M, Xu X, et al. Highly Resolved Community Sewage Metagenomics Unveiling Landscape and Transmission Patterns of Antibiotic Resistome in Hong Kong Populations. *Adv Sci (Weinh)*. 2026; doi: 10.1002/advs.202508389.

73. Mao X, Yin X, Yang Y, et al. Longitudinal metagenomic analysis on antibiotic resistome, mobilome, and microbiome of river ecosystems in a sub-tropical metropolitan city. *Water Res*. 2025; doi: 10.1016/j.watres.2025.123102.

74. Rolbiecki D, Paukszto L, Krawczyk K, et al. Chlorine disinfection modifies the microbiome, resistome and mobilome of hospital wastewater - A nanopore long-read metagenomic approach. *J Hazard Mater*. 2023; doi: 10.1016/j.jhazmat.2023.132298.

75. Zhang Q, Fan Y, Qian X, et al. Unraveling the role of microplastics in antibiotic resistance: Insights from long-read metagenomics on ARG mobility and host dynamics. *J Hazard Mater*. 2025; doi: 10.1016/j.jhazmat.2025.137804.

76. Charalampous T, Kay GL, Richardson H, et al. Nanopore metagenomics enables rapid clinical diagnosis of bacterial lower respiratory infection. *Nat Biotechnol*. 2019; doi: 10.1038/s41587-019-0156-5.

77. Gu W, Deng X, Lee M, et al. Rapid pathogen detection by metagenomic next-generation sequencing of infected body fluids. *Nat Med*. 2021; doi: 10.1038/s41591-020-1105-z.

78. Alcolea-Medina A, Alder C, Snell LB, et al. Unified metagenomic method for rapid detection of microorganisms in clinical samples. *Commun Med (Lond)*. 2024; doi: 10.1038/s43856-024-00554-3.

79. Lucas MC, Novoa EM. Long-read sequencing in the era of epigenomics and epitranscriptomics. *Nat Methods*. 2023; doi: 10.1038/s41592-022-01724-8.

80. Starcevic A, Figueredo RTA, Naldoni J, et al. Long-read metagenomic sequencing negates inferred loss of cytosine methylation in Myxosporea (Cnidaria: Myxozoa). *Gigascience*. 2025; doi: 10.1093/gigascience/giaf014.

81. Jakubickova M, Sabatova K, Zbudilova M, et al. MethylomeMiner: A novel tool for high-resolution analysis of bacterial methylation patterns from nanopore sequencing. *Comput Struct Biotechnol J*. 2025; doi: 10.1016/j.csbj.2025.10.047.

82. Tourancheau A, Mead EA, Zhang XS, et al. Discovering multiple types of DNA methylation from bacteria and microbiome using nanopore sequencing. *Nat Methods*. 2021; doi: 10.1038/s41592-021-01109-3.

83. Sigurpalsdottir BD, Stefansson OA, Holley G, et al. A comparison of methods for detecting DNA methylation from long-read sequencing of human genomes. *Genome Biol*. 2024; doi: 10.1186/s13059-024-03207-9.

84. Nass NM, Zaher KA. From Methylomes to CRISPR Epigenetic Editing: New Paths in Antibiotic Resistance. *Pathogens*. 2025; doi: 10.3390/pathogens14121267.

85. Yan B, Boitano M, Clark TA, et al. SMRT-Cappable-seq reveals complex

operon variants in bacteria. *Nat Commun*. 2018; doi: 10.1038/s41467-018-05997-6.

86. Fu Y, Timp W, Sedlazeck FJ. Computational analysis of DNA methylation from long-read sequencing. *Nat Rev Genet*. 2025; doi: 10.1038/s41576-025-00822-5.

87. Ding Y, Zhao L, Wang G, et al. PacBio sequencing of human fecal samples uncovers the DNA methylation landscape of 22 673 gut phages. *Nucleic Acids Res*. 2023; doi: 10.1093/nar/gkad977.

88. Wilbanks EG, Dore H, Ashby MH, et al. Metagenomic methylation patterns resolve bacterial genomes of unusual size and structural complexity. *ISME J*. 2022; doi: 10.1038/s41396-022-01242-7.

89. Rodger G, Lipworth S, Barrett L, et al. Comparison of direct cDNA and PCR-cDNA Nanopore sequencing of RNA from Escherichia coli isolates. *Microb Genom*. 2024; doi: 10.1099/mgen.0.001296.

90. Conte MI, Fuentes-Trillo A, Dominguez Conde C. Opportunities and tradeoffs in single-cell transcriptomic technologies. *Trends Genet*. 2024; doi: 10.1016/j.tig.2023.10.003.

91. Wen L, Tang F. Single-cell omics sequencing technologies: the long-read generation. *Trends Genet*. 2026; doi: 10.1016/j.tig.2025.07.012.

92. Lim CS, Gibbon AK, Tran Nguyen AT, et al. RIBOSS detects novel translational events by combining long- and short-read transcriptome and translatome profiling. *Brief Bioinform*. 2025; doi: 10.1093/bib/bbaf164.

93. Tan L, Guo Z, Shao Y, et al. Analysis of bacterial transcriptome and epitranscriptome using nanopore direct RNA sequencing. *Nucleic Acids Res*. 2024; doi: 10.1093/nar/gkae601.

94. Riquelme-Barrios S, Vasquez-Camus L, Cusack SA, et al. Direct RNA sequencing of the Escherichia coli epitranscriptome uncovers alterations under heat stress. *Nucleic Acids Res*. 2025; doi: 10.1093/nar/gkaf175.

95. Simo C, Mamani-Huanca M, Hernandez-Hernandez O, et al. Application of nanopore long-read sequencing and metabolomics in an in vitro dynamic intestinal digestion model: A genome-centric metatranscriptomic approach to investigating microbial TMA and SCFA metabolism. *J Pharm Biomed Anal*. 2025; doi: 10.1016/j.jpba.2025.116896.

96. Barber DG, Davies CA, Hartley IP, et al. Evaluation of commercial RNA extraction kits for long-read metatranscriptomics in soil. *Microb Genom*. 2024; doi: 10.1099/mgen.0.001298.

97. Culviner PH, Guegler CK, Laub MT. A Simple, Cost-Effective, and Robust Method for rRNA Depletion in RNA-Sequencing Studies. *mBio*. 2020; doi: 10.1128/mBio.00010-20.

98. Zhang W, Li XJ, Liu F, et al. Fungen: clustering and correcting long-read metatranscriptomic data for exploring eukaryotic microorganisms. *Sci China Life Sci*. 2026; doi: 10.1007/s11427-024-2853-x.

99. Kolmogorov M, Billingsley KJ, Mastoras M, et al. Scalable Nanopore sequencing of human genomes provides a comprehensive view of

haplotype-resolved variation and methylation. *Nat Methods*. 2023; doi: 10.1038/s41592-023-01993-x.

100. Giordano F, Aigrain L, Quail MA, et al. De novo yeast genome assemblies from MinION, PacBio and MiSeq platforms. *Sci Rep*. 2017; doi: 10.1038/s41598-017-03996-z.

101. Kozarewa I, Ning Z, Quail MA, et al. Amplification-free Illumina sequencing-library preparation facilitates improved mapping and assembly of (G+C)-biased genomes. *Nat Methods*. 2009; doi: 10.1038/nmeth.1311.

102. Wenger AM, Peluso P, Rowell WJ, et al. Accurate circular consensus long-read sequencing improves variant detection and assembly of a human genome. *Nat Biotechnol*. 2019; doi: 10.1038/s41587-019-0217-9.

103. Maghini DG, Moss EL, Vance SE, et al. Improved high-molecular-weight DNA extraction, nanopore sequencing and metagenomic assembly from the human gut microbiome. *Nat Protoc*. 2021; doi: 10.1038/s41596-020-00424-x.

104. Gand M, Bloemen B, Vanneste K, et al. Comparison of 6 DNA extraction methods for isolation of high yield of high molecular weight DNA suitable for shotgun metagenomics Nanopore sequencing to detect bacteria. *BMC Genomics*. 2023; doi: 10.1186/s12864-023-09537-5.

105. Jain M, Koren S, Miga KH, et al. Nanopore sequencing and assembly of a human genome with ultra-long reads. *Nat Biotechnol*. 2018; doi: 10.1038/nbt.4060.

106. Wick RR, Judd LM, Holt KE. Assembling the perfect bacterial genome using Oxford Nanopore and Illumina sequencing. *PLoS Comput Biol*. 2023; doi: 10.1371/journal.pcbi.1010905.

107. Costea PI, Zeller G, Sunagawa S, et al. Towards standards for human fecal sample processing in metagenomic studies. *Nat Biotechnol*. 2017; doi: 10.1038/nbt.3960.

108. De Coster W, Weissensteiner MH, Sedlazeck FJ. Towards population-scale long-read sequencing. *Nat Rev Genet*. 2021; doi: 10.1038/s41576-021-00367-3.

109. Serghiou IR, Baker D, Evans R, et al. An efficient method for high molecular weight bacterial DNA extraction suitable for shotgun metagenomics from skin swabs. *Microb Genom*. 2023; doi: 10.1099/mgen.0.001058.

110. Child HT, Wierzbicki L, Joslin GR, et al. Comparative evaluation of soil DNA extraction kits for long read metagenomic sequencing. *Access Microbiol*. 2024; doi: 10.1099/acmi.0.000868.v3.

111. Slizovskiy IB, Oliva M, Settle JK, et al. Target-enriched long-read sequencing (TELSeq) contextualizes antimicrobial resistance genes in metagenomes. *Microbiome*. 2022; doi: 10.1186/s40168-022-01368-y.

112. Chklovski A, Parks DH, Woodcroft BJ, et al. CheckM2: a rapid, scalable and accurate tool for assessing microbial genome quality using machine learning. *Nat Methods*. 2023; doi: 10.1038/s41592-023-01940-w.

113. Weirather JL, de Cesare M, Wang Y, et al. Comprehensive comparison of Pacific Biosciences and Oxford Nanopore Technologies and their applications

to transcriptome analysis. *F1000Res*. 2017; doi: 10.12688/f1000research.10571.2.
114. Watson M, Warr A. Errors in long-read assemblies can critically affect protein prediction. *Nat Biotechnol*. 2019; doi: 10.1038/s41587-018-0004-z.
115. Pop M, Salzberg SL. Bioinformatics challenges of new sequencing technology. *Trends Genet*. 2008; doi: 10.1016/j.tig.2007.12.006.
116. Koren S, Walenz BP, Berlin K, et al. Canu: scalable and accurate long-read assembly via adaptive k-mer weighting and repeat separation. *Genome Res*. 2017; doi: 10.1101/gr.215087.116.
117. Berlin K, Koren S, Chin CS, et al. Assembling large genomes with single-molecule sequencing and locality-sensitive hashing. *Nat Biotechnol*. 2015; doi: 10.1038/nbt.3238.
118. Rautiainen M, Nurk S, Walenz BP, et al. Telomere-to-telomere assembly of diploid chromosomes with Verkko. *Nat Biotechnol*. 2023; doi: 10.1038/s41587-023-01662-6.
119. Nurk S, Walenz BP, Rhie A, et al. HiCanu: accurate assembly of segmental duplications, satellites, and allelic variants from high-fidelity long reads. *Genome Res*. 2020; doi: 10.1101/gr.263566.120.
120. Kolmogorov M, Bickhart DM, Behsaz B, et al. metaFlye: scalable long-read metagenome assembly using repeat graphs. *Nat Methods*. 2020; doi: 10.1038/s41592-020-00971-x.
121. Feng X, Cheng H, Portik D, et al. Metagenome assembly of high-fidelity long reads with hifiasm-meta. *Nat Methods*. 2022; doi: 10.1038/s41592-022-01478-3.
122. Benoit G, Raguideau S, James R, et al. High-quality metagenome assembly from long accurate reads with metaMDBG. *Nat Biotechnol*. 2024; doi: 10.1038/s41587-023-01983-6.
123. Trigodet F, Sachdeva R, Banfield JF, et al. Troubleshooting common errors in assemblies of long-read metagenomes. *Nat Biotechnol*. 2026; doi: 10.1038/s41587-025-02971-8.
124. Zhao W, Zeng W, Pang B, et al. Oxford nanopore long-read sequencing enables the generation of complete bacterial and plasmid genomes without short-read sequencing. *Front Microbiol*. 2023; doi: 10.3389/fmicb.2023.1179966.
125. Sanderson ND, Hopkins KMV, Colpus M, et al. Evaluation of the accuracy of bacterial genome reconstruction with Oxford Nanopore R10.4.1 long-read-only sequencing. *Microb Genom*. 2024; doi: 10.1099/mgen.0.001246.
126. Sereika M, Kirkegaard RH, Karst SM, et al. Oxford Nanopore R10.4 long-read sequencing enables the generation of near-finished bacterial genomes from pure cultures and metagenomes without short-read or reference polishing. *Nat Methods*. 2022; doi: 10.1038/s41592-022-01539-7.
127. Wick RR, Judd LM, Cerdeira LT, et al. Trycycler: consensus long-read assemblies for bacterial genomes. *Genome Biol*. 2021; doi:

10.1186/s13059-021-02483-z.

128. Brown CL, Keenum IM, Dai D, et al. Critical evaluation of short, long, and hybrid assembly for contextual analysis of antibiotic resistance genes in complex environmental metagenomes. *Sci Rep*. 2021; doi: 10.1038/s41598-021-83081-8.

129. Bertrand D, Shaw J, Kalathiyappan M, et al. Hybrid metagenomic assembly enables high-resolution analysis of resistance determinants and mobile elements in human microbiomes. *Nat Biotechnol*. 2019; doi: 10.1038/s41587-019-0191-2.

130. Jin H, You L, Zhao F, et al. Hybrid, ultra-deep metagenomic sequencing enables genomic and functional characterization of low-abundance species in the human gut microbiome. *Gut Microbes*. 2022; doi: 10.1080/19490976.2021.2021790.

131. Jain C, Rodriguez RL, Phillippy AM, et al. High throughput ANI analysis of 90K prokaryotic genomes reveals clear species boundaries. *Nat Commun*. 2018; doi: 10.1038/s41467-018-07641-9.

132. Koren S, Bao Z, Guarracino A, et al. Gapless assembly of complete human and plant chromosomes using only nanopore sequencing. *Genome Res*. 2024; doi: 10.1101/gr.279334.124.

133. Benoit G, James R, Raguideau S, et al. High-quality metagenome assembly from nanopore reads with nanoMDBG. *Nat Commun*. 2026; doi: 10.1038/s41467-026-69760-y.

134. Xue W, Liu Z, Zhang Y, et al. LorBin: efficient binning of long-read metagenomes by multiscale adaptive clustering and evaluation. *Nat Commun*. 2025; doi: 10.1038/s41467-025-64916-8.

135. Han H, Wang Z, Zhu S. Benchmarking metagenomic binning tools on real datasets across sequencing platforms and binning modes. *Nat Commun*. 2025; doi: 10.1038/s41467-025-57957-6.

136. Pages-Gallego M, de Ridder J. Comprehensive benchmark and architectural analysis of deep learning models for nanopore sequencing basecalling. *Genome Biol*. 2023; doi: 10.1186/s13059-023-02903-2.

137. Singh G, Alser M, Denolf K, et al. RUBICON: a framework for designing efficient deep learning-based genomic basecallers. *Genome Biol*. 2024; doi: 10.1186/s13059-024-03181-2.

138. Urel H, Benassou S, Marti H, et al. Nanopore- and AI-empowered microbial viability inference. *Gigascience*. 2025; doi: 10.1093/gigascience/giaf100.

139. Child HT, Wierzbicki L, Joslin GR, et al. Automated environmental metagenomics using Oxford nanopore sequencing. *BMC Genomics*. 2025; doi: 10.1186/s12864-025-11989-w.

140. Motone K, Kontogiorgos-Heintz D, Wee J, et al. Multi-pass, single-molecule nanopore reading of long protein strands. *Nature*. 2024; doi: 10.1038/s41586-024-07935-7.

141. Wang J, Chen J, Pan H, et al. Nanopore-based massively parallel sensing for peptide profiling and protein identification. *Nat Commun*. 2026; doi:

10.1038/s41467-026-69628-1.
142. Liu X, Dong Z, Timp G. Calling the amino acid sequence of a protein/peptide from the nanospectrum produced by a sub-nanometer diameter pore. *Sci Rep*. 2022; doi: 10.1038/s41598-022-22305-x.
143. Liu T, Conesa A. Profiling the epigenome using long-read sequencing. *Nat Genet*. 2025; doi: 10.1038/s41588-024-02038-5.
144. Monzo C, Liu T, Conesa A. Transcriptomics in the era of long-read sequencing. *Nat Rev Genet*. 2025; doi: 10.1038/s41576-025-00828-z.
145. Shaffer JP, Nothias LF, Thompson LR, et al. Standardized multi-omics of Earth's microbiomes reveals microbial and metabolite diversity. *Nat Microbiol*. 2022; doi: 10.1038/s41564-022-01266-x.
146. Go D, Yeon GH, Park SJ, et al. Integration of metabolomics and other omics: from microbes to microbiome. *Appl Microbiol Biotechnol*. 2024; doi: 10.1007/s00253-024-13384-z.
147. Powell JE. Charting immune variation through genetics and single-cell genomics. *Gigascience*. 2026; doi: 10.1093/gigascience/giaf161.
148. Google (2026). Gemini (3.1 Pro) [Large language model]. Response to queries made by Xing Rao, Yuzheng Gu, Gabriella, and Jiayi Ma. 04/2026. https://gemini.google.com

Table 1. Studies demonstrating the implementation of LRS for isolate microbial genome reconstruction (2013–2026)

**Year**	**Study**	**Platform strategy**	**Isolate(s) / dataset**	**Main contribution to genome quality**
2013	Koren et al.	PacBio RS	6 bacterial genomes + 2,267 reference genomes	LRS reduced assembly complexity, showing over 70% of known bacterial and archaeal genomes could be finished using a single PacBio library
2013	Chin et al.	PacBio RS SMRT	*Escherichia coli* K-12 MG1655, *Meiothermus ruber* DSM1279, *Pedobacter heparinus*	Demonstrated non-hybrid finishing from a single long-insert library improved resolution of long repeats, and consensus accuracy exceeding 99.999%

			DSM2366	
2015	Loman et al.	ONT MinION	*Escherichia coli* K-12 MG1655	Produced a single 4.6 Mb contig with 99.5% nucleotide identity, providing early proof that nanopore-only data could recover complete chromosome structure
2017	Wick et al.	ONT MinION + Illumina HiSeq	12 *Klebsiella pneumoniae* isolates	Multiplex MinION sequencing with hybrid assembly could enable a scalable, automated bacterial genome closure with fully resolved assemblies
2017	Teng et al.	PacBio RS II + Illumina HiSeq	*Burkholderia pseudomallei*	PacBio resolved the high-GC, two-chromosome genome with >99.9% identity, while Illumina assemblies remained fragmented
2017	George et al.	ONT MinION + Illumina	8 *Enterobacteriaceae* isolates	Chromosomal contigs matched >95% of the reference chromosomal sequence length. Plasmid reconstruction recovered 70% and increased to 78% with combined assembler strategy
2019	De Maio et al.	Illumina + ONT MinION or PacBio RS	20 *Enterobacteriaceae* isolates	LRS improves the reads on repeats, plasmid, and complex regions. In hybrid workflows with Illumina data, it enables more complete, accurate, and automation-friendly bacterial genome assemblies
2019	Sydenham et	Illumina MiSeq	6 *Bacteroides fragilis*	Generated complete circular assemblies of all

	al.	+ ONT MinION	isolates	isolates, identified 11 circular plasmids, and resolved complete insertion sequences upstream of AMR genes.
2022	Sereika et al.	ONT R10.4	Pure cultures	Assembled near-finished microbial genomes without polishing, reaching 99% accuracy
2024	Sanderson et al.	ONT R10.4.1	13 bacterial isolates	Updated chemistry and ≥40× coverage enabled accurate bacterial reconstruction using just LRS reads
2024	Luan et al.	ONT R9.4.1 + Illumina MiSeq	15 *Salmonella enterica serovar*	LRS reached 99.95% accuracy for complete genomes, but "near-perfect" 99.9999% required hybrid approach
2025	Fan et al.	DNBSEQ-T5 + CycloneSEQ-WT02	*Lactiplantibacillus plantarum* BGI-J9	A hybrid of SRS and LRS strategy achieved a complete genome assembly with 100% completeness.
2025	Abdel-Glil et al	ONT R10.4.1 + Illumina MiSeq	*Y. Similis* DSM 18211, *K. pneumonia* DSM 30104, *E. coli* ATCC 25922, *E. coli* ATCC 35218, and *A. baumannii* DSM 30007	Demonstrated that nanopore-only assemblies are highly reproducible, though short-read polishing further refines consensus accuracy.
2025	Wang et al.	DNBSEQ-T5 +	*Lacticplantibacillus*	Achieved a complete genome assembly, replacing

		CycloneSEQ-WT02	*plantarum* BGI-N6	fragmented draft structure with a fully resolved genome.
2025	Xu et al.	ONT R10.4.1	*E. coli* isolates	Provided a high-quality reconstruction of genomes, plasmids, and ARG contexts, yielding 1016 genomes and 2647 circular plasmids
2025	Wang et al.	DNBSEQ-T5 + CycloneSEQ-WT02	*Lacticaseibacillus rhamnosus* OF44	Achieved a complete genome assembly of a single-contig with 99.99% completeness and 0.19% contamination.
2025	Adams et al	PacBio HiFi + Illumina NextSeq	*Staphylococcus aureus* 63-2498	Produced a closed single‑contig chromosome assembly (98.62% complete) along with three plasmids
2026	Nagy et al	ONT R10.4.1 + Illumina NovaSeq	92 *Enterobacterales* isolates	Demonstrated similar results from LRS only assemblies to hybrid assemblies when using Autocycler+Medaka assembler.
2026	Rao et al.	DNBSEQ-T5 + CycloneSEQ-WT02 (Hybrid)	*Lacticaseibacillus paracasei* BGI-N2	Achieved a complete single-contig genome assembly of 3,102,337 bp, with 46.4% GC content and 2,937 CDs.

Table 2. Representative LRS studies for MAG and cMAG recovery from metagenomes.

Year	Study	Platform and strategy	Metagenomic dataset	MAG/cMAG recovery
2019	Bertrand et al.	Illumina + ONT nanopore; OPERA-MS hybrid assembly	Mock communities and 28 human gut metagenomes	Improved contiguity ~200-fold; recovered rare-species genomes and >80 closed plasmid/phage sequences.
2020	Moss et al.	ONT MinION R9.4; Lathe workflow with short-read polishing	13 human stool metagenomes	Recovered 20 circular bacterial genomes directly from microbiomes.
2021	Singleton et al.	ONT PromethION + Illumina; long-read assembly with short-read support	Activated sludge from 23 Danish WWTPs	Recovered 1,083 HQ MAGs, including 57 closed circular genomes.
2022	Bickhart et al.	PacBio HiFi + Hi-C binning; MAGPhase strain-level resolution	Sheep faecal metagenome	Recovered 428 MAGs, including 44 single-contig circular MAGs and 220 lineage-resolved MAGs.
2022	Feng et al.	PacBio HiFi; hifiasm-meta assembler	Seven empirical metagenomic datasets	Reconstructed tens to hundreds of complete circular bacterial genomes per dataset.
2022	Kim et al.	PacBio HiFi, including Sequel II data; metaFlye/HiCanu/hifiasm-meta plus cMAGfilter	Five human faecal metagenomes	Recovered 102 accurate cMAGs, including uncultured gut taxa.
2022	Gounot et al.	Illumina HiSeq4K + ONT MinION/GridION/PromethION; OPERA-MS; Hi-C subset	109 Southeast Asian gut metagenomes	Recovered 4,497 medium/HQ MAGs; 1,708 were absent from short-read-only analysis.
2024	Benoit et al.	PacBio HiFi; metaMDBG assembler	Human gut, anaerobic digester and sheep	Increased HQ circular MAG recovery, with improved plasmid

			rumen datasets	and virus assembly.
2025	Sereika et al.	ONT P2/P24, SQK-LSK114/FLO-PRO114M; Flye/Medaka + mmlong2 workflow	154 soil and sediment samples	Recovered 23,843 MAGs, dereplicated into 15,640 species-level MAGs.
2026	Benoit et al.	ONT R10.4.1; nanoMDBG assembler	Human gut, Zymo Fecal Reference and soil metagenomes	Recovered more near-complete MAGs than ONT comparators; showed ONT can approach HiFi MAG recovery at matched depth.
2026	Shaw et al.	PacBio HiFi and ONT R10.4; myloasm assembler	Gut, oral and other metagenomic datasets	Improved strain-resolved assembly and recovery of complete circular contigs.

Figure

Click here to access/download;Figure;Figure 1.pdf

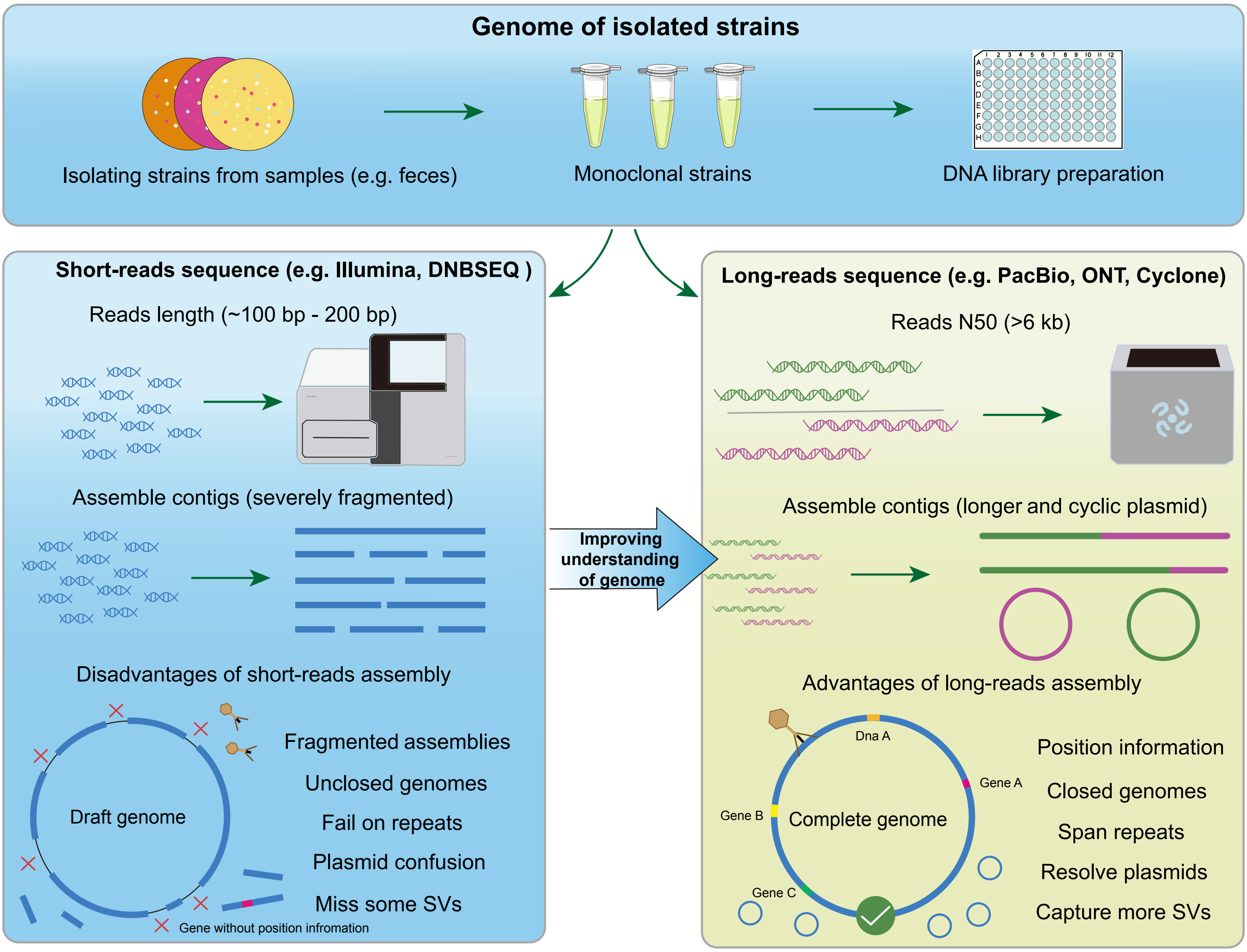

Figure

Click here to access/download;Figure;Figure 2.pdf

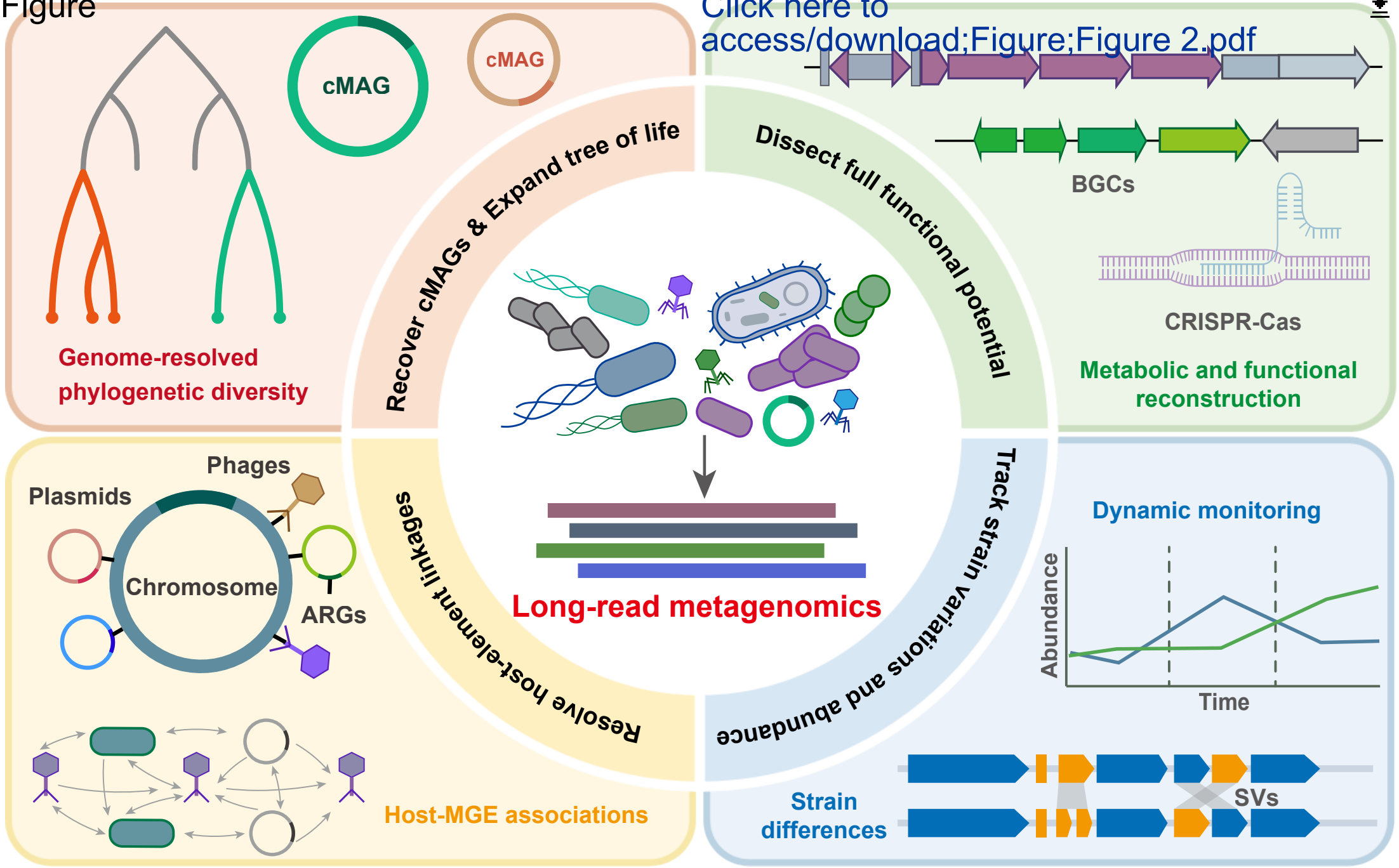

Figure

Click here to access/download;Figure;Figure 3.pdf

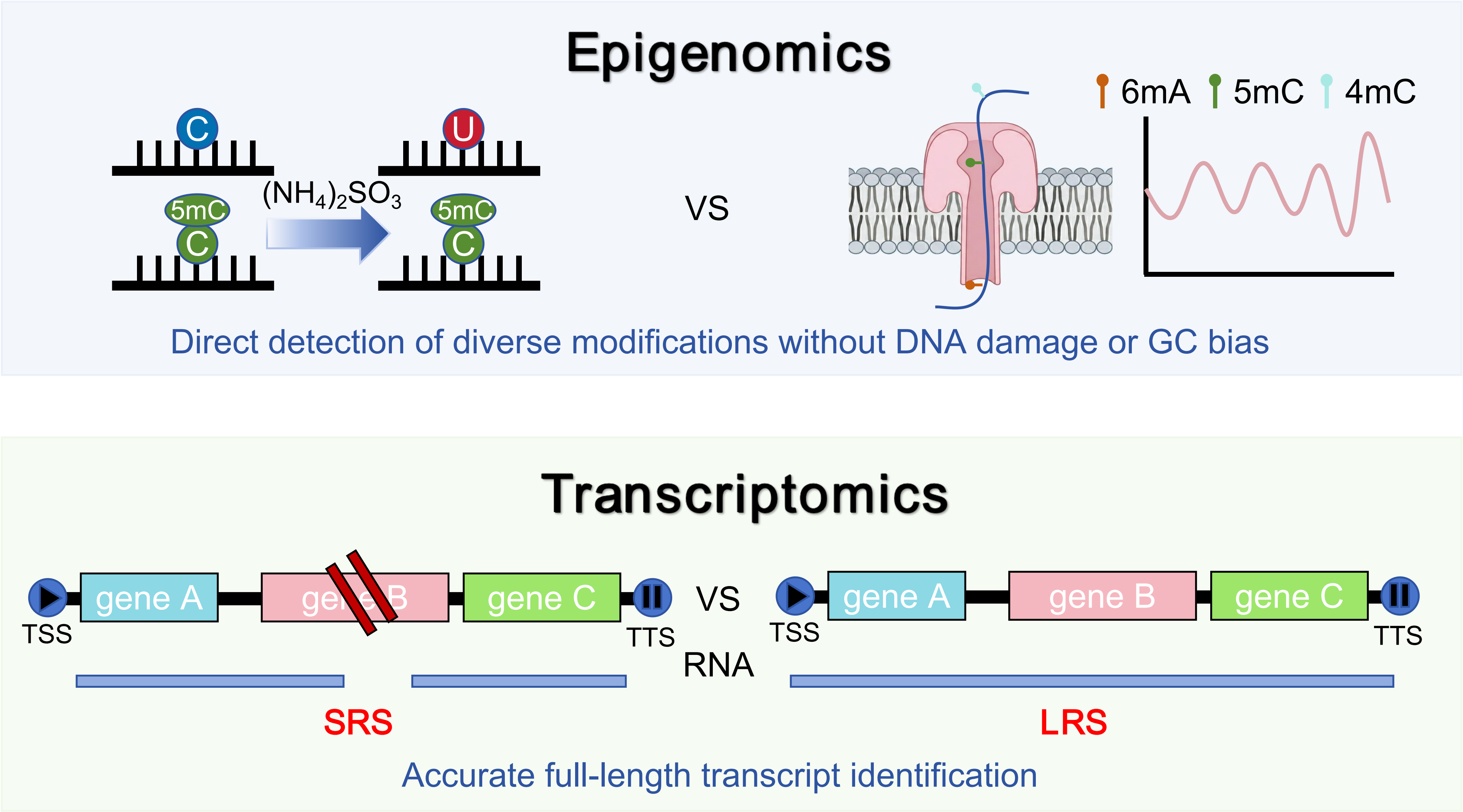

Figure

Click here to access/download;Figure;Figure 4.pdf

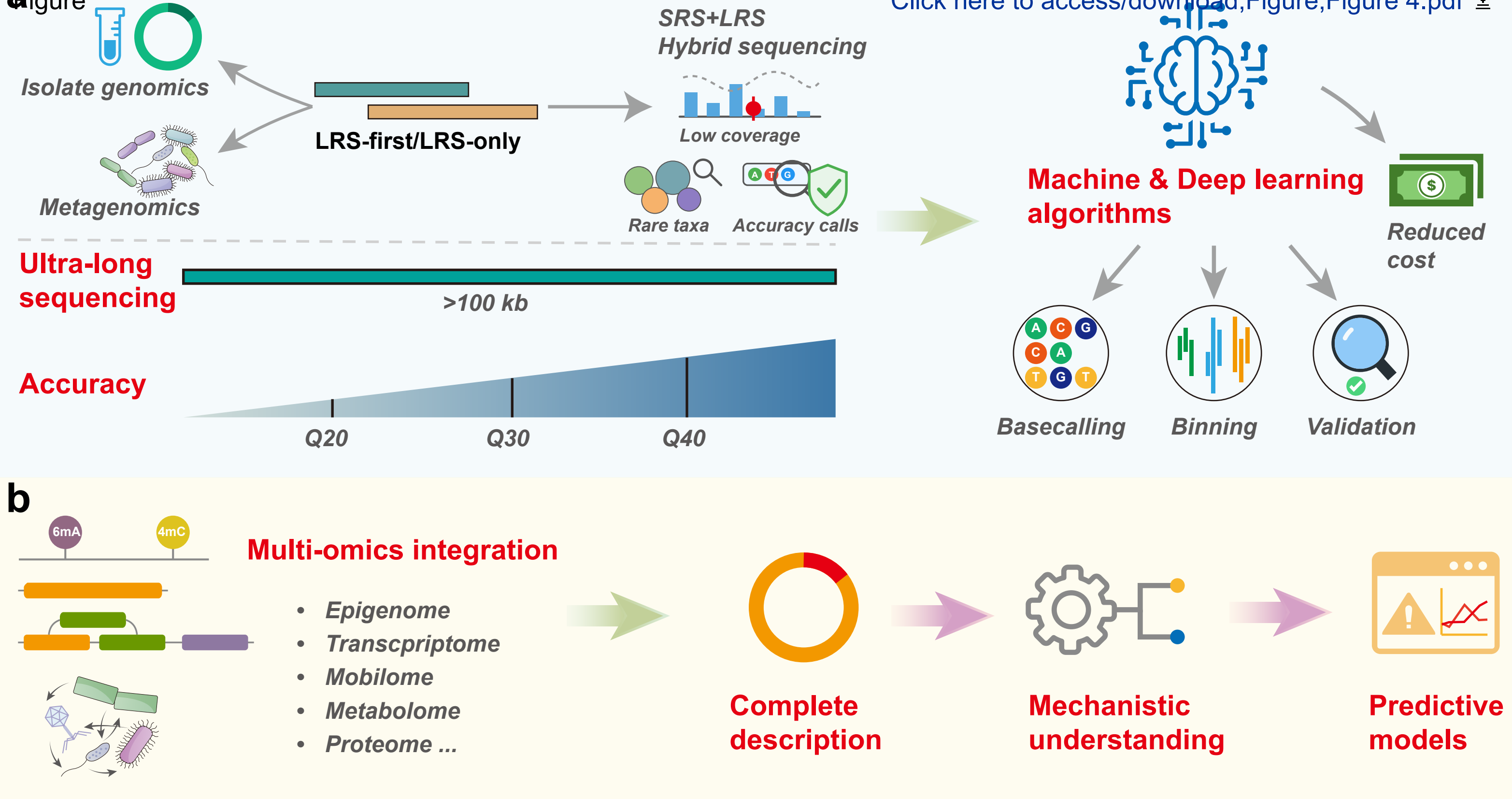

Dear editor:

We thank the editor and reviewers for the constructive re-review comments. We are pleased that the manuscript has been provisionally accepted pending minor revisions. We have carefully addressed all remaining points and included the three relevant GigaScience articles.

Responses to reviewers:

Reviewer #2 (original reviewer)

Summary: Rao et al. have sufficiently addressed my previous comments. I would however suggest to address the following minor comments prior to publication.

Minor comments:

L250: I would suggest to briefly explain "Hi-C".

Response:

We thank the reviewer for this suggestion. We have added a brief explanation of Hi-C at its first occurrence: "..... Hi-C, a proximity ligation method used to link DNA fragments from the same cell, ..." (Lines 251).

L257: "bin" -> "MAG".

Response:

We have replaced "bins" with "MAGs" in this sentence. (Line 258)

L274: "HiFi" -> "PacBio HiFi".

Response:

We have replaced "HiFi" with "PacBio HiFi" in this sentence. (Line 275)

L431: Remove the "the" from "the high sensitivity".

Response:

We have removed the "the" from "the high sensitivity". (Line 432)

Reviewer #3 Reviewers' comments: Prior concerns that remain insufficiently addressed

1. Major comment 4 (AI-use disclosure / journal compliance). This is the main leftover issue. Reviewer #2 asked for a proper summary of use and for the outputs to be provided as required by the journal. The revised disclosure is still only two sentences: Gemini 3.1 Pro was used for language checking and wording suggestions, and no AI-generated content was incorporated without author review. That still does not say how the tool was used, which sections were edited, or what the outputs were. There is still no supplementary file. Reference 145 is also incomplete ("Google (2026). x (3.1 Pro)"), so the required model/version citation is not in usable form. If the authors maintain that only language polishing occurred and that no output needs to be deposited, they should state this explicitly, list the users, model, version and date in

the disclosure itself, and correct reference 145. If any AI text or figure output was generated, that material still needs to be deposited as previously requested.

Response:

We thank the reviewer for this comment. We have revised the AI-use disclosure to specify the tool, model, date of use, and purpose of use. "The revised disclosure states that Gemini 3.1 Pro was used only as an auxiliary tool for grammar checking and wording suggestions during manuscript preparation in April 2026, and was not used to generate original scientific content, figures, tables, references, or conclusions. All AI-assisted suggestions were reviewed and revised by the authors." (Lines 748-752)

We also corrected Reference 145 (now Reference 148) as follows: "Google (2026). Gemini (3.1 Pro) [Large language model]. Response to queries made by Xing Rao, Yuzheng Gu, Gabriella, and Jiayi Ma. 04/2026. https://gemini.google.com "

2. Major comment 2, remaining consistency items. "Parsing" has been removed from the main text, but Figure 2 still says "High-resolution haplotype phasing" without defining the term. That is the same accessibility/terminology problem Reviewer #2 raised. Please either define haplotype phasing in the figure legend or use the same "strain-level resolution" wording already adopted in the text. Also, Reviewer #2 asked for a thorough reference check after the invalid citation. The invalid citation is gone, but the broken "x (3.1 Pro)" entry shows the reference list still needs a final pass. I could not inspect the figure graphics themselves in the Word file provided, so I cannot confirm that the capitalization issues in Figures 1 and 4 were fully corrected; please make sure this was done in the submitted figure files.

Response:

We thank the reviewer for pointing out this. To avoid introducing another undefined technical term in Figure 2, we replaced "High-resolution haplotype phasing" with the wording used in the main text, "Strain-level resolution." (Line 231) In addition, we performed another final check of the reference list and corrected Reference 145 to provide the complete model and version information for Gemini 3.1 Pro. We also rechecked the submitted figure files and standardized capitalization in Figures 1 and 4.

Additional editorial request:

In order to better contextualize your work within the broader scope of methods and applications published in GigaScience, we kindly ask that you refer to the following three relevant articles from the journal in your revised manuscript (e.g., in the Introduction, Section 4/5, or Discussion, as appropriate): 1. Starcevic A, Figueredo RTA, Naldoni J, et al. Long-read metagenomic sequencing negates inferred loss of cytosine methylation in Myxosporea (Cnidaria: Myxozoa). GigaScience. 2025;14:giaf014. doi: 10.1093/gigascience/giaf014. 2. Ürel H, Benassou S, Marti H, et al. Nanopore- and AI-empowered microbial viability inference. GigaScience. 2025;14:giaf100. doi: 10.1093/gigascience/giaf100. 3. Powell R, et al. Charting

immune variation through genetics and single-cell genomics. GigaScience. 2026;15:giaf161. doi: 10.1093/gigascience/giaf161.

Response:

We thank the editor for this suggestion. We have added and discussed the three relevant GigaScience articles in the revised manuscript. Specifically, Starcevic et al. was cited in the section on microbial epigenomics (Reference 80), Ürel et al. was cited in the future analytical development (Reference 138), and Powell et al. was cited in the discussion of broader multi-omics integration (Reference 147).
