# Supplementary material for: Promises and pitfalls of long-read sequencing for resolving microbial complexity
Source: Gigascience. 2026 Aug 17;15:giag087. doi: 10.1093/gigascience/giag087 (PMC13543314; doi:10.1093/gigascience/giag087)
Supplement: Supplementary file 6 [file giag087_reviewer_1_report_original_submission.pdf]

Reviewer Report

Title: Promises and Pitfalls of Long-Read Sequencing for Resolving Microbial Complexity

Version: Original Submission **Date:** 5/17/2026

Reviewer name: Reviewer 1

Reviewer Comments to Author:

This review provides a comprehensive overview of the paradigm shift that long-read sequencing (LRS) has brought to microbial genomics and appropriately discusses its current technical limitations. A particular strength of the manuscript is the unusually rich set of case studies focused on pathogenic bacteria and antibiotic resistance genes, which the authors discuss from several complementary angles. This emphasis is, in my view, the most distinctive contribution of the review. Based on this strength, I would like to suggest the following improvements to further enhance the manuscript:
* Addition of a schematic figure that specifically illustrates how LRS has benefited pathogenic bacterial genomics would substantially help readers situate the technological gains in a translational context.
* The long-read metagenomics sections would benefit from additional content connecting LRS to pathogenic bacterial genomics. For example, the current status of real-time pathogen detection enabled by long-read metagenomics is a particularly impactful topic and would be a natural extension.
* If the authors retain the current emphasis on pathogenic bacteria throughout the manuscript, a corresponding revision of the title to more accurately reflect this scope would be warranted.
Major comments
1. Section 2.3 â€” Claim that long reads outperform short reads in SNP detection (Lines 205-208, p.10)
The current text states that ONT long reads outperform short reads for SNP calling. Given that, on a per-base basis, short reads still have a substantially lower error rate than long reads, this conclusion may strike many readers as counter-intuitive. In all likelihood, the long-read advantage is restricted to specific genomic contexts (for example, repetitive regions and loci embedded in structural variants or mobile genetic elements) where short reads cannot be uniquely mapped. I recommend that the authors explicitly clarify the underlying reason why long reads outperform short reads at SNP calling in these specific contexts. This framing will help prevent readers from overestimating the general per-base SNP accuracy of LRS platforms.
2. Figure 2 / Section 3 â€” Why does cMAG construction expand phylogenetic diversity? (Lines 288-289, p.14)
The current sentence â€” "LRS facilitates the direct reconstruction of cMAGs, thereby significantly expanding the known phylogenetic diversity" â€” is asserted without an explicit rationale. If the sole goal were to expand phylogenetic diversity, conventional 16S rRNA gene surveys would, in principle, already suffice. I recommend that the authors clarify (and cite supporting primary literature, both in the main text and in the Figure 2 caption) the specific reasons why cMAG construction is required to expand phylogenetic diversity beyond what marker-gene approaches can achieve.
3. Chromosome-MGE linkage in metagenomic contexts (Lines 292-294, p.14)
I agree with the statement that long reads enable the separation of chromosomal contigs and MGE

contigs in single-isolate assemblies. However, in a true metagenomic context, identifying the chromosomal host of an MGE remains difficult even with long reads alone. The current wording risks overstating the present capability. I suggest the authors add a sentence describing which complementary strategies can (at least partially) establish chromosome-MGE linkages in metagenomes with appropriate citation, such as proximity-ligation methods (Hi-C / 3C), methylation-based host assignment, and ultra-long reads that physically span the integration site between the MGE and its chromosomal flanking sequence.

4. Section 3.2 â€” Mechanism of strain-resolution long-read metagenomics, and recent tooling (p.16)
The emphasis on strain resolution as a key strength of long-read metagenomics is well taken and highly valuable for the field. To strengthen this section further, please add a concise explanation of how strain resolution is actually achieved with long reads (e.g., preservation of haplotypic linkage on a single molecule, phasing of SNVs/SVs across long stretches, and traversal of structurally variable accessory loci). In addition, the recent release of myloasm (Shaw J, Marin MG, Li H. High-resolution metagenome assembly for modern long reads with myloasm. Nat Biotechnol. 2026; https://doi.org/10.1038/s41587-026-03053-z) makes strain-resolution long-read metagenomic assembly substantially more accessible in practice, and I encourage the authors to mention this development in this section.

5. Section 4.1 â€” HMW DNA, low-abundance modifications and sequencing depth (Lines 488-491, p.23)
Two related points require clarification here. First, please explain explicitly why HMW DNA extraction improves the accuracy of methylation calling. Second, the statement that ONT "lacks sufficient accuracy in detecting very low-abundance modifications" is essentially equivalent to saying that sequencing depth is insufficient â€” and this is not a limitation specific to methylation analysis. At current depths, low-abundance chromosomes and MGEs frequently fail to assemble at all. I therefore recommend either reframing this limitation more precisely or, ideally, if possible, providing a practical description for the sequencing depth required to recover reliable methylation profiles from representative samples such as the human gut microbiome.

6. Table 1 / Section 6 â€” Over-representation of CycloneSEQ studies and disclosure of competing interests
In Table 1, every entry from 2025-2026 uses a CycloneSEQ-WT02 + DNBSEQ-T5 hybrid strategy and is contributed by BGI-affiliated groups. Given that several of the authors of this review are affiliated with BGI Research, the present selection risks the appearance of a non-representative emphasis on a single platform that is commercially associated with the authors' institution. I recommend two adjustments: (i) explicitly add a Competing Interests statement disclosing the authors' affiliation with BGI and the relationship to the CycloneSEQ platform; and (ii) balance the recent rows of Table 1 (and the corresponding narrative in Section 6) with comparable 2024-2026 studies using ONT or PacBio platforms. This will substantially strengthen the reader's confidence that platform recommendations in this review reflect the broader field rather than the authors' in-house technology.

7. Section 6 / Abstract / Conclusion â€” Tone of the "hybrid remains the most practical strategy" recommendation should be revised
The manuscript's top-line recommendation â€” that "hybrid sequencing strategies remain the contemporary method" (Abstract line 47; Conclusion line 753) â€” no longer reflects the current state of the field. As the authors themselves note in Section 6, complete or near-complete genomes can often be recovered from isolates and microbiomes without mandatory short-read correction (Lines 667-669), and

the field is moving toward Q30-class long reads as a realistic standard (Lines 671-672). In practice, for the most common use case â€” isolated genome sequencing â€" LRS-only workflows are already sufficient, and hybrid sequencing is only required in specific situations such as low-coverage regions, capture of rare community members in metagenomes, and applications where the highest possible consensus accuracy or conservative variant interpretation is critical (as the authors describe in Lines 657-662). I recommend that the authors revise the overall tone of Section 6, the Abstract, and the Conclusion to reflect this reality: namely, that LRS-only should now be presented as the default for isolate genomics and increasingly for metagenomics, with hybrid sequencing positioned as a targeted strategy for the specific conditions in which it remains clearly justified. Without this rebalancing, the current top-line statement risks understating the rapid accuracy gains of the past two years and inadvertently steering readers away from workflows that the same Section 6 acknowledges as already viable.

8. Sections 4.2 and 4.3 â€" Unclear metagenomic relevance of full-length transcriptomics and single-molecule proteomics

Sections 4.2 (full-length transcriptomics) and 4.3 (single-molecule proteomics) are written essentially as general technology overviews of long-read transcriptome / proteome sequencing, without demonstrating how either technique is actually applied â€" or could plausibly be applied â€" in a microbial metagenomic context. Given that the central theme of this review is the resolution of microbial complexity in complex communities, it is unclear what additional value these two sections contribute in their current form. Specifically: (i) Section 4.2 discusses bacterial isolate transcriptomics in detail, but does not address the metatranscriptomic challenges that would actually be relevant to community-scale analysis (e.g., full-length RNA recovery from complex communities, rRNA depletion, assignment of transcripts back to specific lineages or strains); (ii) Section 4.3 contains essentially no microbial application examples, as refs 96-100 are technology-development papers, and the connection to "microbial complexity" is not made explicit. I therefore recommend either substantially expanding these sections to include concrete metagenomic / community-level use cases (even at the pilot or proof-of-concept stage), or, if such examples are scarce at present, relocating this material to an Outlook box or a subsection of Section 6 (Future perspectives) and tightening the body of the review to its actual scope.

Minor comments

1. p.4, Lines 82-83 â€" Additional citation suggested

The discussion of prophages confounding short-read assembly would benefit from an additional citation: Wirbel J, Hickey AS, Chang D, Enright NJ, Dvorak M, Chanin RB, Schmidtke DT, Bhatt AS. Long-read metagenomics reveals phage dynamics in the human gut microbiome. Nature. 2025; 1-9.

2. Insufficient in-text citation of figures

Although the manuscript contains four figures, the figures are not adequately cited at the relevant points in the main text. Please add explicit in-text citations at every point where the corresponding figure panel supports the narrative, so that readers can map the discussion to the visual summary.

Methods

Are the methods appropriate to the aims of the study, are they well described, and are necessary controls included? Choose an item.

Conclusions

Are the conclusions adequately supported by the data shown? Choose an item.

Reporting Standards

Does the manuscript adhere to the journal's guidelines on minimum standards of reporting? Choose an item.

Choose an item.

Statistics

Are you able to assess all statistics in the manuscript, including the appropriateness of statistical tests used? Choose an item.

Quality of Written English

Please indicate the quality of language in the manuscript: Choose an item.

Declaration of Competing Interests

Please complete a declaration of competing interests, considering the following questions:

- Have you in the past five years received reimbursements, fees, funding, or salary from an organisation that may in any way gain or lose financially from the publication of this manuscript, either now or in the future?
- Do you hold any stocks or shares in an organisation that may in any way gain or lose financially from the publication of this manuscript, either now or in the future?
- Do you hold or are you currently applying for any patents relating to the content of the manuscript?
- Have you received reimbursements, fees, funding, or salary from an organization that holds or has applied for patents relating to the content of the manuscript?
- Do you have any other financial competing interests?
- Do you have any non-financial competing interests in relation to this paper?

If you can answer no to all of the above, write 'I declare that I have no competing interests' below. If your reply is yes to any, please give details below.

I declare that I have no competing interests.

I agree to the open peer review policy of the journal. I understand that my name will be included on my report to the authors and, if the manuscript is accepted for publication, my named report including any attachments I upload will be posted on the website along with the authors' responses. I agree for my report to be made available under an Open Access Creative Commons CC-BY license (http://creativecommons.org/licenses/by/4.0/). I understand that any comments which I do not wish to be included in my named report can be included as confidential comments to the editors, which will not be published.

I agree to the open peer review policy of the journal

To further support our reviewers, we have joined with Publons, where you can gain additional credit to further highlight your hard work (see: https://publons.com/journal/530/gigascience). On publication of this paper, your review will be automatically added to Publons, you can then choose whether or not to claim your Publons credit. I understand this statement.

Yes Choose an item.
