# Supplementary material for: Promises and pitfalls of long-read sequencing for resolving microbial complexity
Source: Gigascience. 2026 Aug 17;15:giag087. doi: 10.1093/gigascience/giag087 (PMC13543314; doi:10.1093/gigascience/giag087)
Supplement: Supplementary file 8 [file giag087_reviewer_2_report_original_submission.pdf]

Reviewer Report

Title: Promises and Pitfalls of Long-Read Sequencing for Resolving Microbial Complexity

Version: Original Submission **Date:** 5/19/2026

Reviewer name: Reviewer 2

Reviewer Comments to Author:

In this work, Rao and Gu et al. review the application of long read-sequencing to reconstruct microbial genomes from microbial isolate culture and metagenome-assembled genomes from microbiome samples. They provide an overview of the main long read-sequencing approaches and the analytical challenges they improve on, notably the resolution of extended repeats, genome rearrangements, and the detection of epigenetic modifications. While the authors cover the recent literature, I have the following comments:

Major comments:

1. The authors cite the work by Agustinho et al. (2024) Nature Methods. I found the overlap between the 2024 review and the present review quite large. Please specify what novel information the present review provides compared to previous reviews on long read-sequencing in the context of isolate genomes and/or metagenomes. It might be advisable to adjust the text accordingly for the reader to better appreciate what sets this review apart from other, very recent works.
2. I found the text to read repetitive frequently, such as about fragmented assemblies using short reads and the possibility to sequence free of prior amplification with long reads. The authors refer to improved assembly using long reads but only introduce in section 5.3 how assembly actually takes place; a different order would help reduce redundancy.
3. The authors declare no competing interests. However, Rao and Zou are both affiliated to BGI Research, with BGI producing the CycloneSeq platform. I think that in the interest of transparency, this should be disclosed by the authors.
4. The authors repeatedly refer to "physical linkage". Please carefully review this and appropriate use of respective phrasing in the text. While long read-sequencing can produce circular chromosomes and circular plasmids, physical linkage of these genetic elements is only established through complementary approaches, e.g., proximity ligation approaches. Put differently, circular chromosomes and circular plasmids are not automatically put into their shared organismal context through long read-sequencing.
5. In the interest of consistency, please include a table like Table 1 but for metagenome-assembled genomes. Importantly, the use of long read-sequencing for isolate genomes is largely an accepted standard by now, while the use for the recovery of metagenome-assembled genomes remains a challenge.

Minor comments:

1. Please use nuanced language. Long read-sequencing has not "shattered", and definitely not "thoroughly", anything just yet, albeit it is a promising technology. Moreover, it remains to be seen whether there has indeed been an "era" of epitranscriptomics inaugurated. While I agree that the ease of accessing epigenetic modifications has been simplified with some long read-sequencing approaches

(by ONT and PacBio), this has yet to be applied on a larger scale. Related to this, please check for the repeated use of "bottleneck". And why is it surprising that long read-sequencing "enable the recovery of thousands of complete ribosomal RNA operons …", given that this is exactly where long read-sequencing can show its strengths?
2. The authors refer to "complex" when referring to microbiomes. I assume this is to differentiate microbiomes from isolate cultures, but it is extraneous if it is clear whether isolate genomes or metagenome-assembled genomes are targeted. More generally, what constitutes a "complex" microbiome and what constitutes a "simple" microbiome? I would advise against the use of such terms whenever possible.
3. Please pay attention to the introduction of abbreviations before their use, e.g., "TSS", "TTS", "SNVs", or "SV". Notably after possibly adjusting the text order as pointed out in the major comments. Moreover, if an abbreviation is only introduced but never reused, please consider not writing it in the first place, e.g., "WGS" which is introduced in line 247 but not used thereafter.
4. What do the authors mean by "haplotypic parsing"? Did they mean "haplotype phasing"? If so, please adjust throughout.
5. Please pay attention to the correct spelling throughout, notably including also the figures. "CRISPR" is misspelled in Figure 2.
6. What is the purpose of reference 144? I understand that the authors want to disclose the use of Gemini for language redaction, but the included link is a generic link to Gemini and not to the prompts that were used. On that note, from the reading it was readily apparent that a large language model was used as the language was often times exaggerated, see also my earlier comment on the advice to use more nuanced language.
7. There appears to be an issue with Table 1. The description of the right-most column seems to overlap for the first line.
8. Regarding proteomic sequencing, I would ask the authors to nuance this a bit. This approach is not yet readily available and is likely to take several more years until it might be applied outside of specialized labs or as proof-of-principle. It seems that mass spectrometry based proteomics will remain the norm for the near future. To me, the text currently reads as if proteomic sequencing had entered the "mainstream", which is not the case.

Methods

Are the methods appropriate to the aims of the study, are they well described, and are necessary controls included? Choose an item.

Conclusions

Are the conclusions adequately supported by the data shown? Choose an item.

Reporting Standards

Does the manuscript adhere to the journal's guidelines on [minimum standards of reporting?] Choose an item.

Choose an item.

Statistics

Are you able to assess all statistics in the manuscript, including the appropriateness of statistical tests used? Choose an item.

Quality of Written English

Please indicate the quality of language in the manuscript: Choose an item.

Declaration of Competing Interests

Please complete a declaration of competing interests, considering the following questions:

- Have you in the past five years received reimbursements, fees, funding, or salary from an organisation that may in any way gain or lose financially from the publication of this manuscript, either now or in the future?
- Do you hold any stocks or shares in an organisation that may in any way gain or lose financially from the publication of this manuscript, either now or in the future?
- Do you hold or are you currently applying for any patents relating to the content of the manuscript?
- Have you received reimbursements, fees, funding, or salary from an organization that holds or has applied for patents relating to the content of the manuscript?
- Do you have any other financial competing interests?
- Do you have any non-financial competing interests in relation to this paper?

If you can answer no to all of the above, write 'I declare that I have no competing interests' below. If your reply is yes to any, please give details below.

I declare that I have no competing interests.

I agree to the open peer review policy of the journal. I understand that my name will be included on my report to the authors and, if the manuscript is accepted for publication, my named report including any attachments I upload will be posted on the website along with the authors' responses. I agree for my report to be made available under an Open Access Creative Commons CC-BY license (http://creativecommons.org/licenses/by/4.0/). I understand that any comments which I do not wish to be included in my named report can be included as confidential comments to the editors, which will not be published.

I agree to the open peer review policy of the journal

To further support our reviewers, we have joined with Publons, where you can gain additional credit to further highlight your hard work (see: https://publons.com/journal/530/gigascience). On publication of this paper, your review will be automatically added to Publons, you can then choose whether or not to claim your Publons credit. I understand this statement.

Yes Choose an item.
