# Supplementary material for: Promises and pitfalls of long-read sequencing for resolving microbial complexity
Source: Gigascience. 2026 Aug 17;15:giag087. doi: 10.1093/gigascience/giag087 (PMC13543314; doi:10.1093/gigascience/giag087)
Supplement: Supplementary file 9 [file giag087_reviewer_2_report_revision_1.pdf]

Reviewer Report

Title: Promises and Pitfalls of Long-Read Sequencing for Resolving Microbial Complexity

Version: Revision 1 **Date:** 6/30/2026

Reviewer name: Reviewer 2

Reviewer Comments to Author:

Summary: In this revised version of their review manuscript on long-read sequencing for resolving microbial complexity, Rao et al. implemented my comments to a limited extent.

Major comments:

1. Despite the authors' claim to have reduced redundancy, many parts still read repetitive. Notably, the point about reduced fragmentation due to longer reads being used in genomic assembly is coming up again and again. Often even as the main point, thus adding little new information. After all, this is what long read sequencing was developed for. This point also only differs marginally between isolate cultures and microbiomes. Meta-assemblers provide additional solutions for the difference in coverage but otherwise are highly similar to "non-meta" assemblers. Moreover, long reads hold potential beyond contigs, i.e., due to their length, relevant functional information covering neighboring genes could be recovered. As one early concrete example of repetitiveness, take L64+L65 and L72ff. Basically, L72ff are examples of "intricate genomic architecture". However, the reading flow is disrupted and comes back on a previous point. This is persistent throughout the manuscript.
2. I find that the points I raised have been addressed rather superficially. My comment on "haplotype parsing" was apparently only considered for Figure 2. However, uses in the text remain unchanged. Capitalization is inconsistent, notably in Figure 1 and Figure 4. As listed below several minor comments remain. The references seem not to have been checked thoroughly, e.g., L958 "48. . !!! INVALID CITATION !!! [45]."
3. The review requires a certain domain knowledge to be accessible to the readers. The authors refer to several things that are not explained, e.g., de Bruijn graph-based assembly vs. OLC assembly. This limits the accessibility to the uninformed reader.
4. This is up to the journal, but I could not find where the authors comply with the requirement of "A summary of use (particularly in the introduction or among methods) needs to be included at the end of the paper, and the outputs should also be included as a supplementary file hosted in GigaDB or other open repositories." The authors only mention that Gemini 3.1 Pro and what for, but not how or what the outputs were.

L60: Why is DNBSEQ listed here, representing the arguably most recent technology in the field? Other approaches, such as by SOLID or IonTorrent, predate DNBSEQ by many years. Hence presenting it in this way is not logical to me.
L79: Where is "MGE" introduced?
L88: Remove "the" from "the microbiological"
L98: What do the authors mean by "integrative perspectives"?
L106: Not sure the "single" is necessary.

L134: Not sure what is meant by "high accuracy read per base"?
L135: What is specifically meant by "complex regions"? What qualifies as such?
L157: The approaches were not "focused" on drafts but there was simply no better solution readily available. The reference to "finished gapless" is redundant with what follows ("structural completeness").
L204: Consider removing "where SRS alignment …" as it is redundant with all the rest.
L249: What do the authors mean by "inference" here?
L267: Not sure what the "moving away beyond taxonomic inventories" brings in here. This applies to SRS as well as LRS.
L298: Why are SRS binning strategies "prone to missing low-abundance" but LRS binning strategies are not? In fact, LRS has a much lower sequencing depth, which is a considerable challenge for low-abundance organisms.
L301: Long-read metagenomic sequencing does not "generate" "molecules"; it generates sequences.
L318: Not sure what is mean by "converted" in this context.
L326: "parsing" remains used.
L328: Wrong capitalization at start of sentence.
L425: I do not understand how LRS connects "function to mechanism". If at all, the authors show that mechanism is hard to get to, not least due to epigenetic/posttranscriptional/posttranslational modifications.
L431: Formatting of heading missing.
L435: Real-time sequencing is a particular feature of Oxford Nanopore Technologies approach, but not common to LRS. Please adjust accordingly.
L442: What does "mNGS" mean?
L486: Why is "core" needed here?
L491: Why is that argument brought up? SRS was never designed to provide single-molecule solution; at least no SRS solution got meaningful adoption/market share for that.
L515: "HMW" not introduced.
L534: "TSS" and "TTS" not introduced.
L540: What is meant by "defined" here?
L569: Why "intact", when it already include "HMW" in the sentence? Importantly, how likely is it to recover fully intact DNA, i.e., a full genome as a single, intact molecule?
L691: Please use "cMAGs" to be consistent.
L699: "AI" not introduced. Importantly, assuming "Artificial Intelligence" is meant here, this is a very generic term.
L766f: Not sure why "gapless" is needed in addition to "completeness".
L768: Why is "microbial" added here when referring to metagenomics?

Methods

Are the methods appropriate to the aims of the study, are they well described, and are necessary controls included? Choose an item.

Conclusions

Are the conclusions adequately supported by the data shown? Choose an item.

Reporting Standards

Does the manuscript adhere to the journal's guidelines on [minimum standards of reporting?]() Choose an item.

Choose an item.

Statistics

Are you able to assess all statistics in the manuscript, including the appropriateness of statistical tests used? Choose an item.

Quality of Written English

Please indicate the quality of language in the manuscript: Choose an item.

Declaration of Competing Interests

Please complete a declaration of competing interests, considering the following questions:

- Have you in the past five years received reimbursements, fees, funding, or salary from an organisation that may in any way gain or lose financially from the publication of this manuscript, either now or in the future?
- Do you hold any stocks or shares in an organisation that may in any way gain or lose financially from the publication of this manuscript, either now or in the future?
- Do you hold or are you currently applying for any patents relating to the content of the manuscript?
- Have you received reimbursements, fees, funding, or salary from an organization that holds or has applied for patents relating to the content of the manuscript?
- Do you have any other financial competing interests?
- Do you have any non-financial competing interests in relation to this paper?

If you can answer no to all of the above, write 'I declare that I have no competing interests' below. If your reply is yes to any, please give details below.

I declare that I have no competing interests.

I agree to the open peer review policy of the journal. I understand that my name will be included on my report to the authors and, if the manuscript is accepted for publication, my named report including any attachments I upload will be posted on the website along with the authors' responses. I agree for my

report to be made available under an Open Access Creative Commons CC-BY license (http://creativecommons.org/licenses/by/4.0/). I understand that any comments which I do not wish to be included in my named report can be included as confidential comments to the editors, which will not be published.

I agree to the open peer review policy of the journal

To further support our reviewers, we have joined with Publons, where you can gain additional credit to further highlight your hard work (see: https://publons.com/journal/530/gigascience). On publication of this paper, your review will be automatically added to Publons, you can then choose whether or not to claim your Publons credit. I understand this statement.

Yes Choose an item.
