# Supplementary material for: Promises and pitfalls of long-read sequencing for resolving microbial complexity
Source: Gigascience. 2026 Aug 17;15:giag087. doi: 10.1093/gigascience/giag087 (PMC13543314; doi:10.1093/gigascience/giag087)
Supplement: Supplementary file 11 [file giag087_reviewer_3_report_revision_2.pdf]

Reviewer Report

Title: Promises and Pitfalls of Long-Read Sequencing for Resolving Microbial Complexity

Version: Revision 2 **Date:** 8/12/2026

Reviewer name: Reviewer 3

Reviewer Comments to Author:

Reviewer #2 requested major revision. Most of those points are now handled. A few prior concerns are still incomplete, as listed below.

What has been adequately addressed

Major comment 1 (repetitiveness). The Introduction is reorganized so that SRS limitations are grouped before LRS is introduced. Several redundant sentences were cut or shortened (including the earlier Koren et al. discussion and the repeated draft-vs-complete contrast). The text now spends more time on plasmid/phage context, ARG localization, and strain variation, rather than repeating that long reads reduce fragmentation. Some residual wording remains (the Abstract still opens with assembly fragmentation; section 2.1 is still titled "Achieving gapless genomes: from fragmentation to continuity"), but this is no longer the same problem raised last round. I consider this comment adequately addressed.

Major comment 3 (undefined terminology). The de Bruijn vs OLC distinction is now explained in Section 2. Brief definitions were also added for SNVs, SVs, Q30/Q40, and the main methylation types (6mA, 5mC, 4mC). This comment is addressed.

Line-level comments. The specific wording changes claimed in the response letter are present in the revised text, including: DNBSEQ listed as an example rather than a chronological first; MGE defined at first use; "integrative perspectives" replaced; "single-isolate" dropped; "high accuracy read per base" removed; Sheppard et al. rewritten without "inference"; the unsupported SRS-vs-LRS low-abundance binning claim deleted; "converted" removed; remaining "parsing" wording replaced in the Abstract, Section 3.2 heading, and Conclusion; real-time sequencing limited to ONT; mNGS spelled out; HMW/TSS/TTS introduced; "intact" removed from the HMW DNA sentence; "AI" replaced with machine-learning/deep-learning; and the redundant "gapless" wording removed from the Conclusion. These items are addressed.

Prior concerns that remain insufficiently addressed

1. Major comment 4 (AI-use disclosure / journal compliance). This is the main leftover issue. Reviewer #2 asked for a proper summary of use and for the outputs to be provided as required by the journal. The revised disclosure is still only two sentences: Gemini 3.1 Pro was used for language checking and wording suggestions, and no AI-generated content was incorporated without author review. That still does not say how the tool was used, which sections were edited, or what the outputs were. There is still no supplementary file. Reference 145 is also incomplete ("Google (2026). x (3.1 Pro)"), so the required model/version citation is not in usable form. If the authors maintain that only language polishing occurred and that no output needs to be deposited, they should state this explicitly, list the users, model, version and date in the disclosure itself, and correct reference 145. If any AI text or figure output was generated, that material still needs to be deposited as previously requested.

2. Major comment 2, remaining consistency items. "Parsing" has been removed from the main text, but Figure 2 still says "High-resolution haplotype phasing" without defining the term. That is the same accessibility/terminology problem Reviewer #2 raised. Please either define haplotype phasing in the figure legend or use the same "strain-level resolution" wording already adopted in the text. Also, Reviewer #2 asked for a thorough reference check after the invalid citation. The invalid citation is gone, but the broken "x (3.1 Pro)" entry shows the reference list still needs a final pass. I could not inspect the figure graphics themselves in the Word file provided, so I cannot confirm that the capitalization issues in Figures 1 and 4 were fully corrected; please make sure this was done in the submitted figure files.

Methods

Are the methods appropriate to the aims of the study, are they well described, and are necessary controls included? Choose an item.

Conclusions

Are the conclusions adequately supported by the data shown? Choose an item.

Reporting Standards

Does the manuscript adhere to the journal's guidelines on [minimum standards of reporting?]() Choose an item.

Choose an item.

Statistics

Are you able to assess all statistics in the manuscript, including the appropriateness of statistical tests used? Choose an item.

Quality of Written English

Please indicate the quality of language in the manuscript: Choose an item.

Declaration of Competing Interests

Please complete a declaration of competing interests, considering the following questions:

- Have you in the past five years received reimbursements, fees, funding, or salary from an organisation that may in any way gain or lose financially from the publication of this manuscript, either now or in the future?
- Do you hold any stocks or shares in an organisation that may in any way gain or lose financially from the publication of this manuscript, either now or in the future?

- Do you hold or are you currently applying for any patents relating to the content of the manuscript?
- Have you received reimbursements, fees, funding, or salary from an organization that holds or has applied for patents relating to the content of the manuscript?
- Do you have any other financial competing interests?
- Do you have any non-financial competing interests in relation to this paper?

If you can answer no to all of the above, write 'I declare that I have no competing interests' below. If your reply is yes to any, please give details below.

I declare that I have no competing interests.

I agree to the open peer review policy of the journal. I understand that my name will be included on my report to the authors and, if the manuscript is accepted for publication, my named report including any attachments I upload will be posted on the website along with the authors' responses. I agree for my report to be made available under an Open Access Creative Commons CC-BY license (http://creativecommons.org/licenses/by/4.0/). I understand that any comments which I do not wish to be included in my named report can be included as confidential comments to the editors, which will not be published.

I agree to the open peer review policy of the journal

To further support our reviewers, we have joined with Publons, where you can gain additional credit to further highlight your hard work (see: https://publons.com/journal/530/gigascience). On publication of this paper, your review will be automatically added to Publons, you can then choose whether or not to claim your Publons credit. I understand this statement.

Yes Choose an item.
